# Trilocular phenotype in *Brassica juncea* L. resulted from interruption of *CLAVATA1* gene homologue (*BjMc1*) transcription

**DOI:** 10.1038/s41598-017-03755-0

**Published:** 2017-06-14

**Authors:** Ping Xu, Shiqin Cao, Kaining Hu, Xiaohua Wang, Wei Huang, Gang Wang, Zewen Lv, Zhongsong Liu, Jing Wen, Bin Yi, Chaozhi Ma, Jinxing Tu, Tingdong Fu, Jinxiong Shen

**Affiliations:** 1 National Key Laboratory of Crop Genetic Improvement and National Engineering Research Center of Rapeseed, Huazhong Agricultural University, Wuhan, 430070 China; 2 Oil Crops Research Institute, Hunan Agricultural University, Changsha, 410128 China

## Abstract

As a desirable agricultural trait, multilocular trait of rapeseed (*Brassica rapa*; *Brassica napus*; *Brassica juncea*), always represents higher yield per plant compared with bilocular plants. We previously isolated a trilocular gene locus, *Bjmc1*, and identified a set of molecular markers linked to the trilocular gene. With a map-based cloning, we identified that the *BjMc1* was located in B genome of *Brassica juncea*, and it was a *CLAVATA1* (*CLV1*) gene homologue. The insertion of a copia-LTR retrotransposable element 1 (RTE1) into the coding region of *BjMc1* interrupted its transcription in rapeseed, leading to the trilocular phenotype. Phylogenetic analysis showed that *Mc1* genes were conserved and widespread in land plants. Two amino acid sites had undergone positive selection in the ancestor of *Mc1* genes, and then purifying selection was the dominant force after the divergence of dicots and monocots from their common ancestor in the evolutionary process, indicating that *Mc1* genes are conserved in modern land plants. Our results provided new insights in molecular regulatory mechanism of multilocularity in rapeseed, and better understanding of molecular mechanism in crop yield improvement.

## Introduction

Upon germination, the shoot apical meristem (SAM) produces primary leaves, and after the vegetative-to-reproductive switch, it is transformed into inflorescence meristem, and then floral meristem forms on inflorescence meristem, which generates floral organs, including sepals, petals, stamens and carpels^1^. In rapeseed (*Brassica rapa*; *Brassica napus*; *Brassica juncea*), morphological studies have shown that the wild-type (bilocular) silique development starts when two carpels initiate from the floral meristem to form a hollow cylinder of cells which grow apically to generate the gynoecium. Simultaneously, two medial ridges grow through directed cell division towards each other to fuse and create the septum. The septum divides the cylinder into two locules corresponding to the original two carpels. However, the multilocular gynoecium consists of more than two carpels and the multilocular silique usually has more than three locules^2, 3^.

The silique trait of rapeseed directly affects the seed yield, and yield enhancement has always been one of the major goals of rapeseed production and genetic improvement^4–6^. Multiloculus, leading to a slightly fewer silique per plant but containing much more seeds per silique than biloculus with similar seed size, has been considered a great potential for developing high-yield rapeseed varieties^7^. However, most rapeseed varieties planted worldwide are bilocular. Classical genetic analysis demonstrated that two recessive nuclear genes controlled the multilocular trait in *B*. *juncea*, and one recessive nuclear gene was responsible for the multilocular trait in *B*. *rapa* ^8, 9^. A series of molecular markers linked to the multilocular gene locus had been identified in *B*. *juncea* ^10^, which could promote the utilization of multilocularity in breeding of high-yield cultivars.

The multilocular trait in *B*. *rapa* was found to be controlled by a *CLAVATA3* (*CLV3*) gene homologue, and it suggested that the feedback loop involving *CLV3* and *WUSCHEL* (*WUS*) played a major role in carpel development^11^. Multilocular trait was also observed and genetically investigated in *Arabidopsis* ^12–15^ and tomato^16^. In *Arabidopsis*, *CLAVATA* (*CLV*) signaling pathway regulating *WUS* expression is a key component of the network that controls stem cell renewal and differentiation, and the previously described mutants, such as *clv1*, *clv2*, *clv3* and *crn*, showed the phenotype of multilocular siliques due to the disturbance of stem cell growth balance^17, 18^. The *sqn* mutant showed multiloculus, and *SQN* was found to be required for the normal accumulation of various miRNAs, indicating that miRNAs might be involved in the regulation of silique trait^19^. In addition, a recent study showed that another receptor kinase signaling pathway involving *ERECTA* (*ER*) regulated the stem cell growth, and the *er* mutant exhibited a similar silique trait with *clv* mutants^20^. In tomato, the mutation of the homologues of both *CLV1* and *CLV3* resulted in the increased number of fruit locules. Moreover, the mutation of the homologous genes in *CLV* signaling pathway in maize^21, 22^ and rice^23^, such as *THICK TASSEL DWARF1*, *FASCIATED EAR2* and *FLORAL ORGAN NUMBER1*, could also the increase the seed number per inflorescence, which was similar to multilocular trait in rapeseed.

As a member of *CLV* signaling pathway that regulates *WUS* expression, more than 10 alleles of *clv1* with multiloculus have been discovered in *Arabidopsis*, and the mutants exhibited weak, intermediate and strong multilocular phenotypes^12, 24, 25^. The *CLV1* gene encoded a putative receptor kinase^26^, and mutation at different sites in the gene sequence could lead to different degrees of multilocular phenotype. Moreover, all the *clv1* mutants with intermediate and strong multilocular phenotypes were dominant negative, and the mutant of *CLV1* homologous gene in tomato was also expected to be dominant-negative^16, 27^. Similarly, most of the mutants with multiloculus discovered in *Brassicas* showed variable valve numbers^10, 11^. However, it has been unknown whether the molecular mechanism controlling multiloculus is similar between *Arabidopsis* and *Brassicas* or the dominant-negative character is associated with the instability of multilocular silique trait.

In previous study, we found that the trilocular silique always had three locules, and the trilocular plants had significantly higher yield per plant than the bilocular plants^2^. The trilocular trait of *B*. *juncea* was controlled by two independent recessive nuclear genes, *Bjmc1* and *Bjmc2*. The *BjMc1* and *BjMc2* gene were isolated from the same plants and mapped by molecular markers^28, 29^. In present study, we cloned the bilocular gene *BjMc1* and trilocular *Bjmc1* respectively. A Copia-LTR retrotransposable element 1 (RTE1) inserted in the coding region of *Bjmc1* was identified in trilocular plants, which interrupted the transcription of the target gene. We also found that two amino acid sites had undergone positive selection in the ancestor of *Mc1* genes, and purifying selection was the dominant force after divergence of dicots and monocots from their common ancestor in the evolutionary process of *Mc1* genes, indicating that they were conserved in modern land plants.

## Results

### Fine mapping of *BjMc1* gene

**Figure 1:**
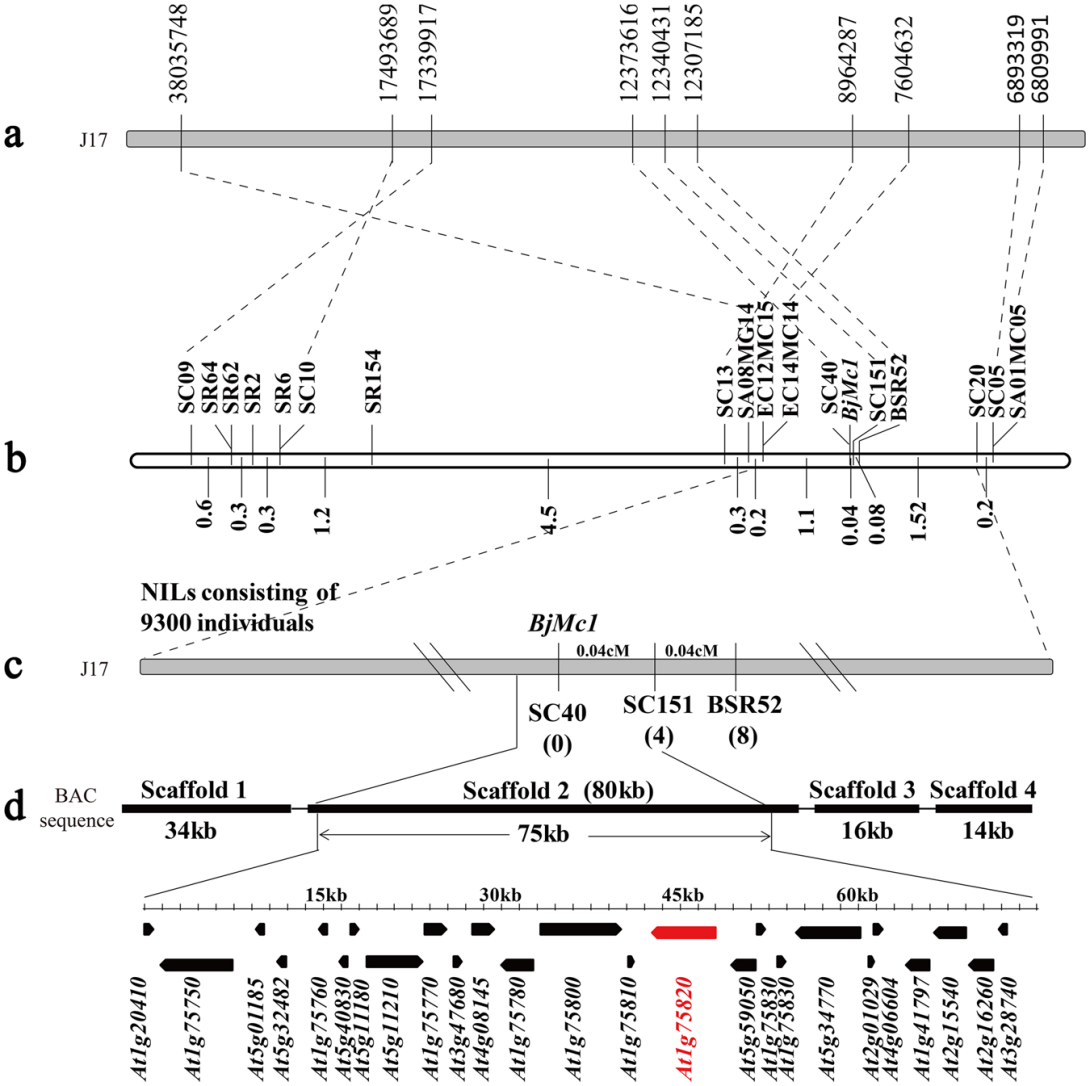
Map-based cloning of *BjMc1*. The number below the marker indicates the number of recombinants between individual markers and *BjMc1* locus. The pentagons represent the predicted genes in the 75-kb target region on chromosome J17 of *B*. *juncea*. The candidate gene of *BjMc1* is indicated by red color. (**a**) The physical location of molecular markers of *BjMc1* on J17 chromosome. (**b**) The genetic linkage map of *BjMc1*. (**c**) Genetic distance of the three markers identified in this research. (**d**) the scaffolds of BAC clone 83D02.

*BjMc1* gene was previously mapped to a genomic region between marker EC14MC14 and SC20, which could delimit an interval of 2.7 cM^28^. Compared with the bilocular siliques in NILs of *BjMc1* gene, the trilocular siliques displayed shorter, wider and flatter (Supplementary Fig. 1a,b). But the inflorescence meristem and floral meristem did not show differences between bilocular and trilocular plants in BC_6_F_1_ generation (Supplementary Fig. 2a,b). To identify the *BjMc1* gene locus, we further screened a BAC library of purple-leaf mustard with the primer C1-1 (Supplementary Table 1). Two positive clones, 26P20 and 83D02, were identified, and four scaffolds (designated as scaffold 1, 2, 3 and 4) (Supplementary Data 1) were obtained by sequencing 83D02. Two sequence-characterized amplified region markers SC40 and SC151 (Supplementary Table 1) were identified according to scaffold 2, and one SSR marker SR52 was identified based on scaffold 4. Subsequently, polymorphic markers SC40, SC151 and SR52 were used to search for the recombinants identifiable between SC13 and SC20. Among the 242 recombinants discovered from the NILs population which consisted of 9,300 individuals, 0, 4 and 8 recombination events were detected for SC40, SC151 and SR52 respectively. Finally, the candidate region of *BjMc1* was found to be restricted between SC151 and EC14MC14 at a 1.14-cM region. Twenty-five open reading frames (ORFs) of scaffold 2 in which both the co-segregative SC40 and nearest marker SC151 located were predicted according to: (http://linux1.softberry.com/berry.phtml?topic=fgenesh&group=programs&subgroup=gfind), and were annotated by *Arabidopsis* genes, including a *CLV1* gene (*At1g75820*) (Fig. 1). For each of the 25 ORFs, we comparatively sequenced the genomic fragments covering the promoter region and the complete coding region from both the bilocular and trilocular plants in BC_5_F_1_ population. The results showed that only the predicted gene which was homologous to *CLV1* (*At1g75820*) showed sequence variation. Moreover, the co-segregation molecular marker SC40 was just located in the ORF homologous to *CLV1*. Thus, the *CLV1* homologous gene was selected as the candidate gene for further investigation.

### *Bjmc1* gene transcription of trilocular plants was interrupted by a Copia-LTR RTE1

**Figure 2:**
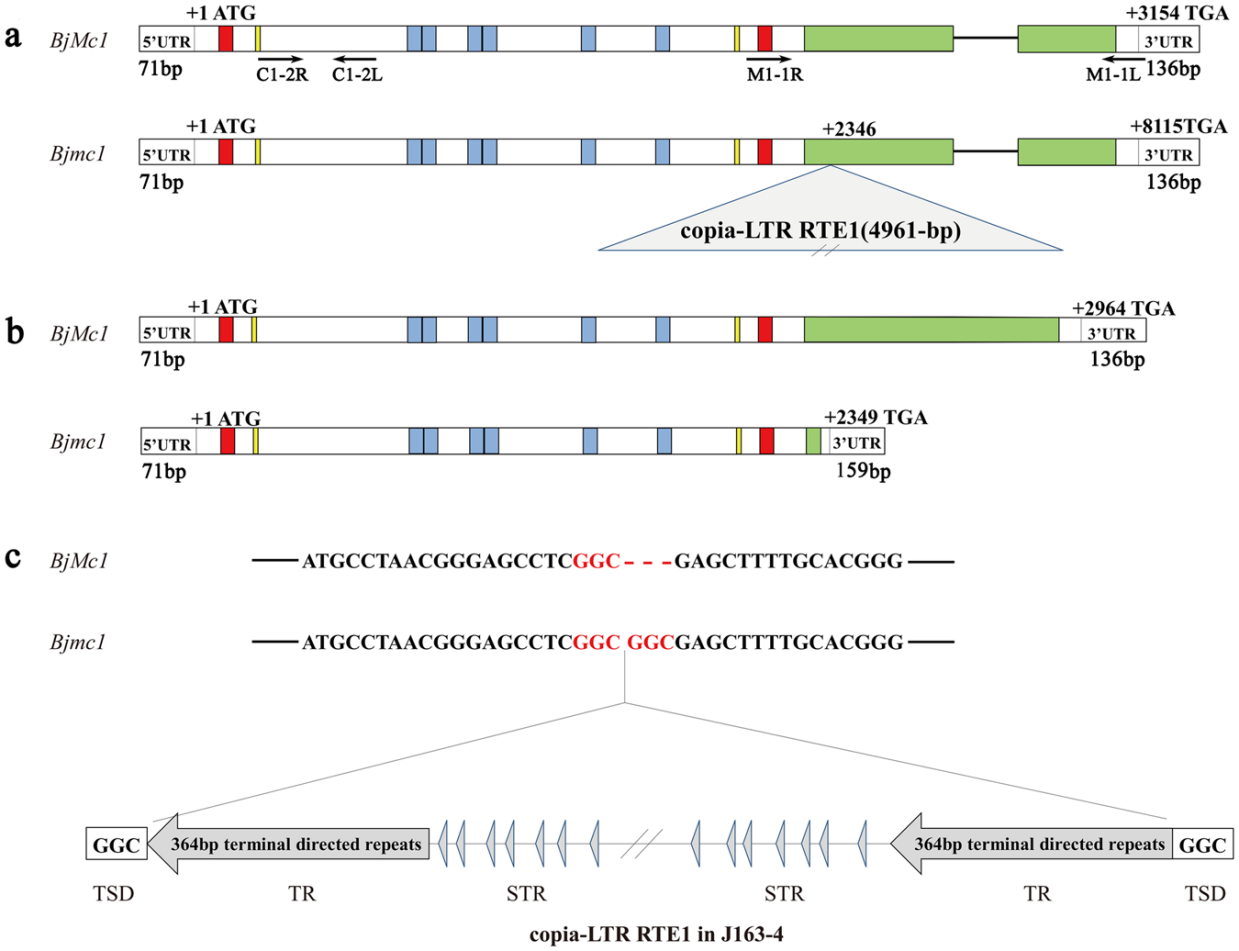
Gene structure of *BjMc1* in bilocular and *Bjmc1* in trilocular plants. Red box () represents the putative transmembrane domain, yellow box () represents the cysteine domain, blue box () represents the LRR domain, green box () represents the serine/threonine protein kinase. (**a**) Genomic gene structure. The arrows indicate the binding sites of primers (**b**) cDNA gene structure. (**c**) Structure of the copia-LTR RTE1 found in the *Bjmc1* genomic gene.

**Figure 3:**
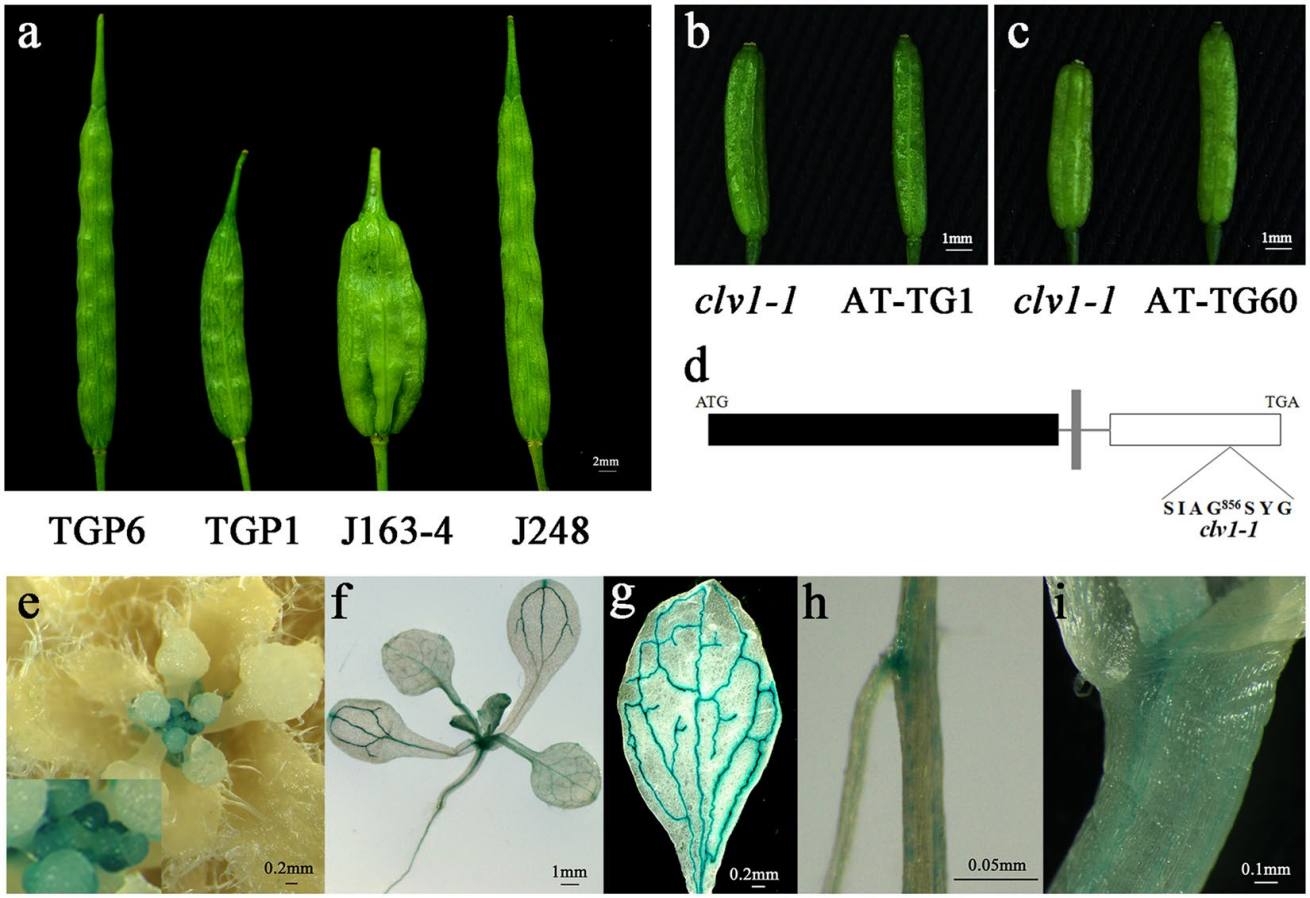
Functional analysis and expression pattern of *BjMc1*. (**a**) Silique phenotypes of *p35S*::*BjCLV1b*-transgenetic T_0_ line TGP6, *pBjCLV1b*:*BjCLV1b-*transgenetic T_0_ line TGP1, multilocular parental J163-4 and bilocular parental J248. (**b**) Silique phenotypes of *Arabidopsis* mutant *clv1-1* and *pBjCLV1b*:*BjCLV1b*-transgenetic T0 line AT-TG1. (**c**) Silique phenotypes of *Arabidopsis* mutant *clv1-1* and *35S*::*BjCLV1b*-transgenetic T0 line AT-TG60. (**d**) The mutation sites of *clv1-1*. (**e**–**i**) Representative histochemical analysis of GUS expression in tissues under the control of the *BjMc1* promoter in the transgenic *Arabidopsis* T_2_ plants. (**e**) Early inflorescence with flower bud meristems. The solid arrow indicates the flower bud meristems on the early inflorescence (**f**) Seedling 10 d after germination. (**g**) Rosette leaf. (**h**) Root. (**i**) Stem.

A pair of primer CV4 (Supplementary Table 1) was designed based on the conserved reference sequences of *Bra015812*, *AT1G75820* and scaffold 2 of 83D02. Two homologous copies, respectively designated as *BjCLV1a* and *BjCLV1b*, were detected in bilocular plants of *B*. *juncea*, and the sequences were shown in Supplementary Data 2. No sequence variation was detected in *BjCLV1a* between bilocular and trilocular plants, while an insertion of a 4961 bp Copia-LTR RTE1 was detected in the coding region of *BjCLV1b* of trilocular plants (Fig. 2a), and the sequence of RTE1 was showed in Supplementary Data 3. The sequence analysis further indicated that the putative Copia-LTR RTE1 had a 3 bp target site duplication (GGC), 364 bp terminal directed repeats and a number of subterminal repeats (ATCAGCGACT) (Fig. 2c). To validate the candidacy of *BjCLV1b* for *BjMc1* locus, two constructs *p35S*::*BjCLV1b* and *pBjCLV1b*:*BjCLV1b* were transformed into the J163-4 plants. A total of 27 *pBjCLV1b*:*BjCLV1b-*transgenetic plants and 20 *p35S*::*BjCLV1b*-transgenetic plants were obtained in T0 generation. 18 out of the 27 *pBjCLV1b*:*BjCLV1b-*transgenetic plants showed chimeric phenotype, which showed 2–6 bilocular siliques besides the trilocular ones in one plant. By contrast, 16 out of the 20 *p35S*::*BjCLV1b*-transgenetic plants showed completely bilocular siliques (Fig. 3a). The phenotypes and genotypes of four *pBjCLV1b*:*BjCLV1b-*transgenetic and five *p35S*::*BjCLV1b*-transgenetic lines were analyzed in T1 progeny of plants. As shown in Supplementary Table 2, in all five *p35S*::*BjCLV1b*-transgenetic lines, the transgenic plants with completely bilocular siliques were observed. By contrast, only one of the four *pBjCLV1b*:*BjCLV1b-*transgenetic lines showed a T1 progeny plant with bilocular silique. The expression levels of *BjCLV1b* in early inflorescence of 4 transgenic lines with *pBjCLV1b*:*BjCLV1b* (TGP1-4), 11 transgenic lines with *p35S*::*BjCLV1b* (TGP5-15), and the bilocular and trilocular plants of BC_5_F_1_ generation were detected by qPCR using the primer DL2. As expected, compared with the bilocular plants of BC_5_F_1_ generation, the *p35S*::*BjCLV1b*-transgenic plants showed much higher expression level of *BjMc1*, while the *pBjCLV1b*:*BjCLV1b*-transgenic plants exhibited similar or lower expression level (Supplementary Fig. 3a).

**Figure 4:**
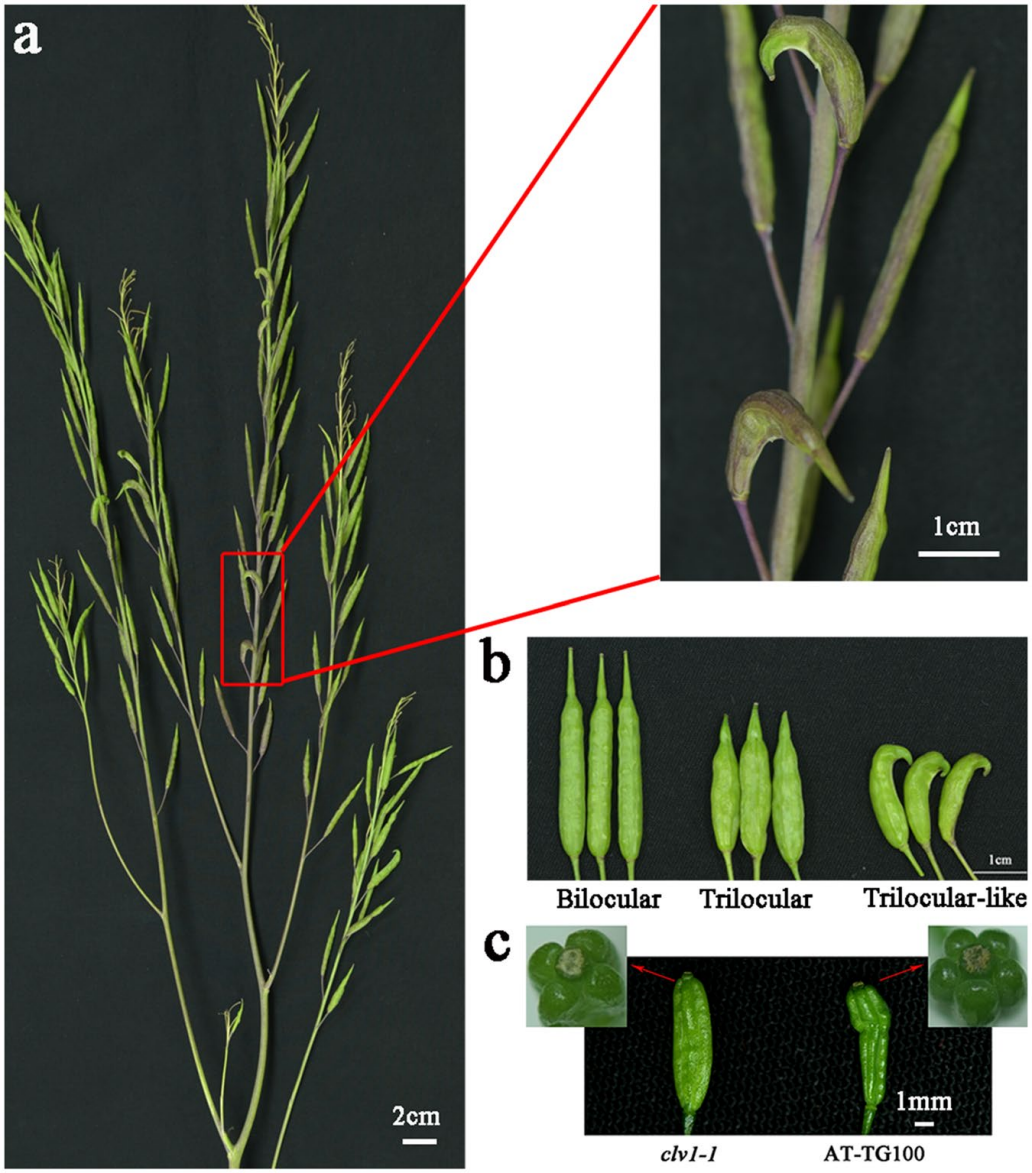
Dominant negative phenotype. (**a**) Silique phenotype of the bilocular plant in BC_5_F_1_ generation. (**b**) Bilocular silique, trilocular silique and trilocular-like silique. (**c**) Silique phenotypes of Arabidopsis mutant *clv1-1* and *p35S*::*bjmc1*-transgenetic T0 line AT-TG100.

In addition, the constructs of *p35S*::*BjCLV1b* and *pBjCLV1b*:*BjCLV1b* were transformed respectively into *Arabidopsis* multilocular mutant *clv1-1* which showed four valves per silique (Fig. 4c). A total of 56 *pBjCLV1b*:*BjCLV1b-*transgenetic plants and 32 *p35S*::*BjCLV1b*-transgenetic plants were obtained in T0 generation. 33 out of the 56 *pBjCLV1b*:*BjCLV1b-*transgenetic plants showed chimeric phenotype, while 17 out of the 32 *p35S*::*BjCLV1b*-transgenetic plants showed completely bilocular phenotype (Fig. 3b,c). Further analysis of phenotypes and genotypes confirmed that the transgenic events were cosegregated with the bilocular trait in T1 progeny.

### *Bjmc1* encoded a putative truncated protein

Semi-quantitative reverse transcription polymerase chain reaction (RT-PCR) was performed to analyze the transcription levels of *BjMc1* and *Bjmc1* of early inflorescences of bilocular and trilocular plants in BC_5_F_1_ generation. C1-2 and M1-1(Supplementary Table 1) were designed according to different parts of the gene sequence (Fig. 2a). Both C1-2 and M1-1 could identify the transcription of *BjMc1* in bilocular plants, while only C1-2 could identify the transcription of *Bjmc1* in trilocular plants, indicating that the truncation of transcription has occurred in *Bjmc1* gene. Although the transcription of *BjMc1* and *Bjmc1* could be detected by C1-2, the *Bjmc1* expression was down-regulated in trilocular plants (Supplementary Fig. 1i).

To investigate the transcription level of *BjMc1* and *Bjmc1* in detail, the full-length complementary DNAs (cDNAs) were identified by RACE technology. The cDNA of *BjMc1* was 3,155 bp in length, which was composed of two exons and consists of a 71 bp 5′ untranslated region, a 2,964 bp ORF and a 136 bp 3′ untranslated region. The cDNA of *Bjmc1*, with a full-length of 2,583 bp, comprised a 71 bp 5′ untranslated region which contained the same sequence with the cDNA of *BjMc1*, a 2,349 bp ORF and a 159 bp 3′ untranslated region (Fig. 2b and Supplementary Data 4). *BjMc1* encoded a putative protein of 987 amino acids and contained two putative transmembrane domains, a putative extracellular domain consisting of 6 complete Leu-rich repeats (LRRs) and a putative intracellular domain containing all of the conserved residues found in serine/threonine protein kinase. The LRR region was flanked by a pair of conservatively spaced cysteines (Fig. 2a,b). Sequence analysis indicated that the putative *Bjmc1* protein consisting of 782 amino acids contained the extracellular domain of *BjMc1*, and further analysis showed that *Bjmc1* protein was derived from the truncation of *BjMc1* gene at Gly^782^ by Copia-LTR RTE1 (Fig. 2b and Supplementary Fig. 3c), proving that the serine/threonine protein kinase domain of *BjMc1* gene was required for the gene to control the development of siliques in *B*. *juncea*.

### The dominant negative effect of *Bjmc1*

In back-cross populations, we observed that some bilocular individuals (*BjMc1bjmc1bjmc2bjmc2*) showed several triloculus-like siliques which showed trilocular shape but with two locules and only a few siliques had three locules (Fig. 4a,b). The silique trait of 219 bilocular plants was investigated in BC_8_F_1_ generation, and 92 individuals were found to have triloculus-like siliques.

A previous report has demonstrated that all intermediate and strong *clv1* alleles in *Aribidopsis* are dominant negative^27^. Similar to the homozygous mutant of *CLV1* homologous gene, the heterozygous plants also exhibited weak fasciation in tomato^16^. Moreover, the *Arabidopsis ER*, a Leu-rich repeat receptor-like Ser/Thr kinase, regulated organ shape and inflorescence architecture, and a truncated *er* protein that lacks the cytoplasmic kinase domain confers dominant-negative effects^30^. In our research, *Bjmc1* was shown to be able to encode a truncated protein with a similar structure with *er* protein, indicating the possibility of dominant negative effect. To verify whether *Bjmc1* protein functions in the development process of carpel, the construct *p35S*::*Bjmc1* was transformed into *clv1*-*1* mutant. A total of 62 transgenic plants were obtained and 25 of them showed siliques with five valves (Fig. 4c). The phenotype analysis of T_1_ progeny showed that the transgenic events could be cosegregated with the phenotype of five valves. The over-expression of *Bjmc1* could aggravate the multilocular phenotype of *clv1-1*. These results further supported the inference that the truncated *Bjmc1* protein showed the dominant negative character in controlling carpel development.

### Expression pattern and subcellular localization of *BjMc1*

To investigate the expression pattern of *BjMc1*, quantitative real-time PCR (qPCR) analysis was performed using the total RNA extracted from various organs of bilocular plants in BC_5_F_1_ generation. The results indicated that the highest expression level was detected in early inflorescence, but overall lower and variable expression levels were detected in different developmental periods of siliques. The expression level of *BjMc1* was relatively high in SAM, the stem and the root, but it was much lower in the silique peel and the leaf (Supplementary Fig. 3b). To examine *BjMc1* activity in greater detail, the expression of GUS in the transgenic plants was detected in the flower bud primordium, the SAM of early inflorescence and mainly in the vascular tissues of other organs, including cotyledons, rosette leaves, stems and roots. But with the development of flower buds, GUS staining faded away in the transgenic plants (Fig. 3e–i). Consistent with qPCR analysis, the flower bud primordium in early inflorescence showed the highest GUS expression. These results proved that the *BjMc1* gene was expressed in the flower bud primordium, indicating that the carpel number was determined at the stage of flower bud primordium formation in *B*. *juncea*.

To investigate the subcellular localization of *BjMc1*, we fused the coding region containing putative transmembrane domains and LRR domains, a without serine/threonine protein kinase of *BjMc1* with the coding region of an enhanced GFP driven by a double 35S promoter. This chimeric plasmid was transformed into *col-0 Arabidopsis*. The result showed that the *BjMc1*-GFP fusion protein was localized in the plasma membrane (Supplementary Fig. 1c–h), which was consistent with the previous study^31^ and suggested that *BjMc1* could retain some conserved functions of its homologs in other species.

### Expression analysis of genes involved in early inflorescence

**Figure 5:**
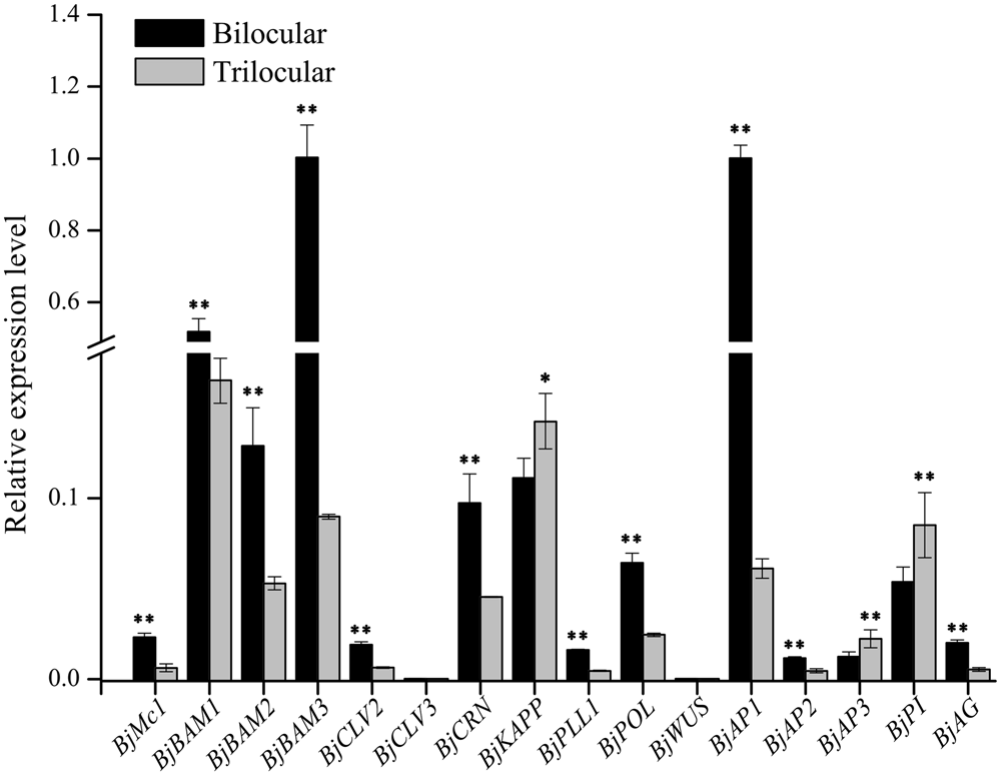
The difference of expression level of related genes between bilocular and trilocular plants during the flower bud differentiation period in BC_5_F_1_ generation. *P < 0.05; **P < 0.01.

According to the expression pattern of *BjMc1* gene, the homologous genes of *CLV* singling pathway, including *BjCLV3*, *BjWUS*, *BjCLV2*, *BjCRN*, *BjPOLL*, *BjPLL1*, *BjKAPP BjBAM1*, *BjBAM2* and *BjBAM3*, and the homologous genes of ABC model of floral organ identity, including *BjAP1*, *BjAP2*, *BjAP3*, *BjPI* and *BjAG*, were chosen to perform the qPCR analysis using the total RNA extracted from the early inflorescence of bilocluar and trilocular plants in BC_5_F_1_ generation, and primers (Supplementary Table 1) were design according to these homologous gene sequences. The results showed that, in trilocular plants, the A class genes, *BjAP1* and *BjAP2*, were down-regulated significantly, the B class genes, *BjAP3* and *BjPI* were up-regulated significantly, and the C class gene, *BjAG*, was also down-regulated significantly (Fig. 5). In trilocular plants, the *CLV* signaling pathway genes, *BjCLV2*, *BjCRN*, *BjPOLL*, *BjPLL*, *BjBAM1*, *BjBAM2* and *BjBAM3*, were down-regulated significantly, and *BjKAPP* was up-regulated. However, as two key components of *CLV* signaling pathway, *BjCLV3* and *BjWUS* did not show significant variations between bilocular and trilocular plants in early inflorescence (Fig. 5). These results indicated that *BjMc1* gene participated in *CLV* signaling pathway, but it was not the key pathway to control the carpel development in *B*. *juncea*. The mutant of *Bjmc1* gene in trilocular plants resulted in the significantly expression variation of ABC class genes, indicating that *BjMc1* involved in the pathway of flower bud formation to control the carpel development.

### *Mc1* genes being conserved and widespread in land plants

Blast searching against the plant database revealed that *Mc1* genes were widespread in land plants and each sequenced land plant genome contained at least one gene encoding *BjMc1* homologue. To explore the evolutionary process of *BjMc1* in land plants, we characterized CDSs and proteins of *Mc1* genes from species representing the main lineages of land plants, including moss *Physcomitrella patens*, lycophyte *Selaginella moellendorffii*, Amborellaceae *Amborella trichopoda*, 9 monocot species and 24 dicot species. The *Mc1* genes could be divided into three major branches on the phylogenetic tree (Supplementary Fig. 4), and *BjMc1* was in the branch of the Cruciferae species. Structural analysis of *Mc1* genes in land plants was performed by comparing the exon-intron organization (http://gsds.cbi.pku.edu.cn/). It was shown that the coding regions of all *Mc1* genes of land plants were interrupted by 1–2 introns except for *PpaMc1*, which contained 8 introns and 9 exons (Supplementary Fig. 5).

### Two amino acid sites underwent positive selection in the ancestors of *Mc1* genes

In this research, we attempted to further reveal the evolutionary process of *BjMc1* homologous genes in land plant species. The LRT of positive selection was applied using *codeML* method and the codon substitution models^32–34^, and all *Mc1* genes of sampled land plants were tested respectively. First, one-ratio model was used to determine whether there were variations in *d*_*N*_: *d*_*S*_ ratio of the codon position for *Mc1* genes in land plants. Overall, the maximum likelihood estimates for *d*_*N*_: *d*_*S*_ values were close to zero (Supplementary Table 3), suggesting that purifying selection was the predominant force in the evolution of *BjMc1* in land plants. Second, the LRTs to compare the data fit to models M1a vs M2a and M7 vs M8 were used to determine whether positive selection promoted the divergence of *Mc1* genes in land plants. No amino acid site was influenced by positive selection during the evolution of *Mc1* genes in land plants. These results indicated that the primary constraint for *Mc1* genes in land plants was purifying selection.

To assess whether the ancestors of *BjMc1* gene had undergone a pattern of molecular evolution in the evolutionary process of land plants, branch-model of *codeML* was performed. Six branches were selected from the phylogenetic tree (branch I, II, III, IV, V and VI) (Supplementary Fig. 4).We found that for branch I and II, the branch-model permitting a class of positively selected codons with *d*_*N*_: *d*_*S*_ > 1 had a significantly better fit to the data than the branch-model in which this class of codons were restricted to *d*_*N*_: *d*_*S*_ = 1. However, this was not the case for branch III, IV, V and VI (Supplementary Table 4). The results indicated that the evolution process of *BjMc1* gene might be influenced by positive selection in its ancestors of branch I and II. Because LRTs suggested that positive selection acted on the ancestral species of *BjMc1* gene, the method of Bayes empirical Bayes^34^, namely branch-site-model of *codeML*, was used to evaluate the positively selected sites and their posterior probabilities. A total of 32 codons were identified with a >50% posterior probability of *d*_*N*_: *d*_*S*_ > 1 along branch I. Of these codons, 2 amino acid sites had a >95% posterior probability of positive selection. Although 2 and 6 codons were identified with a >50% posterior probability of *d*_*N*_: *d*_*S*_ > 1 along branch III and branch IV respectively, no amino acid site had a posterior probability ≥95% of positive selection (Supplementary Table 5 and Supplementary Fig. 6). The two positive sites of branch I were located in the first cysteine domain and a key component of serine/threonine protein kinase domain respectively. When dicots and monocots were diverged from Moss, Lycophyte and Amborellaceae, the 139^th^ amino acid tryptophan (W) located in the first cysteine domain was mutated into phenylalanine (F), and the 899^th^ amino acid cysteine (C) located in a component of serine/threonine protein kinase domain was mutated into valine (V) except for in *Prunus mume*, *Eucalyptus grandis* and *Sesamum indicum* (Supplementary Fig. 7). In the long-term evolution process, both the two sites were preserved after mutation, indicating that the two amino acids played vital roles for the gene functions in dicot and monocot plants. The most reasonable explanation for these results was that in the evolutionary process of *BjMc1* gene, the ancestor had undergone adaptive evolution during a short period of time before the divergence of dicot and monocot plants from their ancestors on the earth, and then purifying selection was the dominant force for the evolution of these amino acid sites. These results also suggested the adaptability of these conserved *Mc1* genes to the land environment today.

### *BjMc1* gene located in B genome of *B*. *juncea* originated from *Brassica nigro* (*B*. *nigro*)

To understand the origination and evolution of *BjMc1* gene in detail, BLAST analysis was performed in Cruciferae using the protein sequence of *BjMc1* as a query. Aside from *BjMc1*, 13 homologues of *BjMc1* were identified from 8 species of Cruciferae, and one homologue was identified and isolated from *B*. *nigro* using primer BNI-1 designed based on *BjMc1* and *CLV1* sequences. Maximum-likelihood analysis using protein sequences classified all *BjMc1* homologues into two groups: *BjMc1* and its 7 homologues from *Brassicas* were classified into one group (Group I) and the other 8 homologues of *Cruciferae* were clustered in the other group (Group II) (Supplementary Fig. 8). Furthermore, *BjMc1* was located in the same clade with the homologue *BniMc1* of *B*. *nigra*, whereas *BjCLV1a* was located in the same clade with the homologue of *B*. *rapa*, indicating that *BjMc1* of *B*. *juncea* was from *B*. *nigra* (BB) and *BjCLV1a* of *B*. *juncea* was from *B*. *rapa* (AA). The *B*. *juncea* genome sequence information has been released recently^35^. The 24 AFLP markers obtained in our previous research and the three molecular markers obtained in this research were BLASTed against the *B*. *juncea* genome sequence database, and in total 16 molecular markers shared high homology with J17 (Supplementary Table 6). Therefore, it was suggested that the *BjMc1* locus derived from *B*. *nigro* was located in a genomic region of B genome (J17 chromosome) in *B*. *juncea*.

## Discussion

In recent years, the multilocular trait, which was thought to possess the potential to increase crop yield, was discovered in rapeseed. In the present study, we cloned the *BjMc1* gene controlling the carpel development in *B*. *juncea* through fine mapping and transgenic complementation. An insertion of a RTE1 disrupted the transcription of *BjMc1* (Fig. 2), leading to the result that the *Bjmc1* gene could translate a dominant negative protein and the trilocular silique grows on J163-4 plants. The analysis of the molecular evolutionary process revealed that *Mc1* genes had been conserved in modern land plants, and two amino acid sites of this gene located in the first cysteine domain and a key component of serine/threonine protein kinase domain had undergone positive selection during a short period of time before the divergence of dicot and monocot plants from their common ancestors on the earth. Our results also showed that the target gene cloned in this study was from J17 genome of *B*. *juncea*.

**Figure 6:**
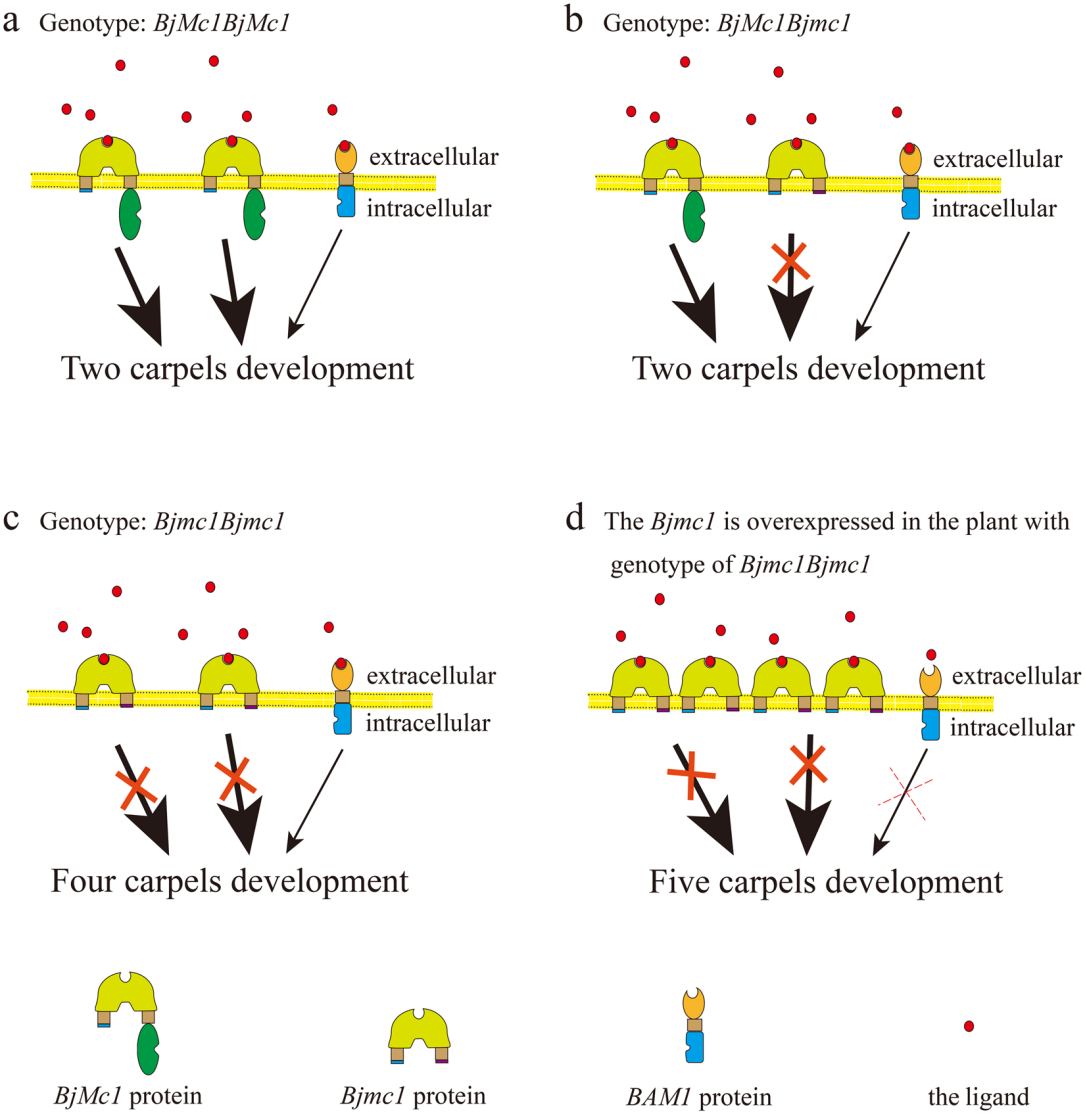
Model of dominant negative receptor action in *B*. *juncea*. A putative model for the role of *BjMc1* protein, *BARELY ANY MERISTEM 1* (*BAM1*) protein and the ligand protein in regulating the carpel development. Bold arrows indicate that the *BjMc1* protein plays a dominant role in regulating the development of carpel. (**a**) Scenarios for plants with genotype of *BjMc1BjMc1*. The ligand could bind to both the *BjMc1*and *BjBAM1* protein, but *BjMc1*protein was the dominant receptor kinase. (**b**) Scenarios for plants with genotype of *BjMc1Bjmc1*. The ligand could bind to the *BjMc1*, *Bjmc1*and *BjBAM1* protein, and the *BjMc1*protein was the dominant receptor kinase. (**c**) Scenarios for plants with genotype of *Bjmc1Bjmc1*. The ligand could bind to the *Bjmc1* and *BjBAM1* protein, but only the *BjBAM1* protein which does not play the dominant role in regulating pathway has the normal function. (**d**) Scenarios for plants with genotype of *Bjmc1Bjmc1* and *Bjmc1* overexpression. More ligands are possessed by the *Bjmc1*, and little ligand could bind to the *BjBAM1* protein.

Although mutilocular phenotype has attracted much research attention in the past few decades, the instability of multilocular trait has always confused researchers. All *clv1* mutants in *Arabidopsis* exhibited weak, intermediate or strong multilocular phenotypes^27^, and previous studies had shown that most *Brassicas* plants with multiloculus had variable valve numbers in the siliques of the same plant^10, 11^. However, in this study, J163-4 planted in both central and northwestern China exhibited four valves stably in trilocular siliques. We transformed the *Bjmc1* gene into the *clv1-1* allele in *Arabidopsis* for over-expression, and proved that the truncated *Bjmc1* protein had a potential dominant negative effect, which could have played a key role in maintaining the valve number per silique to be four. Moreover, the over-expression of *Bjmc1* gene in trilocular plants of NILs is in progress to further prove the dominant negative effect of this mutant gene. The dominant negative model was proposed by previous research: if a protein was monomeric, the activity of a protein was limited by the availability of substrate, then a variant capable of binding substrate without carrying out a subsequent catalytic step could be inhibitory^36^. In *Arabidopsis*, *clv1-20* showed a weak multilocular phenotype^17^, while single *barely any meristem 1* (*bam1*) mutant did not display multilocular phenotype^37^. Double mutants between *bam1-3* and *clv1-20* exhibited a strongly synergistic defect of extra carpels similar to those of *clv1-4* which showed a strong multilocular phenotype and was thought to be a dominant negative mutation, indicating that *clv1-4* dominant negative mutation caused inhibitory interaction between *clv1* and *bam1* protein^17^. In addition, the *BjMc1* was a Leu-rich repeat receptor serine/threonine kinase, which could be bound by the ligand to the extracellular domain. In our study, the truncated *Bjmc1* protein contained the putative extracellular domain but without the putative intracellular domain (Supplementary Fig. 3c). Hence, it indicated that although the ligand could normally bind to *Bjmc1* protein, the truncated protein had lost the function of serine/threonine domain, and acted as a monomeric variant in the molecular mechanism. Although *BjBAM1* interacted synergistically with *BjMc1*, the *BjMc1* protein was the dominant receptor kinase to regulate the carpel development, because it was *clv1* but not *bam1* that showed multilocular phenotype according to researches in *Arabidopsis*. The dominant negative molecular model of *Bjmc1* protein was shown in Fig. 6.

In this research, although the *pBjCLV1b*:*BjCLV1b-*transgenic lines did not display a complete bilocular phenotype, the exogenous gene introduced into J163-4 had worked. Because the J163-4 plants always exhibited trilocular silique but the transgenetic plants showed chimeric phenotype and the exogenous *BjMc1* gene expressed (Fig. 3b). There were several explanations for this phenotype. First, the potential dominant negative effect of *Bjmc1* gene might make the exogenous *BjMc1* gene not working completely. Unless the expression level of the exogenous gene was much higher, the similar or lower expression level to that of endogenous *BjMc1* gene could not restore the triloculus; Second, the transgenic receptor material was the trilocular parental line J163-4 rather than the trilocular plants in NILs. Although the genotypes of the trilocular parental line and trilocular plants in NILs were the same, their genetic backgrounds were quite different. In our present research, we are trying to introduce the *pBjCLV1b*:*BjCLV1b* construct into the trilocular plants in NILs. Third, *BjMc1* gene took part in the development of SAM. In Soybean, *Dt2* regulated stem growth, which produced semideterminate plants terminal racemes. When *35S*:*CDS-Dt2* construct (*Dt2* with 35S promoter) and *Pro-Dt2*:*CDS- Dt2* construct (*Dt2* with putative endogenous promoter) were introduced into Thorne which displayed the indeterminate stem growth respectively, only *35S*:*CDS-Dt2*-transgenic lines displayed semideterminate stem growth^38^, and this situation was similar to our research. So, we speculated that the genes involving in the SAM development were controlled by some regulatory mechanism, which could make similar phenotypes in this kind of genes. In our study, although *pBjCLV1b*:*BjCLV1b-*transgenic lines did not show a complete bilocular phenotype, *35S*::*BjCLV1b-*transgenic lines showed a complete bilocular phenotype in *B*. *juncea*. Similarly, the transgenic lines of *Arabidopsis*, *clv1-1*, also showed similar phenotype with J163-4, and *Mc1* genes in different species were conservative. Overall, *BjCLV1b* was the target gene of *BjMc1*, and the investigation will be carried out to explain the chimeric phenotype of transgenic lines in our following research.

Blast searches revealed that *Mc1* genes were widespread in land plants, and analysis of gene structure revealed that *Mc1* genes in land plants were conserved, especially at serine/threonine domain. There were two *Mc1* copies, *BjCLV1a* and *BjCLV1b*, in *B*. *juncea*. The evolution analysis revealed that *BjCLV1a* located in A chromosome was derived from *B*. *rapa* while *BjCLV1b* located in B chromosome was derived from *B*. *nigro*, indicating that the evolution of *BjMc1* and its homologues in polyploidization followed a regular U-triangle^39^ evolutionary pattern. In the analysis of evolution process, we found that the dominant driving force for *Mc1* genes in land plants was purifying selection, which contributed to functional stabilization. The mutation of *BjMc1* homologous genes led to a larger fruit size in tomato, increased kernel row number in maize^21, 22^ and seed number per silique in rapeseed. The mutation of *Mc1* genes always could increase the yields of crops or vegetables. These findings indicated that modulation of the pathways that control fundamental stem cell proliferation had the potential to enhance crop yield. Although *Mc1* genes were important to plant growth, their mutations did not exhibit negative impacts on plants, suggesting that they could be an excellent gene resource for breeding of high-yield crops. Therefore, homologous genes of *BjMc1* could be edited by genome-editing technology or silenced by RNA interference technology in crops. Moreover, because *Bjmc1* was dominant negative and recessive, it could be overexpressed in other crops whose *BjMc1* homologous genes had been silenced to obtain the cultivars with consistent high yields. In future work, we will continue to focus on the application of the dominant negative effect to crop breeding for higher yields.

## Methods

### Plant materials

Homozygous near-isogenic lines (NILs, BC_4_F_1_) were constructed using a trilocular line J163-4 and a bilocular line J248^28^. Based on the NILs, a BC_5_F_1_ population (3,700 individuals) and a BC_6_F_1_ population (5,600 individuals) were further developed in 2012 to 2014 to fine map *Bjmc1* gene. All of the identified individuals with recombination events at *Bjmc1* locus were further determined by progeny testing in 2013 to 2015.

*Arabidopsis thaliana* (*Col-0* ecotype) was used to reveal the expression pattern of *BjMc1* gene by b-glucuronidase (GUS) staining and subcellular localization. *Arabidopsis clv1-1* was purchased from ABRC (http://abrc.osu.edu/), and its mutation site was shown in Fig. 3d.

### Availability of data and material

All data generated or analyzed during this study are included in this published article and its additional files.

### BAC screening and sequencing

Positive clones from the purple-leaf mustard (*Brassica juncea*) bacterial artificial chromosome (BAC) library were identified through a two-stage polymerase chain reaction (PCR) screening method with C1-1 primers designed from the sequence of *Bra015812* which was located near the homologous sequence of molecular marker SC13 in A07 chromosome of *B*. *rapa*. The phenotypes were similar between J163-4 and *clv1* mutant in *Arabidopsis* (Crooijmans *et al*., 2000). BAC DNA was sequenced as previously described by Yi *et al*.^40^.

### Genetic mapping and positional cloning

Bulked segregant analysis^41^ was conducted to screen the molecular markers linked to *BjMc1* locus. Simple sequence repeats (SSR) was designed according to the reference sequence retrieved from the positive BAC clone. The parents and bulks were subjected to SSR marker analysis. PCR was performed according to Xu *et al*.^28^. PCR products were then separated on 1% polyacrylamide denaturing sequencing gel and shown by silver nitrate staining. Linkage analysis was performed using Joinmap4^42^. All genetic distances were expressed in centiMorgan (cM) using the Kosambi function^43^.

### cDNA preparation and 5′- and 3′- rapid-amplification of cDNA ends (RACE)

Total RNA was extracted from various plant tissues using an RNA extraction kit (RNeasy Plant Mini Kit; QIAGEN). The first-strand cDNA was synthesized using 2 mg of RNA and 200 units of M-MLV reverse transcriptase (Promega Kit) in a volume of 25 ml. The 5′- and 3′- RACE reactions were performed using the SMARTer RACE Amplification Kit (Clontech) according to manufacturer’s instructions.

### Constructs and transformation

The genomic fragments of the candidate gene were amplified from the biolocular plants from the NILs using high-fidelity PCR. A 6,613 bp genomic DNA fragment spanning from 2,692 bp upstream the translation start codon to 767 bp downstream the termination codon of *BjMc1* using primer E48-2 (Supplementary Table 1) was amplified. The correct fragment confirmed by sequencing was then cloned into Pst I - kpn I site of pCAMBIA2300 vector to construct plasmid *pBjMc1*:*BjMc1*. To prepare *p35S*::*BjMc1* construction, we cloned a 3455 bp genomic DNA fragment spanning from start codon to 301 bp downstream the termination codon of *BjMc1* using primer E46-1 (Supplementary Table 1) into Pst I - kpn I site of pCAMBIA2300, and double 35S promoter was cloned into Hind III - Pst I site of pCAMBIA2300. To prepare the Pro_BjMc1_-GUS construct, the *BjMc1* promoter region (from 1 to 2499 bp) using primer E58-1 (Supplementary Table 1) was amplified. A cassette containing GUS coding region followed by nopaline synthase polyadenylation signal from pBI101 vector was subcloned into the binary vector pCAMBIA 2300 with restriction enzymes Hind III and EcoR I to construct promoter-GUS fusions. The amplified fragments were subcloned into the modified binary vector pCAMBIA 2300 to yield the 2499 bp *BjMc1* promoter-GUS construct. To prepare *p35S*::*Bjmc1* construct, a 3036 bp genomic DNA fragment spanning from the start codon to a 687 bp downstream DNA fragment using primer HY-4 (Supplementary Table 1) was amplified. The correct fragment confirmed by sequencing was cloned into Xba I - Sac I site of pMDC83 vector to construct plasmid *p35S*::*Bjmc1*. These constructs were introduced into the host cells of *Agrobacterium tumefaciens* GV3101. The *pBjMc1*:*BjMc1* and *p35S*::*BjMc1* were transformed into the trilocular line J163-4, whereas Pro_BjMc1_-GUS construct was introduced into wild-type *Arabidopsis* (*Col-0* ecotype) and *p35S*::*Bjmc1* was transformed into *clv1-1* by floral dipping^44^. The T2 transgenic plants of Pro_BjMc1_-GUS were grown for GUS staining.

### Expression analysis

Total RNA was extracted using Trizol reagent (Invitrogen). The tissues of the early inflorescence of trilocular plants and SAM, early inflorescence, 1–4 mm ovary, 4–7 mm ovary, 7–10 mm ovary, silique peel, leaves, stem and root of bilocular plants in BC_5_F_1_ generation were selected, and 3 mg of total RNA from each tissue was treated with RNase-free DNase I to remove contaminated DNA respectively, then reverse transcribed into the first-strand complementary DNA (cDNA) with M-MLV reverse transcriptase (Fermentas, Vilnius, Lithuania) using oligo d(T)_25_ primer. The reverse-transcribed products from various tissues were used as templates for qPCR assay using the Bj3 primer (Supplementary Table 1) which could particularly amplify *BjMc1* copy to examine the expression of *BjMc1* gene. The qPCR was conducted according to Li *et al*.^4^. The measurements were obtained using the relative quantification method^45^. *ACTIN2* gene was used as the internal control for *B*. *juncea* ^46^. All expression level data obtained by qPCR were based on three biological samples and three replicates for each sample.

The cDNA of *BjMc1* and *Bjmc1* were detected from the reverse-transcribed products from early inflorescence of trilocular and bilocular plants in BC_5_F_1_ generation using the semi-quantitative RT-PCR by C1-2 and M1-1. The *ACTIN2* gene was used as the control. Semi-quantitative RT-PCR was performed as following: 94 °C for 3 min; twenty-five cycles of 94 °C for 30 s, 55 °C for 30 s and 72 °C for 45 s; and a final 10-min elongation step.

### Histochemical GUS staining

Twelve independent T_2_ transgenic lines of Pro_BjMc1_-GUS were subjected to histochemical GUS assays. Seedlings of 10 d and various organs of the transgenic plants were incubated at 37 °C overnight in 5-bromo-4-chloro-3-indolyl-b-glucuronic acid solution and then cleaned in 75% (v/v) ethanol. The treated tissues were observed on an Olympus IX-70 Microscope equipped with Nomarski Optics^4^.

### Subcellular localization of *BjMc1*

The *BjMc1* coding sequence without the termination codon (TAA) was amplified from the biolocular plants in BC_5_F_1_ generation by PCR using YXB4 primer (Supplementary Table 1). The amplified cDNA fragments were inserted downstream of the double 35S promoter through Xba I - BamH I site in frame with GFP in pMDC83 vector. This plasmid was transformed into *Arabidopsis*. The roots of transgenic plants in T2 generation were incubated with 10 μM FM4-64 for at least 5 min before observation. The emission light was dispersed and recorded at 500–540 nm for GFP. Confocal images were taken with a Nikon Eclipse80i fluorescence microscope equipped a water-immersed ×40 lens with an excitation wavelength of 488 nm and the following detection wavelengths: 500–540 nm for GFP and at >650 nm for FM4-64 (Nikon, Japan). All fluorescence experiments were independently repeated at least three times.

### Identification of *Mc1* genes from Cruciferae and other land plant species

To identify *Mc1* homologous genes in Cruciferae, BLAST analysis using protein sequence of *BjMc1* as a query was performed in Cruciferae (http://brassicadb.org/brad/, http://www.arabidopsis.org/, http://122.205.95.67/blast/blast.php).

To identify *Mc1* homologous genes in the land plants, the coding sequence (CDS) of *BjMc1* was used as query to search the National Center for Biotechnology Information (http://www.ncbi.nlm.nih.gov/), Ensembl Plants (http://plants.ensembl.org/index.html) and the Arabidopsis Information Resource database (http://www.arabidopsis.org/). The most highly similar sequence was selected from each species. The deduced nucleotide and protein sequences of land plant *Mc1* genes identified in this analysis were used for further analysis.

### Phylogenetic analyses and detection of positive selection

Using the MEGA5^47^, Cruciferae *Mc1* amino acid sequences were aligned by ClastalW and land plant *Mc1* CDSs were aligned by ClastalW condons, and finally the FASTA formats were exported. The maximum-likelihood approach was used for the phylogenetic analysis of Cruciferae and all the land plants, respectively.

To test the selective pressure of *Mc1* genes during the long period of evolution in land plants, the value of *d*_*N*_: *d*_*S*_ ratio (or *ω*) for *Mc1* genes was calculated with the program *codeML* from Phylogenetic Analysis by Maximum Likelihood (PAML) v4.4^32^. In this study, three likelihood ratio tests (LRTs), M0, M1a vs M2a and M7 vs M8, were used to examine the selective pressure. M0 was used to calculate the average *ω* value of all codon sites, and the other two LRTs were used to detect the role of positive selection. For one LRT, the differences of log likelihood of the two models were compared using chi-squared (χ^2^) statistics. In our analyses, the degree of freedom was 1 for M1a/M2a and M7/M8 tests^33, 48^.

The improved branch model and branch site model^49^ were also used to detect the role of positive selection on the land plant *Mc1* genes. In these two models, six branches were selected from the phylogenetic tree (branch I, II, III, IV, V and VI); and when any one of the branches served as the foreground branch, the remaining branches were background branches (Supplementary Fig. 4). For the analysis of branch site model, we compared the null hypothesis (*ω* fixed to 1) with the alternative hypothesis (free *ω*) to test whether positive selection affected the evolution of land plant *Mc1* genes. The Bayes empirical Bayes procedure in *codeML* ^34^ was used to calculate the posterior probability that each site in the foreground branch was subjected to positive selection.

## Electronic supplementary material

Revised Supplementary Figures and Tables

Supplementary Datum

## References

[1] Barton MK (2010). Twenty years on: the inner workings of the shoot apical meristem, a developmental dynamo. Dev. Biol..

[2] Lv ZW (2012). Primary study on anatomic and genetic analyses of multi-loculus in *Brassica juncea*. Chin. J. Oil Crop Sci..

[3] He YT (2003). Anatomic and genetic studies on multicapsular character in *Brassica campestris* L. Chin. J. Oil Crop Sci..

[4] Li SP (2015). *BnaC9*. *SMG7b* functions as a positive regulator of the number of seeds per silique in *Brassica napus* by regulating the formation of functional female gametophytes. Plant Physiol..

[5] Liu J (2015). Natural variation in *ARF18* gene simultaneously affects seed weight and silique length in polyploid rapeseed. Proc. Natl. Acad. Sci. USA.

[6] Zhang LW, Li SP, Chen L, Yang GS (2012). Identification and mapping of a major dominant quantitative trait locus controlling seeds per silique as a single Mendelian factor in *Brassica napus* L. Theor. Appl. Genet..

[7] Zhao HC (2003). Performances in main characteristic of multilocular *B*. *juncea*. Acta. Agric. Boreali-Occident Sinica..

[8] Choudhary BR, Solanki ZS (2007). Inheritance of silique locule number and seed coat colour in *Brassica juncea*. Plant Breed..

[9] Yadava SK (2014). Tetralocular ovary and high silique width in yellow sarson lines of *Brassica rapa* (subspecies trilocularis) are due to a mutation in *Bra034340* gene, a homologue of *CLAVATA3* in *Arabidopsis*. Theor. Appl. Genet.

[10] Xiao L (2013). Genetic and physical fine mapping of a multilocular gene *Bjln1* in *Brassica juncea* to a 208-kb region. Mol. Breed.

[11] Fan CC (2014). A novel single-nucleotide mutation in a *CLAVATA3* gene homologue controls a multilocular silique trait in *Brassica rapa* L. Mol. Plant.

[12] Clark SE, Running MP, Meyerowitz EM (1993). *CLAVATA1*, a regulator of meristem and flower development in *Arabidopsis*. Development.

[13] Muller R, Bleckmann A, Simon R (2008). The receptor kinase *CORYNE* of *Arabidopsis* transmits the stem cell-limiting signal *CLAVATA3* independently of *CLAVATA1*. Plant Cell.

[14] Forstheofel NR, Wu Y, Schulz B, Bennett MJ, Feldmann K (1992). T-DNA insertion mutagenesis in *Arabidopsis*: prospects and perspectives. Aust. J. Plant Physiol..

[15] Kayes JM, Clark SE (1998). *CLAVATA2*, a regulator of meristem and organ development in *Arabidopsis*. Development.

[16] Xu C (2015). A cascade of arabinosyltransferases controls shoot meristem size in tomato. Nat. Genet..

[17] Durbak AD, Tax FE (2011). *CLAVATA* signaling pathway receptors of *Arabidopsis* regulate cell proliferation in fruit organ formation as well as in meristems. Genetics.

[18] Cheng ZP, Yang ZN, Zhang S (2013). *CLV1* interacts with *UFO* in modulation of gynoecium development in *Arabidopsis thaliana*. J. Plant Biol..

[19] Prunet N (2015). *SQUINT* promotes stem cell homeostasis and floral meristem termination in *Arabidopsis* through *APETALA2* and *CLAVATA* signaling. J. Exp. Bot..

[20] Mandel T (2014). The *ERECTA* receptor kinase regulates *Arabidopsis* shoot apical meristem size, phyllotaxy and floral meristem identity. Development.

[21] Bommert P (2004). *thick tassel dwarf 1* encodes a putative maize ortholog of the *Arabidopsis CLAVATA1* leucine-rich repeat receptor-like kinase. Development.

[22] Bommert P, Nagasawa NS, Jackson D (2013). Quantitative variation in maize kernel row number is controlled by the *FASCIATED EAR2* locus. Nat. Genet..

[23] Suzaki T (2004). The gene *FLORAL ORGAN NUMBER1* regulates floral meristem size in rice and encodes a leucine-rich repeat receptor kinase orthologous to *Arabidopsis CLAVATA1*. Development.

[24] Medford JI, Behringer FJ, Callos JD, Feldmann KA (1992). Normal and abnormal development in the *Arabidopsis* vegetative shoot apex. Plant Cell.

[25] Pogany JA (1998). Identifying novel regulators of shoot meristem development. J. Plant Res..

[26] Clark SE, Williams RW, Meyerowitz EM (1997). The *CLAVATA1* gene encodes a putative receptor kinase that controls shoot and floral meristem size in *Arabidopsis*. Cell.

[27] Diévart A (2003). *CLAVATA1* dominant-negative alleles reveal functional overlap between multiple receptor kinases that regulate meristem and organ development. Plant Cell.

[28] Xu P (2014). Identification of molecular markers linked to trilocular gene (*mc1*) in *Brassica juncea* L. Mol. Breed.

[29] Wang G (2016). Fine mapping of polycyetic gene (*Bjmc2*) in *Brassica juncea* L. Acta. Agronomica Sinica..

[30] Shpak ED, Lakeman MB, Torii KU (2003). Dominant-Negative Receptor Uncovers Redundancy in the *Arabidopsis ERECTA* Leucine-Rich Repeat Receptor–Like Kinase Signaling Pathway That Regulates Organ Shape. Plant Cell.

[31] Stahl Y (2013). Moderation of *Arabidopsis* root stemness by *CLAVATA1* and *ARABIDOPSIS CRINKLY4* receptor kinase complexes. Curr. Biol..

[32] Yang Z (2007). PAML 4: phylogenetic analysis by maximum likelihood. Mol Biol Evol..

[33] Nielsen R, Yang Z (1998). Likelihood models for detecting positively selected amino acid sites and applications to the HIV-1 envelope gene. Genetics.

[34] Yang Z, Wong WS, Nielsen R (2005). Bayes empirical bayes inference of amino acid sites under positive selection. Mol. Biol. Evol..

[35] Yang JH (2016). The genome sequence of allopolyploid *Brassica juncea* and analysis of differential homoeolog gene expression influencing selection. Nat. Genet..

[36] Herskowitz I (1987). Functional inactivation of genes by dominant negative mutation. Nature.

[37] DeYoung BJ (2006). The *CLAVATA1*-related *BAM1*, *BAM2*, and *BAM3* receptor kinase-like proteins are required for meristem function in *Arabidopsis*. Plant J.

[38] Ping JQ (2014). *Dt2* is a gain-of-function MADS-domain factor gene that specifies semideterminacy in soybean. Plant Cell.

[39] Nagaharu U (1935). Genome analysis in *Brassica* with special reference to then experimental formation of *B*. *napus* and peculiar mode of fertilization. Jpn. J. Bot.

[40] Yi B (2010). Two duplicate CYP704B1 homologous genes *BnMs1* and *BnMs2* are required for pollen exine formation and tapetal development in *Brassica napus*. Plant J.

[41] Michelmore RW, Paran I, Kesseli R (1991). Identification of markers linked to disease-resistance genes by bulked segregant analysis: a rapid method to detect markers in specific genomic regions by using segregating populations. Proc. Natl. Acad. Sci. USA.

[42] van Ooijen, J. Joinmap®: software for the calculation of genetic linkage maps in experimental populations, version 4. Wageningen, the Netherlands: Kyazma BV (2006).

[43] Kosambi D (1943). The estimation of map distances from recombination values. Ann. Hum. Genet.

[44] Clough SJ, Bent AF (1998). Floral dip: a simplified method for Agrobacterium-mediated transformation of *Arabidopsis thaliana*. Plant J.

[45] Livak KJ, Schmittgen TD (2001). Analysis of relative gene expression data using real-time quantitative PCR and the 2^−ΔΔCT^ Method. Methods.

[46] Ame´lie AK, Eve S, Stephen JP, Smita K, Peter JE (2013). Suppression of the *SUGAR-DEPENDENT1* triacylglycerollipase family during seed development enhances oil yield in oilseed rape (*Brassica napus L*.). Plant Biotechnol. J..

[47] Tamura K (2011). MEGA5: molecular evolutionary genetics analysis using maximum likelihood, evolutionary distance, and maximum parsimony methods. Mol. Biol. Evol..

[48] Wong WS, Yang Z, Goldman N, Nielsen R (2004). Accuracy and power of statistical methods for detecting adaptive evolution in protein coding sequences and for identifying positively selected sites. Genetics.

[49] Zhang J, Nielsen R, Yang Z (2005). Evaluation of an improved branch-site likelihood method for detecting positive selection at the molecular level. Mol. Biol. Evol..

