# Supplementary material for: Trilocular phenotype in Brassica juncea L. resulted from interruption of CLAVATA1 gene homologue (BjMc1) transcription
Source: Sci Rep. 2017 Jun 14;7:3498. doi: 10.1038/s41598-017-03755-0 (PMC5471281; doi:10.1038/s41598-017-03755-0)
Supplement: Supplementary file 1 — Revised Supplementary Figures and Tables [file 41598_2017_3755_MOESM1_ESM.doc]

**Trilocular phenotype in *Brassica juncea* L. resulted from interruption of *CLAVATA1* gene homologue (*BjMc1*) transcription**

Ping Xu^1^; Shiqin Cao^1^; Kaining Hu^1^; Xiaohua Wang^1^; Wei Huang^1^; Gang Wang^1^；Zewen Lv^1^; Zhongsong Liu^2^; Jing Wen^1^; Bin Yi^1^; Chaozhi Ma^1^; Jinxing Tu^1^; Tingdong Fu^1^; Jinxiong Shen^1 ⃰^

**Supplementary Figures**


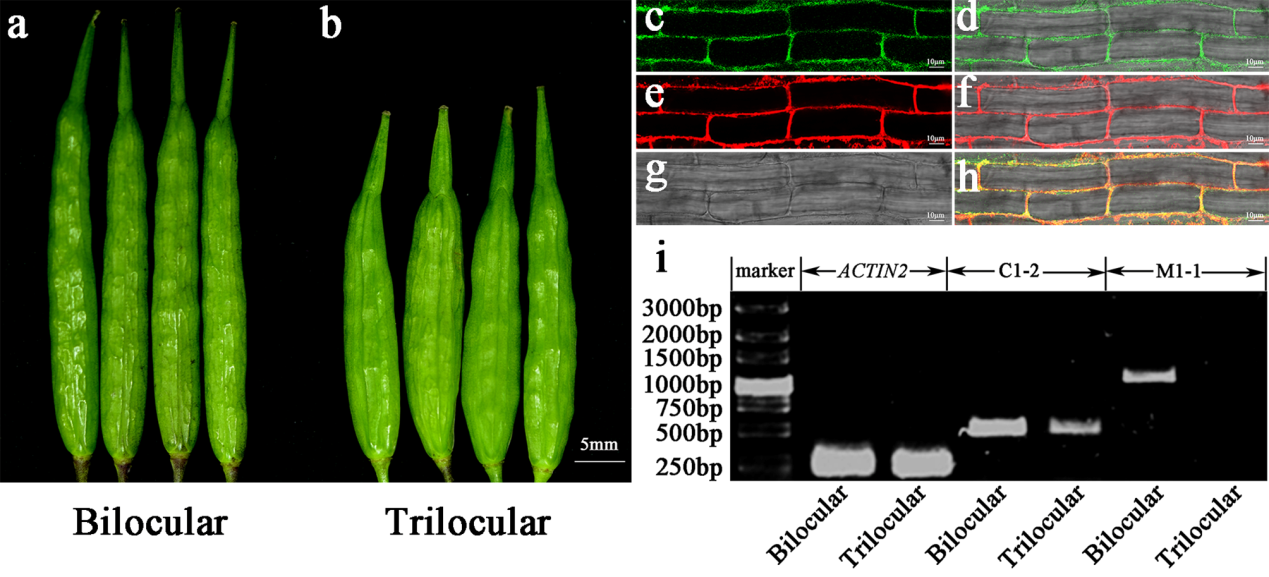


**Supplemental Figure 1.** **The silique phenotypes in NILs of *BjMc1*, subcellular localization of *BjMc1* in *Arabidopsis* root cell and the expression of *BjMc1* and *Bjmc1*.** (**a**) The bilocular silique. (**b**) The trilocular silique. (**c**) localization of *BjMc1*-GFP in dark-field. (**d**) Localization of *BjMc1*-GFP in bright-field. (**e**) The plasma membrane displayed by FM4-64 staining in dark-field. (**f**) The plasma membrane displayed by FM4-64 staining in bright-field. (**g**) Bright-field image of *Arabidopsis* root cell. (**h**) The merged image of GFP and FM4-64 in bright-field. (**i**) The expression level of *BjMc1* and *Bjmc1*detected by C1-2 and M1-1 using semi-quantitative RT-PCR.


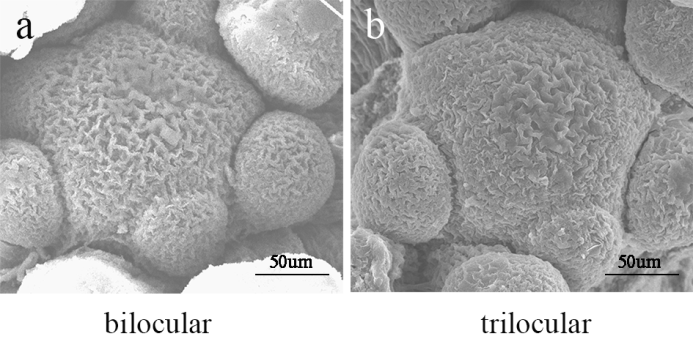


**Supplemental Figure 2. Scanning electron microscope detection of the inflorescence meristems and floral meristems of bilocular and trilocular plants in BC_6_F_1_ generation.** (**a**) the bilocular inflorescence meristems and floral bud meristems. (**b**) the trilocular inflorescence meristems and floral bud meristems.

**
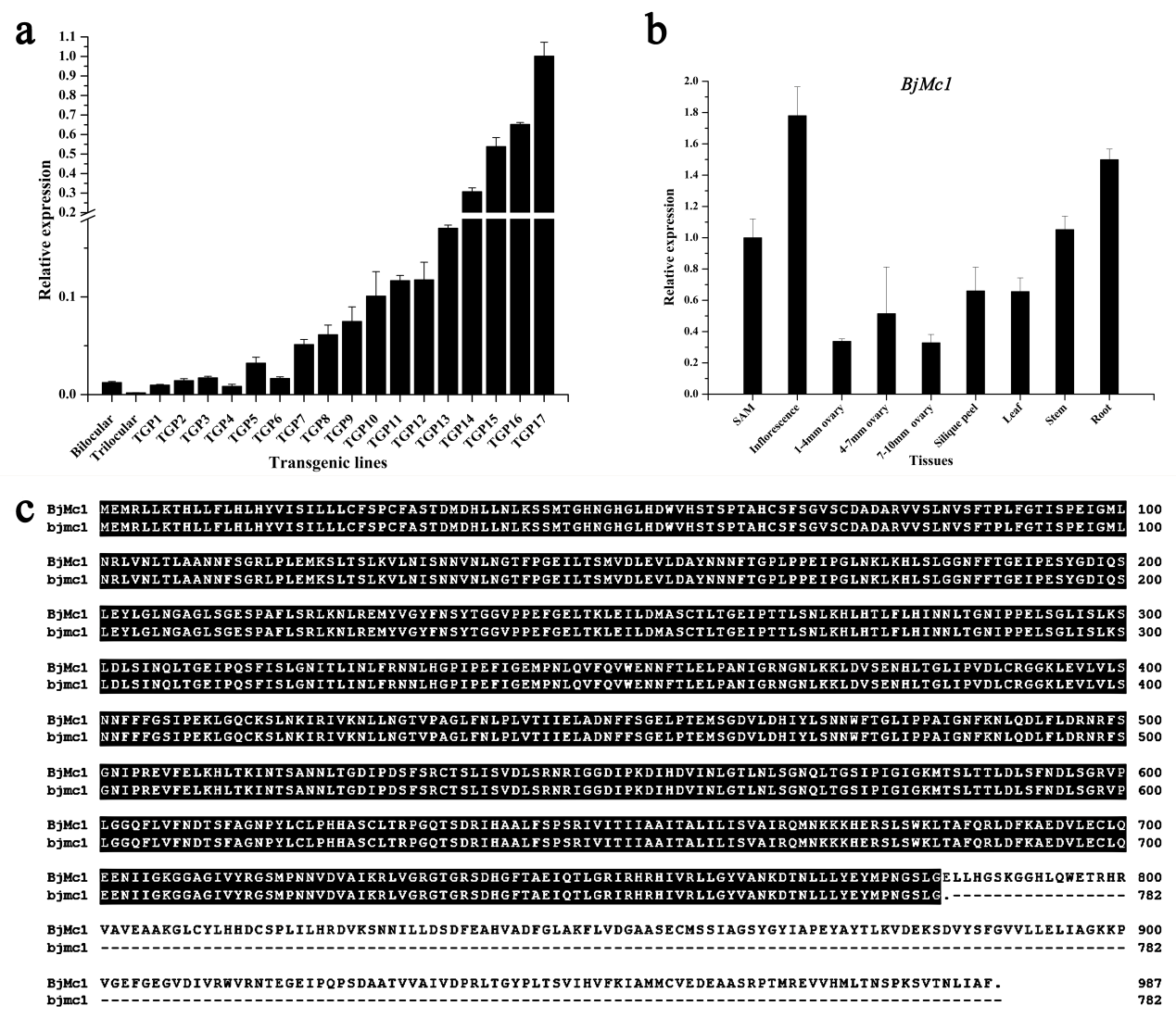
**

**Supplemental Figure 3.** (**a**) Expression pattern of *BjMc1* in early inflorescence was analyzed by q-PCR. *ACTIN2* was used as a control. TGP1-4 showed *BjMc1* gene expression in *pBjCLV1b:BjCLV1b*-transgenetic T_0_ lines, and TGP5-15 showed *BjMc1* gene expression in *p35S::BjCLV1b-*transgenetic T_0_ lines. (**b**) The q-PCR analysis of *BjMc1* in different tissues of bilocular plants in BC_5_F_1_ generation. *ACTIN2* was used as a control. (**c**) Protein *CLUSTALW* alignment of *BjMc1* and *Bjmc1*.

**
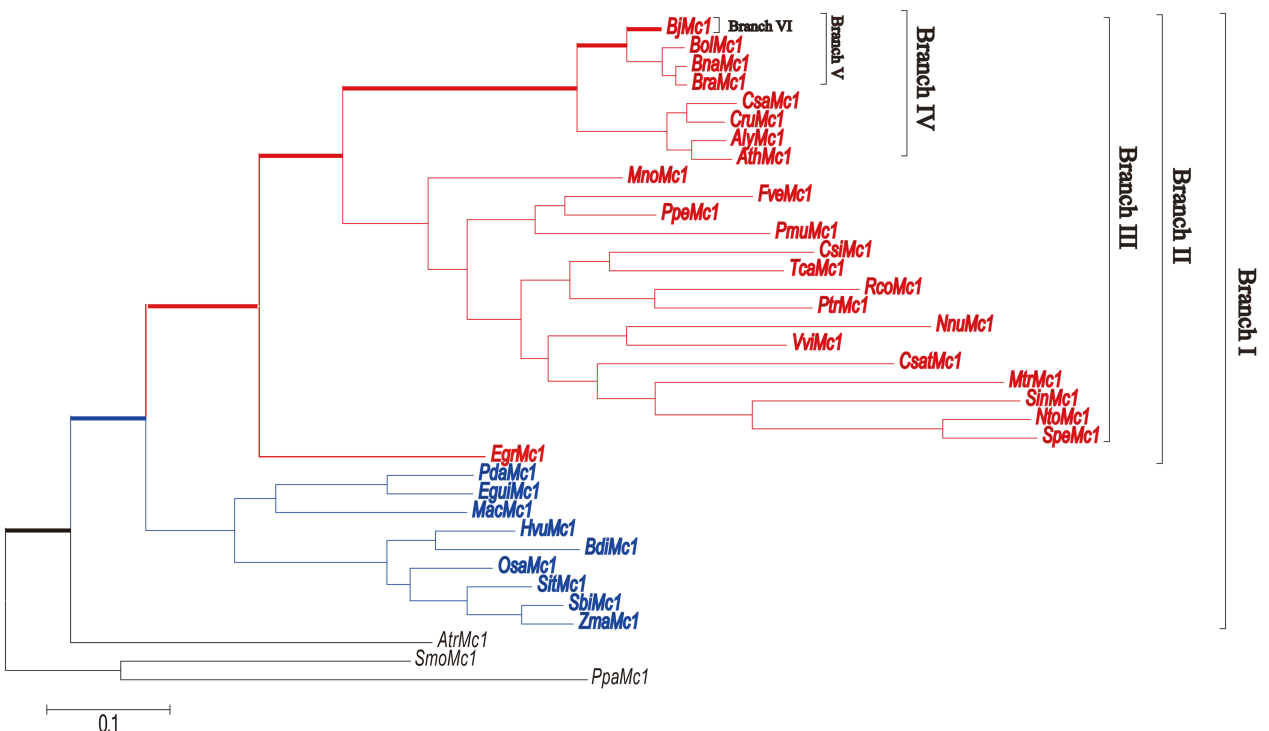
**

**Supplemental Figure 4. Phylogenetic tree of the land plant *BjMc1* homologous CDSs.** The maximum likelihood phylogenetic tree was constructed using MEGA5. The numbers above the branches represent the level of confidence (% bootstrap support 1,000 replicates). The lengths of the branches refer to the amino variation rates. The evolution track of *BjMc1* is shown by bold lines.

**
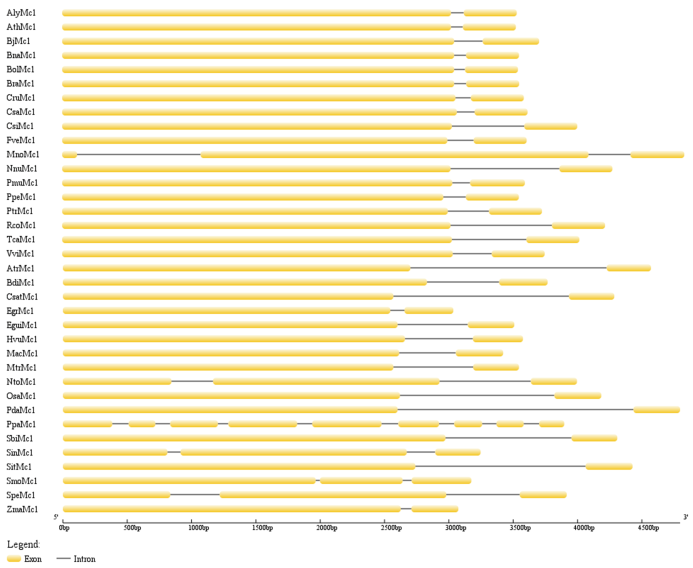
**

**Supplemental Figure 5.** **Exon-intron structures of plant *BjMc1* homologous genes in land plants.**

**
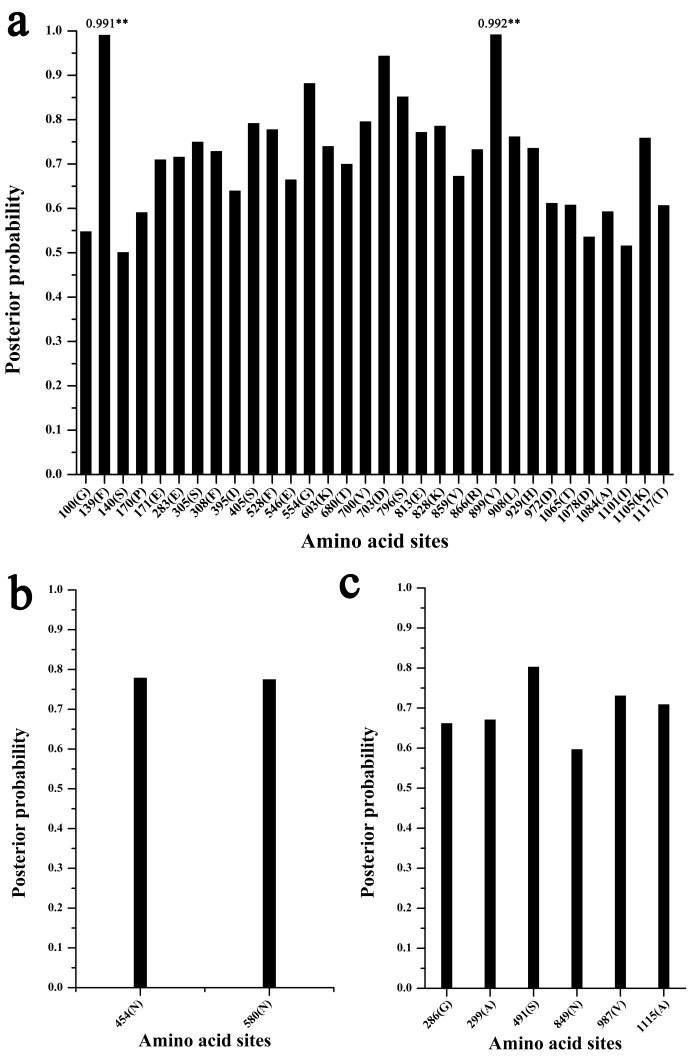
**

**Supplemental Figure 6.** **Codons detected by branch-site-model of *codeML* with a > 50% posterior probability of *d_N_* : *d_S_* > 1.** (**a**) 32 codons were identified by branch I as foreground branch. 2 amino acid sites have a 95% posterior probability of positive selection. (**b**) 2 codons were identified by branch III as foreground branch. (**c**) 6 codons were identified by branch IV as foreground branch.

**
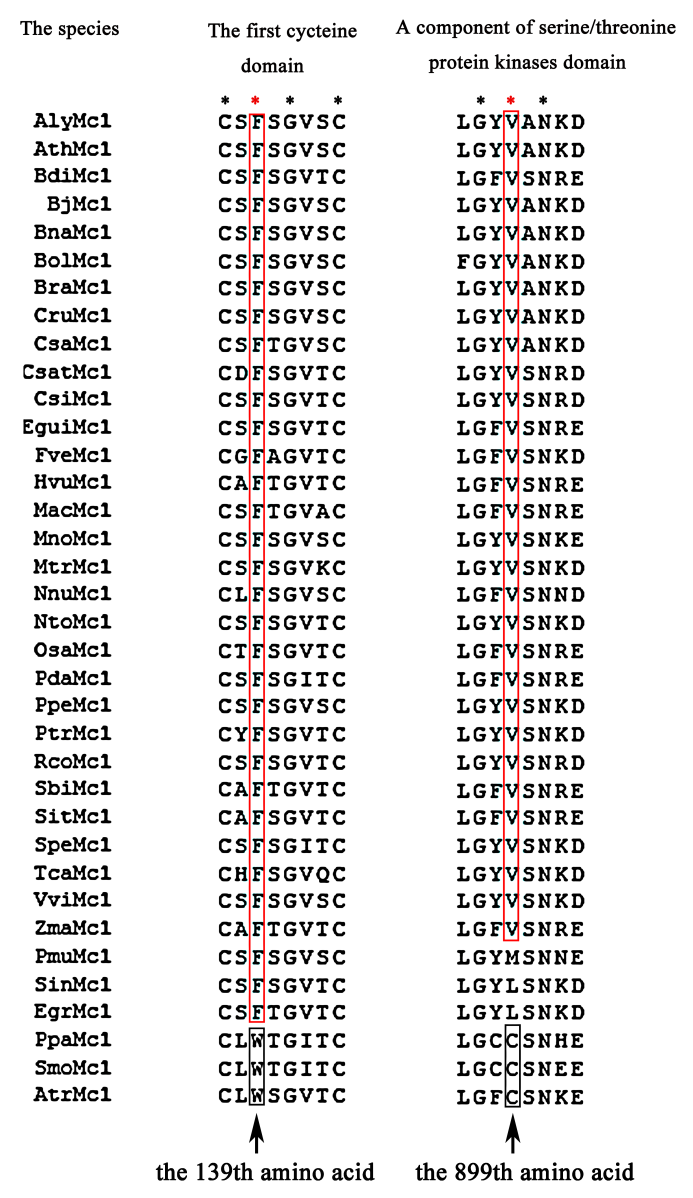
**

**Supplemental Figure 7.** **Two amino acid sites having > 95% posterior probability of positive selection.** The positively selected amino acids are indicated by red boxes, and the corresponding amino acids in the ancestor are indicated by black boxes. Black asterisks show the conserved amino acids in all plants. The red asterisks show the 139^th^ and 899^th^ amino acid, respectively.

**
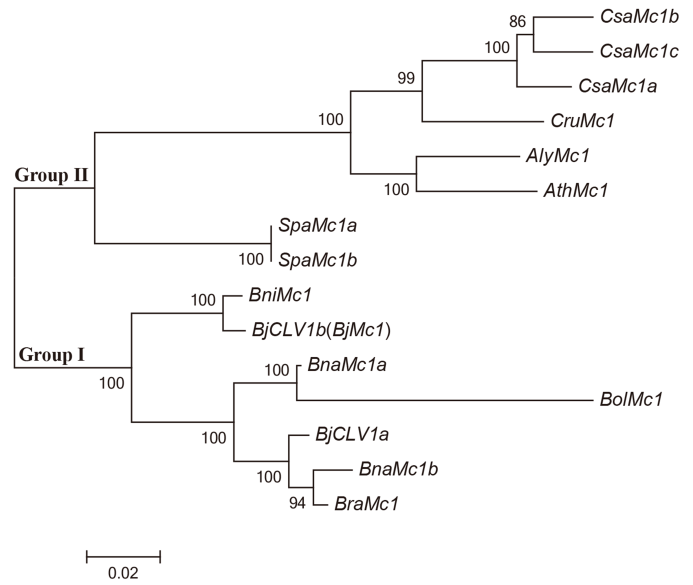
**

**Supplemental Figure 8.** **Phylogenetic tree of the Cruciferae *BjMc1* homologous proteins.** The maximum-likelihood phylogenetic tree was constructed using MEGA5. The numbers above the branches represent the level of confidence (% bootstrap support 1,000 replicates). The lengths of the branches refer to the nucleotide variation rates.

**Supplementary Tables**

**Supplemental Table 1.** Summary of primers used in this study

| Primer name | Sequence 5’-3’ | purpose |
| --- | --- | --- |
| SR52L  SR52R  SC151L  SC151R  SC40L  SC40R  C1-1L  C1-1R  CV4L  CV4R  M1-1L  M1-1R  BNI-1L  BNI-1R  BJ3L  BJ3R  DL2L  DL2R  E48-2L  E48-2R  E46-1L  E46-1R  E58-1L  E58-1R  HY-4L  HY-4R  YXB4L  YXB4R  C1-2L  C1-2R  AP1L  AP1R  AP2L  AP2R  AP3L  AP3R  PIL  PIR  AGL  AGR  CLV3L  CLV3R  CLV2L  CLV2R  CRNL  CRNR  WUSL  WUSR  PLL1L  PLL1R  POLL  POLR  KAPPL  KAPPR  BAM1L  BAM1R  BAM2L  BAM2R  BAM3L  BAM3R | GGGGACCTGTTATGAAGCTCTA  GCAAGTGTGCCTGATGTGTTAT  TAACCTTGAGACCAACACTCCG  CCGATTCGTGTCCAGATCTATG  CTCCAACAAGACCACTCCAAAG  AATGACTGGTCACAACGGACAC  GGAGGAGCGATCACGGATTCAC  AGCAAGCCCAAAATCAGCAACA  AGCATGTGCACAACTTCCCTC  CACTGTTCTTTCTCCGGCGT  GCCTTTGATTGGGTCAGAACTC  CTAACGCGTCCAGGACAAACC  TCTTCATAATATGCCATTGT  TAAAAGCCATCATCAATAAG  CAAGACCACTCCAAAGCTATACAC  GTTAGTTGGACGCGGAACG  CGCGGCTAACGTCAGATTC  AATGACTGGTCACAACGGACAC  **CGGGGTACC**CGATTCACGCTTCCTCAACACATACCTT  **AAAACTGCAG**TTAGCGGGGTTAAGATATATTCGCCAAAAG  **CGGGGTACC**CGATTCACGCTTCCTCAACACATACCTT  **AAAACTGCAG**ATGGAGATGAGACTTTTGAAAACACACCT  **TCCCCCGGG**TTTTTCTTTCTTTCTTCAAAGCTGTC  **AAAACTGCAG**AACTGGATATCTTGCGGATCAA  **CGGGGTACC**ATGGAGATGAGACTTTTGAAAACAC  **AAAAGTACT**CTGCAAGACGGACTGAGTTAACA  **CGGGGTACC**ATGGAGATGAGACTTTTGAAAACAC  **CGCGGATCC**TTGAAGGCACTCGAGGACG  TTCTAGCTTCGTTAATTCGCCG  TCCCACGGCTCACTGTTCTTTC  AGACAACGAGAGCAACCTCAGC  TAGAAGATGGGAAGGGGTAGGG  TGGAATCTAGCGTCGGAGACAA  TAACGGCTGTGTCGGTTCCA  GCAGAAGATCCTCACTATGGCC  ATGATGTCAGAGGCAGATGGTG  TGAATCGGCTGGACTCTGTATC  CCAGATGGAGTACCTCATGACG  ACACAACGAACCGTCAAGTCAC  CAACTTGGCCGATTCTTGCT  TCACCATCATCTTGCGAACC  TATCACTCACGCCAATGCCA  TCTGGTTGGTGGACTACCTTCTT  AGTCGTTTGGGAGATCCTGCT  TCCTGCTTCACAGACAGCAGT  CCCTTACTTCTCCCGTTTGC  GCTCCAGCTTACAACTTCTTCG  GAGAAGCACAAGAGCCTCTACC  CCGCGTCATCAGACCAGCT  GAAGCCGTTAGCGTTCACGA  ATAAACCGCTGAAACCGAGG  TGAACGGTTTTATGTCAGGGC  TCTGCACCTGAGGGTTTTTTT  CAAACGGTCTGCTGAATGAAG  CAAGACGGGGGATGGAACA  CGAGAGCGTGTCCCGTAGTG  GCCTATGATGTTGTCTTCCTTGA  CAAAGCTCTTACTCGTCCTTGG  CGGCGTCGTGCTAAACTTCA  GTTCCCCGAATCAGACCTGTTC | Fine mapping  Fine mapping  Fine mapping  BAC screening  Homologous copies identification  Semiquantitative RT-PCR  Identifying the homolog of *BjMc1* from *B. nigra*  qPCR analysis of *BjMc1*    qPCR analysis of *BjMc1* and *bjmc1*  p*BjCLV1b*: *BjCLV1b*  construction  *p35S::BjCLV1b*  construction  Pro_BjMc1_-GUS construction  *p35S::bjmc1* construction  *BjMc1*-GFP construction  Semiquantitative RT-PCR  qPCR analysis of *BjAP1*  qPCR analysis of *BjAP2*  qPCR analysis of *BjAP3*  qPCR analysis of *BjPI*  qPCR analysis of *BjAG*  qPCR analysis of *BjCLV3*  qPCR analysis of *BjCLV2*  qPCR analysis of *BjCRN*  qPCR analysis of *BjWUS*  qPCR analysis of *BjPLL*  qPCR analysis of *BjPOL*  qPCR analysis of *BjKAPP*  qPCR analysis of *BjBAM1*  qPCR analysis of *BjBAM2*  qPCR analysis of *BjBAM3* |

Note: Black bold letters show the protection bases of enzyme digestion sites; red bold letters show the enzyme digestion sites added to the 5’ of primers

**Supplemental Table 2.** Phenotypic segregation for bilocular siliqua in the T1 progenies

| No. of the transgenic plant | The construct of transgene-positive plant carrying | No. of positive T1 progenies | No of bilocular T1 progenies |
| --- | --- | --- | --- |
| TG1  TG2  TG3  TG4  TG6  TG7  TG8  TG10  TG14 | p*BjCLV1b*: *BjCLV1b*  p*BjCLV1b*: *BjCLV1b*  p*BjCLV1b*: *BjCLV1b*  p*BjCLV1b*: *BjCLV1b*  *p35S::BjCLV1b*  *p35S::BjCLV1b*  *p35S::BjCLV1b*  *p35S::BjCLV1b*  *p35S::BjCLV1b* | 10  15  6  10  7  9  6  9  11 | 0  0  0  1  3  8  6  4  7 |

**Supplemental Table 3.** Parameters of site-model tests on *Mc1* genes in land plant lineage

| model | Ln likelihood | Parameter estimates | Model comparison | Positive selection sites | 2△lnL (p-value) |
| --- | --- | --- | --- | --- | --- |
| M0: one-ratio | -68595.392871 | ω0=0.18666 |  |  |  |
| M1a: nearly neutral | -66750.588095 | ω0= 0.10632 p0= 0.65798  ω1= 1.00000 p1= 0.34202 | M1a Vs M2a | None | 0.00(P=1) |
| M2a: Positive selection | -66750.588095 | ω0= 0.10632 p0= 0.65798  ω1= 1.00000 p1= 0.27844  ω2= 1.00000 p2= 0.06358 |  | None |  |
| M7 | -65580.169131 | p=0.45028 q=1.36586 | M7 Vs M8 | None | 0.0086(P=0.93) |
| M8 | -65580.173493 | p0 =0.99999 (p1=0.00001)  p=0.45029 q=1.36589  w=5.84507 |  | None |  |

**Supplemental Table 4.** Parameters of branch-model tests on *Mc1* genes of ancestral branches of all species representing the main lineages of land plants

| Tested branch | model | Ln likelihood | Parameters | Model compare | Positive selection sites | 2△lnL (p-value) |
| --- | --- | --- | --- | --- | --- | --- |
| Branch I | one-ratio | -68595.392871 | ω= 0.18666 | one-ratio  Vs  Branch model | None |  |
|  | Branch model | -68588.823012 | ω_0_=0.18512 ω_1_= 999.00000 |  | allowed | 13.1396 (P<0.01) |
| Branch II | one-ratio | -68595.392871 | ω= 0.18666 | one-ratio  Vs  Branch model | None |  |
|  | Branch model | -68572.239040 | ω_0_= 0.18269 ω_1_= 2.31443 |  | allowed | 46.3076 (P<0.01) |
| Branch III | one-ratio | -68595.392871 | ω= 0.18666 | one-ratio  Vs  Branch model | None |  |
|  | Branch model | -68595.228556 | ω_0_= 0.18705 ω_1_= 0.15682 |  | Not allowed | 0.3286 (P=0.5664) |
| Branch IV | one-ratio | -68595.392871 | ω= 0.18666 | one-ratio  Vs  Branch model | None |  |
|  | Branch model | -68591.549342 | ω_0_= 0.18841 ω_1_=0.12893 |  | Not allowed | 7.6870 (P<0.01) |
| Branch V | one-ratio | -68595.392871 | ω= 0.18666 | one-ratio  Vs  Branch model | None |  |
|  | Branch model | -68591.536621 | ω_0_=0.18782 ω_1_=0.09597 |  | Not allowed | 7.7124 (P<0.01) |
| Branch VI | one-ratio | -68595.392871 | ω= 0.18666 | one-ratio  Vs  Branch model | None |  |
|  | Branch mode | -68588.872703 | ω_0_=0.18777 ω_1_=0.06741 |  | Not allowed | 13.0402 (P<0.01) |
|  |  |  |  |  |  |  |

**Supplemental Table 5.** Parameters of branch-site-model tests on *Mc1* genes of dicot lineages and monocot lineages

| Tested branch | model | Ln likelihood | Parameters | Model comparison | Positive selection sites | 2△lnL (p-value) |
| --- | --- | --- | --- | --- | --- | --- |
| Branch I | Branch-site-model  (ω_2_=1) | -66741.121701 | p_0_=0.43026 p_1_=0.22327 p_2a_=0.22811 p_2b_=0.11837  ω_0_=0.10493 ω_1_=ω_2_=1.00000 | Branch-site-model (ω_2_=1)  Vs  Branch-site-model (ω_2_>1) |  |  |
|  | Branch-site-model  (ω_2_>1) | -66724.236936 | p_0_=0.63019 p_1_=0.32179 p_2a_=0.03179 p_2b_ =0.01623  ω_0_=0.10553 ω_2_=999.00000 |  | 139 F  899 V | 33.76953  (P < 0.01) |
| Branch II | Branch-site-model (ω_2_=1) | -66737.054455 | p_0_=0.55162 p_1_=0.27792 p_2a_=0.11335 p_2b_=0.05711  ω_0_=0.10564 ω_1_=ω_2_=1.00000 | Branch-site-model (ω_2_=1)  Vs  Branch-site-model (ω_2_>1) |  |  |
|  | Branch-site-model  (ω_2_>1) | -66750.588111 | p_0_=0.65798 p_1_=0.34202 p_2a_=0.00000 p_2b_ =0.00000  ω_0_=0.10632 ω_2_=1.00000 |  | None | 27.067312 (P < 0.01) |
| Branch III | Branch-site-model  (ω_2_=1) | -66750.588100 | p_0_=0.65798 p_1_=0.34202 p_2a_=0.00000 p_2b_=0.00000  ω_0_=0.10632 ω_1_=ω_2_=1.00000 | Branch-site-model (ω_2_=1)  Vs  Branch-site-model (ω_2_>1) |  |  |
|  | Branch-site-model  (ω_2_>1) | -66749.178168 | p_0_=0.65703 p_1_=0.33650 p_2a_=0.00427 p_2b_ =0.00219  ω_0_=0.10676 ω_2_=998.99996 |  | None | 2.819864  (P =0.09) |
| Branch IV | Branch-site-model  (ω_2_=1) | -66750.588090 | p_0_= 0.65798 p_1_= 0.34202 p_2a_= 0.00000 p_2b_= 0.00000  ω_0_= 0.10632 ω_1_=ω_2_=1.00000 | Branch-site-model (ω_2_=1)  Vs  Branch-site-model (ω_2_>1) |  |  |
|  | Branch-site-model  (ω_2_>1) | -66748.109059 | p_0_= 0.65119 p_1_= 0.33946 p_2a_= 0.00615 p_2b_ = 0.00321  ω_0_= 0.10581 ω_2_= 52.56187 |  | None | 4.958062  (P < 0.05) |
| Branch V | Branch-site-model  (ω_2_=1) | -66750.588143 | p_0_=0.65798 p_1_=0.34201 p_2a_=0.00001 p_2b_=0.00000  ω_0_=0.10632 ω_1_=ω_2_=1.00000 | Branch-site-model (ω_2_=1)  Vs  Branch-site-model (ω_2_>1) |  |  |
|  | Branch-site-model  (ω_2_>1) | -66750.588095 | p_0_=0.65798 p_1_=0.34202 p_2a_=0.00000 p_2b_ =0.00000  ω_0_=0.10632 ω_2_=1.00000 |  | None | 9.6×10^-5^  (P = 1) |
| Branch VI | Branch-site-model  (ω_2_=1) | -66750.588095 | p_0_= 0.65798 p_1_= 0.34202 p_2a_= 0.00000 p_2b_= 0.00000  ω_0_= 0.10632 ω_1_=ω_2_=1.00000 | Branch-site-model (ω_2_=1)  Vs  Branch-site-model (ω_2_>1) |  |  |
|  | Branch-site-model  (ω_2_>1) | -66750.588096 | p_0_= 0.65798 p_1_= 0.34202 p_2a_= 0.00000 p_2b_= 0.00000  ω_0_= 0.10632 ω_2_= 1.00002 |  | None | 1.0×10^-6^  (P = 1) |

**Supplemental Table 6 Results of BlastN searches using sequences from the fragments of molecular markers linked to *BjMc1* gene**

| Markers | Linkage group（position） | Identities |
| --- | --- | --- |
| EA03MC07 | J17(7898531--7898229) | 537，2e-151,295/303,97% |
| EA10MG02 | J17(8964287--8964433) | 291,2e-77,147/147,100% |
| A13MC02 | J17(21703564--21703394) | 283,5e-75,164/171,95% |
| EA14MC05 | J17(6893319--6893664) | 638,0.0,338/346,97% |
| EA16MC09 | J17(21374205--21374371) | 331,3e-89,167/167,100% |
| EC03MG14 | J17(16753383--16753687) | 605,e-171,305/305,100% |
| EC13MC15 | J17(17339917--17339349) | 1128,0.0,569/569,100% |
| EC14MC03 | J17(17493689--17493852) | 235,1e-87,164/164,100% |
| SA01MC05 | J17(6806691--6806764) | 452,e-125,228/228,100% |
| SA08MG14 | J17(38035748--38035519) | 194,4e-47,201/223,86% |
| SA13MG16 | J17(38139981--38139721) | 494,e-138,258/261,98% |
| EC14MC14 | J17(7604632--7604484) | 264,6e-69,145/149,97% |
| EC08MG05 | J17(16761515--16761630) | 222,1e-56,115/116,99% |
| SC40 | J17(12373616--12370879) | 5428,0.0,2738/2738,100% |
| SC151 | J17(12340431--12340212) | 422,e-116,220/221,99% |
| SR52 | J17(12307185--12306928) | 448,e-124,250/258,96% |
