# Supplementary material for: Trilocular phenotype in Brassica juncea L. resulted from interruption of CLAVATA1 gene homologue (BjMc1) transcription
Source: Sci Rep. 2017 Jun 14;7:3498. doi: 10.1038/s41598-017-03755-0 (PMC5471281; doi:10.1038/s41598-017-03755-0)
Supplement: Supplementary file 2 — Supplementary Datum [file 41598_2017_3755_MOESM2_ESM.doc]

**Trilocular phenotype in *Brassica juncea* L. resulted from interruption of *CLAVATA1* gene homologue (*BjMc1*) transcription**

Ping Xu^1^; Shiqin Cao^1^; Kaining Hu^1^; Xiaohua Wang^1^; Wei Huang^1^; Gang Wang^1^；Zewen Lv^1^; Zhongsong Liu^2^; Jing Wen^1^; Bin Yi^1^; Chaozhi Ma^1^; Jinxing Tu^1^; Tingdong Fu^1^; Jinxiong Shen^1 ⃰^

**Supplementary Datum**

**Supplementary Data 1:** Sequences of scaffold 1, 2, 3 and 4 of 83D02

scaffold 1

CTTGTCGTCCCAACTGAACCAAGTCGGGTTTCCAAAACTAGCAAACTCATTTCCATGCTTAGTGACATAATCTAGATAAGTTTTGTCCTCCGTCGCGTGGTACAACCAGCTAGCAGCCCATAGAAGCTCGTCACCATACCCCGTGGAATTGTAAAACTTCTGAACCTCCGGTATGTTGACACTGTAAGAGCCTCGTTTAGCATCAGCGAAATCAAACAGCTGCTTGGCGTGTTTCAGAAGCGTGGCTGAGTATTGCGGGTCGCTGTCTTTGAACACCAAAGACGCTGAAGCCATGGCTGCAGCAGTCTCCGCAGCAACCTCAGTCCCTGGAGTGTCTGCATCGATCTTGATGAGTGGTCTCTTCCCTTTCATTGTCTCAGGTCTCTCCCAGCACGGATGATCCACCTCCGGATCTCCCACCTGGATGTAGAGGACGTTATCAGAAGGATGAGCTTTGATGAAGTAGTCAGTGGTCCATCTGAGGGAATCTTTAGCAGGATCCAATAAGTTCACAACATCCATTTGATCAGCGTACTCAAGAATGGACCAAGACAAGACGGTGGCAGTGAAAGCCATAGGGAAACCAAACTTGATATGATCACCAGCGTCATATAAACCTTTGGAGAGATCAAGATTCGCTTCACTTCCATCTTTAAGACCTGAATCTCCTCTCCAAGGTATCGTATTATTCACCAACTTGCCAGCTACATCATCAAACAATCAAATTCATCTTAAAAAAAAAAAAAAAAAACAATGAGGAAGCGTGGAAACGTGGAAGCACGACGATAATAGAAAATGGTTAAAAAAAAAAGGATCATGAAGGAGAGAGGTTTTAGATACATTTTTGGATGTCGAAGAACTGTAAGGCGAGTTTGAGAGCGTCGGCGTATTTCTTATCGATGGCGCCGGGAGGTCCAGGGACGGGACCAGCTCCCTCGTCGTCGTCGTCGGATTTCTTCATTTTGGATTTGACCATGTAGACGACGGCGAGGATGACGGCGGCTAGTACGATGAGAGCTACGAACCAACCGAACCATCCTCTCGACTTCGATTTCTCCCCCATTGTGTGATAAGAACTGCCACCAGAGAGATCGGTTAGATCCGGTTCAATGCAGCTGCGTACGGCGATTGGAGTAGAGGAACTAGTCTAGCTCGCAAGATCTGTGGAATCAGCGAGATGGATTCGTGTTTGTGAATTGTGAGGAAGAATTTGATGGAGACATGTCACTGTCACTACTTCAAACCCAGTGGTGGTTGTTATGTCGGTGGAGTATTAAGCGGGGGCTTGTTTCAGATCTTCCCCTCCCTTTCCCCCTTTTTTATATTTTTTTTTTCTCTCTGTTATATAATCATTATTGGGCTTTTCTGGGCCTTAGAGTGTGAAAGTATAATTTATTACGACTTACAGTCGCGTTAATTTATTATTATCATCACATGGTAAAATTTCTAGAAAAAAAATTAGAGTAAGTTGATAATTGTTGTAAATAGAAGGTGTGAAAGATGTGTAAATCTTATTCTACTTATTCCATTCGACCAGATGTCTATATATATATACATGAGATAAATCTTATNNNNNNNNNNNNNNNNNNNNNNNNNNNNNNNNNNNNNNNNNNNNNNNNNNNNNNATGTAAAAGGATAAACATTAACATATAGATAAATATGGAAATATCTCAATCTTCTAAGGCATTAGAGATTGGGTCTGCTGGATGGATGAACGGGTTTTGATGACTTGGGCTTGTTCATGGGTCGATCACCAAGTGATCCTTAACACTCCCCATTGATCGATACATCCAGGCTCAAGGTTCATTCATGCTTTGATGTAGCCTCATTAAAACCTCTCCTGAAAAACCCAAAACCCAATGTGGTAAAAGGGAAACCAAGGACAGGAAAAAGAGTACAACACATGAACTCCCCCTGATGACTACATCTTCGAAGATCCTTCTATTTGGTGCACTCCATTCTTGTAGATGATCTTCTTGCTTGTTGATGTAGCAATAGTTCCTTTGTCGGATTGTAACATGTCTCCCATATCTTCTGGAACTCCATTGAGACATGGTCAAGACGTGTAGCCCTTATAGCTAATCACTCCGTGAACTGGTCTCTCTATAGCTGGTCACTCCATGTGATGGTCGGACGGCTAATGGCTTGTACTTGGATAGATTTTCAATCCTTGATCAAACTGTACCTGCAGTTTCTGTTATGTCCCACATACATATGTATATATATAGGTCATATGGCTTAGCCATACTGTTGACACTTTAATATATACTTGGTCATCAAACTATGGATCTATTTAGCTTTAACTAAATATGTTTTCTGGTTACACTCTTGTACCATTGTGGATTTCTTTTATCCATTGATAAATATAAGGGTTCCTTTATAATCCCACACAATAATCTTTATTTAAAACAAAGTGATCCAAAGTTTTCCTTTGATGGCTTCCCATGACCTTCTTTATTTTAGATCAAACCACGTGCATATGCATGAGGGTCAGGTTATGGACTTAGTCCATTCATGCCTGCATCTCGCCAATCAATGGTCGAGGTATTCATTGCTCAATGTTGCTGTAACAACATGGCTTATACGTGGTGATTATATAAGTGAGGTTTGCATACAACCTATGCTTCCTTTAGAACCTTATTTCTATCACTTATACTTCAGATTATCTATACTGATAGTTAGTACCTTAAGTCCATTCCATGACCTAAGCTCATATCCTTTTGCCATATGAATATTTGAGCTGAAAACTAAGTCGGACATGGTCATGGTCGGCAAGGACTTAATGGCGTGGACCATATGACTTTCTTTGCCTTCCCTTGTGGCCATCTTCCACCTCATTATATGGTTTAGGACGTGGTTATCACTTATAAACACGCCTATGGTTTGCTGCAATTTTATTGCAGTCATAACACATATAAATATGGTCTGTTTATCCAATGGTTTGAACCATTCAAACTTTCTATGGATTTGTCCAGTATATACACAAGGATTTTCCTTCATAATTTATGGACTGGTTGTTCTTGATAGAACTTATTTACTAAGTATAACTTAGTCCTTTATATACAGCTGTTTCTAATACAGTCCATGAACAGCTCGGGAACGACACTGTTTTATGAGAATTCCTTTTCTCATCCTTTAAGATCTCTTGGATCATAAGTTATATAGGTACCTCTATATCCTTTATCCAGTGAATATCATAGTGCTTACTACTTCATTATTTCTTTCCAGACCTTATCCAATTTTGGATTGTAGTAGCATCCACCACATAGGAATTTATTTCCTTGTAACATGTTCCTTAGTCCTTTTGGATAACCTTGTGTATTAAGCTATTCATTGAATGCTTTAGGTATGGACATGGACGACCCTTTGCCTATCCGGTTATGTCCTACATCTCACGGTATCACAAGACTCTTATGTCACTGGTCAGATGGCGTTTCCATATTATATATGTCCCATACGCCCTTCTCCTTTATTTAACCTTAAAAACCTCACGTTCCCTTTATGATTTTATGAGTACTCTAACGTGGGTTAATGATCCTCTCTTTAGTCAAGTGCTTTCTTTCTCATGAGCTTTATGTATATATACATCATTGGTGTTAACACTCTTATGTTGTTCCATTATGTTCCAGACATGATCTTGATCAATTTGAGATTCTTATCAAGGACCTTTGTTTACCTTTATAGCTTGGCGTCCCAAGACTATATGGTTTGGTACCTTAGATGTTTAGCTGCGCAGCCTTTTCTCAATGTCTGTTCTGGTTTCCTTAGTGAACCCGGATCCTTTATTATGAGCACTTTTTATCTTTCTATCTGAGGTTCCTTTTCTTATGGAACATACTCGTCTATCCTTTAGACTTTATAGCACTTGATTATGTCTCTACCAGGACATTTATTTCGTGGTGCTAAGCTGGAATGACTTAGTCATTGGGTCTAATCTGTCTGGCTATCATCTAATATGATTTTTCTTAGTATGGACGTCCATGATTTATAATCTATGGTCCGAGGATCTTGCCAAGACATGGATGGTTCATACCAATCCGTTTCATATATTATTTACCAGCTTATAGCTTTCTCCATTTAATGTTGGATATACGGATATACAATCCGTGTCCTTTAACCATAATCTAATCACCCATGTTAGGTTCATGGTTCCTTTGGATTCGTGGGAGAAAGATATTTTCCTTATCCAACATATATTTCCATTCTATCTCATTTCCTTATGAGGTTCCATTCTAGACGGCACATATATTTGTGGTGGTCTATATATAATCTCTTAGGATGGTGTCTGGATTATGACCCTTTATCATACTTTAGATGGGAATATCTATGCTTTACTTTAATGGTCTGATGCATCATTCCTTTCATACACATGTAAATACTTTGATGTCTCCAAGCATATAGACTTTTAGTCTTTAAGATTAATATGTCCTTAATCAAATATCTTTGTGACCTCTGTATGGAGAGTCAGAGAACTAGAAGTTCATATATCTGATTGTCTTTAAAGGACAATATGTCTGAGTTTAACTTGGCCGCTAGATTATGGTGTGTTCATGTATCATAGGGTTATCCTCTCTCCCCCTCATGAACATACTGTGATCTGCGGATGCTCATGACTTTTCTCATGACTTTTATAGTCTAAACCTCATAGGTTATGGCTCTATAGGCCAAGTTATCACGTTTTGGCTATCATGTTCATGGTTATATAGTATGGTCATGTGCCACACGGAGTGTCTATTCTCCCCCTCATGAACATGCTATAAAATCGTTTGTCCCTTACACAAAGATAATATGCCATTTCGTGATGCTTTGTGACAATCAAACCTTATGAATTTCCCTTGTGTACATGTTTCACAATGTGAGATTTATGGGATAATTTTTTTGTGCCTTTAAACTTTGCATCAAGTTTATTTTTCGATCAGAATGACTAATTATGTCGTACCATTAAGTATAATTTCGTGGTCAACCTTTCAGGTTACCTTAAAACCACTTGTTCCTTATCATATTGATCCCTTGCATAGTCTAGATCAATATGGAGAGTAAAATCTTGACCCCTTTGGCGATTTTCCTTAAAAAAAATCTGTAGGATTTTTCTTCCCTTAATCCATGAAAAATCTGTAGGATTTTTCTTCCCTTAGTCCATGGAAACCATTTTTCTCTTGAGTTGATCATACTGATCCCAATACTCAATAGGACTTATATGTGTGGTTAAAAAAAATCTTTGGTCAAGAAGCTGATCCCATGTTTTCATTTGTCATAAGAAAAAAAAAATTTCCATCAAGCAAATAAATAAAAACCTTTTATTAATGCTTTGTAAATGACAAGTTAAAATGAAAACAACACCAAATCATAAAATAAAATTCAGAACATCGAAATCCCTTTATTCGATGAATAAATCAAGATCACTATCCACGTGAGTCCACTCGGATTCTTCCTCCTCAGCTTCATCAGCTTCAGGAAATATCTGATCAAATTCTTGTCTCAGCCTGTGTATGTACATGATCTCATCAAATTCCTTGATGTTCTTTGCTCTTATCTGCTCCTTCTTCTCAACCGCCAATAGGATGTTGAGTAAGTCGAAGTAAGTGGTGCACATACTGGCGTGATATGATAACCTCACATCCTGCGGATGGATCGTTTCACGAGTCTTAACGAGCTTGTCCTCGTTGGTTATCAATTCACCACACAGATTAAGAGTATAAGAGATTCTCATAAGGTCAGAATGATAGTCTCCCACGGACTCATAATCCTGGAACCTGAGCTCACGCCATTCTCTTTCAGCTTCATGTACTACTGATTCTGAGTATTTGTTATTCAATGAATACCAGAGATCATGAGGATCCTTAATGTACTTGTACATCGTTCTCAGTTCCTCACAGAGATGATTTTTCATGATTCTGAGGGCCCATTGCCTCTCACACGCAATGGTATCATTGTTGAATTTGACACACTCCTACAGTCCCCTAGACTTTAATGTGCCAGAAGTGTTCATGGTCCATACAACATAGTTGTCTCCGGAGAGATTTAGGACACTGTATTCCGAGTTGTCTGATCTCGACATCTGAATCATATTAAACAATGATCAGTTAGTTTCTCATGCCATATGGCTATGTAAGGAAACTTAGGCTCCCACGGCCATATAGACAATAAATGTCGTTTGATTATAAAGCCATTATATAATCAAGGCTCCCATGGCCACTCATCAATATGAGATTTCAAGCAAGTGATTTAAACAATCTAGAACTAAATAATAGATTTATAAATCAAACTATATGACCAAGATGCTTGAATCAATCAATAAAAATCTCAATAATTATTCAGATCAAGCAAAATAATTTACTAAGTCGACCAGGATGCGAGCAATCAACACAAAATATCAAACATGTAAAATCGGGTGATATTTGGTTTTAGGGTCCGCATGGGGTCGAGGGTCGAACCAGGTTCAGGGTTAAACCAGGGTCGGGGTCGAGCCAGGGTAAGGGTAAGCCTGGGTTTGAGGATAAACCCGGGTCAGGGTCGGAGGTTAAACCTCAATAAGCCCGTGTAAGGGTCAGGCCCGGTTAGGGCGAGGGTTAACCCCGGGTAAGCCCCGGGTTTGGCAGTTAGAGATTGTTTTCGATAGATTTCATGCTTTAGTTTATAAACAATATGGTTTTGTTTATCTGAATGATACAATCCCAATTCAGATGTGATTTGTAATTTCGAAATATATAACCAATATTTTCAAAAAAATAGTTTATGCAACCTTAGCAAAACTCGGATCATATTATGGTTTTAGTCGAATGATGCAAACAAATACACAATGATGCAATTCAGAGTTCAATCATGATCTAGGTGATTCGACAATATGATCAAACAATCAAGAGGAATTGATTTTAGTTAAGCAACCTTAGCAAAACAGTTTCTAGGTGATTTATGCAATCAAGTTATTGAAAAAAATAAACAATACTAGATGCAAATCAAGGAAAAAATTCGAACCATATTAAAAACTCTTTTAGGGTTTTCGAAAATTTGGTTTAATTTCAGGACTAGCATATTGCAATTTAACAAAATCTAGCAATTTCAGAAATTTAACAATTTCACATAGTCTCAATATGGAAAAAAAATAAAATACCATATTTCCAAATTTTAATCAGTTCGAAATTTTAATATGAAACTAGAACCTCAGATTTGACAAATTTCTATTTTTAATCAATCAGCAAAACAATATGTAAATTGTGCTAAAAAATAGAAAATCGGATTTAAAAAAAAAAAGGAAAATTTCGATTTTAATCAACTTTGTCAAATTTTAAACTTCCAATTCGATTTAAACAGTTCCAAATTACTCAATTAACAATTCTAGAAATCAAATCAAGCAATCAGGAAAGTAAAAAAAAAAACAAAATCAAGTTTAAATAAATTAGGGTTCTTGAAAAATTCGATCAAAATTTATGTTTTTAAGGGGTTTGATCGATTTCTTTTTGGTTTCAGGTTTGGGGTTTGATTGAGTTTTCTCAAATCAATTGTTTTTCGAGATTCAGAAGCAAATATTGCTTTGATTTTAATTGGTTTCCCAATCTCTAATCGAACTTATGGTTAATGAGCTCCGATTTACTCATGGAAGAGATTGGACTTATTAAAACTAGGGTTTTTATGGGGTTTTGCAAAGTTTTAGAAGTCATATCCTTTAATCAATCAATCTCAAATTCGATTATGGTTCTCTGTAGAGTTTCAGATTTTATTTACCTTAACTTTGAAGAATTTGAACTGGACCACCGAGATAGAGAAGTGATGGAATTGAATTTGTCCTAGTTCCTTGCGCAGGTACAAGTTGACGGCGCTTCAAAGAACAGGGGCTAGAGGTCGCGTCCGAGATCGTATGGAGTCGAGGTCGGCGGCGTTGGATTCCTCTCTTCAAGAGCTTTGATTCGGTAGGTCGTTCGTCTACTGGTTCCTTGTGGTTGAGGAGAACGAGTAGATTGAAGGTGGCGGCCGAACTAGGGTTTTAGAGATTTTTAGGTTAACTCGAGTTTTTGAGGCTCGTGTTGATAACGTGTTGTAAATAGAAGGTGTGAAAGATGTGTAAATCTTTATTCTACTTATTCCATTCGACTAGAGGTCTATATATATACATGAGATAAATCATAANNNNNNNNNNNNNNNNNNNNNNNNNNNNNNNNNNNNNNNNNNNNNNNNNNNNNNNNATGTAAAAGGATAAACATTAACATATAGATAAATATGGAAATATCCCAATCTTCTAAGACATTAGAGATTGGACCTGCTGGATGGATGAACGGGCTTTGATGACTTGGGCTTGTTCATGGGTCGATCACCAAGTGATCCTTAACAATAATAATTGAGAAAAGCTGATTCATTTTCCTGATAAGTAGTAGTATTAATCTGAACTAAAAAAGCAAGTTAACTTTGGAATTATTGGGACTAGGACTAAAAAGTTGGCTCCCTTGCAAATATGCATTTCCTGAGCTAATTTCGATATCTTGTTCTCATTCTTTTATATGATTCAAAAACAAAAGTGGTGGTACGTCATTTTCTGATCACTCTCTGGTGTAAAAGTCACTTGTCTTGTATTTGCGTCTCGGATGATGATGTTGGATACTGAAGCTGATCAACAACGATCAAAGCAAAAAAGCGACCATCGAGAAGGAGTTTGACACCGTTCACTCAACAATGTATTCGACAGGAATAACACCCGGAGAGAGTTCACCGAGCAGACGGAACTCAACTGCAACAACAATCAACTGAGGAAAAATCTTGATGTTCTGACACAACTGCAAATGTATCGCAAGTTGGCAGAGAGAACTGGATTCAAACACTTCTTTGATCCAGCAGCAATGGAAATCGTAACAGGTCCATGTCAGTTCTGTTGACTGCTCTGAGGTCAGGGATAATTGATTTGGAAACGGAAGATGACAGGTTTAGTATCACACAAACTTCATCAACGCGCTTGAATGAAGGTGTTTACTTTGCCGCTTTAGACCTGTTTGAGAATCCTGGTCTCAGAGAGAGACCTTTATCTCTCTCTTAAAGCTAACAAGTTACACGCTTGACAAGTTCAGTGCAGGAAGCTTACTCCTTCAATTGTTACAAGGTCATGATTTCTCACATATCTGGTGAACAAAAAATACAGTATTGTACAAATGTAGACACACAATGAAGAAAGGGAGGAGAAGAGACTCTTTGATATGGTTATCTGGTGTGCAAGATATCTAGTCATCATCCTTGAACTCCCTGACCACCAGAACAGAAAGAACAACAAAATGTGTGTTAGGATCAATACAATGTGCCAAAAAAAAAAAGAACAGAGTCGTGTACCTTCGATCAAGGTATCGAGGTTGAACACCAGAGCCTTGACAAGTAGTGCAGGTTAAAGAACCAGCACCATCACAGTTGATACAGCTCGAGACTTCCTTCTCTCCTCCACCAAGCTCCACCGTCACGTTACCACTTCCCACACACAACCTGCACTTCTCTGCTCCATCAGCAAGAACACACATAACGTTCAGCTCATAAACATCACAAAACAGGTCGAGAGAGGAGAGATTAGAATCGAATTCACATACGAGCTCCAGTGCCATTGCAAGGAAAACAAGGTTGAGTGTTCTCTCTCTTAGCCTATGTAGAAAGATAAACACTAAACAATGTTAAACTTCATTTCACACCAATGCACTCACTCAGAAGCATAAGCTTAACTAAACCTAGCTATGGATGAACACAGAGAAGGGAACGTGAAGACTCACGGCATTATCGATCTGCGTCTCGTAGAAGACAGGGATTCCGATTCCCAGTGCGACGCTCGCCACGCCAACAGAGATTGCGATTACCTAATAACGAAAAATCAAAATCGATAATCACTTGAAGACAGTGTGAGAAGGTATTCTGTATATAGCAAATAGATGTGGATCTAACCGTGTTCGTATCGACTTCGGATTTGATTCGTGGATAAGATGTTGCCGGTGGCCGGAGATTCCTCGCCGAGAGTAAACGGGAAAAAGACGGAGTGGCATTGATTGGACGGTGGATGAACGGAGATTGAAGACGAGGCAGAGATGGAGATGATACTGGCATTTTCGCCTTTGTTTGGTGTGAAGAAGCTACGCAGAACTTCGCGTGAGCGCGCGAAGATGAGAGGAGGAAGACGAAGAAGAAGAACCAGTGGTTTTGGGTTGATCTCAGCCACTCGATTACTCACTGCGGCTATGATTTGTCCACGTGGCCTATTCAGTTTTAAAGATTTTCTCTTTTAAAAAATTTAGTAATTATCGCCTTATCCGGTACACACCCGCTCAAAAATGTAGAAGATCGTAACCGTCATCAAATGCTAAACCAACGGTTGAAGTATAAACCGAGCACTGTTTCAAGTATCAACTTACAAGTAGCGAACAAAATCTATTTTTGATAAAAAAGGATTCGCAAGTCACGATCGATACGAAACGCAACCGTTTGCTTTTGCCTCTTCCTCTTCCTCTTCCCTTTCTTTCTCTATACTTAAGTTTTGTTGTCCTTATCATATCTCCCCCCTTCTCCTTCTCGCTTTCAACGCCGAGAAACGTAAACGTCGCTGTTCAAATCGTTCCTTCTTAGAGATCTCCTAATCATTCTTGACGTTTTCTCATATAAGGTAAGTTAATTGATCAACCGTTTAGACAACTGCTTCTCCATATGTTTTCACGAGTTGTGTTCGGTAGTAAAATTTTATAGGACGGCTTTTTCGTCTATGAACTTAGACATCATATGATAAGTTTTAGTCATCAAACTATGCACTACTTGAAACAATCCCTTATATGAATTGTAGTTGAGACGTAACGTTTTTTTTTTATTACCAGGGTGGCTACAATGATTGGATCGTTTCTGACAAGAGGCCTTCTGTATGCTATACTTCTTCAAAACCCATGATCTTTGCTTTTTGAGTTTTTAATAACCAAAGTGGTTTGAAACTGACTAATGATAAATGTTTTAACCGCTGACAGAATGGTGTTTGGATATGCATATCCTGCTTATGAGTGTTTCAAAACGGTCGAACAGAACAAGCCTGAGATTCAACAGCTCCAGTTCTGGTGCCAATATTGGTAATGTCTACATATGAGATAGGTTCAGTCATGTATTTCTTTTCTCGACCGTCCGCGTAGTACCTTGAGGTTTACGTTATTGTACATCTTGTAGGATTCTTGTAGCTGCTTTGACAATCTTTGAAAGAGTTGGTGATACTTTTGTTTCCTGGTAAGAATCTCTTATTGAGTCTAGGTAAGAGTTGCATCATGTTGTTGTAATCTTTCTTTTCACCATCATTTTTTTTTCTTGATGAAGGTTACCAATGTACAGCGAAGCAAAGTTGGCATTCTTTATTTACCTCTGGTTCCCAAAAACCAAAGTAAGTAAAAGCCCTTTTTGAGATTGAGAAGTATTTTTTAAAAATCATTCTGATTCTACTGGCCTTATATACTCTCTAATTTCAGGGAACCACATACGTTTACGACTCTTTCTTCAAGCCATATGTCTCAAAGCATGAAAACGAAATTGACCGTAGCTTGACTGAAGTAAAGACCAAAGCTGGAGATATGGCAATGATATATCTCCACAGAGCAATCAATCATGGACATACAAGATTCTTTGAGATATTGCAGTATATTGCTGAACAATCATCACCCAAACGTCAGTCTAAGGTATTAATTAATGCTAATTTAGTTCCTTCACCTCTACGTCTCATGTGTTTTGCTGTCCTGATTACGGTAATCAATTTTTTCTGTTTGTTAACAGGAAGAGAAAGAGACAGCAACACCTGAACTTGGTGATCCAACTCTGAAGATGACACAAAACAAAGACAAGTTACCTGAGATAGAATCATCCACGAAGAAAGACTAATTAAAAGGCTGACTTAGGTGGCAAAGTATTGAGGATTTACACATGGTTCAATGCAACTTTGTATTTTACCTTTTTATTAACATTTAATAGGAACAAATAATATTGAAACATAAATAAAGAATAATTTTAATATCATACATGGTTCGTGCAAGTTTCATCATATTCAGATGTAAATGGCTTAGAAAAAATAATAATAACTTTGAGGTTCTAGAAATGGAATGAGAATGGTATTGAAGTTGGTTTATCATTATTAGAACCCGGCTTATAATGGGCCTAGACCTAAATCCTTCTCTACCCAATATTGGCCCAGTCACTTGCCCGTCCATGTCACCATGGTCGTATCTTTATTTTAAAACACTGTACAAAGTAATGTAAAGAATGAAAGGGCTGTGATAAAAAAAAAAAAGAATGAAAGGGCTTAAAGTAAAAAACGATACACAACCATAGGACCACACGGTTACAAGTTGTGACATATCATCGATGAATTGTTAAGCACATCAATATTTACACAATTTTCTAGAGATATTTCATAATGTAATGTACGCACAAGTAACCAAATTAATTTGATGTAACATGTAAAGAAAGTCCCAAAACAGTGAGTTGTCGAGAGACCGATAATTTAAGAAGGAAACGAATGATAGGTGAAGCCACTATTAAGTGTTAGGGACCATTTTTGAATATAAGTTATTGTATATCCTATCTTCTCAATCAATTGCATTAATTGATGATATGAAGTTTGGTGTGTGATTCAGTGTGAAACTAAAAGAGTGTCAGCAAGAACATCTTATTTTTTTTTTTTGAACTAAATGAACATCTTATATTTCATCACTCTTTTTGATTCTCTCTGAGCAACACAACAAATAATGAGATAATTTTTAGGTAGTCAGTTCCAAGACTGGTCTCGTTTTCTCACATCAAATTTTCAACAAGTTAGTTGGTTATAGAGTGTGCGAGCGAAGATAAATACATTGCAGTTGTTAAAAGTTTGTTGTATCAAAATAGATTGTAATTTCATTACAGAATTTCCCGTCTCATGCTTAGGCTCTTCAAAACTTAAAGCGAGTTAAACAAAGTTAACAGCGTCAAGAGACGTTATGTCTGAACTTTATTTGAAATTCGTAAACAAAATGTTCGACCGCATTATTGTTTAGTTTGTTTGTTTAATAAGTTAATATGTTAACATTTATGATTAACCTAATGATATTCTATCAAGTTGAAACCTAAAGTGTTTATGAATTATAATATGATGATATAGTTTGTAAATTATTGAAAGTACGAGGGATCTTCAATATTTTTGGGTGGGAGATGTAATTCAATTACAAACGTTACGCAAGGGATCCCTTGCGGGGCTTGATTTGGTTCATAAACTTTATGATTATACGAAATGGGGACACATTTGTCTCCATCTTTTAATCTTTTATTTGAACCGTGTCGCCAAATCTTTTAAAACCACTAACCAACACGTGCACTTTATAAGTTTATATCACTGTAAAAGAATAGATTATCAAGAGAGTAAACAAACATTTTGTATCTAGAAATATCTCCTCNNNNNNNNNNNNNNNNNNNNNNNNNNNNNNNNNNNNNNNNNNNNNNNNNNNNNAAAAAAAAAAAGACTGAAGAAATTCATTACAAGATCCATAATTCATAGCTATATTCAAAAATATTGTGTACATGCAAAGTTTTCATATCTCTCTGTCGTGTTTTAAAAATCTTATATCTTTCGTGGCTCTATATTTAATGCATATTAAAAGCACTTGAAATAATACAAACGGGGAATTGGAAGTTCTCTTTACTGCAATACAAGATGGGTAATACTAATATTTATATCAGTTTTAAAGTTACATATGGGGAAAATTATTAACCAGAGACTAATGCTGATCATCCACGTGCTTATTAGAGATTCTTTAACATGCATGCAGGTGGAGTAAATTTTCTGTCAGTCCAATAAATTCCATTGTCACCGGAGTAACAATTGTATTTAAATGATATATGTACGTAACAATTGGTTTCGAATTTCAAACTTGTTAACATACAAACACATTCTCAAAGGTCTCGCGCTACTTTTCTTTGTCAAACGATGCCTACATTGCTATATAAATTATAAAATGTAATTATATAAAGTTGAATGAAAAGACTTTACAAATGATCTCTTGTGAATTTTACAACTAGGTTTATTAGAGTGGAGGCAAAAGAGAGGGACAAATGTGGAACACGACAAGTGAGAAGGCCAAATCGACTGCGTGATAAATCCAAATATTGCGGCGAATGTGAAGGTCTTCGATTTTTACATCCGGTCAACAGTCAATTCTAACACTTGTGATTAATCTACTTTATGGTTTTGATGAATGATTAATACAAATTACTCAAATTTTTGTGTGGTGAGTTTAATCGTAATTAATATAACAACCTAAAAAAATAAAAAATTATTAAGCTGAGTTGCATTGTCTTGTGAATTACTACTATGGTATTCTTTTATTTGGAGTACTATATTAATAAACGCAGTGTCCCATAACTTCTTACACTAACTTCAAGTGTAACTCCCCCACTATCTACAGTAGTGAAATTAATTTCTGACACCCTTAAGTTATCTTGCCCGAAATCAAATTCTTATTAACACGAAGGACAACATTGTCCAACTATAGAATACTTATACCTATATCGAATTTAACACAAATTAGTATATGTAAGTAAAGTAAAATTAGCAGAATTTATATATAGGAGATTTAAAAATTTAAAGACATTGAAAAATTCAATCCGAGACAAATAGAACATTTGAAATTCGTAATCACAGTTCCAAATACGAAGTTTCCAAAGACCTTGTCTGTTGTTTAACGTGTAATCTTGATCTGTTTAAACAATTGTTTTGTCCATACATACAAAACATTATTTTAGCAGGATTATTATTATATATTTGATGTACTAAAATCATTTTCTTTCTGGCGTACATGCATGGAATCTCCAAAATTTTAATAATAATCTACACAAACAAATATTAAATAAAAAATTGAGTATAATGATATTACCAAACAGATGCAATATTAAAGAGTTGGGATGCTTGCGTCACATTTTTTTCTTCTGCATTTCGACCAATAATAGCTTTAATGACGTTGACCAAGTCAGCTCTTAAGAAGGTCAACAACTGTAATGGAAGATTATAGGGAGATTATATGCAAGGAAGCAGTTAACTGTTAAAAGCACATACCAAGCCAACCAAGCTCGTCCAAAAAGATTTAATTTCAATAGGAGTTCCTCTAACGAATTTTCTTATGGTTTAAAAAGCAATTCATAACTCAAAACCGCATATTAAATACTGTTCTTTCTTTTGAGAAAAATACAGAATTAACTGCACCACAGCTAACAAAGCATTAACCAGATACCAAAATTAATTAGAAAGATTTTTTGCTCTGACAGAATATGATATCTGAGTATAGGTAAGTACACTATATTGCAATTAAAAGCTTATAATTAAAAATTAATGAAGGGTAAGAAAGTAAAACAGTAAATAAAGAATTAAAGAAAGATAGAGCCCACCACTTTAGTTTTCCCTCCTTATTATCCCATCTTGGATCCATCTTCGATTTCAACACCAAAATAAAAAGCTTATAATTAAAGAACCATAAAGAAACTCTCACAACCTCAAGAGCTTCTCTCTCAGTTTCAATGGCTTTACTAACCATCTTGCCGGAAAACGCAGAGCCTCCAAAACATACTCCACCGCCTAGCAAACGCAAGAAACGCGAGAATCCCACCGAGAAACCGCAGAAACCACGCAAACCTCAAAAGCAAAAAGCAGCACCTCAGAAGCAACCGTCTTCATGGGACCAGATCAAGAATCTCTTGACTTGCAAACAAATCGAAGGGTCAAGAGTTCACGACCCATCCAAGAACTCTCAACCCGCTCCGTCCACGACGTCTGACTTATCACCTTCCAAGCTCAGCTCGTCGTGCAGCTCCATATGCAGTTTTCGAGACGTGGCTCACGGAAACACTCGTGTGGTTCACAGAGCAGACAACTCTCCAGACGTAGCTAACTCGGCTACTGCTGCTGACTCGGAGACTCGTCTTCTGACTCGGAAACCTGGTCAACACGGTTCTTCGTCTCGGTCTCATACATCTGGCTCAACTAGATCTAACGCCAGCGGAAGTTACACGTCGTCCTCAACGACGTCGTTCAGAGCCATGCAGTTCCGTAAACTCTCTGGATGTTACGAATGTCACATGATCGTTGACCCTAGCAGGTAAAGAACCACACAAATAAAATTAACTGTGGTTATATTTTTGTATATTTTCACTTTATTAATTTTTGGAATGTTATAAGGTATCCGATTTCGCCAAGGGTTTGTGCTTGTTCCCAATGTGGAGAAGTTTTTCCAAAGCTTGAAAGCTTAGAGCTTCATCAAGCGGTTCGTCACGCAGGTAATGCTTCTTTTTTTTGTTCTTTAGTTGGGACTTTATCACTTAGCTAAAGTAGTAGTATTTAACTAGGCTCTAGCTACACTATTTACTTTGGTATGTGTATAGTCACGTTACTAAAGGGTACTAAAGTTAACCCTAGTAATAAGAAGACCTTTTAATTCCTGTGGTGACATTTTACTAATGCAATTTGCTTCGTAAAGCAACGATTATATTTTGTAGAACTAAAAATTTGTTTAAGATCTCAGTCCATAGTGATGAATGATGATTACACATGTTTTTGGTGAACAAGCACTAGGATTTATTATAAGATTTACCATAATGTTAAGCAATAATTAACGAATATGTCTTCATGTTATAGCCTAGACTCAGTTTTCTTAAAAGGAATCAAGATCTCAATGCTATTGTCGGTGATAACATCTATCAAATAATATTATGAACATTTGGCATTATGTTACAGTTCGTAACTATATTAAAATTCTCATAACTGCATCTCAAAGAAAAATACAAAATAGAAGAGGAATATTAATGCAGAAACAATATATTCAGTAGTTTCCATACCTGTCATTTTTGCTTGGAAACATTTCGGTGCGAATGGGACCATTTGTACAACAGCTGTCTCAGTTCTTAGCTAAATATTTTTGACACATGATACTATTACGTGTCATATTTCTACTAGTTAGTACAAGAATTAAAACCTTCACCTGACAAGCCGTACTAAAACTTTTGACTTTTTTTTTAATTATCTTCTCTGATTTAAACGTTGTCGTTTGATTGCAGTTTCAGAGTTAGGTCCGGAAGATTCGGGTCGAAACATAGTGGAGATCATATTCAAGTCAAGCTGGCTTAAAAAGGACAGTCCAATCTGTAAGATCGAACGGATACTAAAAGTACACAACACTCAACGCACGATCCAGCGGTTCGAAGATTGCCGAGACGCAGTGAAGGCGCGTGCGCTTCAAACCACAAGGAAAGACGCTCGTTGTGCTGCCGACGGCAACGAGCTTCTCCGCTTCCACTGCACCACTCTCACTTGCTCGCTCGGATCTCGTGGCTCGTCCTCTCTCTGCTCTAATCTCCCCAGCTGTGGCGTCTGCAACGTCATTCGCTACGGGTTCCAAGGAAAGTCCGGTGCCGGAGGAGTCGCCGCCACCGCAAACTCCAGCGGCGGTGTGAGGACGACGGCAAGCAGCGGAAGAGCTGATGATTTGCTGAGGTGTAGCGATGATGCGAGGAGAGTGATGCTTGTGTGTCGTGTGATCGCTGGGAGAGTTAAGCGCGTCGATTTACCGGCGGAGGAGAAGAAGTCTTCAGTGGAGGAGAAGAAGTGTCCGGTGGAAGATAACTCGACGGTTGGAGTTTCTTCGAGCGGCGGTGTGTTTGACTCTGTGGCGGTCAACGCCGGAGTTTATTCTAACTTGGAGGAGCTGGTGGTTTATAACCCAAGAGCTATTTTACCTTGCTTCGTGGTTATTTACAAAGTTTTAGAATCTTGAGTTTGTAATATTTAACTTCAGAAATAATGTTTTTTTTTTCTTTCTTTTTGTTTCTGTAGTGTGTGCTTCTAACTTTGTAATATTGCAGGGAGACTAGGTTAATCATCATTTCGGATTTTAAACCTGCATCATAAATCCCATCTTATTATTGACTAATAACTGTCGAGTGTAACATAGTGAAACTTTAGTTTTATTAAAGTAATAATTCAAAACACTGTTAGCGTAAATAAGATATGTACTGTTTTTTTTAACCACAGTAACTTTTCATAGCTGCGTCCACACTGGGTGGGCATCAAATACTGTGGGAAGTGGCAGAGAGTGATATAATTGCTACCACATGCGTACTCAGTATATATTGTTTTATTCTCCCAAGTTCTTTTGCTTGTTTTTAGATTTTAGGGAAAGCTGATTTGTTTTAGATAACCATGCAAAGCTGTTTTTTCACTTAAAGAAATACTGTATTACTCAGATAAGTTCAAAATTAGCCCATAAATAGTAAAGGGTTTGCACGCTGAGAGAGGCAGCCATTGGAACTTTCCTCGTTGTCAGAAACTGCTAGCCTACGTTTGTTTATCTGATGATAAGTAAGTAAATAACTCCTTTGCTCCTTGAAGTTGCAATCAGCGATATATGGCAGCCCAAACTACCATTATGTACTGGTATTAAACTAAACTCCAGCCCAATTAACTTTGACTCCCAGTTGGTAGCACTTAAGACACGTCCTTGACGTTGTTGTGACATATTATTTACACGAATTATATTTCCAAACTCAAAGGTGATGGCCCAATTGAATTAGAAAGTAACCAAAAATTCTTTGAAAATGACAAACTTTTGGACGATATATCAAATAGAATATTGAGTTATTATGAGTTTTTTTGGGGGGTAAAATGTTAAAGAGTTATGTATGTGGTTTAGATTTAAGTAACCTTCCGAAAAAATTAAAGTACAAGACGTAATCATAACCGTCATAAGACCTTTTGTGATCAATGGCGAACGCAGAAAGTAGACGCTAAGTAGATACTTGAACGTGGGAATACAACAAAGTTTGGGGTTGAGAAGTATATTCTTAGGAACACTTTATTGTGTTGTTTTCTTAGATGATGTTGATGCAATCATGCATAAGATCTTGCAAAGCAAGAATATGAATCAGTAATGGCTTGCTTTATGCTCCTCTTTGTAAAACGCTGCATTTGGGAGGGGGAAAGCATGATGTTTAAAAATTTGGAAGGGAGGACATTCTTTGTAAGAGAAACAAGAAGAATATAACCTCTCCTGAGCATTTGCATGTAACATGAGAGCTCCTGAGTTTCATTTAACCACTTTATTGCCAGTTAAGAGTTAATTGTGTATCGCAAATCAGGAGAAAAGAGTCTTTTACGTTATGTACTCTAAAGATGATCTGGACATGATATGGTAAACACACTGGCTACATTTTTTGTATTTTTTTTCAGTTGAATTTGTGCATTTTTAAGTAACTGATTCTGTTTTTTTCTTCTTCATTATATACAAGTATTAGAAACAAATGTCAAATTGGCCTGAGCTTCCTGTAATTTGCTTTGCAGCTAAGTAAAATATAATATTTTTATTACATGATAAAATTCAAAAGGGTTTTTCAAAACTTAAATAAAATTTCTGTGAAATTAATATAACTATTTTGAGTTAATTATGTTATGAGTTATCGAGGTGGTTTAGTTTTAAGTTTAAGGAAGAACTAAAGTACAAGACATAATCATAACCCTCATAAAACCTTAAATTTATAATAAAGATCCTAATCGAACAATCATTTCAACAAAAGCCTCCTAATTCATCTATGTTCTCTCCATGTCTCCTCCTCTCTAATTTATTTCCTCATTATCTGCCTTTTTGTATGTCAAACTCTTGTTTTGTTCTACGAATAATGTTTTTGACGCTTAATGTTTGCTTACTGGTCCCAAAATTAGTTGTGCCCTAAAACTAGGTCAAAAGTTTTAATGCAGGACCACAAAACTCTGCCTGAATGTAATCTCTAATTACAGCTTTAATTATGCATTAATTATAGCGTGAAATGAACCTATGATTAATCATACGTACATAGATCAATTCCTAGTTCGTCTTTTTTCTTTATATACGTTGAAAGTTTTAAATCTAAAGAAAACTTCAGTCAAGATCATGGTTCCTTGTGTTATCTTAATGTTATATTAACATTTATATATAGCAAAAGATATTGATCTTGTTTTCAAATGTTGCAGATGTTATACTTAGATAGAAATTTCATGTTTGACATCTCCTGAAACCTCTAGTATTTTTAAATACACATCGAGACAATTAAATTGGATTAAGAGCCTTGATAGTTAAAATGGACCCTACATATGTTCAAAGGATAAACACATCTCAAGAAATTCTATGGTGGTTTAATCATTGAAACACTCTCTTCTCCCATATTTTCCTCTCTGATGAATCATTACAAATTTACTTGGGACTTATTTTATAAGAAAAAAATGAATGCTATCTTGATTAAACTATCAAAAACATCAACGTACGAACTATAGCTTTCACCATTCATATAGACAAACCCACAAGATCCTGCAAGATCGGTTTTTATAAACAAGGATCATCATCATGAAGCTCTTCCACAAGTTCCGAAAGATTCTCATGAGGCTCATCGATTTCACTCTTCCTTGCTCCTCCCACCGACAAAAGAAATCAGGCGAGGGAGAGAGGTTTGAGCCGCCGAAGATATCTTGCAGTAATTCGTACTATTCTTCTCACGTACATTACAATGAAGCTATCGCAGATTGCATAGAGTTCTTCAACAAGTCTTCGACTATGTCTTGTGATGCGGATCATGAAGGTCGTCATGTAGACCAGCGTGACTGTTTTTACGTTTAATTACATCTCAAAAGCTCACATGTTTCTTTTTGTTTTGCTATAACATCTGATTCGAGGCTTTGCTTTTGTTTTGCTTATTTGAGTTGTTGTGATTTTGAAACACTTATATTATATGGTCAATTATAACTATCGTCATTTTTAGGACTAGTTTTCTTAAAACTTTCTAGCATTTTCATAGTTTTCAAACTATCATACTTTGAAAAGAAATGGATGTAACTATGTGAACATTTCTTACGAGTCTAGTTTTCTTAGATTTTCTAGCATTTCCATCTAGGTTGAAATATTCATATTGGGGTTTGGATATCAGAATCTCGAGTTTTCAAATTGTAATTTCAATCATGATTAACCTGTGGACGTTTTAAATGGTAAGGTTACACGAATATTTACATATATTATGGATTAGTTAAAATTGTTTTTGTTTTATATATTTGATAGCTCCGGTAAGAACATATTTATTGTTACAGTGTGGACTTCTATTCGAAGGATCACTACACTGCGTGAACCGAATTTGTATACTTTCTAACTAATGTTATAGAAGTAACATGATCGTATGTTGCCAGATTATCAAGTAAAACTGCATTGGCCGAAGCTAAAGTCCACACTATTTACATAAATGTATAGTTTTTGTTTAAAAGAAGGTTGGAGGAACATGGAAAATGTTTAGCACATTTCTAGATTTTGCATATTACTTGATCAAAACCATGCGGACTTGGTTTTAATAATATATGTGCGTTCCAATATTAAGTTTTATTTATTTGAAGAAGGAAATTCAAGAACCATTTACGAAGGTCATACATATGACATTATTATCATAACCACAACAAAACACACGAGAGAAAAGAGAAGAAAAATTTATACACCAAGCTTGTTTCATTGCAAAACCTTTTTCTTTGAGAAGATTCCTCCAATGAACAAAGGGATCAAACTCTTCTTCTTGGTCGTCTTCGTCATCTTCTCCTTCTTTCTCATCGAAGGGTGTCTCTCAAGAAACATATCCACCACCGGACCATTATTATCACCCGTCTTAGGGTTTACAAATTTCACGTATCTCTTCTCATTCATCGTTCTCAACCTTGGACTAGTTCCAACTGGTCCATCATCAGCATGTGGAACCAACACTTCGAACTTGGTTTTGAAAACAGTATCATCGACTTTAGTTAAGGGTTCCTCGGGAACTACTCTCAGCTTCTGAAGCTCTTGTCTTGTCCTTTCAAGTTCTTCCTTGAGAGAAGACAGAGAGTTTCTCATTTGCATGCTTTCTTCTTTTGCTCTTTCGAGATTGTGTTTTGTCTCTTCAAGCTCTGTCTCAATCTTTGCTGGATCCTCAAATTTCTCACCCTCCATCTGTTTTCATCATCAATGGTATTGCCATAATCTCGAGATCATATGAATAAAAGTTGAAACTCAAATAGATTATATTGAGAGAGAATAGGACTTGCCACTTTGAGATGGTTGGAATAGACTTGACTGGCTAAGACTCTCTCACCAAACAAAGCTACAGCTTCTTTGACGGTTCTAAACGGTGCCCTTGTATCGATCTCTGCTCTCTTCATAGTCTCCATCATATGGTTGCTGCTTAGTTCTGTGAAGAAGTAGAGAGATACATAAGCTTTGCTTATAGCATGGTGGCCTTAGAATTGAGTAAAAGACAGACGCAAAGCATATTAGTTTCTTCATCTCCACTTCTTCAGAATACTCTTCTTTTCAAACACTTTTTATATATTTATGGACTATCTATTTTATATATAAGATTGTTCATTAAAAACAAAGGATTTGTACTAGTTTTTTTGGTAAACTTGTACTAGTTTATTTGATATATCACATCACACCCAGATTCAAATAAGAGTTTACGTGTGTGAGTTATAGATTAAACAGGCTGACAAAAAAAAAGATTAAACAGAAAATACCATAAAATATCATTATAACATCTTTTTATTCCAAAATAGCACAAAGAAAAAAATCTCAGAATAACATTAATTAAAAGAAAAATATATTTTTTTGTTTTATATTTGAGTTTAGATTTATTGATTTGGGTTTAATATTTCAGGGGCAGGGTTCTGAACTTAGCATAAAAGTTTAAAGTTTGGAGTAATTAGTCATTTACCTTAAATTAATGCTATTTTGAAGAATTTTTTCTTTGTATGTTATTTTTAGCATATTTTTTTTGTTATTTAAGGAATTTGCTGAATAAAAATACATGCTACAAGTTTTACAGAGGATTTGTTGGATTTTTATTAGCTTGATGCACAAGAAACTAAAAAGATGCTTTTAAAAGATCTACTTGGAACTTTACCCTTTTTCAGAACACGTTGCTCGTTTACATAAAGAGACATTAAAGAGTTTTCTCAAACAAGTAGGGTTCAGTCATCTCTCTCTCTCTCTCTGACAATCCTAGTTCATCCTATTTTCATCACCTGAGGACAGAAACAGAACTCTGTTTTTAACTATAGAGCAGAACATCTTCACAACTGCTCACTGCAGAGTAGTTGCAATGGTGTTCTTGTGTCTTCGTAGATTAGTGGAAGATGGTGTTCTCGTCTGTTCATCACTGTGCGCAAATGGTTATTGTTATGGTGTTGACTCCTCGACATTGCTGCAGCAGCAGCCATGACACGGTGTGGCTGTTGGGATACATTGTTGATGTACCTTGGGGAGGAAGCAATGTGTAATGGTTGAATACTAGTCTGGTTTAGGCAGAGGTTTGGTTTACTGTATTGGTTCGATAATGGCGGCACAGTGTTTCTACTTCTTGGTGGTCTGTACTGAGCAGCCCACAGCCTTTTCTGAAGCTGTTGCTGCTGCTGCTGATGGTTTGCGTTGTAAGCGGTTTGGAATCGACTAGCTAGATAAGGAGACATTTGCTGCATCTATTAAGATGAAAAGTCACACTATTATGCAACAAAATACACTACTCACAATCAAAACTTTGATTACAAGAGATTTAGGTCACCTGTTGTTGGTTATTGTGAGATGCAGAGATAGGATACTGTGGAAACAAGTTTGGCAATGGGAGATGGGATTGTGCTTCTCCAGAGCATGAAAGAGACAGGAAGTCGCAGTTCTGTTCATTTGAAGAGACTAGAATCAGAGAGGCAAACAATAAACTAAAGACAAGAACAGTTATGTTTGTTACCTGTTTCTGTGCAATGTGTGGTGCATAAAGCTCAAGAGAAAGCTGAGTCATGTTTTCTTGCACCAGTTTTGAACTTTGATAGCTCTTGGATGGAGCAACCATGGTATTAAAATCACTAATGATTGTCACAGGATCAGGGAGTCTGCATTGTGACATTTCTAAAGATCTTTCTTCGGTCTGGTTAAGAAGTGCCACGGGACTACTTTTGCTGCTCTGTAACACTTTGATGAGACGACAAATGTAAAAGTGAGAGGCACATCTCTTCAAATTTCTCGGACGGACTCTTAACGGTTCACTCAAGAGTGAAGCATTCTTGGTAGGAGAAACAGCTTGACCTATCATCCAATGTGGAAGCTTTGTAGAAGAGAGTAAGTTTAACAGCGTTGGCTTTGTGCTTACAGGTTCATTCACTGAGCTGCTTTGCTTCAATCTATATCAAAAAAGTAATTAGAACACTAAATCAAAAAAGAAAAGTTATTAAAGGAGAAGTCAGGTACAAACCCGGTTAAGCTGCACTGGTTATGATGTTGCTGTAACGGCTCCTCATCAATCTGCTTAGGTTTGGATGAAGAGAGAAATGAAGTTGTAGGTTCAAGTGATTCAGCTCTGGTTGTACATTCAGTTTCCACAACTAACGTTGAGTCAACTTTGCTGGTTTCTTTGCCATCAGACAAGAGAGGATCACAAGTGTTCTTATCAATGGAGTTAGTGTCAGTAAACATATCAGCTAAACCGTATAGCGTCTCTGCAACTTCTTTCTCATCTTTTGAAATGGACTCGGCAGCTTTTGCATCCTAACCAATACAGAATTTCATGAACTCATTATACTAGACTATAAAATTTTACACAATAATTTAAGAAAAAATTGTAGTAATCAGTAAAATAAAAAAACAATCACTACTTGATTCTTCTTTTTAACCAAATCAATGTTAAAAGATTCAATTCCGGTTCTGGACCGGTTGAGTTTCTTGGAAGCAGAAACAGATTCAACATTCCTCTTCTTCATGGCTGCAACCCAAAGTTCACAAAAAGAAATAATAGTGAAAAAAAAAATTACTAAAAATATAGATTTGGTGGAGTAAAAGTATGTCACTGAGACAAAAACAAGGCACCTGAACGAAGCTTCCTTGGTACAGAGGCATGATGATCCACACCATGAGTGACCTGAAGTTAAGAAAGAAAGAAAGATTAATATGAAGGGACTTGTGTGTTGTGAGTTTACGTATAGATGAAGAAGTTACATTTTTGGGAAGCCTAGACTTCTTCTTCTTCTTGAAGACGTTGTTTTCTAAACCTGAAGCAGGAAATTCGTTAGAAATCATCTTCTCTTTTTTCTCATGATTCATATTGTCATCAACATCAGCTAAACCCAGTTTCTTCAACATTCCTTTCTTCAATTTTCCTAATTCCATGAGAATATAGAAAGTAAATGAAAAAACAGAGCAAATGACGACAATAAGAGAGAATTTTGGATTTTTTTTAGAAAAAAAGAAAAAAAGATTTGTAAATTTACGAGATAGTTTATTGGAAAATCGATTGGTTACTCCTCCACGTCTCACATCTCGACGACTCATTTCCATTTTTTTTTCTATTCACTCTGTCTCCTTCACCACTCGAAAAAGAAAAAAAAAAGAAAAATCTCAGTTCCACATCTGCAAAACAAAACAAAAGTATCACAGCACTTTAGTTACTTACACAAGAACAAAAGAAAAAGGGAGAAAAGTAAAAAAAAAGAAAGATGTTAAAAGAGAGAAAAGTTATAAAGATCGTTTCCTCGTGTCTCTCGAAGAATCAGCTTCTTCTTTATAAAAGAAAGACAAGAAACCAAAAAGACATTATCATTTGTTTTTATGTAATAAAAGGAGATACTAAAGGAGGATTTGTAACATTCAACCCGTGATATATACCTCGCTCACATCATTAAATTTTTGAAAAAAAAAACAAGGAAATTTTCAATTTTCATATAGTGAGAAAAAAAACTTCATCAATAATTATAGTAGTCAGTGTTCTCCAATCTAATCAAGCATTAATTTGTAACTGACACAGAAGACGAAAAAAGGTTTAATAATGGTACTCTTTATCTGTCTCACCAGGTTTAACACAGAACAAGTTTATCGCAAACACACAGAAGCTATAGCTATTCTACGCTTAGATTCATCGGTATACTATAAACAAACGTAAGAGATCAAATGAAAATTAAACCAAGCTCTCTCCTCATTTAAACTAAAATATTTTTATCAAAGTTTGCACATTCTGAAAAGAAAAGACGGATAACCCGAAGCTGTCGCTTCGCTCTCGATCGTATTTTATAATTGTCACACACACGAAATGACTGAACGCTTAATTCTATCTATCCTTCACTCTCACAAACCAATGGTGTGTGATAACGAAGATACTATAACCGTTCGATTGTTTTAAGTGTTCCGCCACGTAATTGCTGAGCAGATCATGTGTCGGACAAAGTCAGCATTTAAGAGCTGAAAATGTACTGTTCCTGCATGGGTTTCCTATGAGATATGATCTGGATCCGTACATCAGGTACTTGAGTTATATTGGTTAACTTAGAATTACTATTGATGATGGAGGTTGTAAGTACTTGCAACCATTTATAACCAAAATGTTACATTACCCCCCAAAAGCTATGATAACTTCGTATAACGGGTCTCCTCCGTAACTCAAACGTCTGGCCTGTCTTCCATAAATACGAGAACTCAAGAAGAAAGAATGCCGCGGCGTTTTTTTCTCTCTCTTATCTTAACTAGAGGTTTTGCCCGGACTACGCCCGGGTTTATAGGGTTTATTCAGAGAACTTGAAATAAATGAAGAACATGAAATGAATGAAGAACTTGTTAAACCAAATTGTGGTCTAGTTCTAGTATTACCTAATATTTGAAGTATTTGGCTCTTAAGTACCATTCATGTATTTTTGAGAATTATATTAGATAAGTTTGCTTTTTAGTAGGATTCAGATTTCTGGCTAAGTTATAAGTATTTAGGCCGAATACGTTGCTGATGCATTGTGTAGTTTTGTGTACGGGATTTTGTCGAATGATTTATTTATTTATTTATTTTTCCGTGTTGTGTATAATGGTGATGAATGGAATATTGGTAGGCACACAACTTGATTAGGTTGATATAAGAATGACTGGTGAATTCATGTGTTGATATTTTCATATCTTATCTTCGTAATGATTTGATAGGCCATTAGCGTTATTAGCGATTTCGTGTTCAAAAGGTGAGGTTTATGGAGTTGGTTAGTGTTAACTTAGGTAGTTATTAGTATATAAATAATAAAAAGTGTAAGAATGATATAAAATTTGGTTTTAAAAATTAATAATTTATATGAATAATAATTTAACTATTTTTTATTTTACCCAATCTTAATTTCAAAAATATAGAATCGTAATAAACTATATTTTTTAAGAGAATTTAAAACTCTTGAAATTTATAAAATCATAAGGTTACAAAATTATTTATTTTTAAAGTTTTTACGGGACTTTGTTTAAAATAATTTGTGGTGGACATAAAAAGTCTGAAATATGTCAACTACACCTGAGGCAGACGAAGCTGGTGTAAACAAATATCTCATCAATCACACTGAAAACAATCTTCGTTTTTCGATCTTGCAAACTAAAAATGCTAAATACAGAGGAAATTGTGTATACTTTTCTGACAACGAAAAAACATGAATTGACGACCTAACATTTTACTCAGAATCAGACAAAATTTCAGATTCAAAGGTCAGATCTTTGAAGTCCAAACATTTTAAAACCCAATTTTCACTTAATCATTATATTGGGCCATACAACTATACCAAATTCTATTTTTTGGTTGACCTGTTTTAGGCTTTTTTTTTGTCAATCCAAATCATTTTTAACAAAGTCCAGTAAAACCTCTAAAAATAAAAACTTTTGTAACTATATACTTTTATAGATTTCTAGAGTTTTTATTTTTTTTAAATACCATTTATTAAAATTTTATCTTTTTGAAATTAATTTTGGGTAAAATAAGAAAAGTAATTTAATTTATTGTTCGTGTAAATTGTTAAATTTTTAAAACCGAATTTCATATATTTTCCTTACACTATTTGATTTATAAACTAACAACTTCCTATGTAACACACTATCTAATATCATAAACCTCGGCTTTTGAACTTGTAAGCGCTAATAATGGTTGATCAAATGATTACAAAGATAAGATATGAAAATATCAACATATATGAATTCCTGGTCATTCTTATATCAACCTAATCAAGCTGTGTGACTTTCAATATTTCATTCATCACAATTATAAACTCCCTGAAAAAAAACAACGACAAAATCATGTACACAACGTATCAGCAACGAACTTGCCCCCGGGGGAATTTCTTACTGCAAACACCATTCTCCATGCAAGGAGATGCTAATCTGTCTTTCCCACAGGGACTCATCATGTGTTACTCTCATCATTTATTTACTCGTACCAGTTTTGGTTACAGTGCTGGAATTAAATTATATCCGGTTATCCAGTTTATTCATCTTAACTATACTGATTTATCCAAATACAAACCAGAAAGAAAATTAAACCAATGAATACCAAGAAGGTACTCCGTATCCATTTACTTTTTTCACAAAGAACTCCTCTTTACTTAGTGGAGATTTCGTTTAACTCAATATTATTAGTCGCTATGCTCTTATTATGCTATGGTAGCCTGAGCCTGCACGAAGATAATTTCCTATCCCAAGATGGTTAATTGGAACAGAAGAATGCTCTGGATCATGGGCCTAGAGACACATGCTTTCTCTTTTCTTCTTTTTTTTCTTCGCTGTGAGAGATGTGTATATGAATCAGCCCCTACTGTTAGCAAGAAGTTTCACTCCCCCCCAACATCACAAATCAAGTGTGGAGAGCCGCGACCTTTTGAGACAAAGCATCAGCTGCATCATTGTTCGGTTCCCCGTGCTACAAACAATCAAGAATGCGAAGGAATAAGTTGTATAAGTACAAACTTATAATATATACATCTTTTAAATGCGATTCTTGAATTTTTTTTTACCTCGGACTGGTTTCTTTGTTTCACGCCTATGCATTTAGATGCACTGATGCTGTTACTTTTCAGCAAGCTTCGGGAACTGTAACCCGTCTGGCTCATTCCGTTCATGTAATTTGAGCCTTCCTGTTGAGAATTTTGAGGGCTTTCGGGTGTCGCGGCTAAAGAAGCTGCAGGTCTGTTCTCTTCCTTAGCAGGCACCCTCTCTCTGAAACAGAAAGGTACCAAACACAAAGAAACAGACGTGAAGATGCAAATTTAATAAACAAAACAGCAAAAGAGATAGTGTACTACCTAGGCAAAGAAGCATGCCGTTGTTGAAAAGGAGCGCTTCTCTCACCCTTACTGTAAAGTTCTTCAAGATGTGCAAACTGTCTCTTAAACCGATCAACACCACTGCATTTTCATTTTTGATTTTTAAAGAAGATATTTATATATCTTATAGTAACTCGTATAGAAAATTTTGAATATAGATTGTAAACTGTTATAATATAAGTACCTAGGGTACATGAAACTCGTCTGTTCTCCACCTCGAAGATATTCTTGCAGCATCTGAGGATGGTACTCTAAAATCTGCATTGTTTCACATGCAAGTTTAGTAAGAACAACAATTAAAACGATGAATACATGTGGAAGAAAAAAAGAACAGTACTGAGTCTATGAGATTTAATGTACCTCTCTATAGATCAATTCCCTGACGTCTTCTTTCATTATCTTCCTTCTTTCAAACTCAAACTCAAGTTTTGGAATCGTTTGGGTAGATGGTTCCCGATCCACGTTTGCCAGTCCATAAAAGTAAGGATCTGCTAGTGCCTGTTTATACTCACACAAACACACATTTCACACATGTTCAATGGTCTGATACTTTGAAGAAGTGCAAAAAATAGTATTAGAGGAGCTTTACCTCTTCAGTTGTGGCCTGTGGGACGGTCTTTGGGATCAAATGCAAGAAGGCGGGGCAGCAAACGAAGAGCCAACGGGTCTACATGAGGAAATTTGTGAGTGAATGGAACTGGCGGTTTTCTCCTCATGTAGCCGAGATATCTCCTCCTCTTTTCATTACGAATCTGCAACAGAAGTACAAAAACATATCAAAAGATTGATCACTATTACAGATAAGAGCAAACTGATGTCTCGTATGTGTACCACCAACCACAAACTGACATACATATGACCTGAGGTCCCCTACCTATTAATATGATTCTAACCCGAGCTAGGCCTAGACTATTTGCCATAAAACTGTGGCTGGAAAGCACAGGTAAATAAATATTCATAGCTTACGGAGAAACTTTAACACATAAATAGTAAGACATCAAAGAAACTCCACGCTAAGACATTGTAGCAAACAAATTAAAAAAAAAATGATAAAACTTTAAACTGATTAATACCAAATGACCCTTGAGATAGCTTCCGGAGAAGGAGTACCAAGCAAATCAGCCATAATATCCAATTGGTGCACCACGTTCTTCCCAGGAAACAATGGCTTCCCGGTGAGCATTTCTGCAAATATGCACCCTATGCTCCATATATGAATGGCAGGAGTATACTGGAGGAGAGAAAGAACAACATAAGAAAAAAAAATCAATCTTCAGATTGTGATGTCATAATAAGCGAAACAGGTAAAAAATCAGATTTTATTTTGGAGAAGAAAGAGCCACAAAGTTCTGGTGCACGGTACCATCTTGTAGCGACATAGTCCTGCCAAGAAACGAAAACATTTTTCGTTTTCGCCAAAAGAAACTGAAACAAATGATTATAAGAAGCTAATATCGGTTGACAAAAAAAAATCAGAAGCTAATATCTCCTATCTCCGATGCATCCACAGATTTTAGAAGCACTACTTTTTCGGGAAGTATACAAGAAGTCAAGGACTTCAGAGCCTTGTCTGAGTTTTTGGTTCCATAGATTCCTGGACTAAAATAGAACAACCTATCTTCTTCCACAGATGTCAGCATTCTGGATACCAGCTTAAAGTGATCAGCTTCGATGGGTTTATGGTGATTACCTTCCCCCATTGCTAATATGTTCTCATAGAAAAAAAATATGAAGCCGAGTAGCTCTGCTAGCTTTATGGCTAGGACACTGAATAAGAGTTGCCTTTCTGATGAGTACCAGATGGAAGATGAAAAGCTGCCCCATGGCTTCTGAAACATAGGCCGTTAACAAAACCAACATAACCTGTAAGAACACAAAAAAATTACAAACTTCATAAGGCAAGAGAAATTGCATTAGATTTCATGAGAAATAAGCAAGTGTGAAGAGCATACAGATGTTGGAGCAAGGGATCATCTAGGGAACACTACATGAAGCCTTCTCTTGAGTTCGGTTTCCATCCCTTTCGTTCCCAAAAAACATCTGGAAAATATGGCAATGGATGGATGTTCCACCTTCTATAATTAGCTGTTGCAACAAAAGGCAATCAATGTTGTTACGTGAATGAGTTATGGGTTTTTGTTTTAAGTAGTTGAGGAGATAATACAAACCATGAGCAAGACAAAACCCCATTAGAAGTACGAAACTGGTATCAAGTTTCTGATACTTATCAAGTTCCTTTTCTTCACACAGTTATCGAGCCACTGATTACACCAAATATCCAAAGAGAAAGCTTATCGATAAACAAACAAACAAAACAACGGAGTTTCAGATTCAGGAGTTAAACCACCAAACTTTACCCTGCAACTGTGAGCAAACCTCCAACACCCCCATTTACATGGCCTGCAATGCTACTACTGCGTTAAACTTAAATAAAACACAAAGAAACCGTTCAAAATTTCATAACAAATGTGGTTATGAGAAAAGGCAAACCTCTAAATCACAAAGTGAGCAATAAAAGATATCATCTTCCTCCTCATTAGGGTCTGGTGTAACAGCATCATCTTTCATGGTGAATAGTCCCACAAACACACACCAGTCACTGCATTATAGGAAATAGATGGAAACAATTACTAATATATCCATGCCGCTAACGTAATTGAATTAGGAATAGATGAATAGCATATCAAAGTATGTCTTCCAGTCTATCTAACAAAACCAATCGAGAGATACAGAAATAACTTCTGAAGCATTTGTTACATGTTTTGTTTCCAGGACAAAAGGGAGCATACCTGGTAATCAGAAAGACCCGTTCGTTAAGTTTTTCAGACACATTACCACTGCAGAGAAAGGCGGTTCGGTGGATGTGTTTCATATGATTATCAAGACGATACTGAAGCAGCGGTGTTGGGATTCCAGCTCGCCAATTCTGCTCATTCACCGTATTTGTTATAGTTTCGTACGGATCTTAAGGTCAAACATGGAATCTGAACAGTAGGTAAGATTCAAATTCAAAACATCAATATATCGGATGCCGAGATATCATTGAGATTCGTCTTCAATAAATCGATATTTGATTGCTTCTCTGCAACTTCCGATTCAAGCTTCTTTCTTACATATATATTTTTTTCCGGTGAATTTTGCGAGGGGTAGAGATTGGTGATGAAAGGGGTTTCTATAACGTGAGAAATAAAAGGAGTTAGAACGATAACGTGTAGGAGTAAAAAACAGAACTGATGATCGAGAGTGGGTGATAAATTCAGATATACAAGGATGTGAAACGGTGCCGACGAAGTGGAAGAGCCAGAGTTGATCTTCGCTTCCGTCGAAGATTATTAGATATGGAGAGGTGTGACGTAGCAAAAAGAAACCATTTTATTGGCTGAATTAACATGCCGACGTGGATGGCCTAAGAGGGGTTTATTAGCGCCTTTTAATATAGTAGTGATGACTCGTCCAATCAAGGCAGCGCTAACTAGAAGTGTGTCTGAAGAGGTAGAGAGACCCCTCCTAGCTCAAACTCTAGTCTCTAGAGAGAGAGAGATTTTTGTCGATGTTTAAATCACCGGCTATCATTATTTCTCAATGTTCGGTCATTTCATTTCATTCACACTGAAATCGACAAGTCAAGTGGTTTCTGAACTGATAATATATAAACAATTGTAAATGAATACAATTTCACTACATATTTGGGGCGAAACATGCTCTGGAAAAAGACCCATTGTTACGATTTGTGTCAAGGCCCATAAAATGTGCAGTTGTAAGTTGTGCAAATAAAGATTTTAGAAAAGACATGTAACTTGAGGCAACGCCCATGTGACATTGTGACCTACGTTATCACCTTGATAGTTAATTTTTTTTTCTCCGTCAAGGATGTTACGATTAAAAATTAGAGGCATTAAAACTTATCAATTTTCCCTAGAATGGGAAAATTGTTTTTTTAGACCAAAAAAATACATACATTGTCCTAATAGGATAAAATCTATTTTGTACCATATTTTCCTTTAATACCCTTTTTATTTATCAAAATAGATCAAAATAAATAGTTTTACAAGCTAAAAAAATTATGAAAAATACTAACAACTAAACAAATACCTAGATCAATGTAATGAAATTTTTTTAGGATTCTAAAACTTGTTAAATAAAAAATTAAGATTCAGTTCTACGCATAGTAGATTGTATGTTCTACAGAAAATGATTTAGTGGAAACTGATATCTACGTTTATTAAACAATCTACTTATATTAGAATATGACATCTACCGATATTACTGTTATCTAAATATATTTAGAATCTATATTCTACTCTACATCTTTCAATCTAAATTCCGTAGAATATAAAAATCTACAAATAACTCATAGTTCTAATATTAGTAGAAACGGATTTCAAACTGGTTGTCTTCGTTCTACACATAGTAGATCACAAGTTCTACAGATAATCTAACAAATCTTCGAAAATATGGGAAGTACATTTATGAGAATATTTGCATATCTTTATATTTCCATAACTGAAATCCACGAATTTGTTAACCAAACAGGCAAAGAGTTTTAAAAAAAAAACATCTTTACCTACCGAACTCGCGACATTGTCAACCTTTTTCCTTCACACTAACCGGTAGACTGAAATGGGCTTTACGTATTTACCAAACATTAACTTATATATTTTAACACATACAGTAATAATTAAGTAATGAGCGAAAAGAATATAAAAATTGAATTTTGATAAAAGCCAAAAAATATTGCATACGCTGCTCATTTGTGTTGACGTCACTAGTGACCTGTCGTTTAAAAGATCTTGCAGAGGCGGATGTGGTGAGGAGACAGAACAAGAGAAGAGCAACAGACATGGTGTTTGTGTAATGACGCTTCATCTTGTATGTTTTTTTGGATGAAGTGAGCAAAGCAGAGATTAGTTAGAATTAAAGAGCGTCAAGACTGAGAAGTTTTACAAAAGGAAGTTACAGAGAACCAAACATCTTTCAAATCTCTTTTACAACCTTATTAAGAGAGCTTTAAACTAATAGAACGAGGATCACAACAATGGGTCTATGTTGGAATTCATGAGTGTTTGTCTCATGTCAAGAGAGTATTCAGTTAACT

scaffold 2

TGAACTAACTCCTTTCCTCTGGTTATTCTCTTCTTCATTTCAACAGTCTAAAGGAATGGAAGTAAATAAACAATACACACTGATCGCATTAAGAGACACAACCCTTTTAGGTCAATTTTCGGGGTCTACGGATCAAAAGACATTCGAGTCAGTTTCAAAAGTATCAAGGAAGACATTAGACTACGTTTCTCTCTCGTGAAAGATGATTTCACAGCAAAGCATGCAAACTTCAGCGACTCGTATTAGGCTTAGTAGACATCTTGGTGTTGTGTTCTGTTTTGTCAACATCCCTGCAAAACTCTGAACAAGGACGACATGCAAAGACTCCAATCTCTAAGATTTTGTTACCTTGTCACATATTTGGCAGTTTATAATTTTATGTTCTATAAAAAACCAAGCATAGAACATAGGTGAAAACCTAAGAAAGTTGACAATTGTACAAAAAAGAACACAGACTCACCAACGATGCTTTACGTGGATCCAGGACTAGAACTTTCAAGGCATCCTTTACACATATCTTTTCACTCTGCACCCAACCTTAACTGTTATACTTTCCTTTAAGGTCTAACCTACAAAGGAAGAAACATCAACACATGATTGTAGGTAGAAACATCAACACATGTCACATACAAAAAAAAACTTAGAAATTGCAAAAGTGATATACTTTCTAAGACCCAATACGCTGATAAACCAAAAGCCAAACATAAATGCAAGTAAAGCTTTACAAGACTGCACGCTCACTCTCCATGATCGTTGATGGCACAGAGACTTCTAACACAAAGCTATCGAATTCATGACCTTCCTGCTTCACCATATCGTTTGTTGGCATCGGAGAAAACGAACTTCAATATACCTAAACAGACAATGAAGACTTTGGAACAGCTTGACTCCAGTGAAGGAGTATTCTCATTGTCGGATTCTAGCCATCTCCGTAGAGTTGGCGTCGAGACTGATGACAAATCGGAATAAAAACCTCGACGGATACCAATTGGAAAATACATATGACAGACCCCAAGAGATAGCAGATCTTTGACAGCACTAGAATGTAGTGATCGAGCTGCTTGGTACACGCGTCGTGACTTGTGGTCACTGTCGCTTACGAGGCCGGTGGGAAAACCGGCAGAGTCGCCGGAAGAGGGTTTTGTTTCGTCGGATAATACAAGAAAACTAAAATAAAATAAGCCATGGTTTCATTTAATATTTATTATATTGTTTTTGTCATATTTTAATACATTATAATTAGTTTATAATTATATTTTCAAAGGACATACTTGGGATTTTGATAAATATTAGATTATTGGGACAATCTTATGTTAGATTTATCCTGTTAGGACAATGTATATGTTCTTTTGACCCAAAAAAACAATTTTCCCTAGAATAAATTGGGGTAAAATAAAATCAGTAAACAAAAACGCCTCCGTAGCATAGTGGTATTGCGTTCGCTTCGTAAGCGAAAGGCCGCGAGTTCGATCCTCGCCGGGGGCTGTATTTCCTCTTATTATTTGATTTTGTTTGTCTACCCGCGGCGTTACTACAGAAAGTTGCATTTGTCCAACTTATCCTTCATGCTAGTTTTACACTACAACTCTACTATCTACCGATTTGTCATCTTCTTAATTTTTTTGGGAACACTTTTAACCTCAGCTACTATTCTCTATAAATGGATTCTGTGGACCAAAATTTTACATATAGGATCTACTACATTTACGAAATAATCATTGACGCTAACAAAAAAATCAATATAAAATTTTGAGAAACCTATATGTAACATGATAATCAGTCACTCCAATTGTTTTTTTTGTTGAGAAAATAGTCACTCCAGTTGTTCTTATGTTGTTTCTTGAAGTCAAGATCATTGAGGAAAAATCATACTATATAATCAATTCATTTATAAAAGCAACAATAACTTTATTTTAGAAAATGAAATAATACAAGGGGATTATTTCACGCAACGAATGAAAAACCAAAAAACTTGCAACAAATAGACCAATTAATACAGCACCACACACAAAGACGCCGTACGGCTCTTATGACTTATTCGATCCACATATATATATGCCAACAAATATTATCAAACAAAACGACATAGTATTGAACATCGCAGCAATTTGGGAGTATGCTTTTTAAACTTAAGGGCACTTGCGGCGACCACCGTGGGTGGTGAGGCTAGCGTAACACTCGCACTTGTCGTAGTTTCCGTAGGTACCAGGTGGCACACAGTTGCATCTTGCGCAACAAGTCCCGCACGCTCTGTGGCAAAGGTTAGGCCTACTTGAGAGTCTGCACCTTGCTACACAAGCACTTCCACAATCTACAAAATCAAACGTTATTAATTTTTACTAGTTTAACGTTAGGTTTATGTATGAATACTATGTACGTAGATGCTTGTGCGTATAAGGTAATATATTTACTAATTCTTCTGCCGTAACCCCTCTTTTTGTTTGAGTTTTCCTGAAATGAAAATTATAAATTGTAAACATAATAAACAAATAACCAAGTATTAAGTATTTGTGATGAAAAATACGTACCACATCGGCCTGGACGAGTTGGAGAACAAGAAGAGATATGAGAAGAGAAGCAATAAGGGCTTTTGAAATCGCCATATTTCTCGAAGGAGATGATTATAATATGAGAGATATATAACTCAATAAAGATAATATGTGTGTTTTGAGTTTTGTTGTGTCTAAATGAAAACTTTGAGGCTTCTATTTATAGCCCTCCTTTGATGGATATCCGACCCCATATTATGGTCTAGACTATTCTTTTGTTTTAATTATTTCTTATTAATAGTTTTTGGGAAAATATGATACTGGCTTCCTGAGCTTCAGAATCCTTCGGGAAAATAAAATATTGGCTTTCTGAGTTTCAGAATTCGAATATGATACGATAGGAAGTAGCCAAGTAGAACTTTGCAACAATTACGAATGATCATGAACAAAATTAAGTATATCAAACGCATTTTTTAATTTAACTGTGAAGGTCATAAATTTGTAATTTTGTACCAATTATAATTTTCAATAACTATATGTTAACTATATATAGTTAATTCAGAGATGAAAAATGGTTTATCATATTGATGTTTACGTGTGTATGTATAAGATACTTTTTTTCCTTTTCGAAACGGGTCGGTTGGTGCGTATAAAAGTTGTGCGTACGCGTAGAAGAGAATATGATGGTGGGTATTATTTGTGGTCTCTTGTTTTTAATTTCATCGAATGATTCGGTATTTCTCCACCATATTTAATTTATAGATTATTTTGAATTGTTTTGCTTGGTGAACCTCGCTTTAATGATCAGATAACTACGGACCAATTTATGACTACTGTATAAGTTTATAGATTTCTATCAGTTTATAGATTTCTATCAGTTGTGTTGACAAGAAAATAGACTTCTATCAACTACGTACACCGAAACTGCTAAACAGTAAATTCTATTATTGGTTTCGCTTGGGTTTGGTACGTTCTGGTTCTACCAGAAATATTGGCTTCAATGATATATAAATATCAAGATTTTATATTTACAGTGAGCTTGTTTTATGCAATTTTGAAGTATACACGGACTACTAACTCCGGTTTTGAAGTATCTTTGCTATTTCCAGATGTACGTTATCTGGTGAAAAAGGTAGAATTTTTTTTTTTTTTTTTTTTTGAGAAAGAAAAAGGTTGAACTTGCTAAGCGTCTATGTATTCAAATCGAAACTGCGTGTGGTATGTTTTGGTTTTTGATGGGTGATGTTAATTTTCATATTTTAAGTTTTATTGTGTGCTGATCACCGTTGTTTGTTATCGATCACAATTATTGAGAAAATTAATTAAGAAAAAATGGGAGGACACGATGAAACTGTGAGTAACTCACGACGAAAAGGGACTTTACAACACCTTTAATCATAGGTTATAATGAAGTATTTCAGAGAAACAAATAAACAAAAAAATGATAGGTAAGTAAAAATCATTCGACAATCCAAAATATCCATGAAACAAAATTATAATATCTTTCATTTTAATTTAGTCTATTTCAATATCAAATTTAAAATATATAATTAAGATACTACAAAATATGAAAGTATTGGAAATGTACTTATAATATCTCCAATGTAAAATTCCATTTTTTTCAAAACAGAATAATTTTATAATGTGGTTGGTTTTATTCCAATCGTATTCCATATTGAAGTAAAAATAAGGTAATAAACAAAATATAAAAGTTTTATTCTATTTATGGTATTAAAAATGAAGTGAAATTTGAACATTTTTTACTCAAAACTATATTTTAGAGTGAAATATTTAGTGAAGCTGAAGATGTTTTTAAGAGTTTGATGTCGCTGAGACAAGAAGGATGAGGAGATGTTGACATGAGAGGTCCTTTCGAGAATACATCATTTCAGAGCTTGCACTTCTTCTTCTGAGTTATGAGTTATGACCCACTCTCCTCTATAATTCCTTTTGTTTTAAACCGGGTTCTGGATCAATCTTTACTATTTGTTTTAATATTAGCGTTTGTATCTTTAGGGTCCACTCTTCGCTGTTTTCTTGGTATTATTGAATAGTTTAAATAAAGAGTAGCAAAAGTAATTCGTATGATCTGTTTTAAGTTTTTGACGTTTGTTGGAGATTGGATATGCTTTATTGACGAACCAAAAGAATAAGCGCAGGTGTATTTAACCTGACGATGCCTTTAAGCAGTCCATGGCGGGTAAGCTTTTTTTTTTTCCTAAACTGTAAATATCATTAAAAATGAATTGTTTGGCGGGTAAGCTTTGTGAACAAAAAGTTGAATCAGCTCGATTGCAACGGTTGGCTTGGCTTCAGAGTTTCAAAATAACAAGGGAACGAACCCTGGAAAGGAAGAGGTCACGGATCGTCTGTCTTCTGTGTTTGGCCTTTTCACCAACCTTATCGACTCAACCACCATCCGCACGCACATTACACAAAGTCTATAGTATACCATAATCTTTTAATTTAATATTAATAAATTTCTTTGCATGGGAATGTCACTTCAACTATCATGTCTGACACATAATATCACACGTTCGGTAGTCGTCACTTCAATAAACTCTACGTTAAGTCGAATCAAGTTTGCACCCAACTTAGAGTACTGTCCCAATTCGTAAGTATTAGACGTTTTGGTTTTATCTATAGTAAATATATATAGTTTTATAGATCGATCACAGAAAGGGCTTCCATATAAATCCAGTAACATGAAAAGAAAAATCCAGCAACATGTCAGACAACTAAGCAGAATTGTTGTCATGGTAACAATTTAAATTCTAAGGAGCATATTACTATATTAGAAAAGAACCGAGAGTATGAACCATGTTTGATGATTGATTCATAAGTCATAACACAAGAATCGATGAAACGTAAACGGCAACATTATATTAGTTTCCTAAATTGTAGTTGCTTAAATATCTTGATCGCCATTAACTGATCAGAGCTAGGTTATAGCATGCCATGCTATCGAAAATAATCAATTTTATATGCTTTAAGGAGTTTTTTCAAGATAAAGTCCTCATGAATTGCGACACTTTGTACTATCTTCTTTTAGTTTAGTCATCTTGTAGAATAAAATTTTTGTTTCAAAATAATTGTTTAGAATTTTAATATAAAATTTATTAATAATATTTTTCAATTAATTTTTCAATTGGTTGAAATATGATTATGTATATAGGTAATGAAGATTTTATTTTGGAAATATACAAAATTAAAAATTCTCTTAATCTGTATGCATAAACCTAAAATAACAATGAAAACGAAACGGAAAGAGTATTCATTACACTAACTGACGTATAATCGCAATTAAGAGAAAAAGTGATTGAGTAGTGAGTTCTTTAGCCAGCTGTGAAGTGTTGTTGTTCACTTACAACTGTTATGTAGCTAATTGCCAGCCTGTTAACTCTTTTACTTTCTTTTGTTCCTATCCCAATCTATTTATAATAGATCCTATGGATGGTTTGTATTTATATTTTCAGTTGGCTTTCTTTTCTTAAAAAAAAAAGTTGAGGAAATAGAAAGCCAAAAATCAATTTTAACCTCGTCCTTGTATTTGTCATTATTCTCAATTTGTGTGAATAGTAATACTTAAACATATACATGTAAATTGATAGGTAACTAATATTTTGGTTATGTGCAGAATCGATTAGAAATCGATCAAACATGTGTTTGGACCTGCAAATATGTAAAAGTTCCCAAATTTATAAGGATATTGATTGAATTAAGAGGACATAGGGTCCAATATATGTAAAAGTTTATAAATATTTACATAGTTTGTTAATTTGTGGGTAAAATGAATTGTATGCTTTGAAACCCTAAACATTCAGGGCCGATTCTTGTCAATTGAGGTTAAATGTTACATTTGTTTATGCGAGTTCCACCTCAATTTCAAGAAACATTACTCTTCACAAATAATATTTGCGGAAAATCTCTTAACTTTCTGATAAAAATCTTAAATATGCACCCAAGTTATCCGAACATATTTGGTAATATACATCAAGTAATTTGGTGGAATATTAGGTGATATGATTACAAAGTTTCCAGCTGTAGCCGCCGCCAAAAGAGTCGAAATCGAATTGAAAATGAATTTGGATTAGCTCAAATTTGAGCACAAGAGTTTAAAACAAGTGAAAGAAACAAACAATAGCTGCATGAATCAATCATCATCACTCTTTAAAGTGTTAGATAGTAACTAATTCGTACAAGGGATAATAATAGTAATTCGTCAATACTCATTACAGTCATTTGCATATGTATATCCATATAATGATTCTCATTTCCTAAGTTTAGTTTCCCTTAGTGTATAACAAAAAAGTTGTTCAAGTAAAAGCCTACAAGAGAGTATCATACTGGGATCGATTGGTTGGATTGAAGCTGTAGGAAATTTACCGTAGAATTCAGACTATAGAATTTTTTGATGTAGCTATACCTAGTGTACCTGTAAAAATTAAATGAGAAAAATGATAAAAAGAAATATATTCCTACAGCAATATTTTAAAAAGAAAATAAATTTCTACAGCCTTTTTTTTTTGCTTTAGAAAATAAGGTTTCTACAGCCATTTTTTATTTTATTTACTTTAGCTTTAAAAACTAGTTAAAGCATGATTGGTAACTTAAGTGGTTGTAGATATAAATTAAAGCTAAAGTTACTAGTTGCAACCCAACTAATCACTCCCACTATATCGGTAATATGGTATTTGCAACCTAAATTAATTTTATGACTGAATATCGAATTTAAATTTTCAGCATTTGTAAATACAGTATGTATTATCTAAATAGATATATTGCCTTCTAAGTTTTTCATATTAGTAGAAACTAAAAAAGATTAAACCATCTCTAATGATTTATTCTATTTTTTACTTTAAAATAGTATAATTCTATAATAGAGTTGAAGTTTGCTCCAATAGTATTTTATTATATAGTGAAAAACGGAGTAATAACAAAAAATAAACAAATTACTCTATATTTGGAGTAAATCTATTTTTCATTCTATTAGACCATCTCCAATAGTTTGCTCTATTTTTTACTCTAAAATAGAGTAACACTCTATAATAGAATTGGAGTTTGCTCCAATGGTACTCTTTTTTAGAATAAAAAATAGAGTGATGAACAAAAAATAAACAACTTACTCTATATTTGGATTATGTTATAAAATAAAAGGGAAAAGGATATCTTATTCATAAACATATGATCCCTTTATATAGGAAATTACACCGTCATAGTAATATGGAAATAATACAAAATCCTAAATAAGAAAGGAAAAAGGAAAACATACATAACCAGCCGGCTAGAACCAAATCATAAACAAGCCGGTTATTACATCCACTAATCTAATGGATTATAACACTCCCCCTTGGATGTCATAACCATCTAGAGCTCGTGATACACTTTACCAATATTGGTGTTGCCTCATTAAAACATTACCAGTAAAACCCAATTGGGACAAAACCAGGTAAAGGAAAAAGAGTACAACATGTATCACTCCCCTTGATTTGTACCTCCGTGCATGTTCTTCAAGTTGGCGCATGGACAATCTGATAGTTTAGCTTCATGTGCGCCAAGTCTTGAACTGGTCCTTTAATTGGCACGTTCCAAACTGATAGATAAGTTTCATGGACGTGGAGTTGGTGTAGACATGGTGAAGAAGTCGGCTGACATATCATATGACTGAACTTGTAGTACTTTGAGCTTATTCATCATTTTCCAAACGTGTATAACCTGTTTGAGATCTTGTATGGATCAAATGTTATAACCTGCATTATTTATCAAAACCAAACAAACTTCTCTTTGGGTTTGGTTAGTATAAATCAATATTAAACATTTAGTTCCTTTGAAGGTAACATAGGATGTATTAATCCTATCCCATTGCCTCATGTTCAGACATGCCAAATTTATCTAAGCAAGGTGCCAATAGTACTTATGGCAAGGGACGTGTATGGCTTTAGTTCCAATGCCTTATGGTCTAAACATATACATAAAACTATCCGACCTAGTATGGCTTGCCATATCAAGGCGCCAATTATATCAATATAGTGCACTTTTGGTCGGATGTGCATAAGCTTTAATGGTACTTATGTAAAGCCTTTCCTGGACCAAGGACATATGCCTTGTCCAATCTTAGGATCAAAAGGATCTATGACCAAGCTTAGGGGTCCTCAAGACTATGTCTTTTCTTTGGACTAAGTAGGTCTGAGTCCAAGCCAAAACAGGACCTTACGACCATAGGACTTACAATGGTTTATGTCCAGACCAATGTACAATTTCTTTGTCCTTCCTGTGACCAAATGGGTCTATGTCCGGACAAGAGACTTTATAACTATGGAACCATGTAACCATACAATGGGCGAGTCATTCATATAAAACGTTTGAGTACCAAAGGACCTTATAGGACTTACTCAATTGTCCATGCTAATCACTTTAGCACCTTTCTATGTATGCATTTGATCACAAGGATTTCATCTTTAAGATACTCAATTTGTAGTTCTAAACAATCTTTGTTTTTCCCAAAACTTAGTCTTTCCCAAATCTTTCATTTCAAACTCTTTCTTTAGATATTTATTTGGGAAATGTCTCTCCAGAGGTTCCTAGGATATTCTTAGATCATTTATTTGGGAAATGTCTCTCAAGTTTGTTCTCGAGAACTTGTTGCATTTGGNNNNNNNNNNNNNNNNNNNNNNNNNNNNNNNNNNNNNNNNNNNNNATATATATATATATATATTCATTATCCAGTGGACTTATATAAGTAGGCGGTTTTTCCATCCATGAACCGCAAGTATAAGTTTCTTTCTTATGTACCAGACTTATCTAGGAATCTCAACGTTTGTTGCATCCACCACAAGGAGCATATCTCCTTATAATTGATTCCTGATCTTTGTGAGAATCCTTGTGCATATGACCATGCCTTATATCATGTATATGGTTGTGCCTTATATCAAGCATATTTCGCCATGTTCATTTCTTTCACAAAGATTTATTTCTGTCCAACTGGTTTAACATCTAGAGGTGTCCGAACTTATGGGACCAATCCCCTCTTTTCTTAATAAGTTTAACTCCACGGCTTCTTTCCATATGATCTTATTCTTATTGTTGAGTGCACTCATATATAGAAGTGGGTTCATGATCCTCATTCATTTCATAATCTCAAGTGCTACATTGCAAGCATAGTATTAGTATATCATTGATGTCGAGTACTCTTTTGTTCCATTGTGTCCTAAACATGACTTAACTTATTGAGGTTTCATTATGACCTTCAGTACCCTGATGCTAGGCGTCCCAAGCATCATTCTAGGAACCTTAGGTACAACCTCGTCCATGGCCATGGCTTTTAAGTTTATACATCTTATGATTGATTATGTCTAGGAGCCCCTCATCCACGGATTAGCACCTTTCTTTAATATCCAAGGGATCTTATCTTTAGAATTCATTGGTCTATCATGTTTTCAAACATGTCTTAGACTCAGTAGCTACTTGATTGTGTTCTCTTGAACATCAATTCGAATTTGGTGCATTCATAGCTGGTATAGTTGACTTAGTCATTATATTCGGGTCTGCATTGGAATCTGGCAATTTGTTTAAGCTAGCTTTTGTATAATCTTTTGGGCTTCTAGATCTCATTCTAGAATCTGAGGATCTTGCCAATATGAGGATGTTTGATTCCATGTAATTTTCTTTTACCCAGCTAATTCTTTTCTCCCCTTAATATTGGATGTTCGGATTCACTGAAGTGACTATCCGTGTACCTGGCCTTTAAACAAATCATCCGTGGTTGGCTAAAGGTACTTAGAAACATGGGGAAATCATATATTAAGTTATAAACGGCACATCCATATATCTTAAGATGGGATATGTTATGTCTGGCTCTTGACCCGTTAGTAATTTGGGATGGAGAATATCTATGTTCACTTAGATCCGTGTGTATGTCCATGTGCCAAGGAGTGTTTTATACTTACCCCCCCCCCCCATGGACATACCATACGTAATAAATGATTTTGGGATGTGAACATATCAAGACATATAGTCTTTTAAGCGATTTCAAGGAACTCTTTTCCTTTGCCATTAGTTTCATCTATGTTTACTTCTTGTCCTTGTTCATTCTTTGTGTATGTCCACATCTATATTTCTGCCATGGGATGTTTTATACTTACCCCATGGACATATAATGGTTATTAAACATTTGGGACGTGAACTCTTTGACATATAGTCATTTGGACATTTGAAGTAACATATTTTCTTTGTCTTTTGTTTCATCCGGATGAGGATTGTCTATCTTTATCTCTTGTGGACTCATTGACACGTCCATATTGTGTATGTCCATGTGTCATGGAGTGCTTAACACTTACCCCCATGGACATATCATATTTATCGAACACTTGGACGTGAACTCATTAGCTTTTATCAAGACATGTAGTCTTTGCCACTTTCTTATTTCTTTGGGTGATTTCTCTTAAAGAAATTCTTTGTTCTCTTTTACCCATGGTTTCAATATGAGACCATCCATTCTTTCATTTAGACAAATTAGTATTGTACCACAGTCTCTGATGGTACACGGCCTCTATGTGAATAAAATGCACATAGTTTCTATTAAGGATACAATGCACCATCTATTTCTAGATGTGTACCCTTGAGGCCATAGGATATAAGTTTGGCCGTGGCATTTGTTTCTTAAGATGTTGTGGCTTGATCCACTATACACCATAATGATATTCACAAGAATAGACGTTTTGTTTTAGAGATAAAAGCTTTCATTCATTCAAAGATAGAAAACAAGGAAATGAAACAAGTTCAAAAGGAAAACGAGAGATCATGAAAACTAAGTAAAGATGCTAAGTGTTTTGATGTCGAATCAACTGGACTAATTAGGCAATCTAAAGTTTCAAACTCCATAAGATCATTTTTATAAACCATATGAGCTTTTTGGATTCTTCCCTTTTAGACTTTCTTGGTAGAGCTCAATTAGATGATTGGGAGTTCTACATATCTTAGCCCAATGGTTACTCGTTCCACACCTATAGTACACTGATTTGGACGTGCCTCTTCTAAGTCCAGAATTGGACTGATGGTCACGTCCATAAGGGTTCCATCTTGAACCCCGGCCATGGTTTCTTCTACCACGGCCTTGTCCATGTCATCTGCCACGTTTATTACTTCTTTGGCTATCATGGACATGGTTGGTTTCTTTATTTTGTCTTTTTTTCTAGGTGTGTATTAGGTAATGGAGTTGATCCCGGAGATCTCGTTTCACTATCTTTTATTAGCAACTCATCGTTTTGCTCAGCTTGCAATAAACATGATATGAGAGATGCATAAGTAGTGAAGCCCTGTTCACGGTATTGTTGTTGTAACAACACATTGGTGGAGTGAAAGGTGGAGAATGTCTTCTCCAACATATATTTATCATTCACTTCCTCACCACATAGTTTCATTTCGAAAACTATCTTAAGTAGGGCCAAATTATACTCGTCCATGGACTTATAGTCCTAGATTCTGAGATTCCTCCAATCATATAGAGCCTTTTGGAAGTATCATCATTCTTTGGTGATCATATCTAATCTTTAGCTCTTTCCAAAGCTCTAAGGGATCAATGGTTAGATATTGATTGTTTATATTCGACATCTGAAAACATGTGTTTTACAAGTTAGATCTTTGTTTCCAAAACCTATTTGGTTTATGATTTTGATCAAAACATTTTTGACAACAAGTCTGGTCGAGGTTTAATAACCAAGACTAATTAGGATTAATATCCAAAACCAAGATGGTTTATGTTTTGATCAATTCCCAATTGAATGGAAACATTATATGAATCGATTTCTAGTTCAAGGATTTTAATCAAACCGATAACCTAGCAACTTTGTTTCTATCTCAAGAACATGCATCATTCATTCAATCATGAGGTTATCTAACAACCTAAACAAAGATGCATGGTTCATGGTGTATGGGTTGAACCCTACGTACCAAACAAGTAAGAAACAAAACGATTTCATCTTAAGGTTATCTATCAAAACAGACTAGCAACCTAGGCGATTGAATCTATCAACTTAGCAAGGTATAATCAGGACTCATCAATCAAGACAATCAAGACATGAATCAAGGGTTTTAGAACATAGAAAACGATTAGGGTTCATCTTAGTTTTAGGTTTCAATCAATTACTTACCTTTAAACTCCAATTGGATTGAATGGACATATGTATGAGAGTTGATCACGAATTTAGAATCCTTAGCCTCCAATTGGAATATGAATCAGGAATTTGATCTTGGTTTAACTTTAAACACAAGAGTCAAAAGAGAATTAGAATAGAGATCAAAACCAAAACGCAAATTAAACAAAAGAGAGAGAGAGGGATTTGCGCTTTAGGGTTTAGATCAAGGAGCATGTCGTAGCTGATGGTTCTTGAGCAGCTCATGTATTAGGATTTGAAGAGGAGCATGTCATGAATCAAGAACAACTTGTGGCTTAGAGACCTTAAACAAAACGATTCAATAAAGGGTTAGTATGAATCAAAGCAAAATCGCCAACAAGAACAATTTATAGAGAGATTGCGGCTTAGGGTTTTGATTAGGGTTTTGATTCAAGATCAAGTTGCGGCTGGAGCTTAGGGGAAAGCTAGGTTTAATGTCGAAGCTTTGGCTCAGGGATTCATAGATCAATCCTTGTTGGGAATCGGGTTTGGTTTCCAAAGCATATCGGCGGGCACTGTATCTGTGCGTGAGGGCGCGTCGGTGGTCAGATCGACAAGCAGTTTGCATTGATGGATTTGTCTCGTCGAGGGCTTCAATTTGATATCTTGATCGTTTTGTGGTTCCTCACGGTTTGAGAGAACGACCTCTTTGAAGTTCCAAGGTGATTTCCTTTTCGTTTAGGGTTTGATGATTTTTAGGTGAATTGAGGAAATATTTCGTGGGGATTACAGCTAGAGACTATCGTGCTGATAACGTGTTATAAAATAAAGAAGAAAAAAATATCTTATTCATAAACATATGATCCCTAGGAAATTACACCGTCATTGTAATATGGAAATAATACAAATCTAAATAAGAAAAGAAAAAAAAAACATACATAACCAGCCGACTAGAACCAAATCATAAACCAACCGGTTATTACATCCACTAATTTAATGGGTTATAACAGATTAAACCTATTTTTTACCCTATTATAGAGTAAAAAATAGAGTACCATTGAAACACTTAACTCTAAACTCTATTTAAGTGTGAAAAATAGAATGGAATTGGAGATGCACTTATAAATTGAAAAATAGAGTAGTACTAGAACATTTTTACTCTAAACTCTATTTTAGAGTAAGAAATAAAATGGAGTTGGAGATGCCCTTAAGAGCTGGAGAGGCTGTTCAAAATCTCTAATCTACTCCCTTGTGTTTTACAGTAATCTAAAGCAAGAAACATAAACGTAAGAAGGGTGGCCTGCAAAAAAAAAGAACGTAGAGCCAAACTCCAACACCATAATGGGAGCTTGAAACCACACTGTATCTTCTTTTCTCTTTCTCTTCAACGCATTTTACACAACACCTGATGGGAGACGAAGAACGAGCTGGCCACCCATTAAACTGCCAAACACGAAGAAAAACATAAAAAAAAAAGATCTTAGAACACCCATCATTGTCATTATTAAGTATAGAGATTATTAGATAGCATGGAATAATTACAACAAATAAACAGCATTTACCTCTTGACATAGTAGTAGCAGAAGTCAGCGAGAATAAAAGTTTGTACTACTTCAGAGAGGAGGACCATTATAGGCCAAAACCCATATCCTAAAGCAGTCAGTAACCTCCCTCGAGTGTCCCAGACCTGAAGGACCCAGTGTGCACAACTCAAGAACCTGGCTATCCCCAATGCAAAAACGTAATGTGCTGTGAATGGCTCCACGATCTGAGACAAAAGACATGCGTTACTAGTTTAAAACCGTAAAAAGAATATAGATAACGGTTTCAAAAGCTTTTACAAAGCACCTTAGTGTTTTGCATGACTCTAAGCTGAGGGAGAACTGAAACAGCTTCAAGGTATACGCAGAAGGCCCATGAGACTTTGTTGATTATATGGTGACGTGTTGATGGGTGAAACAGGACTGACAGAACAGCACACGGTATAACCTGCAGACATTATTATAGAAAGCAACTCACTTCAACAATGCAACTCAAATTGATATTCAAATTGAACGAAAAATTAATTCTTTAACCAAATTCAAATAAAAATGATTCACATTCTTCAAAGACACAAGAACTTACAACGTAGTAGATAGGAAAATTGTCTTTGTCTTCCATGTAACTAGCTTTGAGCTTGAACCTGATCATGTAGATAACCCAAAGGGTAGTCACCAATGTAGCCGAATCAAGCAATGTGTGAATATCAAACTCCATCACAAAGCTACAGTACAGTCTCACGGCCAAAAACAGAGCCGTTAACTCTTGTGTCTTGAGAGATAACCCTGTCGTCCAAACAAAAAGATTGCTATCTATCAACCCTAATTCGAGAATTTGGCAAGAAAGAGAGATTTTGATTTACCAGCGCAAGTCTTTTCCTTGGTGAGTTTGTAGATAAGAACGGAGATTCCAAGGGCATGGACAGCCTCGGCAGCGACGAAGAAGTTGTCGTGGTCGTGAACAATCATTCTTAAGAGAACGAGAGCAGTCATGGCGGTAACAACACCCAGAAAGGCTTTGACTTTCGGTGGTTGCCGGCGAACCCATGTCGTCACGGCGTGGATTGGCTTATTCGCCGCCTTCATCTCCTCCGATTTCACTTTTCCGAGTATAGAAACGGTAAAAAGTCGAGATTTTTGGTAACTTTGTACGAACCAAATCTATGTTATTACAAAACAAAAAGAAGTCGAAAAGGTTTGACACTTTACGACGGTGCCACGTGTTGAAAGTTAATTTTGTTTAGGTGGAAAGGAGATTAGCGGTGAGCTAACGACTCGCTGCCTTGTCTCCTTGTGGGTATTGTTTTGGGCCTGGATAAATTACAAAATCTTTACAGCCCAACTAGGTATTGGATTGCCCCAAACAATCTACCAGCCCTCTCAAACGGTTTACGGCCTTATACAAAAGTACCAATTTCAGGTCGGGTTCGGGTTGGTTCTTGTCACGTCAGGGTCTTTCAGGTCTTTTGGATCCTAAACATTAAGACCCAAAAATAGGTACTTATAATTTTTCAGGTTGGTTTCGGGTCGGGTTTTGTCGGGTCGGGTCTTGTCGGGTCTGGATCGGTTCGGGTCCATTTGTCTCAGACCCGTTAATACCCATATTTTTTGGATCTTTGTGGTGTCGGATCGGTTCCAGGTATTGCGGATCTATTTTCGGGTAGATATCACATATTTCAGGTTGGTTCCTGGAATTTCAGGTCGGTTCCACATATTTCCGGATCTAAAATGTGAGTTTCGGATCTAAAATATGAGTTTGGGTCTATATATACTCATAAATCTGCATATTACCCGAATATAAAACCATAACCATTTACAAGGAGAAGCGTAAAGGGTACATCAGTTTTAAAATAAGCCATTAAGTCTAGTAAATATCGGTTTCAAAAATATCTGATCGGGTCCCGAGTTGGTTCTGGACCCGAACCTGCTACCCACGGGTCTTCCATTGCTAGATCCAACAGGGTAAAATTCTCGGTTCCAGTTTCGGATCCAAACCTGTATTTTCTGGTCGATTTCGGGTTGGGTCCTCGGATTCAGGTAAAATGTCCATGCCTTATCCCAATTACAAATCCAACTATGTCACAGCCATGGTCCTGTCCTGCAACTGTGAGTATGCAACTCTAATTATAAAACAGCTTGTTGTTCGTTTCTTTATTGTATGATTAATCGCAAATAATTAATTCAAGTAGCTGAAAAATTCAACGATTGAAAATTTTAATTTCTACGACTAGTCATATGATGATTCAGCAAGTAAACCAACAACATTCTGTCGCCAGTTGTAGTTTTTTTTGGGTGCTAACTGGTTAATATTATTCAAAATGAAAAGAGTTCGGATATAACTATGATCCTATACTTTAATTCCCTGGCAAAAAAAGCTAAAAAAAAACAGGGAGTCAAAATAGAACAGCTAGAATGAAACTAGGTGTAAGCAGGTGAGAGGAGGTGATCACTGAATCCATTTAATGAGGAGGTTCCTGAAGTGCTTCATGTTTGATCTTGCGTGGATGATAACTCGAATGCCAGTTGTAGTTGCATTGCATGTCGTAACCACTAGTAAACGAACAGATCCCATAATCACTACGATTTTGTGGGAACATGAAGAACACGTAAAGCTTTTGTGGCGATTTCATTGTTGTACAAAGTAAATGTCAGTGTCAGTGTCTTTGGTGTGTTATATCGTGCTGATTTTGAGAGTGGACACTTGTCGTGGACAGTGCAACTCTAGAGGAGATAGCAACGCTAGCATGTTTCAGCAACTCAGTTGCAATGCATTGCTACTCAGCCTCGGCTCTGTAACGTCATACTCTTGATCAATAAACTTGGCTCTACACTCATCAAACCAAGTCAAACCAAAATTAAAATATTGCAGCTTGCGGTAGTCGCCATCCCATCCTTTTTAATACGCTTAATTAACTAACAAATGAATATACATTCTATATTTGTATCACCTTAATGATATTGTATGGTTTTAGAAGAAAACGTCTTTGCCTCAAATGGCTAACGCGTTATTTATCCAAAACAAGCCTTCTCTGCATCATTTTTGTATTAATTATAATCTATAAGGTATGTATAAAATTAATGGTCTACTTGCAAATTCCATCAGTTAATTAATTAACAAGTTGAATAATCATTTATTAAGTAGTCTACTTGCAAATTTCTTGCTCAAGACTTATTTATTGCACTCGTCTCTCCCTTTCTAGCTACGAGATCACCATAGCTATGTCTTTTTCCAATCACATCGTGTCTCGTTCTCTTCCAATATGGCTTTTTATCTTCCTCATATTCTTAGTATTACTCGGAAAATCTCAAAAGGAAGTTTTGCAAGTTAGAGTGGGAGTAGTTCTTGACACTAATTCGACATTAACAGATTTGAGCTTGCGAGCTATCAACATGTCCCTGTCAGAATTCTATAACACTCATAATGGCTTCAAGACAAAGATTGTCCTCGACATCCGAAACTCCAAAGGAACTGTTGTTGGTGCTGCAGCTTCAGGTACACAATATAGTGTTGTGAAACTTCATCTATCACGACATATACTATATTATCCGTTAGAAGTTATTGCAATTTAGTGTCCATACTATATATTATTTCCATTCTCTTACATATTATATATTTATTTATTTATATTACCAAAATCTTTATCAAAAATTAGAGGCCGGTCACGGCAGGTTGCGGTCAATGCCGCAGACATAATCTGTTGCAAGTTCTACATTTAACACATGAACAATCGCAACTATATATATGAAAAATACTTATGATCGATCAATGATAGATCTGACTGTGTTGTCTAAACCGGCAAATGAGTCATATGAATTCGAAAGCGGAAGGCATTTAATCAGCAATATTTTGACAAACAAAAAAATCTATAATATCTTCATGAAATACAAGGGTGTAAACATAAAACTTCAACTATTATAGAAGAAAAAAAGTCTTTGGCAGACTATAATTCGTAACGCATATATNNNNNNNNNNNNNNNNNNNNNNNNNNNNNNNNNNNNNNNNNNNGATTCATCTAATTGCAATTCTTTTAACTCAAATCAATGAACACACAAAAAAGTACATGTGAACAAGAGACATAACAGAGATAACGGTTTGACATGGAAAAAAATATATAGCGCAATTTTAACATCCTAGAACCAGGGCCGGTCCTGAAATTTTGGAATCTGAAATTATCTTTATGTAAAAATTTTGTTTTTTACAAGTATGTAGGTCTAAAAATATATTTTGAAGCTTAAATTTATGTAATTTTTTTCAAAATTTTAGGAACCTTTAACAAATATTTTATTAGCCATGACTTATGGCTGACCCTGCTTAGAGCAGCGCTACAACGCACGCTCTAACAGTGATTCTTACCATTTAAAATAAAAAACAAATTAACAAAATTAGGAGAGAAAACATACAAAAATTTTAATCTAAGAGGTTTTCAGTTACAAACTTTATAAGACAGCTGTCACTTGAACAATGTTTGAACATGTTTTAAGAAGCTAAAATAATTCTAACGTTAATTAATATTTTTACTTTTTTTTATAGAAATTGTCACTGATTCTAACCGTTTTTTGTCGATTTTAACCGTTAATGCAGCTTTTAAGTATTGTTTTTTTCCCTACGGACCCCAACCAAAATATGAAAAAGGTGAAACACACAACTTTTTCATTAAAAAAACACACACACACACACAACACACACAGCTTCTTACACCAAAGATTCAAACCTAGAAATTTTGAATTTATTGTTCCGTATTCGTTAATATATGTATCCAGTTGTATTCGTTTTGCAAAATCGGCATATCTACGGATATTAAGATATGCAATGTCAAATAATTAAGATTTAGTAAAAAACTAAAAGGTACAATATTTATGAAAAAGATAATAATTTCAAAAACAATAGATAAACTTGGAAAAAACAATACATAAAAACTTGGAAACAAATACATGAAAAATAGCTAATAGCATAATGAAAATGATGGAAAAAACAAAACAAATATGATAATTATCAGATAAATTTTATATGTAATTTTATACTATTATAAATGACATCCTTTATATATTATTTAAGAAACATTACGACATTTAAGATATACCATGTATTATCAATAAAAATGATTTTTAGACTATTTAGAAAAAATAGATTAGTTCGTCTAAAAATATATTGTACTTCTAATCAAACAAACCATAATATTAATTATTAATGTGCAAAAAAAAAAATTCTTCTTTCTTTTCTTAAACAAAATTTACTAAATTACCTTAAGTGATTAAATAAAAATGATAATTAACGGTTTTAAATAATAAAGATTTGATAACAATGTGTATCCTATATTCCTATATCATTTTTGTTTAATTAATATTATTAAAATAAATTAAACAATTATATTAACCATATAATAACATTTAGATTTTTTCATGAATGTTAAATTTTGAATTTTTAAAAATGATTATAAATTGCTAAAACATTTAAGAGTCCTAAAAATTTTGTGATCAATGGTTTAAATATTTTTGTTATAACAAGATATTAATAATCATAAAAGCATATGAGTGTGAAGTCTCATTTAAAAATATTCTGATCAAAGTATATTGTATGCCTATTTTTGTATCTTTTAAATTAACTATATATTTTAAAAATACATAAAAATTTATTTTCAAATTTTCAGTGAACAAGTATTGAGAATTTAATATTTAAATTTCAAAATTTACATTAATTTTTTAAAAAAGATTATAAATTAATAATTCTATTAAAAGTCTCACTTTTAAATTTTGTTATCAATAGTTTAAAATTTGTTTTATAAAAAATACAAATTTAATATATATATATATATGTATATGTATATATAATAATAAAATATACTATATATCTATGTTAATATCATTTAAATTTAATTATATACAATATAAAATAGACAAAATGATTGTTTGAATCTATTTACCATAATATGATTATAAATTAACAAGAGAGATTATTTTGATTTATGTCCTCGTGCCAAAATAATTATTTNNNNNNNNNNNNNNNNNNNNNNNNNNNNNNNNNNNNNNNNNNNNNNTATATATATATATATTCATTATTTTATTATTTCTAATATTTAAAAGAACGCATGCAAAATAAGTAATAACACATTAAAATAATGTATATATAATATGTTCATTTCGCGCAAAGGCGCTAATCTTAACTTTATATGTGTATTAAAGTGATCTGGATATCAGGAGTTAAACTCCTTTAAATCAAGATATTCAGACTTTTTTAACTTAAATGCAATGTTTAATAAAAGCTTTCTAATGCAATGTTAACGTTAAACTTGCAGCTTTATACCTGATAAATAGGAGGAAAGTGGTTGCCATAATTGGACCAGGAAGCTCAATGCAAGCTCCTTTCTTAATCAACCTCGGAAACCATCTCAAGTTCCAATCGTTTCATTCTCCGCAACAAGTCCTCTTCTTGATTCCCTCCGAAGTCCATATTTCATCAGGCCCACACATCAGGATTCAGCTCAAGTCCACGCCATTAGCGCGATCATTGAATCATTTCGATGGAGAGAGGTTGTGACTATTTATGTAGACAATGAGTTTGGAGAAGGCATTCTTCCTTACCTAGTCGATGCTTTTCAAGAGATTAACGTTCGTATCCGATACCGAAGCGCTATATCGTTGCATGCCTCTGATGATCAAATCAAGACAGAGCTTTACAGACTAATGACCATGCCCACTAGGGTTTTTATCGTGCACATGTTGCCTGATCCTCTCGGGAAGAGGCTTTTCTCGATAGCGCGGGAGATTGGTATGATAAATAAGGGTTATGCATGGATTGTTACAAATGGTATAACTGATCTCATGGGAGGATCAAGCTTGGAGGATATGCATGGCGTCGTGGGTGTCAAGACATATTTCTCTAGATCAAAAGAGCTGGCGTATCTTGAATCTCGTTGGCGAAAAAGATTCGGAGGAGAAAAGCTAACCCATTTTGGATACTGGGCTTATGATACTGCCACAGCACTTGCAATGTCATTTGAGCAAATAAGCAACGTAAACATGAGTTTCAGTCAGACCAAAAACATTTCGGGAGGTGATAATGGGACTGATCTTGATGATCTTGGTGTCGCTCTCTCTGGTCCTAAGCTACTTCAAGCGTTATCAACGGTCAATTTCAAAGGCGTCGGCGGGAGATTTCTGCTAAAAAACAGAAAGCTAGAGCCAACGACTTTCAAGATAATCAACATAGAGGAAAGTGGGGAACGAACAGTTGGATTCTGGAAGTCTAAAGTTGGACTAGTGAAGAGATTAGGAGTGGATCAAATAGGCACTAATATCTCACACAGCTCGCGTCGTCTGAGACCAATAATTTGGCCTGGGGACACTACTATTGTGCCTAAAGGGTGGGAGATCCCAACAAATGGAAAGAAGCTGCGGATAGCAGTTCCAAAAAAGGATGGTTTCAACAATTTTGTGAAGGTAACAAAGGATGCAAACACTAATGCTCTAACCATTACCGGGTTTTGCATAGATGTTTTCGACGCGGTAATGAGACAAATGCCATATGCTGTCCCTTACGAGTACGTCTCTTTTGAAACTCCAGATGGAAAAGCAGATGGAAATTACGACAAAATGGTTCATAAAGTGTTTCTTGGGGTAAGTTACATTGATCCCTAGATCGTTCATACCTTTTATTTTATAACTTAGATAAAATGGCTTTTTGAAGGCTATAATTGATAACTAGTAAAATGAATAGATGAGAATTTTAAAATAGTTATTTATGGAATATAATGAATTCCAAAACTTTTAAAACATGGAATTATTTTCTAAATTGAATGTTTCTTTTTTTTTGGAATGATATGAAATACTTTTTTTAACTAGTGATATGAGAAATGTAAATGAAGACATTATTCCAAAAAAAAAGCATTCCGTTTGCATCCAAAAGTTAAATATTGAGTTGACATTTTTTATTTTATTTCTTCAATATAATGTTAACAGGAGTTTGATGGAGCTGTAGGAGATACCACAATCTTGGCTAACCGATCTAACTATGTTGATTTCGCATTGCCATACTCAGAAACGGGTGTTGTATTTGTGGTTCCTGTCAAGGACGAGAGAGAGAAAGGAGAATGGGTCTTCTTAAAGCCTTTAACCAAGGAGCTTTGGTTTACCATTGCGGCTGCGTTCATCTACATTGGAATCATGGTTTGGACTTTTGAGAACATAGAAGATGAGGATTTCAGGATACTGAGGACCATTGAAAAAATATCTAACGTGTTCTACTTCTCATTTTCAACCCTATTTTTCGAACACAGTGAGTTTATTTTATCACACAACTTCAGTTTAATCACACCAAACTATAATGACCACCTATGCAAATTTTTTTAGAGAAGCCATCAAAGAGCATTTGCACAAGGGCTCTTGTTGTGATTTGGTGCTTTGTGGTGCTAATACTAACTCAGAGCTACACAGCCACACTCACATCGATGCTCACGGTTCAAGAGCTTCGACCAACCATGAAACACATGGATGAGCTGAGGAAAAGCGGAGCGAAAATTGGATATCAAGAGGGTTCGTTTACATTCGAAAAGCTGAAGCAACTGGGTTTTCAAGAATCTAGGTTGAAGATTTATAATTCTCCTGAAGAAATGCGTGAGTTTTTTCTGAAAACTAGCAGCAACGGTGGTATTGATGCTGCGTTCGATGAAGTCCCTTATGTAAAACTTTTCATGGCAAAATATTGCTCAGAGTATACCATTATCGAGCCTAGATATAAGGCCGATGGCTTTGGCTTTGTAAGTAAAGCATTAGTTTTCTTTTTTGAGTAAAGTAAAGCATTAGATATAACTAGGGTTTTGGAAGTATTAACTCTTCATGTTTTTTTGGAAGGCTTTTCCCTTGGGATCTCCATTGGTGCCAGATATTTCAAGACATATATTGAACTTAACAGAAGGAGAGACCATGAGAGATATTGAGACCAAGTGGTTCGTTGGAGAACAACATTGTGTGGACCGGACCACATCTGATTCTCCAATCCAGCTCGATCTCCGCAGCTTTCAGGCTCTATTTATGATTGTCTTTGGCATCTCTCTAATTCTACTTTCTATCATGTTGTCTGGTAGAAGATACCTAGAGAACAATAAAGGTGGACAAAATCATCCAGGCGATGGGCCTGAAGGCCAAGCGAACACAGGAGCCAATCAAAACCGTGACGTGAATGAAGGTGGAGAGATAGCCAATATTGAATTAGTTAATATTAGGGGTGGAAATGATGAAGCAGATGATATTGAGAATCAAATTGTGGAAGTCTATGAAGGAGGTAACGTTAGAGATGGAGATGAATCAGATCATGGTGTGGAAGTCAACGAAGAAGGTAATGTTGGAGATGGAGATCATGAAGCAAATCATATTGGGAAAGTCAATGAAGTACATGTGGTGCAACTACAAAGTCAACAGCAGTCACAAGCAGGCTTAGTTCACCGCGGCAATAATAAGAAACTCAGCTCAAAGACTATGCCCTTAAGAAGGGCAGTCCCACCTTTGAGGATGCATTTGGCATAGTTTAAAGAGGTCTTCAATCCGAGTCTCAGTGTGACTTCTGTTTTGAGTTTTTCTAAGAGTTTGTATTGATCAGATTTTTATTCCTTAGAATTTTCAATAAATGCCCTTTAATTTTTTAAATTTCTGTAAATTGTAGAACCCAAGCCCAAAAAAATTTCAAAATAAAGGCCTCTAGAAACTCTAAAAAGAATTTTCTAAAAAGAGAAGAAGAGAAGACATACATTTATTTCACAAGAAAGAAGAAAACATAAGGACACGTGTCATAAACCTAGACCATCAAATAAAATAAAAACGAAACAAAAAGCTTCCCAAAAGATTTATCTTCTTCGTTACCCCAAATCGTCTAAACTCTCAAGCGACCCCAAAAGAACATTACTCGAATGTTACAGAAGAAGAAGAAGACAACAACAACAACAATGGAAGATCACGGTTCTTTCCTTTACCGTAACATGGAAGGTAAACAAGGTTTCTTCAGTGAAATCGACTACGAAGTTTCTTCAATCTTATTGGAACTCTCGGACCCTGTCGTGTTCTCTTCCGACCCTCCTCTTTTTCATAAATGAGGTCGTACTAAAAAGAGGTCTTGCACCGTCTTCCCTCGTCCGCCGAAGATTCCCCCGCCGTGTGCGGAGGTCGTCGAGATGGGAAGTACTTCGAGTTCGTCATGTTTAACCGGCGATGCAAAGAAAACATATCCTCAAAGCGTAATTCTTATTTTTTTTTTCTTATATTATCTGAATTTTAAATTACTATTATTTTCTATGTAACTACTGATTTATTGTTTTTTTTAAATTTAAACGACAGATTAGAAAAGGTTCTATGAGAAATCATAGCTCGGAGCTGAAGATTACTTCTCAGACAAAAGCATCATCGTGTTCGGTTCTCGAGGTTGTTTATTTTCTCCATTTTGTTTTTGTTTTTTTTTTGTTATCTAAAGATATGAAAATATTATTTCAATTTCTGAAATATTCTATGAATATACAGTCGGAGACGAGTTTAATGAGGGCGCAAGCTGGGTTACTACTAGCCCAGCCCATTGGAGAAAACCTGCGTCATCTACAACCGTTAATTTACGTTGGTGATCCGACGGCCAACAAGAGAGATAACTTGGTACGACGTGGCTTTGATCTGAACCTTCCAGCAGAAGAAGAAGGTAAGAGCATGTTCAATGCTGTCTCTTAATCGCGTCTCTTAGGGTAATTAACCCAAAAAATAAATGAAAAAAGCAATTAAAAGGAAAGCACGTCTCTTAATTTGGAGTTGGAAGGCACGTCTCTTAAGTGCAACGTGTCACAAAGGCAATGGGTAGAAGAAACGGTGTAGTCGTTTTTGCTTCTTTCTTCCTTCTCTCCCTTTTTGGTTCCTCTCCGAAGGCGATGAAAATGGTGATTTCTTGGACTCTTGGTGGTTGTCGTCGACGTCTGTAGGGGTGTCTCTCGGCGACGATGGATAACGACGAGCTCTCAACCGGTGGCTAGCCTCGACGAATGAAACGGCGATTTCTCTCCCTCTCGGTGGAAGGAAAACGGCTCACTCTTTCTCGTGGTTGCTCTGTGAGACGAAGAAGAACGGCGATTGTTTGTCAAGGGAATCGAAAAGGTAAAGCATGTCTTTGATTCTTTCAAGTATGCATGTCGGTCATTGTCCCCCTTCTTGTGCGTGAACCGTAAACTGAAGAACTCTAATTTTTTTTTTTGTTTCTATTTTGTGTTAGTACATGGATGGATCAAGAGTCATGATCCAGGATCGTATGAAGTTTGAACAAAAGGCCTACAATTGTTCATTATGGGTAAAACTTTCATCTTTACTCTGTCCAATTGAACAAGGCATTGTGTGTATTCGATGGTGGTTAACGTTTATGCTGTGATTGATGATGATTCTGATCAAGGTTAAGGGACAATGAAGAAAGTCGAGAGTGATGGTTTCCCTCTGTTGAGTTTGATAAAAGGTAAAAGCTTTCTGGCTTTTGAGTTTGATAAAACCATATGGTTCTTTACTGTCTTCAGTTGAATAAAGCATTGTCTGTAATGTAGATTGAAGACGACATGACTCCGGAAGACTTTGCTCTATGACCACAGCAAGGAAGCTATCTCTTTCCTCTGTTAATAAAGGTAATAGCTATCTCTTTGCTCTGTAAATTTTAATAACTAGATTTAAGTGGTTGTCTTTGCTATCTTGATAAGGCATTGTGCTAGGTTAGATAATAACTAGATTTAAGTGGTTGGCTTTGCTGTCTTGAAAAGCATTGTGTTAGGTTTGGTAATAAATAGATTTAAGTGGTTAGTGTTAATATGGTTGTAATAAATTGAGATGAATGAATGCTATAGATATATGTCCAGTCGGTTGTGATCAATGCTTCTCTTCCTTCTATTAAACTTTGTCCTTCTCCTCTTTCTTGAACAAGCGCACGAGTTGTTCATCTCTTCCCACTTCATCCCCTTTGTTTTTTATTTTCTCTCTTCTTTGTCATCTTCCCTTATGGATTCCCATCCATATTGGCAGGGTCCGAAGCTTGTTGATCTACTAATTAGTCAACAAGATATTGCCTTTGGATCCAATGAAGATAGTGTCGAGCAATCTTCAACCCAAGTTCCTTTTCTTGCCACACAAGGTACAGCAGATTCAAACTTCGTTGGAGACAATCCTGCTGACCGTAGAGAAAGAAGAAAATGGACTCCAACGGATGATGTTGTCTTGATTAGCTCGTGGCTTAACACTTCAAAAGATCCAGTAGTTAGCAATGAGCAAAGATCAGGCACGTTTTGGACAAGAGTAGCTGCTTACTATGCAGCAAGTCGTCAAGATGGTGGGTGCGAACAGAGAGGGACTACTCATTGCAAGCAACGTTGGCACAAGATCAATGATGTTGTGTGCAAGTTCACTGGAGCCTACGAAGCTGCAAGCAGGGAGAAAACCAGCGGTGAAAATGAAAATGATGTGCTGAAAAAAGCACATCAAATATTCTTCGCCAACTATAAGAAAAAATTCCTCTTGGAACATGCTTGGAAAGAGCTCAGAAACGACCAGAAGTGGTGTGAGCAATGGTCTGCTAAAAATGAAGGAAACTCTAAAAAAAGAAAGTGTGAGGACGGTGGAGATTCTTCAAGCTCTCAACCATCTGAAACAAAGCGTCCACAGGGTGTTAAAGCCTCAAAGGCAGGTGGTAAGAAGACAGTGGTTGAGGAGAATGAGTCAAAGTTTCAGTCTATGTGGTCTATAAAACAACAGGATCTGGCCATCAAAGAACGGTTGTCTAAGATGAAACTATTGGACAGTCTTCTTGCCAAAAAGGAACCTCTAGCTGAGTGTGAAGAAGCCCTCAAGAACAAACTCATTAATGAGTTGTTGTTGTCTTAGTTTCATGCTCTGTTGTCCTTTAAGTTAAAGTTTCATGTTCTGTTGTCTTTTAAGTTCTAGTTTCATGTTCTGTTGTCTCTGTTTTATGTGGTTCTTGTTGTGTTTTAAGTGCTAAGTTTTAGTTTCTTGTAATGTTTCTGTTATCCTCAAATGTTCAACCCAAGTTTATGATTAAAAGTTGTTATTGTCTTGGTTTAAATGTTCAAGTTTTTGTTGTCTCTGTTTAAATGTTCAAGTTGTTGTTGTCTCTGTTTCTTATGATTAAAAGTTCTTCTTCATCTTGCAGGTTCAATGTGCGATGAGAGCATCTTGCAGGTCACGGGTTTGTTGGGAGCAAAGAAGACAGAGGTCACGGGTTGTAAAACAGAGATTGTCCTTGAAGTCTTGTTGTCTTGTTGTGGTCACGAGTTGTCTTGTTGTGGTCACAGATTGTGTAAAACATGTTGTCTTGTTGTGGTCACGGGTTTGTTGGCACAAAATAGTTGTTGAATTTCGCATCACTCACAATTATAAATTGGTATGTTTCAAGGTTTTGTTGTCTCACCATTCGCTCCATTCTCTTTGCATCTTCTCTCCATTTTCTAAAAGAAAGAAATCTTCTTGTAACCAAGAATCCTCAAACAATTTTCTATCTCTTTTTTATAAAAACAATTCACTTGTAACCAAGAATTCTCAAACAATTTTCTATCTCTTTGGGGGAAAAACAATTCTCTCTTTCTTGATTATCTTTTCAGAAAACTTGGTTATCTTTTCTTTCATTCAAATAAAAATTCTTATCTCATGGCATCTTCTTCTCAAAACACTTTCGACGAGTCAATTGATGAAACTTTCGATCAATATTTTGATCAAGTGTTTGATGAAACATTTGAGCATATGTTCAACGCTCAAGGTGATCAAGAAGATGAAAGGAGGACAAGAAAAAAACGAGTTTTTATCGAACGCAATCGTGAAGAAGGTCATCTCCGGTTATGGAATGATTATTTCAGTGACAGCCCAACATATACTGAAAATCAATTTAGACGACGATTTAGAATGAACAAGGGATTGTTTATGCATATTGTTGATCGCCTCTCCAATGAAGTTCAATTCTTTCGTCAAAAGAAGGACGGTATCGGAAGGCTTGGTCTCTCAACCCTTCAAAAGTGTACAGCAGCTATTCGTGTGTTGGCATATGGTTCTGCGCTTGATGCGGTCGACGAATACCTCAGGCTCGGTGCAACCACTACTCGATTATGTGTGGAAAACTTTGTGGAAGCAATAATAAATTTGTTTGGCGATGAGTACCTAAGGAGACCAACACCGGCTGATCTTCAACGTCTACTTCATATTGGAGAGATTCGTGGATTTCCCGGGATGATAGGAAGCATCGATTGTATGCATTGGGAGTGGAAGAATTGTCCCACCGCTTGGAAAGGGCAATATTCNNNNNNNNNNNNNNNNNNNNNNNNNNNNNNNNNNNNNNNNNNNNTCGTGGTTCGGGAAAACCAACAATCGTTTTAGAGGCGGTTGCTTCATATGATCTCTGGATATGGCATGCGTTTTTTGGACCTCCAGGTACCTTGAATGATATTAATGTTCTTGATCGCTCACCTGTTTTTGATGACATAATAAATGGTCAAGCTCCGCAAGTGAATTTCACGGTCAACGGAAGAGGCTATAATTTGGCTTACTATCTCACCGATGGTATTTATCCGAAATGGGCAACTTTTATCCAATCGATTCGACTTCCACAAGGGCCGAAAGCAGTTTTATTTGCTCAACGTCAAGAAGCTTCCCGAAAAGATGTCGAGCGTGCTTTCGGAGTCTTGCAAGCTCGATTTGCCATTGTTAAAAATCCAGCACTTTCTTGGGATAAAGTCAAGATTGGGAAGATTATGAGAGCATGTATCATACTCCATAATATGATAGTAGAAGACGAACGAGATGAATACATAGACGAACAAGATGAATACACTCAATATGATGTTTCGGATTTCCAACAAGGAGAAGGCAGCGGAAGTTCAAATGTCGATCTCACCTATTATACAGATAGACCTACAAATATCGCCAATCAGATGGGTGTTCGAACAAGAATTCGTGATAGACAAGCGCATCAACAACTGAAAGGTGATTTGGTTGAACATATTTGGCATAAATTTGGACGTGATCAAGACAACAACTGAGCTTGGATGTCTCTTCGAAATTATTATCGTTTATTTCAGTAATCTTTGTTTTTATGTTTGTATTTTAACTTTGTTTTAAAAAAAAAAATTATGTTTAATAATTTTTTAAGAACCCCTAATTAAGATACTACCAATAAACCATTAAAATTAAGAATCTCTTAACAATGTATCTTAACTCTACTTTAATACTAAAAAATCACTAAGAGACCCTAAAAGGGTTCCCCCAATAAACATGCTCTAACCTTATAGCCACCACAATGGTTGTTGATTTTCAGAGTGTAGCTACTAAAGCTCAAGCAGCTGCTCAAGCGAGACAAAGAAGATTAAGTTTAATTAGATCTAAGAAGCGTTTTAGTAGATTCTTATCTTCATACCAATTCAAGTAGGAATGGGGAACTAAAAGAAAAAAACTTTCTGGAAAGATTTTGTTGCTAATATATAGCTTTTATATGCGAATATGCAACCAACATTGCAAGTTTTTCATCTTTTGAATATGAAATAAAAGCTTAAAGGAGAGAAATTATTATTACAAGACAAGGCATTGTGAAGGAAAGAACAAACCAGATAGCAAATACAATAACAAGTAAAAACCATAAGAAAGTAAACTCAAATACTTTTTAAAGACGTTCAACTTGATATGTATATTATATAGTAGTAGTTGGCATAGAGAAAAATCAAGATTCGTAAGCTTCTTCTTCTTCATCCTCGTACTCGCCCTCTTCCTCAGCAGTAGCATCTTGGTATTGCTGGTACTCAGCAACCAGATCATTCATATTACTCTCAGCTTCAGTGAACTCCATCTCGTCCATGCCTTCTCCAGTGTACCAATGCAGAAAAGCCTTACGCCTAAACATAGCCGTGAACTGTTCACTCACTCTCCTAAACATCTCCTGGATCGAGGTCGAGTTTCCCACAAAAGTAGACGCCATTTTGATTCCGGTCGGCGGGATGTCGCAGACGCTGGACTTGACGTTGTTTGGGATCCACTCGACGAAGTAGGAAGAGTTCTTGTTCTGGACGTTCAGGATCTGCTCGTCGACTTCCTTTGTGCTCATCTTTCCTCTGAACATGGCTGAGGCTGTCAGGTAACGTCCGTGGCGAGGATCAGCTGCGCACATCATGTTCTTGGCGTCCCACATTTGCTGGGTGAGCTCGGGGACTGTGAGAGAGATGTACTGTTGGGAGCCACGGGATGTGAGAGGAGCGAAACCGACCATGAAGAAGTGGAGACGAGGGAATGGGATAAGGTTCACGGCGAGTTTCCTTAGGTCGGAGTTGAGTTGTCCAGGGAACCTTAGAGAGCAAGTCACACCACTCATAGTCGCAGAGATCAAATGGTTCAAGTCTCCAACTGCAAATGTTATCATATACGAAAAATATTAGATTTTAGCAATGTTGAAAAAATCGTGAGGGGTAATTAGAAGCGTTTTGCAAAAGACTAATGACTAAGCGGGATATATTTAATGAATTATTGTTTTATTTTTATATATTTAATATTTATTTTTTTAATAATAAAATTATTTTGATGAATTACTAAAAGTAAAAATATATAAAAAGTAGAAGTTAGACAAATTTGTGAATTTACACTAATATTATTGTTTAATTTGGTAAATTTAGATTGAGTTAAACCGATTTAAAAAGGTTCTAACCAATTTCCGCAGAAAACGATTTTTTTTTTAAATGTTTCGGTATAACACTCATTTTAAGAACCAAGTAAATGAACATGAATAGTGGTTGGTGTACTTACAGCTAGGAGTGCTGAGTTTGAGAGTGCGGAAACAGATGTCGTAGAGAGCTTCGTTGTCCAGGACCATGCACTCATCAGCATTCTCAACGAGCTGGTGGACTGAGAGAGTTGCGTTGTAGGGCTCAACGACGGTGTCTGAGACCTTCGGAGATGGGAAAACAGAGAATGTGAGCATCATCCTGTCTGGATATTCCTCACGGATCTTTGATATCAATAGAGTTCCCATTCCAGAACCTGTGCCTCCTCCAAGGGAGTGGCATACTTGAAACCCTGTAATTACAAAATCAAGAAAAATCAGACACGAAATATCATCATGATAGCAGGTGTGAACCAATGTCTAACGCACAACCCCTAGTGTGATATAGCTCGAGGATAACGACTTGATTAAAGATTTGACCAAATGAACTTCAATGTTACATTACAGTGGAGTAAATATGAATAATAATGTAGTTCATAGGAGACCAGATCCAAACAACAATCATGCTGCTATAAGTCGCGACCCTGACCCCACTTGCTCTGTTCAATTATCTCATAAAATGATAGTATGTTCCACCTCGATAGTATCAACACAACAGTAGTAGTGATCAGTTAAAATATTCCATGTGGGTAAGGTTCACGTGATTTACTACAAATCAGGCCACAATACACACTAAAGCCAAATTAAACATTGTCTTTTAGACTAAAAGGAAGTTAACTTTTTCTCCAATATAATTACAATCTAATGACTGCGCTTATATATGTCTGTATTCAGCAATTATGTAATGACTGTAATATTCGATTTGATATATGAAAAGTTTAGCCGACAAAATAAATTGCGGTACAAATCAATAAGTGCTAACAGCTCAGTCTTAACAGTGATTCATAGCTTAATTTAGTTAGTCGGTTTGGGAAAAAAATGAATTATTGAAGAAAAAGCATGGTTAACACTGACAGTAGAGAGACAAAAAAAATTAATTAATCAGAAAACATGCCTAAAATAAGGTTCATGACTCTTAATCCATGCACATAAAACCTGCGATTTAATTCTTCTCTAGATCATGTCTACAGAGAAACGGTGGTCAAATACCAAACAAATACAGTATTACAGATCAATGAAAAAGAAATGTTAATAGATGATGATCAATTATGATTATAATTTTTGTTCTCAAATTAATACTAATTAGTAAATCTCTCTGAAAATCATGAAATAAAAGAAGTTGATACCGATAATATAGAATGTTTCAAATATCGTTTAGTCAAGATCCTCAACACGTGACATGTACGTTGATGATCAAGTGAATAATCCGACACCAATCAATGTTCATATCAAAGCAGAGAACCAATCAGAATCATGCATATACCTTGAAGGCAGTCACAGTTCTCAGCCTCCTTACGAACAACATCAAGAACAGCATCAATAAGCTCAGCACCTTCCGTGTAATGACCTTTCGCCCAGTTATTACCAGCACCCGACTGACCAAACACGAAGTTGTCGGGACGGAATATCTGACCGTACGGTCCCGATCTGATGCTGTCCATGGTACCAGGCTCGAGATCCATGAGAACCGCACGAGGAACGTACCTCCCGCCAGAAGCCTCGTTGTAATAAACATTGATACGCTCGAGCTGGAGATCGGCGGAGTCGCCGTTGTAACGTCCGGTGGGATCAACGCCGTGTTCGTCGCAGATGACTTCCCAGAATTTGGAACCGATTTGGTTCCCGCATTGCCCGCCTTGGACGTGGAGGATCTCTCTCATGGCGTGATTGAAGGAAGATTCTGGTGGAGAGAGATAGACAGAGATGGTGGAGGAAGGAGAGAGAGATGGTGGGGAGGAGAATAAGAAGGAAGCGCGGGAGAGAGAGGGACAAATTGACAGCGTGGGGGAAGATGGATCTTGCCAGCTATAACCGTTACTCTTTCTCTTTATTTGTTTTATTTTATTTTAATAATTATTTGTACCATTTTACATGTTCAGCAAGAACAAATTATAGTTCTATTAAGTTATTTTCTAATAAAATAACAAATTTTAATTGATTTATAAATCTAAATTGGCAACTCTATATTTACATGGATTAGAAGAGTGGCTTGAAAGCTCATTTAAGCCATAAAAGTTCCCTTCCTTTTATACAAAATTCATTGACGGTTGTATTCTTGCAATTTGTAATGCAAGATTATTGTTTTATGAAATGTGAGATTACAAAATTTCTTTGAAAAATTGCATTTTTTCTTCTAAATAAACCAAAATTAATTTCAAATATTTTGTCGCAAAATTAACTTACGTGTATCAGTATTTTTTTGGTCAAAGTGTATCAGTATTTAATTATGCACTCTATCGTCGAAATCAAAATTTTAAATTAATTACTAACAATGTCAAAAAAAAAATTAATTACTAACACCAAAAATCAAGAAAGTATTAACTGAATGATATATATCCCACCAAATTCATTGTGTTATAAAAGGGATTGTGGAATTATTAGAGTAGCTTTTAATAGCCAATGTAATAGCATTTTAAAATTATAATATTAGAATGTAATCTTGTCAATTAAATATTGCATAAACATACAATAATATTTCTAGATAATTAAACTTGTGTTTTGAATCTTGACCCACAATATGAATGTTGTACTAGTGCTACTACTCAAACTTCATTGGAGCTCAACCCCTGTTAATTTTATTTTGATTGAAGGCAACAACAATTGCGAACGATAAAAATAAGTTACATAAATGCATAAACACATGAATGNNNNNNNNNNNNNNNNNNNNNNNNNNNNNNNNNNNNNNNNNTTTTTTTTTTTTTGAAACAACTTGTATTTAGTATCTGAGATGTTTCAGTGACATATCACATGGTAGCAAGAAGTAATAGCGATCTGGAGATCTTGAATACATGTACCTATTTCTATGATAATGATAAAAAACACAATAATTAAGTTTGTTTGAATAATCGTTTATCTTTTGCTCACACACCGATTGTTTTTTGGATCTATCATGCATTATAGACAAGACCAAAAAATTATAGGTAGAAAAATAATAATGTCATAGCACATGTAGACTGTAGTGAGATCTGAGATTTGATCATTTATTTTTATTATTATGTCAAGTTCGATCTACTGTTTTTATGTACGTCAAGATCAATAATTGAACATGTCAATAGGATCCTGTTGTTTTTTACATCTGCTTCCAACCTATTGTAAAAATGTTACTGATGGAGACGTTAACGAAATGGGAGCACGTTAGCTGTATCCATATGAATTCGTATGTATCTAAATTTATTGAAAGAAAAAGTAAACTATAGTTTCATCAACTAGAGTTCTCGTCACAGCCATAACTGGTCCATGCTCAAAACCTAATGGGCTGGGATGGTAAACGTGTGGCCGTATTAGTCACACCCGAACCCCAAAGCCCTAGTCGTCGTCTCTTTATTATAAACCTTTCTTTCTTTGTGAAATTAGATGCGATTTGAATTAGCCGGCGAGTTTTGTCCCTTTTGTTTTCCTTGTTCTAGTTAGGATTCAGATGATGAGATTGGATCTAAAACTCCCACGACGGTGGTCTTATTCTAATGATGTACCGATCCTAACATTCTATGATGATAATACTATTCATCTAGATCCTCCGTTACTTGTAGTACTGTTTTTAGTTTAACTTTGATTTTTAAAATTGTTAGTCCTTCGTATAAACCATCATGAAACTCTTAACATAACACATAAACACATGATTAACTTAATTTATCATTGGAGAGCTATGCCGCAACTGATGAAATTTCCATATTCTGACATTCATCTCTAGATCTTGATCAAGAAACATTGACCTGCTTGCCTTGATTGACCATAGCTATCAGGATTGATGCTCATGAATATCGATAAACCTTAAACCCAAAACACATTTAATTAGTTATAAATTTGTCATACACACTTCGCTGGTTTTAAGTACATAACAGAACTGAGTTGACTTGTACTTTCCGACCTGATCAATGGTGGTTAATCGGGATATTAGATTTAATCTGAATCGATAATGTATAATGACTGAACTGGGTGAGGCTAAAATCTAGGAGCTGTAAATTCCAGTCGATGTCATGAAGTTTAAGTATGTCTTGAGGCTCACGTTTGATATCTTGAGACTATATTTTACAAAAAGGCTTCATTGTGTCCAGGCCCATGAGTTGCTAACTAGAATACCGCCATGAGTTGCTAAAATGAGACCATTGTTAAGTCATATATAACACAGTGATTGGGGGTCACTCGTGCATTTGGGAAATTTATAGACAGCATAACCATCTGTACTCTTATCACTTAATCGATCATGGGCTTATTCGTTATACCAACCCTTAGAGGCCCATTAAGTATTTTATTGTATACTGTATGCGACGGACGATAAGGTAAACAGAAATTGAGTGGCAAGAGGATAGCTTATGATATAGTACAAAGTGCATGTGACATGTCTGATAACAAGATAACAAAGTAACAAAAATGGCCGCTTCGACCCACAAAATAAATAACTTAGTAAGGCAATTATTGAAATTTATATATCTTTTCTATCTTTCACAGTCACACCTCGTTTAAAAACAGTTAAAAGTGACGACATTTTCACACTTGACAACAAAAATATCTAGCAGATACTTATACAGAAACTGTTTTTATTGTTGAACCAACAATTTTTTTTGGTGGAAACCGGGAATATATTGTTGCTCAATCAAGCGCAAAGATATTATATGGATTCTCCAATATAAGCCAGAGGGCCCGCCGCTATCACCAAAGACGTTGAAACATCCACAAGAGTACAATGCCTAGTTGTTCTGAAAATGAAATGATAATTTTATAAATCATATGGTCTTAGACTTTGAAGTTCGTCATCATTTTTGTACTATAAGTCTAGGCACTTGATTATTGTTACTTGCAACGAAAGCACATTATGTATGTTTGTCTCTACTGAGATTCCTGTACGTATAACATAGATAAAGTAATATTGTTTCACACGTCTACGTGGCTATTAATCGTAATTGCGTTATTATTCATTAAAGTTAAAAGAATCTTGAAACATGAATTATCTTACGTTTTGTAGATATTTTATTAAAATTTTGTGCACAAAAAGTAGATATTTTAATTAAAAAGTGGAGGTCAAAAGGTGGTTGAATGTATTCGTTGAAAAACATCAATCAAATACTAACAACACATAAGTGGTAATATAAATCATTTTCAAACCAAACCTCAGACTAATACATTAATTATCGTATAGTTATAATCAACAAATAATTTAGCTCAACATGTCCTTACTAGGTAAAATCTGTTTCGTCTTTGCAACACATTGTGGTAATATAGCCATTTTCAAACCTGTCATTAGACTAACACATTACCGACTAAATAACAAAAGTAGCTCAACATGTCATTTTACCATTATGTAAGATCTTTTTATATGCAGCAGGTGCTTTTCTTAGTCTCAACGGTTAAAACGTCATGATCCAATTACGTGTAGCCACGTAACCAGGCGTTCTATAAGCCACCAAGTACGGTATACACCTGATTGCTACCGACATGATCTATACTAATAAAGTAGGCTTTTTTATCTCCTTGGGGGAAAAAATGCCATGTGGCATTCTTTTTCTAATAATTAAAATATTTTCTCAACTTACATTTAATGGATTTTGGTGTTAATTGTAAAATAAATTGAGTTTTTTAAAACTTTCCTTCATACAAAACCAAGCGTTTATAACAATGTTTTGAATCTGACCCGGATCCGCAGTTGAACCGGTAAATCTGGTGACCCAATGTAAATCGGGTTTGGATTTTGTGAAAATCCGTAAAATACACAAAAACTCACTAAAACACGATACAAGTTGCAGTACTGGTTGAACCAATAGATAATTTGTACTTTTTACTATTTTTAGTTCAAGTTATGTCTTTAATTTTGGTTTATTTTGGATTGGAAGTTTAATTTATTGTTCACATTAGAAATAAATATATATGAATTTTATGTCTTGATTTTTTTAGATGTCATAGCTCTTATGTTACAATTTTTTTTAGATAGTCTAAACTATTTTTTAATATTTTGTATGTACATTTACATACTTAACTTTAGTTTTTATATATTTATTTTCATAGCTAATATATTATTATATGATAAAACTAATTCATTGATCCAATGATCCAGTGATCCGGTAAATCATCCGGTTCAGTGTGCGGATCGGGTTTCAAAACACTGGTTTATAGGATTTCTTTGTATTTTATGTTGACAATGGAGAATATAGCCTCTACGATTCTCCTTCTTTAACGCAATCGACGTAATTGTCCATATAATCAATCCAGAAATCTCACTCATCCCACTATCTCCTATTGAATGCTTCTTTAGTTCCTACTTTCTCAGTTTCATTGGATATGAATAGACGGTGTGAGAGTTCGGAAGATAATCCCTTTACTTTTCTCAATTCAATCATGTCGAATTCCCAGTTTAATTACTCGGAGTCGAAGACAAGCCGGTTTAGAGATGTTGTTCAGACTAGGCTACAACAATTTTGGCAATCCTGAAATGTTAAGAAAGGGATGAGATGTTGGGGTTGATATGTTGTTGATTGATGGGAAGGTACACCGGTTTCTTATTTCAAGTTTCATTGGATATGAATAGACGGTGTGAGAGTTCGGAAGATAATCCTTTTACTTTTCTCAATTCAATCATGTCAAATTCCCAGTTTATTACTCGGAGTTGAAGACAAGCCGGTTTAGAGATGTTGTTCAGACTAGGCTACAACAATTTTTGGCAATCCTGAAATGTTAAGAAAGGGGTGAGATGTTGGGGTTGATATGTTGTCTATTGATGAGAAGGTACACCGGTTTCTTATTTCAGTTTACGAGTTAATGATTGTTTACTTTTAAAGTAGTAGTTTTCTTATGCATCATCTCCTTTCTCAGATGGTGCTTCTACATGCGTCAATCAACTTTCTCAGTTTTAACACCTTTAAACATTTCTTGAAAGAAAACTCAGTCTATGACATGAATATTTAAGATTCCAACTAAATTAACTTTTTTCCGACTATCGATTTTTATTCTATTTTTATTAAATCGATTAGTAACAACTACTTATGCTATTCTATTTTTGAACTAAACAACTTCAAACAAAATAATATACTACGGTCGCTAACCACTATAAAAGATTAGATAAAATATCATTTTTTATATTTTTTCATTTTCATATTTTCAACATATTTTCTTTACTAAGATCATATAATAAATTATAAATCTAAGTAAGAATACAACATACGAACCGGCGCGTAGCGCCGGAATACCACTAGTTCTCTATAAATCATACATCATCTCTTTTTAGCTCTGAGAATCCTTTCTATTATACAAAAAAAAAAAAAGAAGAAGAAGAAGAAACAGAGCAGTATAAAATACTAAAAAAGCTTAACATTAGTTTTCTTCTTCTTCTTCTTCTCAACCCAATGGCTCTAGCGCTGCCATTGATCTTCTTCATGTCCTGGTTATTAACAGGGATTGAATCAACGAGCTTTATCATGGAGAACAAATGCGAATACACAGTCTGGCCTGGACTCCTCTCCAACGCCGGAGTTCCTCCACTTCCGACGACTGGCTTCGTCCTCCAGAAAGGCGAAGAGCGAACCATCACCGCCCCAGCCTCATGGGGTGGACGCTTCTGGGGAAGAACACTCTGCTCCACCGACACCGACGGCAGATTCTCCTGCGCCACAGGAGACTGCGGCTCCGGGACCCTCGAGTGCTCCGGCTCCGGCGCCACCCCGCCCGCCACGCTAGCCGAGTTCACGCTCGACGGATCCGGCGGCCTCGATTTCTACGACGTCAGCCTCGTCGACGGGTACAACGTCCCGATGCTCGTCGTCCCCCAGGGTGGCTCGGGGGCAGAACTGCAGCAGCACGGGCTGCGTCGTGGATCTGAACGGGTCGTGTCCGTCGGAGCTGAGGGTGACGAGCGTCGACGGGGCGAAACAGTCCATGGGATGCAAAAGCGCGTGCGAGGCGTTTCGGACGCCGGAGTATTGCTGCAGCGGCGCGTTCGGGACGCCTGATAAGTGTAAACCGTCGTCGTACTCGTTGATGTTCAAGAACGCGTGTCCACGTGCGTACAGCTACGCTTACGATGATCAGAGCAGTACCTTCACATGCGCTAAGTCCCCTAACTACGTCATCACCTTCTGCCCAGCTCCGAACACCAGGTGAAAATTCCGTTTATGCCCTTAATGTCTCATTACTCATGTGAACATCAGGTTTTGTCGGGGAGGGTATTTCCGTCAAATACAACCGGTAATTTATATTATCACCACTGTTACGCCATTCAGCATGAGTTAGTTGGTACTTCCCTATGATTAAGATTAGGTGCGGAGAGTAGTTTTGTCGAAATAGAACAGACTTCATATTTACTACATTGACATTTAATGAATCATTGAATAACCTTTTGCGTACCTTGCTATCGAAGTCACTTACGTTTTGTTAGATTGTTTTCTTGTCATTTATTGTCACTGTATATTAGAATCTTTTTTTCTTTGGCTTTCTACTGCAAAAAGAAAAGAGATGGGTGTTCACTATGATTTTATTTTATTATATTTTTCCAAAATTATTTTATTTTACCATCGGACATGGGCATCGGGCATAGAGATAGATTTATCGGGATGTGGATACTTAACGGCCCATCAACAAATTTTTAATATTTATCCAAGATCAGGGCGACACGTGGGAAAGCACTAATATATATTGACCTAACGTCTAAGTTTTGTTTTTACCATGGCAGTCAAAAATCATCTCAAGATCAGAGCCCAGACACGAAACCGACGACACCAACAGGGACGTCGACAACTCCAGTCGGAGGAGGTACGACGTGGACGCCGGAAGATACATCGATGATATACGAAGGAGCTTTAGATCAGAGCAAAGCATCACCGTCGTCGTATCATCTTTCGTTATATGGAATCACAGTGACATTTGCGCTGGCGTTTTGTCGGATGTGGTGGCTCTTTTGACCTAACTTTTATTTTGTCTCCCGCGGTAAACACTGAATCAGGACAATGTGGGCCCTCACTAAAGCCTTATCTCTTCCCGGGCCATGTGACCTTTTTTACTTTGAACGACGTCGGAATGAGTCAGAGTTGAAAAAGTTACAGCGAAAAGCTCCTTACCGAAATTACAAAGGATTCTTGTTTTTGGTGAGGAACATGTGGGCAAAGAAAGTGGGCGGCTTGTGGACTTCAAAAAAGACGGGAATCATATTTGTTTAATTTTTAATAAAATTATTAATTAGGATTATAATATAATCTACACTTTATAAAGTAATCTATAAATTTTATTTATTTATTTAAGTAATCTATAAAATTTTAGTTAATATATTGGAGAAATGGGGGAATATTTCATTTGTTTGTTATCTAGTGATCTGCGTTGGCCATTTGAAACATTGAGATGAATAAATACTCTTATTTTATACGTGTTTTGTTATTGTATCAAACAGTTATAATCTGACTTTCTAAGCGCAGATAGCCATAAGAAATTCATCTCTGTTATCAAAGGATAAAAGTATTCATAGAAACATAGAAAGTGATGTGGTAACCACTAGCTGAGGAAGAGATTAAATAGATAATATAGATTAAGGGTGAGTTGATGATGATGAGGTCTTTGATAGCGAGAGTGGGAAATTTGCAAATATAACCAAGTGTCCGTTTTGCTACCTAAGATTTGATAAAATTATCCATTAGTAAAGTCATCCTGGAATATATACACATATTAACCACATGGATGGTAAGATAACAAAAGGATAAGATGTGTCCAGACTAAAGCCCAGTTTCGATTTGCAGAGATCCATTTTAAACCATTGCAAAAGGCCTATGTGAAAGTTGGAAGAATTTTATTCGTAATATCATAGGACATTTTTAAATTAAAAAAAAAAAAAATATCATAGGACATTTATAAACGATTAAAACATGCGTGAATGTCATAAATGACCATGACATGCGTGGCCAGTCTCACGACGTTGCTTCCCCCTTCCACATACGGCGTCATTTACACGAGGGTACCAGCAAACTCTTCTCTCACTTTGTCATCGTCACTCACTAGTCATCGGAGTCCAAGAATCTCTCACCAAGGGTGTTTCCGTAGTTTTCAAAATAACCCCCTTTGGCAAGGATCACGCCTCAGAGTCATCTACCACCACGACATCGCTTTATTTATTAATCTCTCACCGGCGCAGTTGCCACGACGGCGAAAATAAGAAACTCTTTTTATCTCTATGTGTCTAATGGCGTTATCCGACGCGGTTCTCGGTAACCTCGCGGCGATTTACGTGGCGGTTGTTATCGCGATCAAGGTTTACGGAATTGTCTCCGGGACGAGTCTCGGCGCTGGATTCGTCGTTGTAGTTTCGATTACGACGGTCGGAGTTTTGCTTGCGGTCACGCTGGCTTGGGACGTGACTCGCAGGGCGGCGGATGCGGTTTCGCGGTACAATCGCGTAGGCGTTGAGAATCTTAGTCACCTTCAGCATCACCAAGGTGGTGAAATTTGTAAAGGAGGGATTTGCTGGCACGGCGTTGCGGTTCGGTTACCAGCTTCGCAGGTTCGATTCACGCTTCCTCAACACATACCTTACGGTGCTTTCTAAGTATATAACAGCTATTGAGGAATTAGTCATTGGTGTTATTTATTGTAGCTATGAAAATTATGTCTGTGTTTCTTTTTTTAGTGTTATAGCGATGATGAATAATGTTCTTGTTTTTTTTTTTTTGATAAAAAATGATTTTGAATTTAAGAGCTGTGTTAAAGGATTTGAGTGTAATTTGTATGGGCTTGTTGGTGTAGAGAGAACATCCCACATTGTAATGGAGGATCTTGGGTCTATTACCATAATGAACATGTGAATCTATATTTAGATTTCGAGTTGGATATTGGTAGAATCTAGCAGATGAAATTGTTTGTGAGAGGAAAAAGTGGAGTGGATCTCATTTGGATTGGCTCCAGATCATCATCATCATCTTTGATCTAAGACACATGTTTGTCTGCTCTTGTTGATAATACAAATGATGAGTCTGCTTGTAATGAACATATGCTTCTTGGTTTTTATCTCTTTTCATTACTTACGCAACTTTGTCTGAAAGAAGAAATAGCTAAAATTTAAAAGCCATCATCAATAAGCATAAGAATCTCATTACAGACTTAAGAGTAGATAACAAACACATAAGAGGCGATATACAGATCCAACACACAGCAAATATACATACAACTCGGCAATAATTTAAAACGATATATGTCCACTTAAAAAAACAAAAATATTATCATTACACACAACACAATCTTATTTTTTCATTAGCCTTTGATTGGGTCAGAACTCAGAAGGCGATCAAGTTAGTCACGGACTTAGGAGAGTTAGTTAGCATGTGCACAACTTCCCTCATCGTAGGCCTTGACGCGGCCTCATCCTCCACACACATCATCGCTATCTTGAACACATGAATCACACTGGTCAACGGGTAACCAGTCAACCTCGGGTCAACGATCGCAACAACAGTAGCTGCATCCGAAGGCTGAGGTATCTCACCCTCCGTGTTCCTCACCCACCTCACTATATCCACTCCTTCCCCAAACTCACCAACCGGTTTCTTCCCAGCTATCAGCTCCAACAAGACCACTCCAAAGCTATACACATCACTCTTCTCGTCTACTTTGAGAGTGTATGCATACTCTATCAAACCCAAAACAAACAATGATTAGTACATATTTTAGTTGTTAAAGTCACATTCATTTGTGTGTCACAAATCATAGTAATCAGCTATACGACAATAAAACGGACAACAAACTCTAACATATGATTAGTCTCAGTAACATTTAAAGAACATGGCATAGTTTGTTATATAAAACACGTTTAAACTAACCTGGAGCGATGTAACCGTAGGAGCCAGCTATTGAGGACATACACTCAGAAGCAGCACCGTCCACTAAGAACTTAGCAAGCCCAAAATCAGCAACATGGGCTTCGAAATCAGAGTCCAGGAGAATGTTATTGGACTTAACGTCTCTGTGCAAGATCAACGGCGAACAGTCATGGTGAAGATAACACAGTCCTTTCGCCGCTTCAACGGCTACTCTGTGCCTCGTCTCCCACTGAAGATGACCGCCTTTAGACCCGTGCAAAAGCTCGCCGAGGCTCCCGTTAGGCATGTACTCGTATAGAAGCAGGTTCGTGTCCTTGTTCGCCACGTATCCGAGTAGTCTCACTATATGACGGTGGCGGATTCTTCCTAACGTCTGAATCTCCGCCGTGAATCCGTGATCGCTCCTCCCCGTTCCGCGTCCAACTAACCGTTTGATCGCGACGTCTACGTTGTTCGGCATGGATCCGCGGTAGACGATCCCCGCTCCGCCTTTGCCGATTATGTTCTCTTCTTGAAGGCACTCGAGGACGTCTTCCGCCTTGAAATCGAGTCGCTGGAAGGCGGTTAGCTTCCACGAGAGGGATCTCTCGTGTTTCTTCTTGTTCATCTGACGGATCGCGACGCTGATGAGGATCAACGCCGTGATCGCCGCGATGATCGTGATGACGATCCTCGACGGAGAGAACAGCGCCGCGTGGATGCGATCAGAGGTTTGTCCTGGACGCGTTAGGCAGGAGGCGTGGTGAGGGAGGCAGAGGTAAGGGTTTCCGGCGAAGGAAGTGTCGTTGAAGACTAGGAACTGACCGCCGAGTGGTACTCTCCCGGAGAGGTCGTTGAAGGAGAGATCGAGAGTGGTTAAGCTCGTCATCTTCCCGATTCCGATCGGGATCGAGCCGGTGAGCTGATTCCCGGAGAGATTGAGAGTTCCTAAGTTAATCACGTCGTGGATGTCTTTCGGGATATCTCCGCCGATTCTGTTACGGCTGAGATCGACGGAGATTAAGGAAGTGCATCGCGAGAATGAGTCAGGGATATCGCCGGTTAGGTTGTTAGCGCTCGTGTTGATCTTCGTGAGATGCTTCAGCTCGAAAACTTCTCTCGGAATATTCCCGCTAAACCGGTTCCGGTCTAAGAACAGATCCTGTAGATTTTTGAAATTACCGATAGCCGGAGGGATTAAACCGGTAAACCAGTTGTTAGATAGGTAGATATGATCGAGAACGTCGCCGGACATCTCCGTAGGAAGTTCCCCGGAGAAGAAGTTATCCGCGAGCTCGATGATTGTAACGAGTGGTAAGTTGAATAGTCCCGCCGGAACGGTCCCGTTGAGGAGATTCTTGACGATTCTGATCTTGTTTAGAGATTTGCATTGACCTAGCTTCTCCGGGATGGAGCCGAAGAAGAAGTTGTTGGAGAGTACCAGAACCTCCAGCTTCCCACCTCTGCATAAATCCACGGGGATGAGTCCGGTGAGATGGTTTTCAGAGACGTCGAGCTTTTTCAGATTCCCGTTCCGGCCGATATTCGCCGGTAACTCTAACGTGAAGTTGTTCTCCCACACCTGGAAGACTTGGAGGTTCGGCATTTCTCCGATGAACTCCGGTATCGGCCCGTGGAGGTTGTTTCTGAAGAGGTTGATGAGAGTGATGTTTCCCAGAGAGATGAAGCTCTGAGGAATCTCTCCGGTTAGCTGGTTAATTGATAGATCGAGAGATTTTAAGCTGATTAAACCGGAGAGTTCTGGTGGGATGTTTCCGGTTAAGTTGTTGATGTGGAGGAACAGCGTGTGCAAGTGTTTCAGATTACTCAGTGTCGTCGGAATCTCTCCGGTGAGAGTACAGCTCGCCATGTCGAGGATTTCTAGCTTCGTTAATTCGCCGAACTCCGGCGGAACGCCGCCGGTGTAGCTGTTGAAGTAGCCGACGTACATTTCTCTGAGATTCTTGAGACGTGACAAGAACGCCGGAGATTCGCCGGAGAGTCCGGCTCCGTTGAGGCCGAGATACTCCAAGCTTTGGATATCTCCGTAACTCTCGGGAATCTCTCCGGTGAAGAAGTTTCCTCCGAGAGAGAGGTGTTTGAGCTTGTTCAGCCCGGGAATCTCCGGCGGTAACGGCCCAGTGAAGTTGTTGTTATAGGCGTCGAGGACTTCGAGGTCGACCATGGAAGTGAGAATCTCTCCGGGGAAGGTTCCGTTGAGGTTTACGTTGTTGGAGATGTTGAGAACCTTTAGTGAAGTGAGACTCTTCATCTCCAGCGGCAACCTACCGGAGAAGTTATTCGCGGCTAACGTCAGATTCACCAAACGGTTCAGCATCCCAATCTCCGGTGAGATGGTTCCGAACAGAGGAGTGAAAGAGACGTTGAGTGAGACAACACGAGCGTCGGCGTCGCATGAGACGCCGGAGAAAGAACAGTGAGCCGTGGGAGAAGTGGAGTGAACCCAGTCGTGGAGACCGTGTCCGTTGTGACCAGTCATTGAGGATTTGAGGTTGAGGAGATGGTCCATGTCAGTGGAAGCGAAGCATGGTGAGAAACATAGAAGCAAAATCGAGATAACGTAGTGAAGATGCAGAAACAGAAGGTGTGTTTTCAAAAGTCTCATCTCCATTTTTTCTTTCTTTCTTCAAAGCTGTCTTCTCAGAGAAAGAGAGAGAGAATAAGAAGTTAGCGTGAGTTTTATGCGATAGGATGAGTGCACAGATATATATGAACAATCACAATGGCATATTATGAAGATGAAGAAGAGATTCGTAGCAGTACTGTTCTAAAATACCTCTAGACTTTCAGAGAGAATATTATTGCTTTTTTAATCTTTTTTTATATGACCTTGAATGAGAATAAATATACCACTAGAAATATTGCGCTTTAAAAACCCTTTTTTTTTCTCCAATGAATCTCGTAATTACTGGAAAAAAAATTGTGATGGTTACTCTCTATCGAGGTAGATTTATGAAATTATCATACGGATATAAAGTTATAAATGGTTAAAAGGACATTCAGCTTCTAGATATGAACTACGACTAGCAGTCAACATTCATGTGTCAATCGTTCATTGTCTTTTTATATCTCGCATTCTATTTTTTCCCATGTTCTAAAACTCGACCGCCTAGCTGATTTAGCGGCTGAGAAATCGCTATACAGTGAGCAACCGTGGCGAATTGGAGCTATGCGCTTAGAAAATCAGATTCAAAAAAAAAACAACAACATTTTTTACTTATTTGAACCTGTAAATCTAATAATCTACTTTAAATTTGTGAAATTTTGACAAAAACAACAAGAAAAACAAAGAAAATTGGTTTAGAACCCGTCAAAATTTCTTAGCCATCGCGTTTTTACAATTGCAGAGATCTAAAAACCCAAATAGGAAGATGATATATAAATAACGGTTTTACCTTTCATTAAAGAAAAATATAAAAAACTATTAAAGTGCACATCCTAGCACTTGAACTCGGAATCATGTGTTAAATGAAAGAACACTTACCATTGCACCACACTAATTTTATGGTTTATGATAGAGATATATTATAAACAACATAAATAAGATCACCGATTAATTTCCGAGTAATCGTTGATTAATTGATTAGGTGCTAGGCGTTATCCGATCGCACGCGTAGTGCCTACCGAGTTTCCGAACATGGATTTTTTCTAATATTTTTCTTTGAATTATTACAGTTACTAGTAAATTTTAAACCTGAAACTACGATATTTAATATCAGTTTCGTGTCAAATTTCATTTAATCATGTGAATTCTAAACCATATCTATGATTTTTTTCTCGGCTAACTAACGTTTAGTGCTCAAATAACATTTGCTGGAATTGAACCGCAAACCCAAAAATCCACCACTGTATTGTGCTTTAAAGAAACTTTTATCATAGTCTTTTTTACAGTTTATATACAAAAGTTATTTGGTTAGTAGCCGCCACTACACTAGACATGGATAACCGGGAAACGTCGTCGTCGTCTTCTTTCCTCTGTTAAATCTTCTGACACGATTTAAATCGAGAAAGTTTATCTTTTTGGCTATATATATCAAAATAAGTGGCTATCTTCACGGCGTTTAAAATGTAACGGGAGAAAGAAAACGCTACTACAGTAATGTCTATGATCTGAACTGACCGTTATAGACGATAGACAGGACTTAACTTTACGTTCCCTGGCTATAGCTGCTGTGGTTTCTTTCTTTTTATCGCCCTCTTGTCATCTCAACCAGTTTAATCCGACGACATTTTGTTTTCATCGTCACGTGATTAGGAAAATGGAAAATGTATTTTAAAAACTTAAAAAGACAAAGAGGGGAGTTAATGGGCTTATTAAGTAGGGCCTAGTTATTAAATGGCCCGTAGAGAAAGTTTGTCTGTGTCCTTTCCAAAAGAGACAAATCTGCCCCTTCTATTTTGGCTTCCATCTAATAGTCAACGCTCCTTTTCTTGTCTTTGTCTACACTCACTCTTCTTGTAGACCTTCTTCTGCTCTGCTTCAGGGTCCCCACGTTCTATGTTATTTACCTTTGGTTTTGCAAAACTGTCTGACTTTACCCTTATCTTCTCTCTTGTGACATTTTAAAAGTTATGTTTTTGTTTTTTTTTGCTAAGATTATGGTGTTTTTACCTTCTGAAAATCGTTGATTTGATTACAAGCTCGAGTTAACTTCGGAATATGTGGGAAATTTTCGAATATTCTTACAGTTTTGGTTGTTAACAGTGGCTAATCATAAATAAAATCGTAGTGGAAACTCTCGAATATCACACTTAGATCTTTGTAAGAAAAAAAATCTGTCGTTAGTTTAGAATTTCTGAAAGTTCTAAGCTACTTTGACATAGCTAGTCTATCAATGGCCACACACTGAAATGAGTTGATGAATCAACAACTAAAGATTGTTAGTGTTTAGGATCGCGATTACTATTCGTGTGACGGTAGCAAAACACAGAACAAAATATCTATTGGATCACCACTATAGCATAGATAAACTGGATGGCAAAATGTGTATCTTACTTTAATATTATCACTATCGATCGTTGATTCACATTTTCAAGTATTTCCATTTATTTTTCACAGTTTTAAAATATTTTAAGAACTTTACAATTTTGATCCGCAAGATATCCAGTTTATTTCCAATATTTTTCTTAGTTATAAAAGCCATGCTATAGTTTAAACAAGTTTTATGCAGAATCGATTCTGCCAGAATTAAAATGTATTTCTCTCAACATTTTGTAACATAAAAGCTAATGATATATGTAAATTATTGGTTTCCGGTTATACAAGCACTAGGCTTTTGGCGAATATATCTTAACCCCGCTAACAATATTTTATACGATTAAATATCAGGAGGAGCTTTACCAACAAAGGGCCTTTGATTTGTTGAGAAATCTATACACTTGGGGGTTGACATAGACACACAAAGACACTGGGGGACCAATGTTTTAAAAATATGCGGTATACTGGTATAAAGGCCCTTTCCTAAATGGCGCAGCGAATTTCTCATTTTATAGGATGTTGTGTATTCTCTTTTGGATTTGATCTTGATGCATTAGATTTAAGAAAGTAAACGCCATACATACCTAACAACATATAAATATATGCTTTCACGTGTTATGTTTGGTTAATACTGTTAACCGGTTCAAGTGGATTGGACGGTTTGCACAATCAGAGCCACCGGTACGTATTGAACAAAGGGAGATGTCAATATGTGATGAAGAAGAAAAGCATCTTTTCTGTTATTCGCTGAAAATGCCCAAACTTGTGAACTTTGGGGAATAAAGGTTTATGTTCTCTTCAGCAGAAAAAGAGCTTTACTTAGTAATTAGAAATTTTCTTTATGTGGAGAGGAATATATGAACTTTGGGCATTTGCTGATATAATATGACAATACACTTAGGAGCATAATGTAAGACATTTATCATTAATGATTTATGGTGAATGTCTGAATGAACTTTAGGGCTAGCTTCTAGATGAATGCTTATAAATAAATTTGCGAAACTAATTATAGGAAAGAGAAACATAATTAGTAAAACTGTAGAAGCTTGATTCACATTTTCGAAAGAATTCTGGAGTTGCACTTCCAAAGTTCCAATGGTACAATTTGCATCATATGCTACTTTCACTTGTTACTTGAAAGTTAATTACTGTTTAAAAAGTTTGTCTTAAGACAGTTAATGTTTTAAATCTATCACACATTCTAAACATTATATACATACATACATGTATACTTTATTTTATTTGCACTTATTTAATTTGTTTTATTTTTAATAAAATAATTTCGACTCATTTTTTATATTTTAACATATATATATAGTTTCAATTAAATACACTAATTAAGATATAAGACATTAACCTTTTAAATACAAAAAAATAAAAAACATATAATTAAAATGGGGGTATAGTCATTCAAAAATTTAAAAATAAAAGCTTTACAATAGAAACTTTACATACGGATATTGCCCCGGCAAAATATGTTTTTATTTCAATTCTCAAAAAAAAAAAATGCTTTCTTACGCATACACACTCAACTAACCAAAAACTATCGTTCAGTATTATAAGAACGGCATCTTCATAAATTTAGAGGAGATTTATTGAATGTCATGAACGGTGAGGTGGGGCAATCAATGTGTTATTGTACTCCAAAACCTAGTAAACGTTTTTGGGAGTAAACATCTAGAAAATGATACATGATTCGTTCGTGATAAGCTTGTTTGTTTCACATATAATCAACGCACTAATCATTTTGGGATAAGCCTTTCAAAAGCAATCATTAAAAGAAACAAATAGAGAGCAATGATAATTGTATAAGACATATAGGTCATAGTCTTGTCAATCAAGTTAATAATTAAACGGAAAGGCTACAGAAGTAGCTAGTAGAAGACAATGATGAAAACGACGACAAAGTTGTGTCACAAGTGTGTCTTACACTACCTGTCTTGGAAACTGGGGAATGCCTAATGGCCTTTTGAGCTTTTGCTTCAGTCACTACCGATTTTTCCCTGTCCATATTGGCAGCTTTAGTCCCAAATCTATTCAACACATGCAATGATTTTTGACGAAGAAACTTCTCAATCCATGACCCTTCACATTTCACTTTTTGTTCGATTGCACGGCTGATATTTAAATATATAATTATTTATTCTATACATTATGAAGAGGCAATGCAAGTATCTATAAAAGCATTTCTAACCTATTGATATTTTTATCTCTATAATAGTATTTAAAAGTAAATTTACTTTAACCCACTTTTATTTTTTATGTGCAATAGAAATTGTTATTTTTTCATCTAATTATAGAGGAAGAAATAACATTTTTTCTATTTTTACTCTATATTTTAAAATTATTATTTTAGGAAAAAGACATTAGAATATATCACACATCTATTATAGAGTTTTTATATTTTAAAGGTAAAAATAGATAAATACATTTGGAGATTTTCAAGTAGCAACTAAGATATTATCCGTGTTGAAATGTAGATTATGTTTTTTATACATTTAAAAATAAATTAATTTTATTCAATATGCATTATAATATTTTTATAGAATCGAGAAACTCAATTATTTAAATTCATAAAATTGGCAAACCATTTTTATTTGTAAATATCTTAACTTTAAATTTTAATTATGTTGAATATAAATTCAAAATTTTATATAAATAGCTAGATATAAGAATTTAAAAATATACTTTCAAAATTTGTTTGTTTTGATAGAGAAATATTATCCTCTTATTTGAATTGAGAAATATTAGGATAATGAATAACAATCTAAATAATTTAATATTAGTTTAAAACTTAAATTTTTTTATTGGCTATTATTCTCATATTTTTTTGGACTTATATTATTAGTGTTACTAATTTTAGTTGGACCATCGTAACTTTTTTGATAGTCACAAAAAACTATCTATAAAAAAAATTCTATTTTAATAGAGTAGATGTAGTTTCTATTGTATATAATTAAGTTATGTTTTATTTACAACGTTGATATATCAAAAAGTGGAAACTCTGGTTTTATTATACCAGAGGGAAAATAACTCTAGTCTCAGGAGTTATTAACTTATTAATCAAAGAAGTTTAATTAATGTGCGGCTAAGATAGTAGTGGGTCTATTAGCCTGAGGTTCGAGTGGTGAAGATTTTCGTATGGACCTAATCCATTTTATCGAATATGAAACTCCTCCCCTGTTGGCTTTGTTTTGGTCACGTCTTTCGACAAGAATAACACGATTTGCTCTGGTCAAAGCACAAGGCTCTCCATATATGTTACCACTCAAGTTAAATTTACTGGGCCGTCGGGCCTCGAGTCTTGTTAGTAATCAAGATTCATATTTCTAGTTTGAATCCGTGAACCCAATACAAACCCATCAACTAGGACAAGCTATGTCGCCAGGTGCATCAGATAGCTAGTTTGATATAGATTTGAACCAACGATGAAAAGTATCTGTCGGAAATATGCTTAGAACTTCTCCAGGAGAACCACGACAAATAAAGATCATTAAGTCCATAACCAACTACATCTCCGTTTTGATAAGAACGCTGCTTACCTAGCTAAGTCAACTCTAAAATATTAAAATAAGAAAAAATATGGTGTATTTAGTTGACATTTTGGTATTACCATTCAGCAATGAGCATTGATAAATTTTGTCATCCATATGTGTAATGATCTCCAACAAGACTATACATTGTCTAATTAAATCACCAGGGATATGAAAATCATCTTTTTTGGTTAGGGTTTGCTCTGGTACCATATATTGTTATTGTATTGAACAGAAGTGTCCAGGGTTATATATTGACAGAGAGGGACATAATTAATCAAATATATGCAAAACATATGAATACATGATAGAATGAATCTACTAATCTAGGATGAATATTATACAGAGTACCCTAGCACTGGGTTTCAGAATATTGAAATATCAATCGAAAGTATTTTATTACCCGTTTTCACTTTTCTATAAACAGGGAACTCAATGCAAATTCATGAAACCCAAGAACAAGATCCAGCCTGGTGAAGATCTTATCTTCTTGATAAAATCCAATTAATCAGCACTAGCTCAGCCAAACCAGCCCCTTAGGTTATATTACATAGAGCAGTTAGTCAAGATAGGCGGTGGAGAAATCAGCCATAAACCATTACTAACGTACTCTTGCAAATTAATAACACGATGACAGGTTCACCATACCATTGCACTATAAATAGAGATTGATCGTAAGCTTATTTCCTCACACACAACACATACTTCTATTGAAAAACAAGGCAATATACTACTCATGTCTAAGTTAGCTGCCATCATCACCCTTCTCTTCGCTTACTTTGTTCTCTTTACCGCTTTTGGTGAGTAATGATCGTACCATATGCATGTCAAATGTATTTAAAATGTTCCTTTCGTTTATTTTGATTAGTTAATTAACCTCAAGGGACAAATATATGTATACAGAATCACCAGCAGTGGTGGAAGGACAGAGGTTGTGCACGAGGCATAGTAGGACATGGTGGGGAGTTTGTGGAAATAACAATGCATGTAAGAATCAGTGCATGCGACTCGAGGGAGCAAGACACGGATCTTGTAACTATGTCTTCCCATATCACAGATGTGTCTGTTACTTCCCATGTTGATTTTCCAAAATGTCTTTGGTGTTTGTATGTCTATTTTACATGAATAAGTCTGTGTCACTCTAGTGGCCATAAATAAGTCTATAATAAGAGTTTCTTCAATGGAAGTTTCTCAATAAGTTTTTCATATTAAAAGCTAATAAAATAACATTTTGAAAGGAAAGATTGACAAGAGTTTTTAAATTTTAAACAATTTGAAGGTGTATTCAATTTTAAGATTCAAAGCGATTTTAGGTAAAATTTTAGATGAATTTAAATTTATGAATAACTTACTATTTTTTTTTTGTTAAACAACTATAGAATCTCATCTAAAACAGTGAGATTTAGATTTTTATATTTATTTATAATGAAGAAACTACACTAAAACACTCTTAGAAAATTCGTAATTTAAAATATTTTGAAACAATTGATTTAAAATACTTTATCAAATAACAAATTCAATAACACTATGAATTATTTTTCAAAAATTCATTTTTGAATAATAATAAATTTGTCATTTTACTTCAAACTCATTTGAATACATTATTCTTATATAATTGCTTCAAATTTTTATAGTATAAAATTAGTTATTTTTAATTCAATTATGTAAGTAAATAATATCAAAATATTAATATTTTATTTATGTGAAACTTAATGGAAAATTTCGTGCTCTTATGACATGTATGTGTTATATTTCAATGTTTGAATGGGTTACAAACTTATAATATATGATAGAGCGTTCTTTAGCATTTTAATTTAGCACGTACAATTGAAACATTTTGAATAATGCTTAAAATTTCTTAAATTTTTTTTTTCCACTTATTATAAGCATATTGATTTCCAGTATGGCATGTAAGGGTTAGGACAATGTTAACAAACATATGATTACAGCTTAAAAAATTGGTGATCGTTTTTACAGCTTGAAAAATTATATTAAAAAAAGTGGACACTAATAATATAATGAGAGGAAAAATTGCTCATAAGGATGATAAGAAACCTTAGCTAATTATAGCATTCTCTTCTAGCTGAAAGGATTTTTCCTTCGCCTGCAATACAATTACAAGTTGATTCTACGTAATTCTTGAGTCAAAGCTCTATGCCTCTAACAAAATGGAAACTCTTGTTCTATTCTAAGGAGAAAAAGACTCTACTGCACATTATTGATGTATCAATGAAGAATGGCTATAACTTGTATCAATTTCAAATAATGGCATATATTTCGATTCTATTCGTTGTTTTCAAAATAGTGGAATGTTAGTGGATTAGTCTTGAACAATACCGTAGTAACTCCTCATGCAACGAGCTTTGAATATATCTGACGAACCACTAAAATTGTGTAACTATGACTTGGTCAAGATCTTAATTTCTTGAGTCTTAACAAAGTCAAATTAACCAGCCGCCCTTGTGAACGATGTAGCATTAGCTCAAAGCCAAAGCAGCCGCCTGAGTTACTCTAGATAGAGACTTATAGAGACAGCCTTCCATATGTAAACGATATGTGGTGGAGAAACCGGCCATCAACTTTGAATATAGGGCCAGTCTTATCTTCCATATCGTAATTAACTAGAAACAATATATGCTTAACTTGCTGGGTTACAACGTTATTGTGCGATGAACAAGTAAAGATTAACCTACTAGCATTTCACTATAAATAGAGGATGATCTTAGGCTTATTTCTTCACACTTCACACACATACATCAAAACAAATTACTAATAGTCATGGCTAAGTTTGTTTCCATCATCGCCCTTCTCTTCGCTGCTCTTGTTCTCTTTGCTGCTTTTGGTGAGTAATGATCATCAGCATGGCGAAATGAATTTTGGCGAAATGAATTTTAATTTTTTTTTTCAGATTATTTGATTTAACCATGATAAATATAAATATACAGAAGCCCCAACAATGGTGGAAGCGCAGAAGTTGTGCCAGAAGTCTAGTGGGACATGGTCAGGAGTATGTGGAAATAACAATGNNNNNNNNNNNNNNNNNNNNNNNNNNNNNNNNNNNNNNNNNNNNTGCATCAACCTTGAGGGAGCACGACATGGATCTTGCAACTATGTTGTCCCATATCACAGGTGTATCTGTTACTTCCCATGTTAATCTACCAAAACTTATTGGTCTACCAAAACTCATGGGTCTATCAAAATCTGTTGTGCTTGTCCCTACAAGAAAACAGGCGTATACCGACGGACAAAATCGTCAGAAATTCGTCAGAATAGACATATTTTGACGAAAGTGGTCGTCGGTATCATTTGGTCGGAAAAAAAAATTCGTCGGAATTTCTGACGAAATTCCGACGACTTTCTGACGAATACCGAGAAACAACATTCTGACGAACTTCCGACGACACTCCGACAACATCTTCCGAAAACAGAGTTCATCGGAAACACTATATTCCGACGAACCCTTGTTTCGTTGGTATATGCCGACGACCAAGATTCGTCGGAATAAACCGACGAACCAATGGTTTGTCGGAATTATGCAATTTTTAAATATGAATAAATTTTACTTTTTTAAATATTTTTATATTAAAAATTAATAAATAAATAAAATCTAAAATTTAAAAATAATAATATTATAGAATTTAAATTCATACAAACTGAAATAGAAAAAAAAACATTCAGAAAGTTTTAAAATTCATAAAAAAACTAAGAAGAACCCGAAGGAAACAAACGATCTAGCCTCTCTAATATCTCGGCGTTGGTCTTCTTTTGATCCGCCAGCTCAGCAATGATAGTGGCGTTCTGCTCCTCCAATGCCACAATCCGTTCATCTTTGTTCTGTAGTTGCTCCAGAATCATGGGATCGGCATACGGAACTTGCGAAGAAGACGCCGGAAACGAAGAAGTACAGCGGGCCAACCCAACTAAACGGCCTCCTTTCCTTTTGGGAACCGCCTACAAAAATATTTAAAGTTAGTAAACTACATTATAATAAAAAAATTACCTTTTCGACCATCTCATCTATTTGCAATCGGGACAAGTTGGTTGAAGCTCCCGTAGAATCGCCGTCATCAGAGAGAGGCTGAGAAGCTAGAAAAACTTCCTTTTGAGTTTCCACCAACTCAACGACCTCTCTGATCACGGTGTCCTGAATTCGACCCGTCTTCTTGTTAGTTTGAGCCTCCTTAATGAGTTGGAGACGGTCAACGGGATTACCGTCGTTTGCTTCAATCTAAAAAAACAGTAATTAGCCATGGAATATATAAAAATATTTATTTAAATAAATATAAATATAAATTAAAATAAAAGAACTTACAAGTTGATCCTCCTTAGAAGACATAGAGCAAGCGCCGAGGTTGTGCACATACATACCTTTCCCGCCACGATCGCTCTTCCGGTTCTTGGAGTTCTTACGAGACGTCGCTGCAGTGTCGTCCTTCTCCCAATGGACTATCAACTGCTCCCACACCGTGGCCTCTTGTTTTTCTGCCATACTGTATTCCACGCGTTGATCTGCTTCGTATAAGAGTCCATGGCCTTTTCGTTGAATTTCTGACGGACTGTTTCCGTGAGACCTAACTCCCAAGTGAATTGTTGCTATTAAAAACACTTAATTTAATAAGTAGATATATATAAAAAATTTGAAAAATGCTTTAAAAAAACTGAAGAGTTACCGCAAACTGACGAAACCACAGCTTCCGTTCGCCGCGAGGGATCACACTCCACTTCGAATATCCGGTAGGGAGCATGTAATACATCATATTATTGATGCTCCTGCTAATGCCATTGCTCGACTTGTCGAACCTTTAAAAAATGCATGTTAGAAAGTTAATAAATGGAAAAAACATAATGCTACAAATAAAATAAAAATACTAACCAAGTTGTATGTCCCGGTCGTGGGTTGGGATGGAGAACCGGGAGATGCTCTCGACCTGATTGCAATAGTTCAACTGACATGACCCCCGGATCCTGCTGAGCAGCAGCGGGATCGGCAGCTGGAGCGGGAACATATGCGGGAACCGAGTTTTGTTCGTGAGACGATCCCGATGCACGGGAACTGCTCACCGAACTACCTCGGCCGCCACGAAATGCGGAACGACGGGCTGCGGAACGACGTGCATCCTCGGATCTAAGAAAACAAATTTTAATACATCAAAATCTGAAAATGATTTATATATGTATGTATAAAAAATTATAAATGTTTTTAAAAAACTATAAATATAGAAAAGGTTGTTAAATATATTAAAAATGATTTTTACATCTAAAAATGATTTTTATATATATGTATGTATAAAAATTATAAATATTTTCAAAAATGTTTATAAATATAGAAACTTATTTTCAAAAATATAGAAAATTAATTTCAAAAATGTTTATAAATATAGAAAATTATAAATACAGTTTGTTAAATATATAAAAAATTATGTACGTTTTTATCTATATAAAAAAATATAAATCGTTTTTTCTATATAAAAATATTTATAAAACGTTTATAAATATAGAAAAGGTTGTTAAATATATTTATAAAAATTTAAAAACTTTTTATTATATATTATATATTACTGGAAATCCCAAAAACATTTTTAAAAATCAAAAAACGTTTTAAAAATACAAAAACGTTATAAATTTTAACAAATACACAATATAATTCCACAAAAAAGTTAAAATAACAATCCAAACAATAATCCTAAGTAATAGATCACAAACTATCCATTCAATCAATCAAACAACCTCAAATCCTAAGATCTAACTTCCTAAACCCTAAAAAATTGAAAAATACAAAGGTTTTATAGAAAATCTTACATGATTTGTGGTGTGGGGGAAGGGATCTGAGATTGGGAAGAGGAGTCGCCAGAGATGGGAGATTGGATATCGCCGGAGTCGCCGGAGGTCGCAAATCGCAGGAGAGCTTGTAGAGAGAGAGGCAGAAATGACGAAGAAGGAAGAAGAAGGGCTTCTATCTTTTAGGATTCCGACGGACCCGTGTTCGTCGGAATTCTGTCGGAATCATTAAATATATACCAATTGGCGGTTCGCGAAAATTTTCACGCGGTTTGGTTTTCCCGGGTAATTTGATATTCCGACGGAATTAGTAATGTCGTCGGAATTCCGTCGGAATTTGAATCCGTCGGAAAGTCGTCAGTTTTTTCTGACGGATTACCGACGAATTATTCCTACGGAATTCCGATGACTTAGTGTTTAGGGGGCAAAATCCAAATCTTTGATAATATATATAGTATTTGCATAATGATTTAATTATAAAAATGTATTCACAACGTGATTTTACACAAAAACATTTTTGTAGTGTAAGATTAGATGAATATGATTGTATAAGTATATGAGTGTTTAGATGAGATGTTATGAAAACAATGATATACATGCATAAATATTTTAATGAGTTGCTATATTTGAACGATTGCGATCTAATACTATATTAAACACTTAATATGTGTTATTTTCATAGTTTTGGTATCAAAATTTATATAATTTTTGATATGAAATTTTATAAAAATATAAGTTCTCGGAAATTCCTCGGAATATACCGAGGAAATTCCGACGAACTTGCTTAGTTCGTCGGAAGGTCCTCGGTATATTCCGAGGAATTTCCGAGGAACTTGTCCACTTTCAGTGAAACCACAGTTCGTCGCTATGTCGTCGGTATATACTGACGAAATACCGACAGACTTGTTTTTTTTTTTTATAAAAAATACAACTTATGTAAGATGTACAACAACTAATAAATACAAGTCAAATAATAAGAATTCCTCATCAAAATCTTCAGAATCTTCATCAAACTCCCCAACCTCAACTTCTGACTCTGAATGTACAACAACATCAAGTCCAAATTCGGTAAAATTCTCAACGAGTTCAACATCTTCCAAATCTTCAACTGCACTCACGTTGGTGGTAGAGTCTGGTTGTAATGGTTCATTATCAGAAGTTCCATCCACTCGTCCTCTTGGGTTGATTTGCGTTACAGTGACCCATGGATCGTCTCTGTACGTCACCCGAGGGTAACTGATGTAGCACACCTATCAATTATACAACGTATTAGTATTTGATAAACCATTAATTATATAAGAAATTGACATACCTGATCAGCTTGCGAAGCAAGAATGAAGGGATCATAATATTGAAGTTTCCGCCGCGAATGAACCGATGTAACAACAAACGCATCAGTCTTCACTCCTCTATCTGGGGTGGTGTCATACCAATCACAATAGAATACTACACACCGCAATCCAACCATACCAGGAAACTGGATTTCCAATATTTCTTCTATGTTGCCGTAATAGACATCGTCACCGGAACAAGACGAAACACCAGCATCATATGTTGTCCTAGAATGACCTTTCCTTGTGAATGCATATCCTCGTGTACAATATTTTGGATATGATCTGACCACATAGTTTGGTCCCCGCATAAATTCGCGTATCCAATCATCGAACACAAAACCTCTGGCCAAACCATCATTCACCTATAAATTAAAAACATATATGAGTGAAAAGTTAATTACTTTATATTAATTATAAACTAATCATATGCATATATAGGGTAATATGATATATGACTCACATAACTAAGAAGCAATGCAGCAAATCCGTTATGTCTGAGTTGTTGAAGCTCATCTTCTGTCGCATGCCTGTGAGTCATACGCAACTCTGCCATATAAACACTATACAAAAAGATATTATCAATATGAAATAAAATTATTGAATATCAATTTAACAATTTAAATGAATAAATATTTGTACCTCTCCGTAAGTCTCTGCTTCGTGGGTTTTCCACTAAGTCGTCCTATTTCCTTGAACATGCTTGGGACAGTAACATGATATGTTGCTCTCTCCCCTCTGTCATCATGCCGAGCAGGTCTTCGGTTTTTTGTATGCACTTCTGATGGAAAATAGATTTCAGCAAAGATTGCAGTTTCTTCATTGATCGCCTGTGCGACTATAGATCCTTCCACCTTGCTTAAATTTTTGACCTTCTTCTTCAGATGATGCATATAACGCTCAAAAAGATACATCCATCGGTACTGCACATGACCACCAAGTTCCAATACTTTTGCGAGATGAATGGCAAGATGTTCCATTACATCAAAGAATGATGGAGGAAATATCATCTCGAGGTTGCACATGCTGACTGGTATGTTTGCCTTCAGATTACTAATACCCTCTTCAGTCACTGCTATGGTGCATAAATCGCGGAAAAATACTTATCCCTGCATAGATAAGAAGACAAAATTTTCTTAAGTATTTGGATGATATAAGCATAATTGTTAAATATAAACAGTTAAAATGTAAAAATATAAGATACCTGCAATTGCTTCATGAACATTACGTGGCAATAGTGCGGAAAAAGCAAACAGAAGGAGGCGCTGCATAATTACATGACAATCATGACTCTTCAAACCAGTAAACTTTCCTTCGCTTCTATCAACGCAGTTCCACAAATTTGATGCATAACCGTCTGGAAATTGCACTCTATCTGTAATCCAATCAAAGAACTCTTCTTTTCCAGCACCATCCAGCCGATAAATGGGAAAAAGGGACCGTACTTTTCTCATCAACGTGAAGTTTAGAACGATCACATATATCGACTAAATCCAACCTTGACTTCAAATTACCTGGCCACTCCCCCGGTTCTCGCAAAACCAGTAGAAGGGGAGCCTCTGTTCCTATACATCGCCGTTTCAGCAACGGCTGTGAGCGGCGTCTTGATCAGAGAATAATTCGGTGAACAAAAACCAATCTTCTATGTAAGTAAAACGTTGCTAGACGCCGAAACATGGTATGCCCTGATGGAAAAATTGGCATTCGCCGTAGTAACATTAGCAAGAAAATTAAGACCATATTTCCAATCTCACACCATCGTGATCCTCACGACCTTCCCTTTGCGGACAATCCTTCATAGCCTGAGCCAGTCGGGAAGGCTTGCCAAATGGGCAGTCGATTTGAGCGAATACGATGTGGAGTATCACCCAAGAACCTGTGCAAAATCCCAGGTATTGGCGGATTTTCTAGTAGATTACCTACGGGGGACATGACTAACAAGGAGCCGAACTCGACCTGGCTCCTTCACGTCGACGGGTCATTTTCCAAACAGGGTTCTGGGATCGGAATTCGTCTCACCTCGCCTACCGGGGAAATCCTGGAACAATCGTTTCGTCTCGATATTCATGCATCTAACAACGAGGCGGAATATGAGGCGCTCGTTGCAGATTTACGGCTAGCCCATGGATTAAAAATTCGTTATATCAACTTACTGCGATTCTCAACTAGTCGCAAAACCAGTACAGCATAGAATACGAAGCGATGGACGAAGACACATATCTTAAACTCGTCCAAGATCTTGCCCAAGGCTTCGATCTCTTCGCCTTCACTAGGATTTCACGCTTGGAAAACGCCCAAGTTGATGCCTTGGCCACACTCACGTTAAGCTTGGACCCGGGTCTTAAGAGAATAATCGCCGTTGAGTTCATAGAATACCCGAGCATCGAACCGCCAGTCATCGCCAACCAGATTCGGGTGCAGATTGAAGAGGCCGAAGCCGAAAACCAACCTGAAGAAAATTTGGATCAATCTGAATACAGCTGTGACAAGCCATGGTTGGAAACAATTCGAGCTTACATCGTCGACAGGATACTGCCCGCCGAAAAATGGGCTGCCCTCAAGATAAAAGCTCAAGCCGCGCGATATGTTACGGTAGACAGGGAGATCTACAAATGGGGAATTCCAGCCCGCTCATGACCAGCATGGAAGGAGACAAAGCGAGAAAAGTTATGGAAGAGGTCTACTCCAGATCATGCGGAAATCGCTCCGGCAGAAATCACTCCGGCCGAAGGTCGCTCGCAGTCAAAATAAAACGCCACGTTTACTACTTGCTGACTATCAAAGACTGCGACAAATTCGCACGAAAATGTGAAAAGTGCCAAAGGCATGCGTCGACGATCCATCAACCCGCAGAGGTCCTTTCGTCCATCACGTCTCCATATCCTTTCATGCGATGGTCCATGAATATCATCAGACCTTTACACAACTCAAACCAGAAGCGTTTCCAGTTGGTCCTCACAGATTTCTTTTCGAAATGGGTAGAGGCAGAGTCCTACGCGAGTATCAAAGACCTCCAAGTCAAGAACTTCGTATGGAAGAACATTATCTGTAGACATGGAGTCCCTTACGAAATTGTAACAGACAACGGATCTGAATTCATCTCTACCCGATTCGAAGCATTCTGCGAGAAGTGGAAAATACGATTAAACAAATAAACTCCTAGATATACACAATGTAAACGGCCAGGAAGAGACCATTAACAAAACCTTCCTCGACGGGTTAAAGAAGGGCCTAAAAAACAAGAAAGGTCGATGGGCAGAGGAACTAGAAGGAGTTCTTTGGTCACATCGTACCACCCAAGGCGAGCTACAGGAGAAACTCCCTTCACCCTCGTGTACGGAACGGAATGCATGATTGCAGCGGAAGTAGAATTCCCTGGAGTACGGAGAAGATTCCTTCCCAAACGAGAAGATCTCAACAATGAAATGCTGCTGGACGACCTCGATCTTATCAACGAACGACGAGACCAAGCGCTCATCCGAATCCAAAATTACCATCACGCTGCCGCCAAATATTACAACTCAAACCTGCGCCATCGCAGGTTCAAAGAAGGCGATCTGGTTCTACGCAAAGTCTTCCAAAACACTGGCGAACGAAACACAGAAAATTTGTGAGCTAACTGGGAAGGACCGTACAAGATCATAAAGGTGGTCCGATCATGTTCGTATCAGATCGCAAACATGCAAGACGTAAAAATCAAAAGAACCTAGAATGCAATGCGCCTCAAAAAATACTATCACTAGCCGCAACATGGTAATCAAGAACTACGAGATGAATTGATCCCCGAAAAGGATACGTAGGCAACTCGTCAACCGACGTGTTCAGCCATCCCTCTCTAAAAAGGGGGGGGGGAGTGGGTACGTATTCATATACTCGTATACTTCTAAAACTTTCAAAAGTCCCGATATTTTTAAAAATCTTAAAACGCGATCAATCTCTTATCGCCAATCCCACTACTAACTAACGGCTACTACAGCAATCCCAGATTCGCTAAAGCGCCTAAGTAAAAAACAAACGGTCATCGCACAAAAAACAACGAATACCCCGAATGCTCTCAAAAGAAGCATCCCTCGGACGAAACAAGCCTTAACATAAGTAATTCTTCGAAGCTCGTAAGAAAAATACGACATAAGAAAAATTCAACTTTCGACACTGCGAGACATCGCTAAGTGCTCAGACAACAATTTCCGACCTTCTCGAGACGACGTTCTCGTCTTCAAAGCCCAAAATAAATCAAAAACACCTCTCAAAAACTTTAACATGCCTCAAAAATGGCATCATTTCTTTGTTGCTGATCACAACAAACGGACCCTAACGTCCTAAACAGACATTAGCCGCCCTTGAAAGGACATGCTCTCTTACATCCGACAAGGATAACATAGCGCGCTATACAAGAAATCCAAAATTTTGGTTAGCACCTCCAAACAGCCTTTGGAAGTAGCTCGATTCTTGCCAAGACAAGTCATATAAGCCGATAACAATTCACGAACTTTAAAACAGTACAAATCAGGTTAAAATCCCAAACAGGCAAAACAAATGCCGACTTGTCATCGCAAAGCCTTAGGCCGAGAGTAAATCTAGGTCTTGCCCTAAACCCAACCATGCTGGGTCTAGACATCTCAAAGACATAGTATCAACAGCCCGATATGTGATCCCAAAATTTGTCTGTACGTTTAACGAGCCTCCGCATAAACAAGAAACTGCAAGAACTCTCGCCTTGCACAAATGATAGGTATGGTGATCGTCTGAGGGGAAGGAATGATGTGGGAACCGAAAGTCACACCGTCGATTTCCGTTTAAATTAGGAAAACCGTAATTTCTAGAGGTCCGGATTCTCTGCGAGAGCCAACGAAAAGTGATCAAATAAATACGGAAAATATGTAAAGACAATAAATCGAGTTTGGTAAAAGTAGATCATTATTTAGAATTTGATATGAGTGTTCCAGAAGCAAAAGCATACAAGAAGCTTGTCTAGAAACGGGTTTACAAAGGAAATTGTATGACAAAAGCAATAAAACGAGACTAAGTTTATCAGTAGTACCACCTTTGTCAAGACAAAACCTGTGGCCGCAACTCGCTTAGCAATAGAAAAAAAAGACGAAAAAGGTTTTGAAGATTTGGGACTGGACCTTATGGAAGGCTGCCTACGTACCCCTTTCCAGGATCAAGTCGAACGTAGTTCAATTAGAAAGATTTGAACAAGAGATCGAACTGCCTAAACGAGTTCGTCTAGTATGAGTGCAAGTCATAGAAACATGCATGCGAGAATAATGCTAAGGGTTTCTAAGTGCAAAGAGTTCTAGAGTCTACAGTTTTCAAGGGTAAAAAGTCGTCCTCTAGAGTCTAAAAATCGTCATTCTGCATCTTCAACTCGACGTCCTTATATACTCCTCCTAAAGGCGGTTTGCTCTTTCCATTTCTGCCCATTGTCGAGTTTATCGCTTCGCGGAAATATTCCATTTCTCTCTGATCTTCTTGTTTAACTTTGGAAACTTCACATTTATCCTCTGAACTTGACATTTATCTTTTCCTACATAATAAAATATAAACCGTCATAACAATTGGGCTTGATTTCTTACAAAATCGAGAGTGGGCTTTTCACCGTATTCTGGACCCTTTCGGGCCGTTTTCCGACTTAAGAGTTTCTACGATTTTATTGAAATAGTGCTTTTAAGAACCGACGTCGATTCCGAAGAAAGTTCAAATTTCTCGTTTAGTAGAGGCAGTTCAAGGTGTTCAGTTAATCATTGCCGTTTTATACAATCAATTCCAATTCCATAAACGGAAACAAGTTCTTCCGGTTTTCATCGTAAGGTTTCGATGGTGAATTCGATAGAGATGGAACAAGAGCAAGTTCGATCCAACTGCGAAGGAAAAAGCTATGAGGACGAGAAGAAGATGGATTGGGCAATCCAACTCGCCATCATGCTTGGTCCAACTCGCCAAAATGGCTAGTTGGCCATTTTTCTTGGTCCAACTCGCCATTTTGGCGAGTTGGGAGTGTTCCAACTCGCCAAAATGGCTAGTTGGCCATGCTTCTCGGGAAAACAGGCACGAAAGGAGGTAACCGATGACCCAAGGGAAGCCAAAGTNNNNNNNNNNNNNNNNNNNNNNNNNNNNNNNNNNNNNNNNNNNNNNNNNNNNNGTCCGTCCCAAGCCCACTCGCTCTAGGGTGAGCGCGGTCCGTCCCGTCCCGCTTTCCCTAGGGCGAGTGCGGCTCATTTAAGCCCGTTCGCCTTAGGGCGAGTGCAGTCCTACCTGTCCAGCTCGCCCTAGGGCGAGTGCGGTCTTTCCCGTCCCGTCCGTCAGCCAAATGCCGTGCATTCTCGCTTAAGTTCTCGTGATTTCGTACTTCGAACCATGAATCCAATGTTTCCCGTTCCACCTCGATTGGAGTTATCGTCGAAACTTCACGATAGAAACCGAGAAAAAATTGTCTTTGTATAAAAGTTCGAATCAATCGTATAAAACAGCGGCGGTTAACTTACAACACCAATTGTCTTGACTATACGGTTGATTTAAGGATTTTTTTACAAAACCGGCGTCGCACAAAAGGTAACCTCTCGAAAAATCGGAAAAACGTCCTATGCCAAAAAATGGCCCAAAGAGGTCTAAAACACGGCGGGAGGCCCGCTTACGATTTTCAGCGAGATTGAGTCCAAAAGTATTATGACGGTCTATTTTAATTGTTGCGAGAAGATAAATGTTAGGTTTCCATGATAATCATGAAAATGGAATATTCCTACAAAGGGATAAACTTGGGCCCAAAGGCGAAGAGGTAAAAAGGCTGCCTACATGAGAATCTATATAAGGAGAACCAGTGCAAAGGATGCGGGCAGGGGGGGATCTGAAACTTTTAGACCTAGAAAAATCATGTTAGCTTAGGGAACTTAGAACCTAGGTGGCTAAACTAGCGAAGCAAAACTTATAACGTTTTTCTACTTTTTGTTCTACTTTTTTCTTGTTAACAAATCTCTGTTCGTTTTCCTCTCGGAAAGACCTTGTAATCATCCTATTTTTATCAACCAATAAAATGCCTCTGAGCAATACTCATTCAATATCTTGCTATCTATCTCTTTTTCGGTTTCGTGTAATTACGCTGAGAATCCAGACCTCCAAGGAAAGGCAGGTTCACTTGGCCTTCCTCCATTCTTATTCATTAGAACTCTACGGTGTGAGTTTCGGTTCCCACAGCTATTCATCGGAGGTTAACAAAGGGTGGGAGATCGAGAGACAGAGGAGATAGAAAGTTTAACGAAGACTGTTCACAGCGGTTCAGACAAAGAGTCATTCGTCGTAAAGGGAGTCTCACTTGGCTTAATCGGAGGGTTTTCTTATTTTGGGCTTGAGAGACCACTTTGATGAATTCAAAGGTGTTGTATCATTATGGGTTTTTAGACTAATTATTAATTGATCCAATTAATTAATAATCTTGTTATGTACGTTTGAAAAAGGCAAATTTGATTAATATGTGGGTCCCAGAGTTGAAAAATGGACACGTAGACAGCGTTAGGAGAGACGAAAGTGCCTCATTATATATAAGATTAATTAATTTACCAGAAATAATCAAAAATACAATCTAAATCTTTTGATGATTTTTTTATCATTGAAAGATTATGACAATCCTACATTAACCACACATTAAATTAAAATTTAACCTTAGTTGATTAGTTAATTTAGGAGGAGAAGCATTCACATCAAAGAGCGAAAAGAGAATTTTAAAACCCATATTAGAAAAGAAAAAAAAACAAAAGAAAGGATGAAAATGATACTCATAGTAACCACACAAAACACTTATATTTTTATAAGTTGGTCAATTTTTTAAAATTTCCTAAACGTTAGTTGATAAGTGATTTGTTTATTTGTCATCAGCTTAATTACTCTTAAAAATGACATGTATTTTGAAATATGCGACATGAAATTAATTAATTAATGTGACATGTAAATTTTCCTTAATAAACATTTATATTTTTGGTAAGTTTATGAAATATGTTGATGAGTAATAACTCATTTATCACCATTAAAATAATTTTTTTGATAAAAATTATTTTTCTGAAAAGGCTTTCAAAATATGACAATAACTAATAATTTACATATCACTATAAACTATTTTTTATGAAAAATAAAATAATTTTATGCTTAAAATCTGTATTCCTGCGTATGGCGCATGAAAACTTTTAGTTATAATTTATTTGTATAACTATATAGTACAAGATAATAGTATTTTTGTTTGAAATAGTTCTCATGGATGAAGATATATCCTTATGGATTTCATACATTAAATAGTGATATACTAGTCAATTATACAAAGTTTTTAATCTCCGTGAAAAATATCAAAGTGACGCTTATTCAGAAACGGAGGGAGTATAAGATTTGAGACCAATTCCTTAATTTTCCTAAACATGTAACACATGTATAATACTAGTATTTTTCCAGCAATCATTTTTTTTGTCAGCTATCCATCTAAACTAGATCAAGTGGTGGTAGACGAGCCGATCCTCCGAGGAGCACCTCCGTCTGTCCGGATCTGATATATATGAAAAAAATATAACCTCTGTTCCTTACTTTCTTTGCTAGTGCGTCTGCACGATTATTCCTACTCCGAGGAATATGAGACAAACTCACATCCTCGAAGTCCTCCTGTAATCTCTGGTACATCTCAATCTCTGACGCGAATGTAGGCCATTCCATCGGGTTAGTAGTCATGTCCACTAGGTCCGAGCAGTCTATCTCGAACCGTACCGAGGTTATCCTCCTGTCTCTCATACATGAGGCTGCCCAAAGTAAACCTTCCATCTCAGCATGCAGAGCTGAGAGGCTCCTACTGCATGCCCACAGTCCAAAGTACTTAGAACTCATTTGATCCTTAAAACTCCACCCTAAGCCACTGACATTGCCATTACTGATCCATGACACATCAATTTGACATGCAGGGATTCGGGGTATCCAAGGGGGCATTGGAGAGTCTTCCGTAGTAAGAGGATCGCCATGATCTTCATTTGCTTCCTCCTTCTCATTAGCCTTCCTCCAACATTCTGCCTCGAGAGATGCGTGTTGGAGAGTGTCAATCGAGAAGCGACTTTTCCATAAAGAGTTTGTCGTTCCTTGCCTTCCAAATGTACCAACAGATCCATGGGAAGGTATCGAATTGTGGTCTCGATGGAGCCACCTCTTTTCTCTTCCAAAACAGAAAGTTCATGTTTTGGTATATAGATGTACTCAGGAAGTAACCCGGAAGGGACAGATAGTTCGATAAAGCCCAAACCTGTAAGGCTGGGGGACATTCAAAAAGAAGATGATTAATCGAATCCACTGGGCTAGCACACTAGGACAACTCCTATCGGTGCCTAGGTGTCTATAAGTGAGCCTTTCTGCCGTCGCCACACATCCCGAGATAGCCTGCCACAAGAAGTGCTTCATCTTACTCGGGGCCTTTATCTTCCACACATGGCTCTGAAGATTCGTGATACTCAGTTCTATAGCCCTTTCCTGTGTAAGATTCAGCTTGGTCGATCGGAGTAAGTCATAACCGGTTTTAATTGAATACACTCCTGATTTTGTGTAGTTCGAAACATATCCATCCGGAGCAAAAGAGCGAGAAGGTTTTAACCCTAGTATCAATGGAATATCCTCTGGCTGAAAAAAATCCCGTAAAAGCTGGATATCCCACTCCTTGGTATCATTCCTAATAAAGGACTGAACAAGAAGTTTGGAGAGTCTGTATACAATGTGGTCAGCAGGTCTAGGTGGTCGAGCCACTGAGTCTGGAACCTAAGGCTCACTCCAAATTCTTGTATCTCGACCCGTGCCAATTGTTTTTTGTAAACCCGGTATGAGTAGAGGTTTTGCTGCAATGATACTACGCCATCCATAAGATGGTGAGTATGTTCGACGATCTTTCAAGGGAGATGAGTGATTGTAGTACCTTCCTTTTAAAACACATGCCAATAAAGAGTTTGGATAATGAATTAGTCTCCACAATTGTTTTGCAAGAAGCGCTATTTCGAAGTCATGGAGATCTCGAAAACCTAGTCCCCCCGCATCCTTGGGGGTACAGATCTCGTCCCATGCTATCCAATGTAAGCCTCTGCTGTTTTGTTTTGAAATCCACCAAAAGTTGGAAGTTATGCTCTTTAGTTTATCAATAATGCCTTGTGGAAGCAAGTAACTCAACATTACAAATGTTGGAACTGCTTGAGCCACATATTTAATTTGAACTTCTTTTCCTCATTTGGAGAGGAGTCTTGATGACCAAGTGTTGGTTCGTCCATTGAGCCGATCTTGTACAAAGGAGAACACTTTCATCTTCGAGCCACAAATTTTTTCAGGTAATCCAAGGTACATCCCCATACCTTCTTCAGAGGAGAAGCCTAAGACATCTTTTATATCTTGTTTTCGAAAATACTCTACTCGATTTCCAAAAAACATAGAGGATTTAGACGCATTTAGTCTTTGTCCTGATGCCTTCCCATATATATCTAGAATGTCCATGATCTCTTTGCATTGGCTTAATTTTACCTTGCAAAAGAAAAGACTATCATCTACGAAAAAAAGATGAGAGATCTTCGAACTCGCCCTAGCAACATGGAGACCTACAATCTTTTTCTCCACTTCTGTTCCATTTAAGAGAGATCAATACATCTGTACACAGGATAGGGGGAAAGGGGATTTCCTTATCTCAAACCTCTCTTTGGTAAGATTTGTTTTCTTAGTTGTCCATTAACTAACACTTGATATGTGATCGACGTGACACAACACGTAATCATTTAAACCGTAAACCTCTGATGTCATAATTTTTTTCTGCTGAACAAACGAACTCCGATCAATCAAAGAGGTTCAATTAATATGGCTAGGATTGGCATTTAATCAGATAGTAGTGGTCTAGAAGCCCGGCGTTCGAGTGGTGAGATATTTTTCGTATAGACACCTAGTCAATCTCATCAGGTACGAAACTCCATCCATTGTTGGTTTTGTTTTAGACACCTCATTTGACGAGAATATATACGATTTGCTCTGGTTAAAGAACAAAAACTTGAGTTAAAAAAAAAAAAGAACAAGAACTTGGCAATTTTGTCTCCATACTTGTTATCTCTCAAGTCAAGTTCGTTGGGCCCTTGGGCCTTTTCCGTAATCAAGATTCATGTTTCCAATTTGAATCGTTAAATCTATGAACTCAATACCAGCCCAATACCAACCCAATACAAGGCTTGTATCAATGTCACCAAGTGCATCAGATAGCTACTGAGTTCCTTTCCCATTTTTCTCCCATTCGGATACTAAACTGAACCAACGGTGAAAAGTCTTTATCTTCTTGACATAGCCACCTTGATCTGCTCTTCTTCTGTATCTCGATCCAACTCTGTACTGAAAAACCAAACAACCCAGAACCAAACCTAAGAATGAGAAAAAAAAACCCTAACAATGACTATGAATTTTGATTCATTCTAGAAATCTTAATTCATATAAGATATGGTTTAGATTGGTTTCTTATATAATATAAATTAGATTTTGGTATGCCCTTTGAAAACGCGAGATTGTTTTTTATGGACAAATTTATCTTCTTTAGATGTGATAAAGTCAAATTAAATAACACTAGCTCTGCCAAACCAGTCTCTTAGGAATAGACATGAACCAAAACTATTACAAACGTACTACTTGCAAATTAATATCACGATGACAGATTCGTCATTACACGAAAAAGGTCGCCTATCTTAGAGTCATCCATGATCTTGCAAAAGATTTTGAATTCTTAGAATTACCATGTATTCTAAGGATAAAAGGACATATTGCATATTATCGATTTATCAATGAAGAATGGTTAATTAACATCTATTTCATAATTTCCTATTCTAGTTCTATACCCTCATGCAACGAACTTTGAAATCTCTGACTAATCACTAAAATAGTATAATTGCTATGACTTGGTCACGATCTTTAATATCTTGAGTCTTCAAAAGTAAAATTAACCAGCGCCCGTGAACGATGTAGCATTAGCTCAAAGTCAAAGTAGCTGCTTGGGTTAGACTAGATAGAGGCTTACAGAGACAGCCTTCCATAGGTAAAAGATATGCGGTGGAGAAACCAGCCATTAACTTTGAATATTGGGCCAGTCTAGAAGTTAACATATGTTTAATTTGCTGGGTTACTACGTTGTTGTGCGATGAACAAGTAAAAGCTAACCTACTAGCATTTCACTATAAATAGAAGTCCATCTTACGCTTATTTCTTCACACTTCACACAAATTCATCGAAAACAAATTACTAATAGTCATGACTAAGTTTGTTTCCATCATCACCCTTATCTTCCTTGCTCTCGTTCTCTTTGCTGCTTTTGGTGAGTAGTGATCTTATCATATGCATGGCAAAATTAGTTTAAATTTTTTATTTCAGAATATTTGATTTCGCCATGATAGATATTTATATACAGAAGCATCAACAATGGTGGACGGTCAGAAGTTGTGCCAAAAGCCTAGTGGGACATGGTACGGATTTTGTGGAAATAGTAATANNNNNNNNNNNNNNNNNNNNNNNNNNNNNNNNNTGCATCAACCTTGAGGGAGCACGACATGGATCTTGCAACTATGTTTTCCCATATCACAGGTGTATCTGCTATGTTCCATGTTAATCTACCAAAACTCATTGGTCTAACAAAACTCTGTAGTGCTAACATTCAATAAGTCTGTGTCACACTATGAGTGACCTTATAACATGTACCAAGTAAAAAGTATGTTTATGTATGTTTTCTGTGATACTATAAAGTTTTATGTACTACGTACTACTGTTTCTTTTAGTCTATTTATCTGTCTGCACCACGCCCTCAATTGGACACAAAGATTAAGAACATCATTGTCTAGTAATAATAATAACAAAAAATGCATAAGAAAGGATAGGTATGTTCGAATATAAATGAAAACATGAGAGACTTAAACTCAAATGGAAGCATCGAGAATGTTAACAATGGGACGAGCACATGACATGCATCGGTGTCGCTATGCATCATGCTTCCTTCTTTCATGTCAACATATTCTTTTCCAACTTTCACCCACTCGAAACACTGAACCAACGATCCCAGAGCCAGGTTAACTATGAGCTAACCCATACCTAGGACAAGCTCGTCGTCCGATCTCTAATGATATCAACTAACTTTGGTCCACACCTTTTTTTCTCAAACCTTTCTGGTTTGAACTTTTCCGGGTCTTCCAATATATATAGATCTCCATGTATTAGAGAGAAGATCCCACATAAAAAATTTGAAAGAGACTTGAGTAATATATAAGGGATTTGGACCAATCCACTTAGTGCCAATTGGTTTTAAGTTGAAAGTTCATGAAACTTATCATGGTATCAGATCGGGCCCACATCCTGACTCATTAATCCAGCCTAGAAAGTAGCCCGATCATCCCATAGCTGATGGCCTATAAAAGGTTCAGTTCCACTGAAATAGCTAAAAAAAGAGCATTATCTCGGAAGAAGTATGATGGATTCTGCGAGATTATTAAGCTGGGAGTCCAGAAAATTTATTACTATGCATGGCCCATACGTTCACCAACAACATTGTCCCACTTGGAATATCATATCCTGCGACCATGCAATCCTCAGATGCCACATGGGGTAGAAGAGTTGGAGCTATCGGGTATAGGCGTAGGGTCTCCGATAAGATATTTTGGAGATAAAGGGAGATTTTTAATATTAGATTCGTCAATTGACCTGTCTAAACCAATTTGGTTATCGATATCGGTTTTTTTTCCTTTTTCAATATCTCTCGATGGTTTAGCAAATTTGAAATCGTCTATTCTAGGGTTCCAGTTAAGGTATCAGTCCCCGAAAGTATCATAACATATCCAGTCAAAATAATTAAAAAATCACAAGACACTAAACACAAAAAGATTAATGACTAAATATTTACGACAAAGATTCCCTTGATGACAACATGATCTGTGTAATAATCAGGTTGATTATCTTGGAGAGAAAGCAGACGATCGATCATAGTATTACCCTTTTCTTTTCCCGCACGTTTCTCGTCAACAAGTCCTTGGAAAAATTCATCAAATCGATCTGCCACCTTTTTGACACGTCTTTCATAATTAGTGACCCAACACAAGACCGGAAAATATTCAGCAGCGTTTCCAGCGCCGGCACAATCCACCACCTCAGCAATCAACTGCCTAACGCATCTTGCATCATCATCGTGTTCTGTTCCATTCCCGTAAAATCGCTTTCCAGCCACCATTCTCATAACATTGTTGAGAATGAAGTCCACAAACAATGTTCTCATCTCCACTTTCACAAACTCCTTGATGATTTTGATCATGGAATCACAAATGGTTAATTCTCAGTTTTATTTTGTGTATGTAACAAAAACATACACTAAGGCAGGGTGGATTAAGCTACGCAAAATTTAAAATTGGAAAAAGCAAGAAATAAGTATGATAATCCGTAGGAAAGACCATTAGTTATCCATATGACAATATTTATAATCTTACGTAAGTTTTGTGAGTAAATTGGCTAAAGATTCACATACATATTGAGAGTTTTTTGAGTGTCGCAAGATCAAATGTCGGATTTTGTCTTTACGGATGGATAAATAGCTATTAAGCCTGTGAGACGAGAATATCTCAATTGTGCCAAGTCTCCGGAGATTGCGCCAATGATCTCCGTAAGGGGCACTGACCATGGATGTATAGTTGTACGTACTCTACATTGTTTCGAGGTAAGAAACTTTGGTCGATTTGGCTAAGACAACGTCGTTTTTGGTGAAGCATTCGTCAATAACGGAGTAGGAGGAGATGACATACGTGAGTTTGTTTCCCAGGCGGAGGCTGAAAATTGGAGCACCACCAAGAGATTGAGAAAGGGACAAGAATGTGCGGTGCAGCGGCAGTTTAAGGAGGTGGAGGTGTCCGATCACTGGTAATGGTCTTGCTGGACTAGGAGGTAGGTTATATCTTATGTGTTTTCTACTAAACACAAGTTTTAAACAGAGGATTAAGAAGCCTAATAAAATAAAGTGAAACGTCTCCATTGCATTATGATTGATATACTTTGTGATAGTGTTCTAGTGAAACATGTGAATTGAAGTTATATATGCATAGCAAGGGCGAATATTCGTGGACTGCATAATGATCATGGCTTTGTCAATTAAATTTTGATGTGTTATATCAAACAAGGAAATGCTTATGTTTTAATTGATTTGATGTGGATATCAGTAAAATATACAATAAGACTTTGACATGTGTTGATATCATTTTTTACCCAAAATCATAAATAGAGGATGAGACAACATATCAAATTAGCTGACAAATTTTTACTATATTAAAAAGGGAATATTGTCATTTGTAGCTTTACTTTTGCGGATGTGTAAGTATCATAACTAATTTTAAATTAAATAAAGAAATAATGCAGAGAACTGAGTTTAGAATGTACATCTGTGTACATTATTTAATCAACAATATTGTAAATAATGGTAATAAATCATCTAAATTTAATACACGTGTCAGCATTTGTTATTCGTTAATTTACTAGAAATAATAAAATACAATCTAAATCTTTTGGTGTTTTTTTATCACTGAAAATTATATTAGTCCTACTTTAACCACCATTAAATTAAAATTAACCCCTTAGTTGATAACTTAATATAGGAGGAAAATAATTTACATCGAGCTAAAAGAGAATATAAAGCCCATATTTCTTTAGAAAATAAAAATAATAGTACAAAGATAAAGAGAATAAAACTTAGAAAAATTAAAAATGGTTCTATTTGGAGAGACTTTCATCAAATTTTGAGAGATTCTGATACCACTTGTCTCATTCTAAATGATAGAATTTATTTTAATTAAAAAAATTAATTATATAATTAGTTAATCTTAAAATCTTAACATAGTGATATATATATATATATGTATATATTCATTTACATAGATTTATGGATAATGGTGTTCTTAGAACATGTCATTTGAAAAGTTTTCAAGAGATTTCAAATGAATATCTTATTTTTAAGTAGTGAAAAAACGAAGAGAGGATGAAAATGGTACCCATAAAACCCCACAAAACACTTGTATTCTCATGGATGAGGATATCCTTTTGAATTTCATCTAAAATAAAGACAGATATTACCTTATTAATTTAACTTCTCTCGGTCAAGGGTGTTTATACACAATCAAAAAACTTTTCAGTGAAACTATTTAGCCACTTAGTATTTGCATTTCTTATAAAAACCCAAAACCCCATTTTCTAAAGCGATTTCAGTTGTGTAGAAGTATGCACAATGATCTTCCGTCGTGGGAGAAGAAATCTTTCGGTGAGATCCAACTGAACCGAATGTGGCTCTGGTAATTTTTGGTTTAGAGTTAACCACTTATTTGTGTCTTCTTTAATTGCTACTTGTCTGTGGCTTGTGGCTGGGGCTCTCTTAGTAGACCAAGAAAGCTCCGGTCATGGCTGTTGATAGAACAAAGCCTTGCAATGAGGTTGAAAACGATATACTATAAACTCGTATTAAAAAACATTATCCGATGCAATTTACTAATATACAATCCTCTTAGTCTAAGATTTTAAGTATCAGGTTAGGGGTTTAGTAATTTTTTTTATAACAACAAGGCGTTTAGTAGATTGATCATCAGAAAGGTGAGCTTCTTATGTTTAAATAGGACTGGACATTTTATTCGTTAAATTTGATTCGATTCGTTATTCGTTTCGATTCGATCCGAAAATTTCGAATCTCCGTAAACTTTCGAAGCAAAGCAAATACTAAAACTCAATATCCGTTAAAATCGAAGCAAATCACAAAGACTAAAATTTTGGAAAGCGAATATCCGATCCGTCAATATATAAATACATGTATATCTTGATTATATTTAAAGTTTTAAATGTATAAAAATTTATAATTATTATTATGACATATGATTTGACAAATTCTATTCACATTATTACTTATATAAAAATATTAAATAAAAAGAAGAGAAAAAATCTATGACAATTATAATTTTTATTAAGTTTTGTGTTATTATAATTGTTAACCAAGTCTAAAATTTTTACAAAATATGTAGATTCGCTATTTCTTTTAATTTTTTATCTTTATATCATGCAAAAAAATATTTTACAAAACAAATTTGTATCAAATTTTTAAGATTATCTGTATTAATCCAAACAGATTAGATATCTGTAAGTATTCGTAAATATCCGCAAATATCTATTTATTTTCCGAATATCTGTTTTTCCGAATATCCGTATTTTACCGAAGCAAAACAAATCGAAAAATTAGATATCCGTGACATTCGAAACAAATCACAAATACCTTCAAAAACCTAGATATCCGATTCGTGTCCAGATCTATGTTTAAATAACAAAAAATATGAATGGACAAGAAAAAGACCCACAGAAAATATCTGGTTGAGAAAGTAAATTAAGTGTGTAAACTATGACTTNNNNNNNNNNNNNNNNNNNNNNNNNNNNNNNNNNNAAAAGTGATAAAGAACAACGTAGCGTTGAAAGCAGCCATTGCCAGCACACTGTTTATGAATTTGTCAAGATTTCAATTTATCTACGATATTTTGCGTGTTAATGCTGCTAGAATACGGTCCTATGTGTTTAATTTGTGGGGTTGCCATGTTATTGATTGATTGCACAAGTAAAAAACTCACCATCATTTTCCGGAGTGTTGGTCTCAAGGTTACTTCGGTTGGAATTGCGAAATCCCATGTCCAACTCTGTATTATCTAATAAGTACGATATTGTTTATTTTGGGTCTAAGGAGCAAGTCCGCATGGATTTACTTTTAGGTTTTTTTTCAAAAGGTCTCGTACTATTAGAGTTGGATATCTCTTTATATATTAGATTCTTTTTTTTTTGTCTAATTTCTAATGTGGTATTTTATTTGATATCTTACATTCTCCCCCCTCAAACTAAGGACCACACTCATCTCGTGTATTTTTTCTTTTGAGAATGCGTTTCACATTCTCGTTCTTTTGAGAATGTTTTTCCCACTACACACAGGTTTCATGATCTTCTGACGTTTCTGCCACATACCTCCTCTTTCACTTTAAAGGATCTCAATCCTATTCGAAGGATATTTTAGTCTTTTTGCAGATCTTCGTCAAACCTGGAACTCTGATACCAATTGTTGGGATTGCGAAATCTCATGTCCAACTCTTTATTATCCGATTAGTACGATATTGTCCACTTTTGATCCAAAAAACAAGTCTGCATGAATTTACTTTTGGACTCCTTCTCAAAATATCTCGTACTATTAGAGTTAGACATCTATTTATATATTAGACTCTCTTTTTATCTAATTTACAATGTGAGATTTTATTTTATATCTCACAACTTCCCCTGTTAATCTACCGAAACTCTATGGTGCTTAATAGTGCGTGTATTCTTATAAAACAAGTCTGTGTCACTCCATTAGTGGTGGCCGTATGACATGTAAACCTATGTTTACGTTGCATTGCTTATAATTTAAACGTTTATACACGACGTATATTCGTGTTCATAAAAATTAGATACACATATAGTATGGAGTTGTCAACGACATCCACACACATATATGTCGTTTACAGTTCAATACAACAGCGTATAACATTATTTTATTTAATAAGGTTTATAAAAAAAGACTAATTATAGACCGATGACAAAATCACTATAGCATGAAGTTCCTTTTTTTTCTTGCGACTCAAGCAAATGCAACAATATTTTCTTTGGATCCTCTTCCACATCTACGCTAATGCAACAAAACGTAAAAAAATCTGAACACTACAAATTGTGTAACTTTACTTTTTGATTAGAGAGTATTCTATCAATATCACAAGGAGAAAAAAGAATTCTCAGATAAATTTATGAGAACCCCATAAAAGTTACTTAAAGCATTTTTTCTTCGTTTTAGTGGTTCATCTAAAAATTATATATATATCAAAATTTAATAATATACAATCACAGTATATCAAAAAATTTATGTCATTAGAACTTCTGTGAGGTGTTCCTATCTTCTTGAATCTTGACCGATACAAATTAAACAGCCGCACATGTGAACAATTAATGTAGCATTGGCGATTGGCCCAAAGGCAAATCACTCGCTTAGCGCCTAGCTTGCATTAGATAGATCTTTTTTTTGCATTAGATAGATCATGCATCCTATGTAAATATATGTGGTGGAGAAAATAGGCATCAACTATTGATTAGTCAATATCTTATCGTCTATTATTAGCCACTTGCGATTTAATAAATGTCTCCAATGACCTAGATACCAGTATGTGTTTTTCTATAAGAAAAAGAGATACCAGTATGTGTTCCATTTATGGGGTTAAACTTTTATTGCAGGATGAAAAGTAAAAGGCTAAGCTAATCATCATATCATTATAAATATGGGCTCATCTTTGGCTCAATTCCTCACACATTACACATACATACATCACAAATCATGGCTAAGTTTGCTTCTATCATCGCACTTCTCTTTGCTGCTCTTGTTATCTTTGCTGCTTTTGGTGAGTAACGATTTTATCTTATGCACGAAATAATGAGTCTTTTTCCATTTCAGATTATTTGATATTTTAACATCATATATATATATGCAGAAGCACCAACAATGGTGGAAGCAAAGTTGTGCGAGAGGTCAAGTGGGACATGGTCATGAGTCTGTGGAAACAATAACGCATGCAAGAATCAGTGCATTCGACTTGAAGGAGCACAACATGGATCTTGCAACTATGTATTCCCTGCCCACAAGTGTATCTGCTATTTCCCATGTTAATCTACCAACATCGTTGGTGCTTGAATTCTTATTTACCGTTTCTTGTGTGCTTATTTTCGTAAAATAAGTCTGTGTTACTCACTCTATGAGACATGTAGCATATATGTTATGTTGGTTTGTTTTTAATAATATTATAAACTGTTGGCATGTCGAAAATCAAACATTTCTCTTGGGCATCAAACGTGTATCTAGAGTTTGGAGTTTAGTATCAAATATGTAGCGATGTGCTCCTACTCTACTGCGAGTCACTAGTAGGAAGTATTAAAGCTTTCCTGGTCGAGCTTCCTTCACCTTTAAGTTAGGTTACCGTTTACTTTGGGAGTATAGTTGGTGTTATACCTTGATATATAAATGGGGTTTTTTATGCTAGACATGGAACTACTGTGTTTCAAAAAAAAAAAAGACATATGGAACTACATGTTGTAATCCCTTCATAACCTTTTTTCTCAATACTTGAAGATTTGATTAGTTGACCACAAAAAGTTGTATATTGTTCTGTTCTTTCCTTGGTTCTATTTTGTTCTCTTAAACCTTTACAATTTGATGTAAAACTATAAATGTTAGAAAAATGATTTACACAAAACGGTAATAGTATGTTGGTACGTCGAAAGACATAGACAAAATGAAATACTTGGAAGCAAAGATGCAAATGAAGATAAAGATCATGCAAAAATATCATAAGATAGAAAAACGAACTATATGATATCCGCTGACCGATTTCCCAGCGTTTGATTAATCTCCCATTATCATATAAGAAAGAAAAATGAATTATGTTTTATTTGAATGTTACTGAAGAAGATCGGATCAATATAGTAAAATCAATGGCTTATTGGCACTTGACTATTTTCATCAAACTTGACTCGGATTGAATCTATCATGGGAATATTTTATAATTTTTAGAGATCAAGTACAGAACTAATGTTTTCTAGACATTTTGTGACATGCCATTTGGTTGTTTTTCGTGTTAATGTTGAGTGCATATGGTTTTAGGTGTTTTTAGTGCCTCAGTTTATATCATGTTTGAGATTACTCTCGTGTATCAAAAGGCTCAAATAATATTTTTGTTAAAAAGCATCAAGAGGGATAAACCTTGGTTGTGAGACCCAAACCATGTGAATTTTTAAAAATCTTTTTGTAGCACCTAAGTAGGTAGCAACTTCACAAAACCGGTTAGCGTCATCCTTGGTTTAATTAGAGTTAACCATTTTAGTTTGTGGTCTTCGTCTGAGTTTGCTACTTGTCTATGTCTCGTTCTGGAACTCTCTTAGAGCAACTCCAAAAGGGTATCCAACCCACTTCTAAGTGAGTATCCATCTAATTAATTGAGTTTTAATAAAATCAAAAACATGAAAAATACTTAAAGTTGTCTTTAATTCTAGATGTTTTTGGAGTTATATCCAAAAGAAACGTGTCATTCACTGGTTGAGAGTATAAAGAAAAGGGGAAAAAAATTGTCGACTTCTCTCTCTTTTTCGCGGAACAATTTTTTTTTTGCTCCCTCTATCTTGGATTCGTGTTGAGGTCAAAATCGGTCAAAGACGCAATCAACACCCGAAAGTCCTCGGAAGGGATCAAGTGACGAATAAAACCAAGAAAAACTAGTTACTCGACGAAACGTCGGAGGGATGATCACATCGTGGTCCCGGACACCAGCAGGAGCGGCCACGGCCTGGCCCCGGGCGCCGGCT

scaffold 3

CCCCCCCCCACGACTTTTGAATCTCTCTCGATTTCGTGCTTATTTGACCGTTTCTAATTGATTTTAGATATGCGAGCTTTCGATCAATTGAGGCAGCTGGAGGTTTTCTTCAAGGATGAGAGCAGGCATGGATTACCTGTGGTTGATTTGTATGAGCTTGTTCAACATGCCGGCAACATATTGCCTAGGATGTAAGCAGATGGATCTAGATATCGTTGACCTAGTTTTCTCTTGATTGGATTAGGATCTGATAAGACTCAGTTACTTAGCTTGATTGGACTACAGTTATAAGATTAGACTAGGAAAGGGCGATGATCTCGTATGATTTGCATTGATTTGGGTGTTACTGAGTTATTTTCTGCACTGCTGCCAGGTATCTGCTGTGTACAGTAGGGTCTGTTTATATCAAAAGCAAGCAAGCTCCTTCGAAGGATGTTCTCAAGGATCTCGTGGAGATGTGTCGAGGTGTTCAACATCCAATTCGTGGACTCTTTTTGAGGAGTTATCTTGCGCAAGTGAGCAGGGATAAGTTACCTGAGATTGGCTCAGAATACGAAGGGTGGGTCGTAACTATGTGTTTCACTCTCTTTTTCCCATAAAAATTTGGGTGTTGAGCTGTTTGTTTACTCTCCTTGTAACTCCCGTGCAGAGATGCAAATACTGTGATGGATGCTGTGGAGTTTGTGCTGCAAAATTTCACTGAGATGAATAAGCTCTGGGTTCGAATTCAGCATCAGGTGAATTTTTTCGATCTGTGAAGAGTTGGTGGTAGTTAGATACGCATAGCACCTCTGCACTTGCATGCCTCTGACTGAAGCATAAATGAACTATGAAGAGCATGCTTTTGGTCATGCAGGTTGTGTGGTGCTTGTGATTCATTGATATTGGTCAACCTGAGCTGAGGTGTTCCTTGGACTGCAGGGACCTGGAACAGTTCGAGAAAGGCAGGAGAAAGAGAGGAACGAACTTCGTGATCTTGTACTATCGATACCACTTCTGGACTGAATACATATTTTACTATGTCACTGTCCAATAAGTTGTATTTAATGTTTGTCCATCAGGTTGGAAAAAATCTGCACGTTCTAGGACAGATCGAAGGTGTTGACCTTGACATGTACAAAGAGACTGTTCTTCCTAGGGTCTTAGAACAGGTTCAGCAGCTCATTTTTGCATCTATCACCAGTTTCGTTTTGTTATAGATGATACTTCAAGTTCCTGGATAATCTGTTAAATCTGGAAACCTTGAACAGGTCGTCAACTGTAAAGATGAGCTGGCTCAGTATTATCTGATGGAGTGCATAATCCAAGTTTTTCCTGATGAGTACCATTTGCAGACTCTTGAGACTTTATTGGCTGCTTGTACTCAACTGATGGTGTGTACGCTGACAAACGATATTTCTCTCTCTCTTAATCTCCAATGCATTTGTATGTTATCTAATATGATCGGTCGGCTGCTTCCTGATTAACTGTTTCTCATGCTGCCATTGTCTCTTGAAGGGGTCTATCTGAGCTGTCTGTATTGCCATTTACTATTCTTGTTGTTTGCTAGTATTTGGGGTAGATTTTTGTTTAATTTTGGTGACTAGAGCTTTGGCTTACCAGTTAGATGTTTTTAAGGAGGTCCCTGATCTCTTACAATTTGACTGAAATTACAAAATTCTAAGTAAGGAATTCTCGACAAAGAGCACTTTGTTTTGTTTTCATGCTGTGCACTTTTGCCGCTTGAGATCTTTTACCCGTTTTCTTCCTCTCTGACCTTAAGAAATAACTTCTCAGCCAACAGTCGACACCAAGATAGTGTTAACTCAATTGATGGACCGGTTGTCAAATTATGCTGCCTCAAGTCCAGATGTAAGTGTCTACTGAGCTAACTTCGTGCCTTTTAATTTTTGTTCAATGTTACTGAGATGTCTCTCCTGTTCATTACATCTGGGCAGGTGTTACACGAGTTTTTGCAAGTGGAAGCTTTTGCTAAATTGAGCAATGCAATTGGAAAGGTTGGCTCCATGAAGGAATATTTATTATAAATGAAATTTTCTTTTCGCTGTTGGATATTCTTGAAACATAACGGCCCCTAAAATAGTTCTATGTAGGTCCCCAGTACCATGAGAAGTAGTCAGCTTGATTGTACTGTGTCATGATCTATTCCAATCTTTGGTTATCTTCTCCTAATCAACTCTTCTGTTAGGCATCGTCTAATTCTATCACATGTGATCAACAATAAGGCCACGATAGTCTGTAAATGTGTCCTCTACTCGTTAGATTTGAGAGATCAGTAATTAGAGGGGGAGAGCATCTAATCTTCCGCAGAACTTATATAAATAAAAAAAAACTAAAATATCAGCCAAGCTATGAAATTGCTAGATGATGAACTTGATGCTTTTTCCCCGGTCTGGACCCAACTGCCTAGGTCGATGGACTTGATGCTTCGAGTCTTTAGTTCTTCCAGCCTTCTGCATATGATGCTTTAATTTTCTTGAAATACATTTGTATGACTTATGGACTTGGCATGGGTTGCAGGTGATAGATACACAGATTGAGATGCCTATTGTTGGAGCTATGACGCTGTTTGTCTCTCTTCTGACTTTTACTCTCCGAGTTCACCCAGATCGGCTTGACTATGTGGATCAAATACTGGTACGGATTCCTTCCATGGCCATCTTTCCTAAACTTGTGCCTTAGTTAATATTTGTATGATGCATTGTTTAGGTTTACGATTAGTGGAATCTGGTGTAGCCATGGTGTTAAACTAACTCTTGATCCGCAAAATTTGTAGGGCGCATGTGTAGTTAAGCTTTCCAGTATGCCAAAGCTGGAGGACGCCCGTGCAATGAAACAAGTTGTTGCACTTCTGAGTGCCCCTTTAGAAAAATACAATGACATAGTTACAGCTCTTACACTGTCAAATTATCCACGTGTCATGGACCATCTTGATGATGGAACGAATAAAGTAATGGCGATGCTTATTATTCAAAGTATAATGAAAAATGATTCGTGTATATCAACTGCTGATAAGGTATACTTACTAGTTACTATTTTCTTCAACTAAGGACATATATATTATCTACTGTAATAGCATGCAATTTGTGTTCACTAGGTTGAGGTGCTGTTTGAATTGATTAGAGGACTTATTATGGATTTGGATGGAACCGACACAGAGGAGGTGAGGTCACTTTTTTCTTGGTTTCTCCCACGTGGGTTGCAATGAGTAGCTCTCGTCAGCTTTAATGTAGCGTCTTGGTCCTCATGGGTTTCTTGTGTTTGCATTTAGCTTGATGAGGAGGATTTTCAGGAGGAACAAAATTCAGTTGCCCGACTGATACATATGCTTGATAATGAAGAACCAGAGGAGATGCTTAAGGTGAAACATTATACATATACTAAACTTTCGGTTAAATAGTTATATGGCATTATGTTTCGGACCTACTGTCATTCCCATGATTTTAGTAGATAGCTATAGAGCTGTTACCGTGACTTTTTCATTTTAACCTGGACATGGTTTTTATTCCTTGGAATCGTGCTTCTGAGTCTGTTGCTCCACTCCCCTGAAGTTGCCTGCATCCCAAAATTGACCTGCAGGTCGGCGTCAGGCCCATTGGCGATCTTTAACAGAAGTCATATATATATATATATAAGCATGAGTAAGCACATTGTTTACCTGGGTGCTTTACTGATATGCAGATTATATGTGTTGTGAGAAAGCATCTTATGACAGGAGGAACTCGACGCCTACCTTTTACCGTTCCCCCACTTGTTTTTTCTGCACTCAGGGTATATCTCTTATTTTCCCTATAACGTGCTTGCATGTGTGTATATCCAGTGTCTTATATGTACCCCTTGCACTACAAACCCCATCCAGTTTCAAGGCAAACATTGCTTCGGAGATTTGTTGTGCAATGCTATAGATGCTCTCAAATGATGTTAAAATACTCTTATCATTGTTTCTTTCTGGCATTCTTCTTGTCAGTGAAAAGCAAGTTTTATGTATTTATTCGACGTGAATTACAGTATAATGCACGTTTTAACATATGCACATAGATATGTGGCATGCTTTCTCGTGTGATACTACTCCATGATCACTCCAATGAGATTAGATTCTCTGCGTTTAGAAATATGGTTGACTTGAAGAATGTCTTTAATGTTTCCGCCACAGTTGGTCAGGCAGCTAGAATCCCAGGATGGAACAGTAACTGGAGAAGATGTCCCAGCAACCCCGAGGAAGATTTTTCAGATTCTCAATCAGGTTCAGTGAGCTTTCTTTTTTTTTCTTGCCCATACATCTAAGATAGAGAAACAACTGTTACTTTACTGAGGCTGAAATTCCCAGGAGTTAAACATTTATTCTTGTGAATCGTTTATCTCCGTGATGAATTCAGATCATCATTTGATTATCAATGCAGGAGTACTTTCTCTATGTGATTGACTAAAAGCACTGTAGTGTGTATTTAACATATTAGATTCTTCGATTGTCCGAAACTTACACACACTCAGGCTATTTGTGTTAGTTTTGATTTTTCTCTAGGAGCTGCAGTATGCGATAAGTTGTTAAGACACATCATTTTTAATCCCCAATATTTTTAATAATCCATTGAACATGTGCTTTTTGTTCCAGACAATCGAGGTTCTCTCCTCGGTTCCATGTCCTGAACTGGCTCTAAGGCTCTACCTCCAGTGCGCTGAGGTAATATATATGGGGTCTCAACAAAATTCAAATTTACCTGTAGGCAAGGTGTAATCCGACATAGCATTTTCAACAAATTTGTATTGTTTTGGAATGATGATCAGGCTGCAAGTGACTGTGATCTTGAGCCTGTTGCGTATGAGTTTTTCACCCAAGCATTCATACTATACGAGGAAGAGATCGCGGTAGTTTATTTTCCTTTCTGTGTGAACTTTATCCAACTAAAAGCTCTCTTCCTCAATTTTTATATACGTTCGCTGTCTTGGGTGTGCTGAATCATTTTACTATAGGACTCCAAGGCACAAGTCACTGCAATCCATCTGATTGTTGGAACCTTGCAAAGGATCAATGTCTTTGGTGTTGAAAATAGAGACACCTTGACACATAAAGCAACCGGGGTACAATTTTTTCGCCAATGCTTTCTCATTTAAGAAAGCTTTCTTCTCAACTTCTATCTAAACACCTAGTTTTGTGTGTTTTCCTTGTGACCAGTACTCAGCAAGGCTTTTAAAGAAGCCTGATCAGTGTCGAGCGGTTTATGCATGCTCTCACCTCTTCTGGGTCGATGATCGAGATGGCATCCAAGACGGAGAAAGGTATCTACCTCCTCTTTCATTACTCTCATACTTCTCTTTTTTTCTTGATTACCATGGGTTAGTCAGTGATCTTCTTTGTTCTGCAGAGTTCTTCTCTGTTTAAGACGTGCACTGAGGATAGCAAACGCAGCTCAACAAATGGCTAATGCAACCCGCGGAAGCAGCGGACCAGTCACGCTGTTTGTCGAAATCTTGAACAAGTAAAAAAACATCTAAACATTGGTTCATACACAAAGAAGCATATCTCAATATTAACAATTGGTTTTTTTTTTCAGGTACATATACTTTTATGAGAAATGTAATCCACATATCACTCCGTCTGATATACAGAGTCTCATAGAGCTAATCAACACCGAGATGCAAAGCGACGACAACGGTAACAGAAGAATCCACTCAGATCCCTTCTTTACAAGTACATTGCGTTACATAAGGTTCCAAAAACAGAAAGGTGGGTTGATGGGTGACAAATATGACCTCATCAAGTTGTGACTTGTATCCTCCACACAGTCCTGAATCAAATCCATAAGAGCCAAACCCATTTGGATTTGATTTGCTTTTTTTTTTTCTGGAATTTCAGAAGGAATGGTGTAAAAACATGTTTGGTTTCGGGTCCATGACAAAATTGGAGAAACCTTTTTGTAATTTTAATCCTCTTTGTTACTAATACTACTCCCCTATGTGTATAGAATTCTCGTTTCTGAAAACCATTCTTTGAATCTATGCTGCAATTACGCAAAATCAAACACAATACATTGTTGGTTTTTTTTCTTGAAAAAATGATTTGTCATACAAGTAGACGGTATCACAATCACACATCTAATATTTTTAATCACACAATTCATAGGGTTGACATATAAGTAAACAAGTGTTGTGTGATTAACATCTTTCTGATAGTAATTGACTGAATGTAGTTCTTATCACATGTCAATACCAAAAGTAAAGATGGATAGAAAAGACAAAAAAAAAAAAAAACTTGAGCTTACAGTTTTAAAAAAGAGAAGAATTACGATGATAATATCAATTAAACTCGTCTGTTAAAAGTACAAAATGGATTCCAGCATCATTAAAAAAGAAAGATGTATTAAGAGATTAGTCTACAAAGCAAAAGTACAAGAAGAACAACACACCAAGAACTCATCAACTTTCTCTTAACTCAGAATCCTCTTGGCCTCACTTCAAATGACTAACAAGGCTGCATTTGATGCTAGACTCTCTTTTTGTTTGTCTCTTCAGCTTCGTTTGTTACCTACAAATCTTCTTGATTAAAAAAAAGGGATGAGGGTCACAATAAAACACAAAGACTCTTGTTCAATTTCTTCTTCTTTTGATTCGGTGGCTGAAGCACCATTTTTATAGCTGCGTCAAAGACTGCCTTCACGTTCTGCACAAACGTTTTTCAGTCTGTTAATACATGGAAAACCACATAACAAGCGTACATCTTAGATCCTATGAGTGTACCTGTTGAGTCTTTGAACTACACTCAATGTAAACCGGAGATCCTATCAGTTTCTTTAGTTCTTCTCCCTGCAGAGATACAGTTAAGACACATCTCATCTAGTTGTATCTGAAGGAATAATTGGATTGTGGAGGTTACCTACCTGGTTTGTAGTGATGGGCACTGCACCAGGATGGTCTATGAAGAACTGCTTGTCATCTCGAAGATCTGAAAATTTCTACAATTTAAGTATTTTTTTTTCTTGTTATCAGGGAAGAGTTTTTACAGAAGAAGAAGAGAGTCAGATACCAAGTTTTGTTCCAACGAGGATAATGGGAACTCCAGGGGCATAATGCCTGAGCTCAGGAATCCACTGGAATAAGAAACCAATTTCAGAATCAGCTCACCAAAAGTTAATATTTAAAGAGGAAGAAAGTTGGAAGAAAAAAGAGACAAACCTTCTTGGCTATGTTCTCGTAGCTAGCTTTGCTAATAAGAGAGAAGGCAAGAATGAAGACATCTGCTCCACGGTAACTCAATGGTCTTAACCTGTTATAGTCTTCTTGACCTGCACCAACCCCAAGAAGAGTTAGTCAAAAATGAAACAAGATTTGATGGGGTTGGTTTCAAAATGATGGTTCTTATACCAGCTGTATCCCACAATCCAAGATTCACTGTGTTCCCATCGACAACCACATTAGCACTGAAGTTGTCGAAAACTGTTGGTACATAGTCCTGATTAAATTCAATAACAACTAAGTCATGAGATTTGGCTTCAAAAAACCACTCTTTTATATGTAATAATCTAAACTAGTTCTCAAAACAAGTTTAAATGGATTATCATCATTACATTAAAAGGACAATTAATTAAAACACATTTTAAAATAATTTACCAGCTTAGCTAAATTCAAGAACATTGTTAAAGAAGCTTCAAGAACACTACTGAGAGCAAAAAGAGAACAAAGGAGGAGACTTTACCGTAGGGAAAGTGTTGCTGGTGTAAGAAATCAGCATACATGTTTTTCCGACGGCACCATCGCCGACTGTGACACACTTTATGAACCTCGAAGCGCTCATCTCCCCCCGCTAATTCCCCAAAAAAAAAAACAAATGAAAGTCTTCTTCTTTCTCAAATCTCAGCCATTGCCAAGAGTCAAGAATCAAGAATCAAGAATCAAGATACAAACCTTCCCCTAACTCTAGAAAAAAACCTTAAAACTCAAATGAGAGATTGATGAAAGAAGAATGATAAAAATTTTGTATTTTTTTCTCTTTTATTCTGGAGTAATATTTTTGTCTGTTTTATGGCAGCTAAACGGTACACCAAACAACACCTCTTATCATTTACACTCACCCCACTTTAATTTTCTATATATTATTTTTTTCTAAAAAAAATTCGAATTTGAATGACAAATCATATCCGTTGTAATTTACATTATGAAATAAATTTAATGTCAAAGATAGCCAACTACCACCAAAAGAACAACCTGACACACTAGATTATTTGTTAAAAATTTGATTATAATCCTCTAATGACCACGTATCGTCTAAATGAAAATTACAAAAGCAAGAAGACAGGAAATAAATTAATATTATGATTAAAAACTAAAGATAAAGCATAAGCGACAAAAGTATGGGGGAGGCAGGATTGGGATATTAATCATTTGATTGGGAACAATTCTCGTCAAGAACGTGCGANNNNNNNNNNNNNNNNNNNNNNNNNNNNNAAAAAAAAAAAAAAAACCTCTTTCGAATTTATATTATACAGTTTTATTTTCGTGAGTATTTTGTTATTATAATGTTGTAAGATTTCTTCAGTTTTACGACGTATATAATTGATGAAAACTCCTTCAAGAACGTGCTTAACTCTCATTCAACTTACCCGCCAATGAGCCAATCATACGGTCCGTCGCATTATTGCATTATTTTATATAATAGGCAATCAGATGAAGCTAACTAAAGTGTTTAGAGACTTAGAGTTAGCTCTACGCTTCTACCTACTCTGCTTCACCAACAACACCTTCTCACATTCATTACATGAAATATATTCCAGAATTTTGGAATTTTTATTTTAAACTTAACATGAATAATCATGAATTTGAACGCTTGAAAATTTTATAATTGAGGCCACATATTTGGAAAAGATGTAGTGAAATGAATCCTTCATGAGTGAGTCAACATAGTCAAGGAACATCAATATTTTTTTGCTGGAGGATAAAAATTGCCAATATCGGAGTCCGTGGTCCGTGTTACACAAAGGCCACAAAACAATAAAACGAAGCCTTACTAGAGGCCTAGTATCGAATTTAGGCCCACTACTATACTCCCGAAATATCAATGATCATATGATATATTCATAACGAATGAGCATCACATACCATAATGAACGAGGGTTAGTCCAAAAGGGATAGTCACGAAGTCCTCATAGAAGAGCCCCCACTTACAGCTAATTACTACACATACTGTGAAGCGACTACGTGTATGCTTGTTTCAAAGGCATATACACTTTTTCTAGGTTATTTGTGATTTTCAATCTTTTCCCATATGGTTACAGAAATATGTAAATGTTCTATCTTGCTACGGATACTTGGCAAAATGTTCCGCAATTAGAAGATTAATAGGTGTGGACTAAAGTTTGTAGGCTTCTTATTCATTTAGCATTTGCAAAAAAAGGAGAGTGTTAAAGTATGACAAGTGCAAACAGTTTGCATGACAGATGGCCCATAATTATGTCCAACAAACAGGCAGTCCTGAAACTTAAAACCATGGTCGAGACATTGGGCATATATGTAATTTAACAGTAGAGGTTTTTCCATTTTGGGTAGAGATGGCAATTTGACCGCAGCCCGCGGGCCTGGCCCGTAGAAGACCCATGCGGGGCGGGTTTGGGTAGAGCTATTGTAGGCCCATTTATTAGCGGGTCTCGCGGGTTAAGTCTCATGCGGATTGGGCTTTAGCAGTTTGGGCATTGTGGGTCTTGCGGGGCAGAAAGAAACCCGCACCAATTCCTCTTTTGTTGTGTCTCCACCTGAAAAAAAGAGAGAGTGCGAAATGACGATTCATGTTCTCGTGACTCACGACTCCATCGACGAGCATCAGAGGAGCATCACCACGAACCGGTTCTTCTTCTCCTCGACTAAGGCAACCACCAGATCTATTAATCTTTTGCTATTAATCTTTACTAACTTCAACTATTGATATTTTCAGATGATGAAATTGGTGACGAAGAAGAGAAATTACCATCATTTGAATCCATTGTCAATGGTGAAGATGAAGAAGAAGAAGTTTGATTTTTTATTTTGATTTGGTGCTTTGCTTCTATCTACATTTAGAATGATTGTTTGGATTTGGTTTTTATTTACTTTTGGGATTCATTGTTTGGATCTGGTGTTTTGTTTAATTTAAATTTGGTTAAGACTTAAAATTTTTTGCAGTGTTTGTTTGTGCTTTTGGCAATGACTCGTAAAGGAGCTGTGGGAGTTGGTAAGAAACTCTCGGAGTTGACTGATAACTTGATAAGGGCTATATTGTTGATAACGAAAGAGATATACACATCTCTCAAAGTCTCAATTATATGTATACAATGAAATCTAATATGTTTAACACTTGACAAAACAGAACAACCAGTACTTGCTTTGTTTTTGTTGGTAATGACTTAATATAAAATATAATGCCTTTGTGTAGCTATGTTTGTTGATCAACGTAGGATAACATGCCGTTATATATTCATCATGACTTGTCTCTTGTTTGTTAGGTTCAGTGCTTTGCTCTTGTTTAGATTTTTTTTTAATTTTGATTAATGTGTTCTCTTGTTTTGATAGGTTTAGTGCCTTGCTTTTCAAACAACAACAAAGTCCTATGTAGCAGGCGGGACGGGTTTGGGCAGAAGTTTGAGAACCGCGGGTCACGGCGGGTTGACCCGCAAAGATCCGCCAGAGATAGAGACCGCAGCGGGGCGGGCTACGGCGGGGCGGGCAGTCCCAATTGCCATTCCTAATTTTGGGGAAGAGTTAGTTGACACATTCAACAAATGATTTGAATTTCTTTAACTTATATCCGCATCTTACTTCATATAAACTAAAGATTTTGTTTATATAAAGTTATAAACCTAAATGTACAAATCTTTGTTTCATACAACATAACACAATCTAAGCATTAACATTCTATCAGTCACGTAAACTAACACATTTTCTGCGGCAATATGCATATTCGGTCTATGCTATTCTTATGGTACATAAAACTATATTATAACCAAACCAACATTAATATATCTGGTACATGTCACAGAATCACTCATTGATAGTGACAGTCTTATTTTATGTAAAATACACACGATACAGACCAAAGAGTTTGTGGATAGATTAACAAGGGAAGTTGCATACACACTTATTAATTGGGAAGGCAATGTTGCAAGATCCATATCGTGCTTTCTCAAAGTTAATGCACTGATTCTTGCACGCTTTATTGTCTCGACAATTTCCTGATGACCATGTCCTACTTGGCTTCTCGCAAAGCTTATGTGCTTCCACCTTGTTGGTGCTTCTGTATATACATATACCTCACAAGGTTAACTATCAAATACTATGAAAAAGGAAAAAATTAAAATTTCCTTGCATGTGATAATTAGGTTATTACTCACCAAAAGCAACAAAAAGAACAAGAGCAGCGAAAAGAAGAGCGATGATGGAAGCAAACTTAGCCATGATCACTATTAATACTTTAGTTTTCAATGTATATATGTGTGTGAAGTGTGAAGGAATAAGCTTAAGAGCAACCTCTATTTATAGTAGAGTAAAATGATGACGAGTTGGTTTTCACTACTCGTGCATCTTACGATTAAGTGTTTAACCCCACAATTTAGGAAAAAAAACACCGTCTTCTAGTATACAATGTTAACACGCAAGTAGCCATGATAGAAGATAAGATGTTAACCAATACATAAACAACACATTGTCAGACTTTGAAGACTCAGGTACTCTTTGACTTATAATCAGTGCCTGGTTTTACTGCTAAACTGTTCATTTGCGATTCGATTCGATTCGGTGGGTAAAATGACAGTTCACATAGCCACCTTATTTGACTTGGTCAAGAAGTTATAAGTCTTCACCACGACCACATAGCATTAATTTAGGTTACACAAGTGTTAATGACTAGTCACGACCACATCGCATCTAATGGGTATTTTTCTTGCATTTCTTACAATCAACAAACTAATAACATGATTAATGCATGTCTCTTAGAAAGAGTCTTTTAACAAAAATAAGAGATAATCTCATAGCTTTTAATATTAAATTTTAATACTTTGAGAAATTTTATGAGAACCTTCTATTGGAGTTGTTTTTATATCCGTATATCTGACTATTCCTTTGGAATGGAATTTTTGATTATATAAACCTAATTGTATGGATCCTTGCATATACATTTTATCAGTCACGCTAAATTGACAGATTATAAAAGTGTTCCTTGATTTGAATACAATTTCACACTTATTTAAACAAAAATTGACAGATGTTTAGGCAGATATAAGCATTCAGTCTATGCTATTTTATGGTACGTAAAACTTTATATTATAACCAAGCCAACATAAACATATCTGGTACTGTGGTACATGTCATAAAGTCACTCATTGATAGTGACACATACTTATTTTATATAAGATACACACAATTAACCACCAAAGAGTTTATGGATAAATTAACAAGGGGAGTAGCAGATGCACTTGTGAGCTGGGAAGACATAGTTGCAATCCATGTCGTGCTTTCTCAAGGTTAATGCACTGATTCTTGCATGCGTTATTGTTTCCACAGACTCCTGACCACGCCCCACGTGGCCTCTTGCACAGCTTCTGTGCTTCCACCATTGGTGGTGCTTCTGTATATACACACATTTCACAACGTTTATTGTTATATAATATGAAATTTGAAATTATTTTAAATTACATGCATGTATAGTTATTAAGTCATTACTCACTGAAGGCAGCAAAAAAACAAGAGCAGCGGAAAGAAGGGCAACGGTGGAAGCAAATTTAGCCATGATCGTTATTTATACTTAGTTTTCAATGTATATATGTGTGTGAAGTGTGAATAAATAAGCTTAGGATCAACCTCTATTTATAATAGAGAAAAATGATGAGGAGTAGTTTTACTCTAAACATGCATGTTAATTACGATTAAGTGTTTCACCCCCACAAATTAAACAAACACCGTCTTCTAGTACACAATGTTAACACGCATGTATCTCATGATAGAAGATAAGATGTTGACAATACAAAAACAGTGTGCTGATCGGTGCATGGTTATAACTGCTAAATTGTTCATTGCAATTCGAATGGCTGGCTATAACTACTGTTCACATAGGCCAAGAAGTTATACTTATATGTCTTCACCAAGACCACATCGCATTTAGTTTGGTTACACAAGTGGCAGTGATTTGTCACGACCACATCGCCTTTAATTTGACTTTGGATATTTTTCGTGTTATTGAGCTTACAATCATCAAAGTAAAATTCAAATGTAGAGTTAATCTTAGCTAGATGGTGTGGACTAACGCATTCCGATATTTTGTGTAAAATATTTCAAATGACTATTATTTTGTTATGTAATTTGTAGTTCCCACTCTCAAACTAAACTTTTGTTTAATTCGAACCACCTAAGAGAAATCCAATTTAAAAGAAAATAAAAAATATAAAAAAATACGAATTGAAAGAAAAATTATTTTCAACTTCATCGCCTTATCTGCAAGCTTTGATAATAAAAGCCACGTACCAAACAACTTGCTTCATTGCCCTACTAAGACCATCTCCAACCATAGATATGGTGTTAGAGTTCTAAATCAAAATTTTAATTGAAAATAATCAAATTACAAGTTTTAGATACTTGTTTAGTTTTAATCCCTCCAATGGTAAAACCGATTAAAGGTTCTAAGTTTCAAAAATTTGTAAACTTGTATTAATAGATTACTTGCATCACATTCTATAATAAAGAAAACTTATAATTATTGAATTACATAAAATTTAAGTGAACTTGACATAATAACATATAACATAGATTGACAAATCATAAATAAAACTAAACCATTAATAGTTGTTTTGCCTTGTACCAAATTTACTCCATATATGCTCAACCAAATCATTTTTTCAATTGTGCATGTATGTTGCGATCACGAAGTTGAGTTCGAATTGCCATCAAATTGCCAGTATTTGAAGGCGAAGACGTGAAATCCACATGAGAAATTCTGTTTGATTCTACTTGTGTGAATTTTGATACGTCAAATTGAGTGTACCCATCTCGTTCGTCTTCAACTATCATATTTTGTAATATGATACATGCTCTCATAATCTTTCCGACTTTTGGCTTATCCCAAATAAGAGCTGGGTTTTTAACAATGGCAAATCGAGCCTGCAAGACTCCAAAAGCACGATCGACATCTTTACGGACAGATTCTTGTAGTGTAGCAAATAAGGATGCTTTCGGAGTTTGTGGAAGTGAAATAGATTGGATAAAAGTTGACCAGTTCAGATAAATACCATCGATGAGATAGTAAGCCAAATGATACTCATGTCCGTTGACAAAGAAATTCACTTTTAGAGCTCGACCTTGTAATATATCATCAAAAACTTATCAAGAACATTGATATCGCTTGAGGTACCTGGAGATCCAAAAAACGCGTGCCATATCCAAAGATCGTGTGAAGCTAGTTCAAAATCAGAAGAAGAATTAGTAACCTAGTTAATAAATCAGAACATTTTTGGAACACAAAAACAGGAACTACACTCTACCAAGCAATTACTAACCTAGTTAAAATCTAACAACCATTTTCATACTGAAGAAGAATTGATGTAGACAGTCATACTTTAACATCAACCAAACATTTTCAGAGTTAAATAACGAAGCATTGATCACAGCTAAGGAGAAGTGATCCGATTTAACAGCCTACAACCATTGATCACAGACTAAGCTTTGATCACAACCACTTCTCAAATGACACAAACCAAACATTGAAGATCACTAGCAGAACGAGATATATGATTATCATGATTTTATAAACTCAGTAACAAAGATATAGCAAAATCAGAAAACTATAAAATACAACAAGAAATCATATAATGCAAATCGGATTCAAGAATCAACAAAACCAAATCGTACCATGAGAAGAATGATGTTGATAATAGCTAAAAACAAATAAATATCCTACTTATTGATGTTAATAACCCTAATTTCGCAGTTAAACCAAGAACTCTAATTTAGAATATTATGAACCCTAAAATCAAAATACCCTATTCTAGATTCAACAGATTAAATAGACGAATCTACACATTATAAAGCTTCAATCGTACCTAGAGAAGAATGAAGAAGCTCAGTGCATCGAAATCTCTTCGATTTCAAGTCATAAATCTGAAACAGTCAAGCCTCAGATTCCACCAATTTCTCCGTTGATGAAGCCGGTGGTGGAAAGGATGCTTCTGGCGTTGGATTCTAGGCTCAACCGGCGGCAGATTCGATATACAACCGGCGGTTTCATGATTAAGCGACGATGATTCGATTCTCCGTCTCAATCGTGAATTGTGGGAACCGAAATTCGCCTGTCAATTTTAGTTGATTTGGAGGAAAGCCAAGTGAACCTAGCCTTCCCTGAAGGTCCCGGTTATCTGCTGGGCCACACACGACACAATCAATACGACGGAAAGATAAAGAAATATGAAAATCGTAAGAAAAAGATAGAGCAAAAGATCTTATTTCCGAATCCGCGTTTGAGCGTGAACAACAGGTAAGAGCCTCGGCTACAAGGGCTGTCGGCAAGTTCGCTAGTCTAGCAACCTAAGTTCTAACCTAGTTGAGTCGCAGCTCGCTAGTAAAAACGGAAAAAGTGCCTAAGTTGCTCTAAGTGCTATATTGCTCTCCAAAAGTTCTCCTCTTGCTCCTTGCCCCAAGCTCTCCTTATATACTCCTCTTAAGACGGTCTATTTCTCCTTTGGGCCGAATCTGTCGTGAATCGGGCTTCTTCCATTTTCGTCGGTCTTCATGCTTATCGGGAAACTTGACATTTATCTTCAAGAAATCTGACATTTATCTTCGGGAAACTTGACATTTATCTCTTTATGTAAAGATAAACATGGACCGTTAATTCCATTTCGACTTGATCTCCTGGAAAGTCGTAAGTGGGCCGCTTGGTCGCGTTTGGGCCTTCCAGGGCTATTGCTTCGACCGTGGACGCTTTACGGTTTCGCGAGAAACGATCCTGCCATGGTACTTGTTTTACCAAAGGAACCCCAACTCCTCGTATAGTCTAGATGACTTGGTATTTTCAGAATAACCGTGATCGTTTTATACGATGAATCGAAATCCATTCGTCAACCGAACTTTTGGTGGTCTTCCGAAGCCTTCCCGATCTATTTCGACCAGAGCGAAATGGGGTTCTCAGGTTTCATGTCTTCGAGGCGTTGAACGGTGAGTCTTCGAGCAAAGATACAAAATATAGAGTTGGACTAGTTGTCGAGACGGTTTTGCGGGGAGAAGGTCGCACCTGTCGAGTAGTCCGTATGCAGGCGCCCGTGACAGGATGGACGAGCTGGCACCCGGCACCCGTGACCAGCACGGGTGAGCTGGCGCCCAGCGCCCGGGACCAGCGCGGGCGAGCTGGCGCCCGGTGCCCGGGACCCGCACAGGCGAGCTGGCGCCCGGCGCCCGGG

scaffold 4

GGGAGGGAGATGGAATCTAACAGAGATCGTAGTATTTCTGAGGAGATAGCTTGGAGGTGCGGAGCTCGGATAGCATGAGAGCCGAGTACTTGAGAACTTCTCTGAGATTATTCGCGTCCTGAGAAAGAGACGAAGAGAAAATAACTCATCAGATCAAAAGAGAAAATTTTTTAAAAAAAAATTGAGTAACGGACGGACCAAGGCGCGATGCATGAAGAAAGCGTTGTGCTGGATTCCGGCGATTCCTTCCGCCAGCCACTTATCCTCGTCTTCTACTCCGGCGAGCCTCCTCATCTTCTTCTTCCTCCCTGTGTCAGCAGTTGAGCCCACGAATAATAGACTCGAGATTTTCTTTTTCGATTTTTAGATATCTTTTGGTTTTTGTTTTCTTTTAATTTAAAAATCTCAATTATCAGCCTATATTAGGTAGACTATTTGCGGAATTAATATGATTTGTATGTTTATTATATAGGTTTTATCGTTGTAATTATGCTTATAGTTTTTTGTAATATTAATCTTGTTTTCATCAACTTGTGGAGGGTGAAATATTCACTCGACACTAATCAGTTTTTCACTTTTGTAAATATATTTGATGAAAGTAATATAATGCATACTTGTTACGCTATGAAGAAGAAGCGAACCGTATCAAAATTGGGTTTAGATTTAGAGAACTGTTGTTACAATCAGTTACTGTACACGAGTAAACAAAACTGGAACACTAGAACGGGACAGTGTAAGGTAAAGAATAGATCCCGACTTGAGGCAAAAACTGAGCTTTAGGCCAAGACGAATCACTAGATGTCCGCACGTCTTCATCATTCTTGACTGCTGCTTTAAAGTTTGTTCTCTCTTTGGTCTCAAAGAGCCAGTCGTCAGAGGAAGAATCAACCTCCATGGCAATGGAGGAAAGTGGAGTCCAATCATCAAACAATGCATTGTATCGATCTTCTTTGCTCGGCTTATGTTTCTTTCTTTTCTTTGTTTCAGAAATTGCAGCCTGAGATGTTGACGGGACACTGAGTTCAGGCTCACAAGCCTTCTTTGCTTCTTCTATTGCGGAGGGCACATTCAATACAGGCTCACATACGACAGCTTGCGGTGCCTTATGATTGAGAGACTCAGCACCGGAAGTGGAGCAGGTAACAACATCCTCTCGGGGCAGAGAAGGACTTTCGACCTTTGGTGGTTTTTTGAAGGTCAAACGAATACGTAGAGGTTTACCGGCTACAGGAGAAGCTGCAAAACACAAGTACAAGTTTACATGTGAATATCCTAACACAAGATATCCATAGACTGTGTCCATCAAAGTATATTGAAACACACCTTTGATGAGACTTTCAACAGCCGGAGGAGAGCGATCTCTCTTCCTCTTCTTACTATTCTGGCTTCCATCAGATAGATAACCGAGTTGGTTTTGAGGCCCCTCAAGCTCCTCGGTCACACCACTCTTCTCGGACACGCCTGGGGATTCAGTGGACGGGCACAAGAGTTTTTGGTGCACATCATCAGTTTTCTTGGACGAATGTTTGTGAGACTTCTCACGCTTTTCTTTGTGTTTTCTCCTCTCCTTCTTTTCTTGCTTCTCTTTCCGGAGAGCCGTTTTGGAATCAATTACGATTCTTTCGATCTTTAATTCATTAGAATAAAGGGTTTTAGGCTAGAGGTCTTACAGGATCAGTTACAACCATCCCACTCAATGAGGAAACACGAATATACCACACAAGACAGCAAGTAACAGAAAACCAAAGGATACAACTTATAGAATCCTAGACCAGAGGAAAAAACAATGAAGCTTACCTGATCTTTGACGTGATCTAATGAAAATATAGATGTATATAAGAAAATTATATGTTTGTAAACATGCATACTATATTAGCTAATGCAGTGAAAACATAACATATACATATATATATGCTAGGTAACTAAATCATTGGGAAAAACGTAGTTTTATAGCTGTATTAGTGAAAACATGCATATTGGATAGGATAATTGTATGGTTTATAGCTANNNNNNNNNNNNNNNNNNNNNNNNATATATATATATATATCTATATGATAACTATATGGTTTAAACTAATCTATTGAAAACATAGTTATATAGAGATATTAATAAATCTAGGGTTCATGATGCTATATGTGATTCTCAAAGCTACTGAATGAGAATCTAAGCACAACTAAACCAACAAGATTCAAGAATCACGAAAATTATACCCTAGAATTCAATTTCAAAACAAAAAGAAACAAGAAACCAGAATATGTGGCTTGACGAACCTTAGTCGATTCAACGCTCGACTGGTTCCTAGCAATCACAATCGATGGCATGTTTTCCCCTTTTCTTAGATGGAATCGGGAGAGTGGGAGGGTTTGGGTAAAGGGAGGGCGGATGTAATCGATTCAGATCTAATCAGCCAGTACGAAGTTCTCGAGACAATCCTGCAGCTCAAGCTCTCGATTTTGCAGCCCAAATACAGAGAAGCAACCTAAAACCTATCTCTCGACATTGAGATAGAGGCAACCTCGTTCCGTATTTAAAGACGTGTAAGATACTCAGATCCGCCGACTCGGGCAAAGTCGGTCCACTTGGTGGGTTTTTTCGTAATTTCACGTTAGAGATCCAGAAACCCTAATCGTACAGGGTATATGAGTAAATTGCAATCTCGTTAGGCTCGGTCTGGCTTCCGAGAATCTCGACTAATGGGCCTAAATTTAAATATTAGGCTTTACGACTACGTTTTGAAAAGCCCAAATATTTTTCCAAACGATTAAGCTTGTTTGCCGAAACGTTTGTATCATGCCGAGAGGATAGAGATACGTGTACAAGTTGTAACCTCTAGCCCAGCTTATAAGTGAATTAACAAAAAAAAATTTTTTAAAAAAATCTTCACCTATTCATTTGATTGGAAAATGGTATTTGATAAAAGATATCTTTATAAATAAAGTTGAAGTATCTGCCTTCTCCTGCGTTCACATCGCTCTGGAGAGGAGGGGTTTTCTCTACCACGTGTCGCGCGCTGAGTCGTCTGTGTAGTTCACACGTTCTTTTTTGTTGCGCGCGTCGGAACCCATCTACTTCGAACTATCCGCTGCGTTTCTTGTGCACCGTTCACGTTCGCTGAAATCCTCAGACCACGGTTTCGGATATGTAACGATGAGAGTATGTCTTTACTTATCACCATTAAATATTCCCTTGATTTTATCCCACTACGATTTATTGAATCACCTTCTTCTATAAAGTTCAGATGAAATCCCTAAAAAACATTCTATAAAAACCGTTTAAGAAATCTACGGATCAATCTGCTTTAAAAGACTATCATCCATTTCTTACAAGTTACAACACAAACTACAACAATGGTGACTTACCAGACCTTTTTCTCTGATGTTAGCACCGGTCGTTGTTCCGGCACGGTTGTCACGCGTCTTCTGCGATTCTGGGAAACCGAGAGGGAGGAGAGCTGATTGGATTGGACATGCTCCTTCTTGATCAAAAGGTTTGTGTCCTGATAATCCATCGCCTTCAAAAAAAGACTACCTTATTGTCTAACATACCCGACTTCTTAATCCTTCTATCTTCCCTTCATTCGTCACAAATTTTGCACTATATACCCTGTTCGAAAACGCGTTCCAGGCGGCCGGAAACTCGCCGGAAAAGCGTGAGGCGATGGCAAACTGTGGCGATTTGCACTGATTCGGCTTGTTCCTCGGAATTTTTTTTTAAAACCATTGTATTGTAGGTTGTAAACCCAGAATCACATATTATATCATAGATCTGTTCAATATATGTTCAAATCGACAATTATCAACCCAAAACATTATGAAATCGTAAGAATATGTAAAAAAACGGCTGCAAAAATCGCAGGTATGGAGGTTGAAGAAGAGGGTACTTTTGTCATTTCACGTTTAATTAAACCTAGTTCCCAAAAAAGCCTTAAAAAGGTTCCACCTACCTTCGAACCCGGGTCCTCTGCGGCTTTATGTGGTAGGCAAACCACTAGACCACGGAATAACCTTAGGGTAGTTTTCACAAATGTGATATATATACTTTAAATTTACATAAATACCCTAAAATTACTCCCCGATTAATCTCCGCTTAATCCCCGATTTTCTCGTTATACGCTAGGCCCAGCCCGAACGTACGAGGAACGCCTAGCGAGTTCTTGAACATTGACTATATATATGGACTAGAGGGCAGGAATCCACATATCTCATCGTTTCTCACTTTTCTCCACTCCAAAGCTCTCTCTAAGGGCCAGTTCGATTATACCATTCCTCAACTGGTTCTGTCTGATTTAAAGGCTGGTCTTGTCACTGCTAAGTATAAGAAAAGTGATAACAAAAGACGACAAGTCGCTGATAAAGTTATTCCGAAAACAATTGTAAGTGCTTTGTAGTGATAACATCTTAAAGATGTAAGGTCCTGTCTTTTCAATTTCAGTTTGGTGTCAAATTGATTGATTGTTTGTAATTAGTTGTGTAGATGACCATTGATGATGTCAGAGAGAAGTGAAGATATTGGGAGCTTTTTCTGGTCATGACAACTTTCCTCATTTCTATGATGTATATGGGGATCATGATAATGTATATATAGTGATGGAGACATAGAGTAAAATCTCTTTTAGTTCAGATGTTATTCCAAAATCAGCCTGGGAGAGTCTTACCAAGTGGTTTGTTGTTTACAATATGTGAAGGAGGGAGCTTTTGGATTGAACACTCTCAAGGTAAAGGGACCGTAAATTTCAGAAGTATGATTGTTGTTTTATTAGTCTTTGTTTTGGATCGCTTTATAATTGGACATGGTGGTGTTTTATATGCAGAGGTATGAAGCACACGAAGGAGGATGCAAAGACTGTTATGATACAAATATTGAATGTGGTTGCATTTTGTCATCTCCAATGTGTCCTACACCGAGATCTCTAACCCGAAGTGGTACAAAGCTCTTTACTTCAACTTATACATTTGATGTTTCATCGCTATCTTCTTGAGGTTTACTTAAAACGGTTTGGAACAGAATTCTCTTTGTACCTTTAAAGAAGACACCTCCCAGTTGAAGGCAATCGATTTTGGTTTTTTCAGATTACGTGAGACCAGGTAAGAATTTCTCAGATATATATATAGATTTCATCTGATTCAGAAGGTGAAATCTTTGTATATTTATTCACCATTATACGCCTCCTCTAACGGAGTCTTGCAGGAAAGTCACACAACATCAACAAGAGAAATAACCCAAATGTAATGTTTTAACACAGCTCTCTATCTACTCTCTTGCTCTCTCTTCCTAGGATGGCGGATGCAGAGAACAGCACCAAGGATAGGTAACTACTTCCGGTAGACATCTACAGATCTGATAAAGTGACCTGCACAAAGCTACAATCATTCATAGAAGAACTCAAGCAGCCTATCTTCATCACTATGTGGTCATCACCAAGTTACCTTTAAGATCATCTCAATGGTGATGGTGGATCATCTTGGAGACCTCACTGTCACCAGCGCCTCCCTCGCCTCCTCCTTCTGCAAGGTCATTGGCTTCAGTTTTACTGTAAAAGATTTTTATCTTCTCTTGGAAACTGCACCTCCTCATCACTTGATTGTTAATTCTTTCAAAAATATATACAAGCGGTTTTTCTTCTACGTGCAATCTTTGATTGGCATTTTTTTAATAGTTAAACAAACCCCTTACAACATTTGGTTTCGGTTAGATTAACTTTCTATATATATATATCAAACCTTCACCTAAATGGGTAAGAGTGTCTAACAGAGGTTACATAATTATATATGTGATACGTCTTTCAAGTTTCAACATCCCTTAAAGAAAAAAAGTGTGGGAAGAGCGATAATAAACTTAGCTACCCACATGATGTAAAACATCCATCTCATTTCCAGTTTCCAGTTACTTTTTTTTTTACTCAAATCAACTTTTATTATCCAATTCGTGAACACGGGATTACAACCACATAGATTTTTAACCCGACGTACAAACGACGCAATCTAGTAACACCTAGAATCACTAAGTTGGATCCTATGCTACCTGATAGAAGTTGTCGCTGTAACTTTCACCACAATGAACAAAGTTCATCTCACGAAACGAGCTGAACCAACTCTTAAACCCTAAAATTAGACCTCATAGCGGGTATAGTAACTCACAAGAAAGCCATGATACTAAACCGGTTAACCATTCTAATCCAACTATTGAGCCCCTAATGATTTGCATTCAGTACATGTCTCAAAAGATTCGACACCATAGGTGCATCCCGGTCAAACATCAAACCTTCCGGCTAGCACTATCCTGCTTCTAAGCCTGAAACCGAACAAGGACGTCTGGATTGAAATGCGGACCTTGACGTCAGCAAAATGGAACGCAGCACTCCAAATCGCAGCTAGCAAAAGGTGTGGAGACCAACCACCGGTACAACCACGGGGTCGTCAACGGCGCTGGGAAGAAAGTCTCCACCGTACTTGACCACAAACAGCCTCTCCTCCGGTTGACTCATCCGTTGTCGAAGCTCCTTGCTGTACGTCGTCTCTCCGAGAACCGAAAAGACAAAGCCTCTGTTCACCACCTATTACGCCACTCCTCTAACCATCTCAATACCACAAGGGTGAGTAGATCCGGTTTTGAAGAGATGTCTCACTGTGTCACGCGCCGCCGTCGTGACGTTAACGCCTTAGATCTAGAAAAGGGAAAATTGGATCTGTAGCCACCGTCGGTCCTAAAAGCCGACTCCCGCCTCCGCCGTTCTTCCCAGAGCCTCGTTGTCACTCCATAAGAAGTCATACCATCTCCGTCCACATAAAAGTCTCCGGGAGGACTCAACAGCACAGAGCTTCGCACATATCGGCCTGGATTGAGTGAGGAGAAGGAAGCAAAAGGAAAATGAGATTAAGGGATAAGGAGGTGAGCCTCCGACGCCGCCTAATCGACGTCGGAGCTAGGATTGCGGCCGTCTGGTGTCTTCAAGCTTCGAGAGAGAGAGGAGCGTCTTTTTGTTTTTCAGTGTTTCCAGTTACTTAAATTACGAAGATGCAAATCCGAGCTATAAAAATGTGAAAACTCTTAGAAAAAGAGGTATTAATTATTCTTATTTTACATTTCTTCAACTATATCAAAATGAAATAAAATAAAATAAAAAAACACTTTTACTTTTTTAAAAAAAAAAAATTTATGTTCTCTTAAAAAAATAGCGTGTTACTGATTCTTTCAACTCTTTTTTTAACACACTGAAAGATTTCATTCATCCATGAAGTTACAAGGTTGGATACGTTTACACACTAAAAGACCATCAGTACAAAGAGATGACTCTGGTAAGATTGCTAGCAAAAACAAAGGTTTTCCTAGAAACTAAGCCTTAAAACAAGGGAGATAGAGATACAAACTAAACATAAACAAGCGGAGGTTGGAACTCTAGACTAAAACCGATACATGCACATCGACAAGGATAACAAACTGAACTTCAAAGCAAACAAACACCTTGTGAACCTTTGATAAAAAGCCACCAAAACCATTGTTGATGACTGACTACTCCTGTAGAGAATAGCACATGCAAATTCAGAGCACGGAGCTTCAAAGTTCGGCAATCACGGGCTAAGGCGATGTCGAACCATAAACTACTGATTCTTTCCACTCAAACCAACAAAATTTAATAAAATTATAATATGATCTTAGAAAAAAAATTAATTTATTATTTTCAATTTAGAGTAGTTTTCCAAATATTTATGAAAATTAAAATATATTTTAATATAAAATAATTTTATTGCAGATCACCTGCCCGTAGGGCGAGCCAACCCCTAGTGTATTAATATCTTTGACTTATAATAAACGGTTTCAATAAGACATATATAAAGCCTAATTACTCTATTACAATAAGACATGTCTCACACACGCTCGGGATAATATAGATATATATTATCCCGAGCGTGTGTTTGTACTTCTTTTTTAATAATATAGATATATATGTTTGTACTTCTTTTTTAATAATATATATATATATATTATATGTGTATATATATCACATTNNNNNNNNNNNNNNNNNNNNNNNNNNNNNNNNNNNNNNNNNNNNNNNNNNNNNAAAAAAAAAGAATATGATTTTTTTGTTTTGTTAATAACATACATTATGTGAGTATGGATATATGGATGATGACACAATGAAGGTATTCTATTACATATGATAATCAAATTATCTGGTTGCATGTATGACATTTTTGATGGATATGCTCTTTCTGCAACAAACAGCCACAACAAACGGTTATTGAAACCTTCGAAAAGCTTGCAAGAAATTGCGCTTCATCTAAAAATTGAATCTCATATCATGTGTAGAAGTCTTTAAACATTGATCAATGCTAAAACTCTAAGCCCAAACAATAAAACTAATATTAAAAGGGCTAAATCAAGCCATCTTATTGCAGCCACATCAGCCATGAAAATCGTCCAATAGCATTGCAGTATTCCATCTTCGGCCAATGGGAAAAGAGTATCTCATCCACGTCATTTACTGAGGTGAAAACGTTGGGCTTTTGGGTGTTTGGAATTTTGTTTGGGCTGTATTGTTTTTTTTTGTGCTGAGCTCGGCCCATGTTCAGGGATATGTCCGTATGTTCTTCGCCACAATCTTTGTCTCCGCCTTTCGTAACCCTACTCTCTTCTTCGTGCCTTTGCGACTTCCATCTCTTCTATTCCTCAACACTTCAGATCCGTTTCGACCAGTAAATCAACAATAACTTCTCTGATGAATCCATTGATTTAACTCATTCAGTGGTTTCTTTTCGTCTTCCTCTCTGTTTCTCTATATATGAATCGTATTTCATATATCTTATCTCTCCATCCAAGTTAAATGGTTTCACTTGATAATGGATAGTAGCTTCTTCTCATAGACTAACAGGTTTGGATTCATCGTTTACAAGTCCATATTTGTTTACATAACTATTAGCTTCCTTTAATGGTGACGGAGATTCAAAGAGAAGAAACTCCACACGTCCTTCACGGTTCAAGGTTTAAGTGCAAATGCTTTCATCTATAGTTACTATTGACAAGCATAGGACTCTTACTGTTCTGATGAAATGAGTGTCATAAATCTTTTCCAGGATGCTAGAAAGGAACATCGATATGTCTCTCAACTCGTGGTAGGACACAAACAGTATTAGCTCTTGCATCAAAGCAGAAGCTGACTTCACGAATTCTTCAGATTTTGATCCTAATCCAGACCCATTTTATGCATTGTTTCTGTCACTTTCTTTATTGTTTGATTGTTGATATACCTTTTCAGGATGAGATTGTGAAGGAAATCAATGAGCCAAACTCTAGATGGAAAGCTGTTTTTAGTGATCGATTTGCTAGCACCACTCTGAGTTTTTTTACATCCATCATCACATCTGCGACTTTATAGGTTCGGAGCTTTTACTCTTTTTCGATGAAGAGAGTAATGCTTATGCTTGTTCCTGAAATTTTACTTTTCTCCTTTTAAAGGGTTCACAACTTTGATGTTTGCAATCTGTAGAAATTTTATTTTGTTCCGGACCTTTCAAGTTATCACCTTTTACATGTTTCATGTTTATGTTTTAATTACACTTTGTTATGGGTGGCTTTTCTTATATTTTTGAACTAAGATTGTGTTGGTATATGTTATTTTGTTTTCACGTTGCAGGGTATCTAAAGCCACAACTCTTAGACAGAAACCTATATAGCTCATTAACGTAACAACATTGATTGGTTTTTGTCGCAACAGCTAATTATAGAAGGTATAACAGCCATTGCCGTATTTTTCAGATGTTTTATGGCCAAGAAAGGGATGGAAACCGAACTCAAGAGGAGGCTTCATGTAGTGTTCCCTATATAGTCTCTTGCTCCAATATTTGTATGCTCTTCACACTTGCTTATTTCTCATGAAATCTAATGCAATTTCTCTTGCCTTATGAAGTTTGTAATGTTTTGTGTTCTTACAAGTTATGTTGGTTTTGTTAACGACCTATGTTTCAGCAGTCACGGGGCAGCTTTTCCTCTTCTGTTTGGTTCTCATCAGAAAGGTAACTCTTATACAGTCTCCTAACGATAAATCTATCAGAATTACTCTGCTTCATATTTTATTCTTTAACAAGTAATGTTCAAATACAAGCAGAAGATGAGAACATATGACTGTATTAGCAATGGGGGCAGGAAATCAATTTACAGAAGTGTACCCTTTTAATGAGTTAGATTAATCCTCAGATGAAAGCTCTGCTTTTGATTCGTCTGAAAGACCAAGACAAATGTTGATCTCGAATTTTATATGCAAGAAACCAAATAAGGTTAATACGCAACTCTTTTCCAGTTAACACTTTCTCTTCTTTTTTTTGTTTAGTTTAACACTTTCTCTTCTCAGATATAGTATTCAGGTGTCGATAGAATTAGCAGAGACCATCCAAAAAGATAGAAGGAGAGTTCAACTTCTTCAATACTGATCGACAAGCAACCATGTTTACATGTACGCATAAACCCATCAAAGCTCGATCTAAAAAAAATAAAATAAATAAATAAATAAACCCCTCAAAGCTGATCACATTAAGCTGGTATCGAGAAAGCGGACCTCTGTGGAAGAAGACATACTGTTCTATTTTAGTCCAGGAATCAATGGAATCAAACTCAGACAAGGCTCTAGAGTCCTTAGACTCTGGTATACTGCCCGGAGAAAAACACTGCTTCTGAAACTGTGGACGCTTGGAGATAATAGCTTCTGATGATCATTTGTGTCAGTTTCTTTTGGTGACTGCCAAAATGACTCTTACCTTGATGATTTTCCACAATATGGATTGGTATTTATTAAGAAAGAATAAATAGGTTGGCAAAGTAAGTAATTGTATGAAGCTAGCAACTATAAACTCTCAATAAAAACCAACTGTAATCAACATATTCTTCGGAAATTAGATAGTTAAAGCAAATAAGAGCAATTATTTTTAAACAACAGAACCGCTAGCCCTATGTTTTATTTATAGATAAACATTTTATACTTTTTAATAATTGAAATATTTTAGATCAATCAAATTTAAACATTCAATCCAGCAAATCATTTAAAATGAACTTATAAACTAAAAAAAATCACTTTTATTGGAACAAAATACTCGCTTTCTCATAAGAAAACAAAATAATTTAATATACATATAAAATTAGTTTGCAGGTTACAAGTGTCAGTAAATCATAATTCAGAGAACTCCTCCATTTAATTGTTAGTAAAAAAATAGATATTAGCTGAATTTAAAAAATAAAAAACAAATGTAATGATTTGTAAATTTGAAACTTTACAAAAAATACCAAACAAACAAGATAAATGATTAACTGAATCACATTTAATGTTTAATTAATTCTTTATTCAACCTTATCATCATTATATATATGTATATATATTAATTTTAATATTACAAATAATAAAATTTATTAATTTCATATTAACTATTCAATAAAACAAGACAAAAGAAAATGCCTTCAAATTCGTCTGACAATTTTATATCTATAAGTAAGGGGTGTTCAATCCGGTAAAACCGAACCATACAAAATAGAAAAAGTGCTTTGGATTTGTTTGGACCGAATAAACTGAATAGATGTTATTTTTTAGAAACCGCAGTATATGAATATGGTTTGGTATATAAACCGATCAAACAGAAGAAAATACTAGTATACATATATGTAGATTTTGTAATAATATTAATAATATATACATAATTTCTTTTATTAATATGATATTCAAATTCAAAAACTGATTTTTTTTATTTTTTATTTTGTTTTAAGTTAAAATGGTTATTCTGTTATCCTCATTTTTATCAAACTAAATAAAAAATATTTTATTTTTATTGATAACTCTTTGAGCTGAAATATAATTATATAATAATATTATGTTATTATTACTGTCTCTTATTTTGGTATTTAAATAAAACATAAAATATTGTATTTAAAAGTTAGACTGAAGAATATATATTTATGTTTTAATAAATTTGATTTATGTTCAAATAATCTAATTTTTTATGGAAATATATTCTATTTAAATATTATGATATTATTACTTTCTAACTCTTATTTTGAAATTTAAATAAAACAGAAATTATTATATTTAAAAATTAGACTAATGAACATATATATGTTTTAATAAACCTGATTTTTATTCATATAATCTAAAATTTGTTATAAATATATATAGTTATTTTGTTTTTGGAATAAAACCGAATAAAAAAAATCGATGGTATATAAACCGAACCAAACCAAATTAGATATAGTTCTAATATGGTAGCTAATTTCCATAAACCGAAATACCGAAAAAATGATAAAACCAAACCGAAATCGTAACGAAATCCGGATTGAACAGACCTATCTATAAAAGATCTAAATATATATATTTATACAAATACAGTCGATAGAATAGTAATTGTATTTATAATTCAGCTTAAGCTTATAAACAACTTTTATCACTCTCTGTCGGGAACCGGTTCCCTCTTTTGAGCTAACTACATTCATGGGAAAGAAAATACTAAACAATATTTTAAATTGTCTGCTAGACGATGAATGAATAAGTCGCAGGGCGTGTTCATTTTTGATAACAAGGTGCACAACTATCTTTCTCCAATATGTCTGTCTATGATCCATATTTGAGTAAGTGTTTACGTTTACTATTTGCTAAAATCACACACACTATGCTATTTTTTGAACAGAAAATGTTAATTGCTTGCAGGGGACCTGTTATGAAGCTCTATCTTTGGGACCAAGCTGCTTTTGATTTTTGCAAGAAGTTCAGGTCTTCTGAGAACACTCCTTCCATAATTTTGGTGACTACTGTTTACCCCAAGCGTTTAGGAGGTATGCATTTTTACTTTCTCAAACTGAGTAGCACCTTACATTTCCTAAGTTCTCTATCATTTAATAACCACATATGTTTTAGATGTGTGTCGTCTCAGTCGTTTAGGTTTGCTCATTGTAGTTGTTTAATTTAAAAANNNNNNNNNNNNNNNNNNNNNNNNNNNNNNNNNNNNNNNNNNNNNNNNNNNNNNNNNNNNNNNNNNTATATATATATATATATATATATATATATATATATATATATATATCTACTTGAGAGTGAAAGCACAAATATACCACTTACAGGTCTAATTATGTCTCATTTGCCTTTGTCTATTACAACATAACACATCAGGCACACTTGCTCTCACATCCATGGCCTCTTCACGTATGTTTTTAGACTATGATGTCCAACCAACTAAGGAGTATATCGGCTGGTATGTTTTCACTATTTAGTATATAATAACTTGGAGACTTGGCAGTTAATATCAGTTCTGAGTAATGAATTGTTTTCTCACGTTGGGAAGTAATCCAGATATTGCCAAGCAGGTCAATGCAGATGTTGTTACAAAAACTGAGACGCTGACTATTGGAGAAATATTCTCCTACATGAAGCATGAAGCTGCAAAGGTATAAGAAAATATATATGTTTGTAATTTATTTTTAAGAAAATACTTTAATTGTTTACACATTGTCTAAGGATGCATTCTTTGTGTTCACTGCGACAATCGATGATGTTGTCCAAGACTCTCCTTGGTATTATATAGCATGCGGTGGTGCAAAACAAAGGCAACCAAAGGGCCAACCTCCCTAATGTGCGCAAAATGTGGCAAGAACGATGTCGCCGGTGAACCACAGTATGAGCTCTAATCTCTTGATTCAACTTTTAGTTTAGCGGTTACTAACTACTCATATTTTAATGGCAGGTACCTAGCTAAGATTTCTGTTTATGACAAGAATGATGAGGCTGTTTTTGTTCTCCTTGGCGATGCTGGCCGTGAGTTAACAGGGAAGCATGCATCAGAGCTGGTTAGCAATTATTTTGAGGTGCACACTCATGATCTAATTTTTATTATTATATACTACTCTCGAGTTGTTTTTCTTCACACACTTGCGTACTCAGCTGACAAGAATATTACACATTTCAGGGCAATGGAAATAAAGAAGATGGTCTTGAGGTGCCTGTTCCGCAAGCTCTGCTCAACACCATATGACAAACACACAAGTTCAGTATAAAGGTGACTGAGCACAATCTATCTGGCAGGACACGAGTGATAACCGTTACGAAGGTCCTGTCTCCATCTGCTCCACCATATATACCAGAACCAGTAGCATATCCAATTATTGGCTCCGCTAATGAGATATTGGCAACTGGGGATGATGCTTCTGAGCCATTCAAAATCCCTCAAG

**Supplementary Data 2:** The gDNA sequences of *BjCLV1a* and *BjCLV1b*

*BjCLV1a* (3043bp)

ATGGAGATGAGACTTCTGAAAACCCACCTTCTGTTTCTCCATCTTCATTACGTTATCTCGATTTCGCTTCTATGTTTCTCACTATGCCTCGCTTCCACTGACATGGACCATCTCCTCAACCTCAAATCCTCCATGGTCGGCCCCAACGGCCACGGCCTCCACGACTGGGTTCACTCCACTTCCCCCTCAGCTCACTGTTCTTTCTCCGGCGTTTCATGCGACGGCGACGCTCGTGTCATCTCCCTCAACGTCTCTTTCACTCCTCTCTTCGGAACCATCTCGCCAGAGATTGGGATGCTGAACCGTCTTGTGAATCTCACGTTAGCTGCTAATAACTTCTCCGGTATGTTGCCGCTGGAGATGAAGAGTCTCACTTCTCTAAAGGTTCTCAACATCTCCAACAACGTGAACCTCAACGGGACCTTCCCCGGAGAGATTCTCACTCCCATGGTGGACCTCGAAGTCCTCGACGCGTACAACAACAACTTCACAGGCCCATTACCCCCGGAGATCCCCGGGCTCAAGAAGCTGAGACACCTCTCTCTCGGAGGAAACTTCTTAACCGGAGAGATCCCAGAGAGTTACGGAGATATCCAAAGCTTGGAGTATCTTGGCCTCAACGGAGCCGGACTCTCCGGTGAATCTCCGGCGTTCTTGTCTCGCCTCAAGAATCTTAAAGAAATGTACGTCGGCTACTTCAACAGCTACACCGGCGGCGTTCCGCCGGAGTTCGGTGAATTGACAAACCTAGAGGTCCTCGACATGGCGAGCTGTACTCTCACAGGAGAGATTCCGACAACACTAAGTAATCTAAAACATTTGCACACTTTGTTTCTCCACATCAACAACTTAACCGGAAACATCCCACCAGAACTCTCCGGTTTAATCAGTTTAAAATCTCTAGACCTCTCAATAAACCAGCTAACCGGAGAGATTCCTCAGAGCTTCATCTCCCTAGGAAACATCACTCTCATCAACCTCTTCAGAAACAATCTCCACGGGCCGATACCGGACTTCATCGGAGACATGCCGAACCTCCAAGTCCTCCAGGTGTGGGAGAACAACTTCACGCTAGAGCTACCGGCGAATCTCGGCCGGAACGGGAATCTGAAAAAGCTCGACGTCTCTGATAACCATCTTACCGGACTCATCCCCATGGATTTGTGCAGAGGCGGGAAGCTGGAGACGCTCGTGCTCTCCAACAACTTCTTCTTCGGCTCGATCCCTGAGAAGCTAGGTCAATGCAAATCGCTAAACAAGATCAGAATCGTCAAGAATCTCCTCAACGGTACGGTTCCGGAGGGATTATTCAATCTACCGCTCGTAACGATCATCGAGCTCACCGATAACTTCTTCTCCGGGGAGCTTCCGGGGGAGATGTCAGGCGACGTTCTCGATCATATCTACTTATCTAACAATTGGTTTACCGGTTTAATCCCCCCGGCTATCGGTAATTTTAAAAATCTACAAGATTTATTCTTAGACCGGAACCGGTTTAGCGGGAATATCCCGAGGGAAGTTTTCGAGTTAAAGCATCTAACGAAGATCAACACGAGTGCTAACAACCTAACCGGCGACATCCCTGACTCGATCTCTCGCTGCACTTCCTTAATCTCCGTCGATCTCAGCCGTAACCGAATCGGCGGAGATATTCCTAAAGACATCCACGACGTGATTAACTTAGGAACTCTAAATCTCTCCGGGAATCAACTCACCGGCTCGATCCCGATCGGAATCGGGAAGATGACGAGCTTAACCACTCTCGATCTCTCCTTCAACGACCTCTCCGGGAGAGTCCCACTCGGCGGCCAGTTCCTAGTCTTCAACGACACTTCCTTCGCCGGAAACCCTTACCTCTGCCTCCCTCACCACGTCTCGTGCCTTACGCGTCCGGAACAAACCTCCGATCGTATCCACACGGCTCTCTTCTCTCCGTCGAGGATCGTTATCACGATCGTCGCAGCGATCACGGCGTTGATCCTTATCAGCGTCGCGATTCGTCAGATGAACAAGAAGAAGCACGAGAGATCTCTCTCCTGGAAGCTAACCGCCTTCCAAAGACTCGATTTCAAAGCGGAAGACGTCCTCGAGTGTCTCCAGGAAGAGAACATAATCGGCAAAGGCGGAGCGGGGATCGTCTACCGCGGATCCATGCCGAACAACGTCGACGTCGCGATCAAACGCCTTGTAGGACGCGGAACAGGGAGGAGCGATCACGGATTCACGGCGGAGATACAGACGCTAGGGAGAATCCGCCACCGTCATATAGTGAGACTCCTCGGATACGTGGCGAACAAGGACACGAACCTGCTTCTCTACGAGTACATGCCTAACGGGAGCCTCGGGGAGCTTTTGCACGGATCTAAAGGAGGTCATCTTCAGTGGGAGACGAGGCACAGAGTAGCCGTGGAAGCGGCGAAAGGACTGTGTTATCTTCATCATGACTGTTCGCCGTTGATCTTGCATAGAGACGTTAAGTCCAATAACATTTTACTGGACTCTGATTTTGAGGCCCATGTTGCTGATTTTGGGCTTGCTAAGTTCTTAGTGGACGGTGCTGCTTCTGAGTGTATGTCTTCGATAGCTGGCTCCTATGGATACATCGCTCCAGGTTAGTTTAAACATGTTTTAAATAACAAATAATATGTATAAAACTAACTATTGTTTGTTTTGGTTTTGAATTTTGATAGAGTATGCTTACACTCTCAAAGTGGATGAGAAGAGTGATGTGTATAGTTTCGGAGTGGTGTTATTGGAGCTGATAGCTGGGAAGAAACCAGTTGGTGAGTTTGGGGAAGGAGTGGATATAGTGAGGTGGGTGAGGAACACGGAGGGTGAGATACCTCAGCCGTCGGATGCAGCTACTGTTGTGGCGATCGTTGACCAGAGGTTGACTGGTTACCCGTTGACTAGTGTGATTCACGTGTTCAAGATAGCGATGATGTGTGTGGAGGATGAGGCAGCGACAAGGCCGACGATGAGGGAAGTTGTGCACATGCTCACTAACCCTCCCAAGTCCGTCACTAACTTGATCGCCTTCTGA

*BjCLV1b* (3154bp)

ATGGAGATGAGACTTTTGAAAACACACCTTCTGTTTCTGCATCTTCACTACGTTATCTCGATTTTGCTTCTATGTTTCTCACCATGCTTCGCTTCCACTGACATGGACCATCTCCTCAACCTCAAATCCTCAATGACTGGTCACAACGGACACGGTCTCCACGACTGGGTTCACTCCACTTCTCCCACGGCTCACTGTTCTTTCTCCGGCGTCTCATGCGACGCCGACGCTCGTGTTGTCTCACTCAACGTCTCTTTCACTCCTCTGTTCGGAACCATCTCACCGGAGATTGGGATGCTGAACCGTTTGGTGAATCTGACGTTAGCCGCGAATAACTTCTCCGGTAGGTTGCCGCTGGAGATGAAGAGTCTCACTTCACTAAAGGTTCTCAACATCTCCAACAACGTAAACCTCAACGGAACCTTCCCCGGAGAGATTCTCACTTCCATGGTCGACCTCGAAGTCCTCGACGCCTATAACAACAACTTCACTGGGCCGTTACCGCCGGAGATTCCCGGGCTGAACAAGCTCAAACACCTCTCTCTCGGAGGAAACTTCTTCACCGGAGAGATTCCCGAGAGTTACGGAGATATCCAAAGCTTGGAGTATCTCGGCCTCAACGGAGCCGGACTCTCCGGCGAATCTCCGGCGTTCTTGTCACGTCTCAAGAATCTCAGAGAAATGTACGTCGGCTACTTCAACAGCTACACCGGCGGCGTTCCGCCGGAGTTCGGCGAATTAACGAAGCTAGAAATCCTCGACATGGCGAGCTGTACTCTCACCGGAGAGATTCCGACGACACTGAGTAATCTGAAACACTTGCACACGCTGTTCCTCCACATCAACAACTTAACCGGAAACATCCCACCAGAACTCTCCGGTTTAATCAGCTTAAAATCTCTCGATCTATCAATTAACCAGCTAACCGGAGAGATTCCTCAGAGCTTCATCTCTCTGGGAAACATCACTCTCATCAACCTCTTCAGAAACAACCTCCACGGGCCGATACCGGAGTTCATCGGAGAAATGCCGAACCTCCAAGTCTTCCAGGTGTGGGAGAACAACTTCACGTTAGAGTTACCGGCGAATATCGGCCGGAACGGGAATCTGAAAAAGCTCGACGTCTCTGAAAACCATCTCACCGGACTCATCCCCGTGGATTTATGCAGAGGTGGGAAGCTGGAGGTTCTGGTACTCTCCAACAACTTCTTCTTCGGCTCCATCCCGGAGAAGCTAGGTCAATGCAAATCTCTAAACAAGATCAGAATCGTCAAGAATCTCCTCAACGGGACCGTTCCGGCGGGACTATTCAACTTACCACTCGTTACAATCATCGAGCTCGCGGATAACTTCTTCTCCGGGGAACTTCCTACGGAGATGTCCGGCGACGTTCTCGATCATATCTACCTATCTAACAACTGGTTTACCGGTTTAATCCCTCCGGCTATCGGTAATTTCAAAAATCTACAGGATCTGTTCTTAGACCGGAACCGGTTTAGCGGGAATATTCCGAGAGAAGTTTTCGAGCTGAAGCATCTCACGAAGATCAACACGAGCGCTAACAACCTAACCGGCGATATCCCTGACTCATTCTCGCGATGCACTTCCTTAATCTCCGTCGATCTCAGCCGTAACAGAATCGGCGGAGATATCCCGAAAGACATCCACGACGTGATTAACTTAGGAACTCTCAATCTCTCCGGGAATCAGCTCACCGGCTCGATCCCGATCGGAATCGGGAAGATGACGAGCTTAACCACTCTCGATCTCTCCTTCAACGACCTCTCCGGGAGAGTACCACTCGGCGGTCAGTTCCTAGTCTTCAACGACACTTCCTTCGCCGGAAACCCTTACCTCTGCCTCCCTCACCACGCCTCCTGCCTAACGCGTCCAGGACAAACCTCTGATCGCATCCACGCGGCGCTGTTCTCTCCGTCGAGGATCGTCATCACGATCATCGCGGCGATCACGGCGTTGATCCTCATCAGCGTCGCGATCCGTCAGATGAACAAGAAGAAACACGAGAGATCCCTCTCGTGGAAGCTAACCGCCTTCCAGCGACTCGATTTCAAGGCGGAAGACGTCCTCGAGTGCCTTCAAGAAGAGAACATAATCGGCAAAGGCGGAGCGGGGATCGTCTACCGCGGATCCATGCCGAACAACGTAGACGTCGCGATCAAACGGTTAGTTGGACGCGGAACGGGGAGGAGCGATCACGGATTCACGGCGGAGATTCAGACGTTAGGAAGAATCCGCCACCGTCATATAGTGAGACTACTCGGATACGTGGCGAACAAGGACACGAACCTGCTTCTATACGAGTACATGCCTAACGGGAGCCTCGGCGAGCTTTTGCACGGGTCTAAAGGCGGTCATCTTCAGTGGGAGACGAGGCACAGAGTAGCCGTTGAAGCGGCGAAAGGACTGTGTTATCTTCACCATGACTGTTCGCCGTTGATCTTGCACAGAGACGTTAAGTCCAATAACATTCTCCTGGACTCTGATTTCGAAGCCCATGTTGCTGATTTTGGGCTTGCTAAGTTCTTAGTGGACGGTGCTGCTTCTGAGTGTATGTCCTCAATAGCTGGCTCCTACGGTTACATCGCTCCAGGTTAGTTTAAACGTGTTTTATATAACAAACTATGCCATGTTCTTTAAATGTTACTGAGACTAATCATATGTTAGAGTTTGTTGTCCGTTTTATTGTCGTATAGCTGATTACTATGATTTGTGACACACAAATGAATGTGACTTTAACAACTAAAATATGTACTAATCATTGTTTGTTTTGGGTTTGATAGAGTATGCATACACTCTCAAAGTAGACGAGAAGAGTGATGTGTATAGCTTTGGAGTGGTCTTGTTGGAGCTGATAGCTGGGAAGAAACCGGTTGGTGAGTTTGGGGAAGGAGTGGATATAGTGAGGTGGGTGAGGAACACGGAGGGTGAGATACCTCAGCCTTCGGATGCAGCTACTGTTGTTGCGATCGTTGACCCGAGGTTGACTGGTTACCCGTTGACCAGTGTGATTCATGTGTTCAAGATAGCGATGATGTGTGTGGAGGATGAGGCCGCGTCAAGGCCTACGATGAGGGAAGTTGTGCACATGCTAACTAACTCTCCTAAGTCCGTGACTAACTTGATCGCCTTCTGA

**Supplementary Data 3:** The sequence of Copia-LTR RTE1 (4961bp)

TGAGAACAAGAGGAGACAGAGAGAATTTTAATAAAACTTCCTTTTGTATTCACTTTCTCAAAACGTTTCTTTACAAGATAATCGAGTATATATACAACCTCAAGATAAACCCTAGTTCCCTTAACTTTGGACCACTCAACACGTGTCTTCGTCACACACACTCTACACCTTGCAAGGCATCGTAACGGCTAGACAAAGCTATTCCACAGTTTACTCCAGCTGGTGCATTTGTCTTCTTGTTCTGTACTTGTACGAACTCTTCATTACTCTGTTTCAGAGTTTGTCTTTGGGCCAGCATACTTCCTTCAGTTAATGGACCAGTCGCTGATATACCAAGGCCCATCATGGCTTGTTTACTGTCATCCCCCCGCAAACTGATGATGGGTGGAGCACCAACACTCAGTTTGGCTCTGAGACTCTGAAAGGAATGGCGTGGGAGAGATTTAGTGAAGATATCTGCAAGTTGCAGTGCAGAAGGAATGTGCTGAACCTCCAAGACACCAAGAGCCACCCGCTCCCTAACATAATGGTAATCCGTTTCAATATGTTTGGACCGACCGTGAAGAACCGGGTTTGCTGTAAGATAAACTGCAGAGAGGTTATCACAGTGAAGAACAGCCGGCTTTGGTTGTTTGATCTTCATATCACGCATCACAAACGAAATCCATGTTAACTCAGTCCGTCTTGCAGAGCTCGATATTCAGCCTCAGTGGACGACCGCGAGACAGTCGGTTGGCGCTTTGCCGACCAGGAGACAATGTTAGAACCAATCATGACACAAAAACCACCAGTCGACCGTCTTGTCTCCCTACACCCTGCCCAGTCACTATCACTATACGCGACAACATCCAAACCAGAGTTCTTACGGAAGTAAATCCCCATGGATGAAGTACCACGAAGGTAGCGAAGAACCCGCTTCAACAAATGAAAGTCAGCTTCAGTGGGAGAATGCATTCTCTGACAAATGAAATTCACAGCAAACTGAATGTCGGGTCGAGTGATAGTGAGATACTGTAACTTCCCAGCAAGACTCCGAAAGTAGCTAGGCTCAGGGAACGGCCGAGTATCTTCATAAACTTGATCGAGTCGCAAAGGGAGAGGTGTATGAACAGGATTACACGCAGACATATTGGCATGGTGAAGAATCTCTTCAGTGTACTTGGATTGAGAGAGGAAAAGACCAGAATCTGTAATCTGAGCCTGAACGCCCAAGAAGTAGTGCAGATTACCCAAATCTTTCATGGAGAATCGGCTACTGAGCTCAGTGATAAGGGACTTGAACAAGGATGGATTTGAGCCAGTGAGAAGTATATCATCCACATAAAGGAGGAGAACTAATGTGTCGCCATTGTGATGATAAACAAAGAGAGAGGGATCAGCTTTACTGCAGACAAAGCCATACTCAAGAAGGAAGTTACTAAATTTATCAAACCAAGCCCGTGGAGCTTGTTTTAAACCGTACAGAGCCTTCTTGAGAGAGCACACATGATGAGGTTTAGTGCAATCTTCAAAGCCGGGTGGTTGTAGCATATAGACTGCTTCTTGAAGATCTCCATGAAGGAAAGCATTTTTAACATCAAGCTGAGTGATGTTCCAGTTCTTTGAAGCAGCTACAGCAAGAACTATCCGAATGGTAGAAGTCCTAACCACTGGACTGTATGTCTCTGTGAAATCTACTCCTTCTTCTTGATCAAACCCCTTAGCAACTAACCGCGCTTTAAGTTTATTGACTGTCCCATCTGCGTTAAGCTTCACTGTAAAAACCCATCTGCAACCAAGAACATTCATAGTTTCTGTAGCTTCCGTTAAAACCCAAGTGTGGTTCTGATAAATCGAGTCTAGTTCATCCAACATTGCTTGACGCCATCCAGGGTGCGCCAAAGCTTCAGATATAGTCTGTGGTAAGGAGGGAATCGTCTTGCTTGTGACCAGAGCGTAACGTGGATTAGGTTTGCTAATACCAGCTCGTGATCTCGTCATCATAGGATGACTAGGCGCTGCTACGTCTGGTGAGGCACGAGGAGGTGATTGAGCAACCTCAGGAAGACTTGTGGTGGATACGTCGCCTTCAGACAGCTCGAAGTCTTCCTGCCCATGATCAGAACGAGATGGGGTTGCAATAGGCGTATCTTGACCCGTGGTGATAGGAGTATCCTGTTGAGGATACTCTGTAACTTGAGGACACTCTGTAACTTGAGAAGGTACTGTAGGCAGATAGACCGGAGGAGCTTCAAATGCAGTACCATCTTGCCAAGCTTTAAGCAAACCAGAGTTGTGTTTCACAAGCAGATGTTTATACTGTCCAGTGAATGGGAAGGTCTGCTCATCGAAGATAACGTGGCGAGAGATGTAAACTCGACCTGTTGGTGGATGCAAACACCTGTAACCTTTGTACTGAGGATGATAACCGAGGAAGACACACTGTAAGGATCGGGGCTCGAACTTGTGTTGAGCATAGGGACGCAAGCACGGATAGCACGCAGAGCCAAAGATTCTGAGAAATGAGTAGTCTGGTGCTTTACTAGAGAGAACCTCTGAAGGGCTTTTGTTGTTCAGAACAGAAGAAGGGATCATGTTGCTGATGAAGTTAGCAGAGTAGAAGGCCTCAACCCAATATTTAAGAGGAGTGTGACTTTGAAATAACATAGAAAGACCCAATTCAGTAAGATGTCTATGTTTTCTCTCAGCAATACCATTTTGTTGAGGAGTTGAAGGACATGAAACAAGATGAACAATCCCTTGATCTCTGAGATGATTGCGAAACCTTGTGCTCATAAACTCACCACCCCCATCGCTCTGAAAGACTTTCAGTTTCTTGTTAAACTGATTTTCGATTTGTTTTTGAAAGACAATGAACACATCACAAAAATCAGACTTGGCTTTAAGAGGTATTATCCATGAGTAGCGACTAAAGTTGTCAACAAAGACTGCATAATACTTGAACCCTTGAACTGATACAACCGGAGAAGGACCCCACAGATCACAATGTATCCGATCTATGGGTTCCTTGACACTAGACTCTGAAATAAAAAAAGGAAGCTGACTGCTCTTTCCCATCTGGCAAGGTCCACAAACGGGAGAAATGCTGCTCTTATTGACTATGATTGCCTTGCTTTGCTTGAGGAGCTGAAGAATCTGGAAGTTCGCGTGCCCAAGTCGCTGATGCCACACCATATCACTAGCTGCACACTGACGATTGGAGAAGTAAGCCGCAAACTCCTTCTTCTCCAGCACATACAAGCCTCTATTTCGAGTTCCCTTTGTCACCACCTTCTGAGCATCGAGGTCGATGACATAGACAGCATTAGAGTCAAAGAACACCCCACAGGGGTAATCATCACACAGTTTGGATACAGATAATAGTGATTTTTGCATTGAAGGACAGACAAGAACATCGTTTAGAGGAAGGCTACCTGTCTCAGTGGTGAGCGTTGTGGAACCAACGTGGGTAATCGGAAGGAAATTGCCATCTGCCACCATAACATGCTCAGAACCATTGTATGGAGTAGTGTTCTGCATCTGAGTTGTATTAGGAACCATGTGAGCAGTGGCACCTGAGTCAGCTATCCATTCCCTGCCACTAGGATCAGAGACTGGAAAAGCGGACAGAGCATGAGGAACATCGTCACTCTGATAAGAGTTATCAAAGCGGTTCCAGCAACGAAGAGCGGAGTGGCCGGTGCGGCCACAGATCTGACACACAGGGCGAGAGTTGTTGTTGTTATTGCCACCACCAGAGTTGCTGACTTGTTGAGTGAAACCTCTTCCACGAGTTGAGAAGCCACCACGACCAGAATAGCCACCGCGACCACGAGAATAATTATTGCTTCGTCCTCTGTTCGAGTTGTTGTAGTACCCTCGGTGAGTCTGGAAAGCCATGTTGGGAGTAACATCTGATGTTGCTTCATACGACTGAAGACGAGCGTCAAAACCAGAGACATCAGAAATCACATCGTTCAAAGTCGGAGGAGGGATTCGAGTCATGGAGCTCTGAACGACAGTGATAATTGGATCATATTCTCTTCCAAGGCCATTGATAAATGAGAAGATCTTCATGGATTCCTCTATAGGCTTCCCAATGGAGCTCAACTTGTCACAAATGGCTCGAAACTCACGACAGTAGGTAGTAAAGTCTTTACCTTTCTTAGTCATCAACTGGAGAGACCGTCTGAGCTCAAACTCACGAGCAATAGAACTCTTGTTGTAGTTGTCAGCTAACGATAACCAGACATCACGAGATGTAGATAGACCATGTACTGTACCAAGGACTTCCTCAGTAAGTGTTCCAAAGATCCAAGAAATGACGAGTTGATCCGTACAAGTCCAAGCTTCAAAGCGAGGATTTGGGGCTTGAACGTTGACGTTGTCGACCACGGTTGTGACTAGCTCCGGCGGAGGTGGAATCTGACCAGTAGCAAAGCCCAACAGCTTCTGACTGCGAAGAAGAGACTCCATTTGAGTCTTCCATAGGAGATAGTTGCTGTCACTCAGTTTAATGGTAACAGAACTTGTAATGTGAACACTGGTGGGAAAAGGGTATGGATCTTGCTCAGCCATGACAGCACCTGTTAGGGTTAAATGAGCTCTGATACCATGAGAACAAGAGGAGACAGAGAGAATTTTAATAAAACTTCCTTTTGTATTCACTTTCTCAAAACGTTTCTTTACAAGATAATCGAGTATATATACAACCTCAAGATAAACCCTAGTTCCCTTAACTTTGGACCACTCAACACGTGTCTTCGTCACACACACTCTACACCTTGCAAGGCATCGTAACGGCTAGACAAAGCTATTCCACAGTTTACTCCAGCTGGTGCATTTGTCTTCTTGTTCTGTACTTGTACGAACTCTTCATTACTCTGTTTCAGAGTTTGTCTTTGGGCCAGCATACTTCCTTCAGTTAATGGACCAGTCGCTGATATACCAAGGCCCATCATGGCTTGTTTACTGTCATCGGC

**Supplementary Data 4:** The cDNA sequences of *BjMc1* and *Bjmc1*

*BjMc1* cDNA (3171bp)

ACATGGGGACGCTAACTTCTTATTCTCTCTCTCTTTCTCTGAGAAGACAGCTTTGAAGAAAGAAAGAAAAAATGGAGATGAGACTTTTGAAAACACACCTTCTGTTTCTGCATCTTCACTACGTTATCTCGATTTTGCTTCTATGTTTCTCACCATGCTTCGCTTCCACTGACATGGACCATCTCCTCAACCTCAAATCCTCAATGACTGGTCACAACGGACACGGTCTCCACGACTGGGTTCACTCCACTTCTCCCACGGCTCACTGTTCTTTCTCCGGCGTCTCATGCGACGCCGACGCTCGTGTTGTCTCACTCAACGTCTCTTTCACTCCTCTGTTCGGAACCATCTCACCGGAGATTGGGATGCTGAACCGTTTGGTGAATCTGACGTTAGCCGCGAATAACTTCTCCGGTAGGTTGCCGCTGGAGATGAAGAGTCTCACTTCACTAAAGGTTCTCAACATCTCCAACAACGTAAACCTCAACGGAACCTTCCCCGGAGAGATTCTCACTTCCATGGTCGACCTCGAAGTCCTCGACGCCTATAACAACAACTTCACTGGGCCGTTACCGCCGGAGATTCCCGGGCTGAACAAGCTCAAACACCTCTCTCTCGGAGGAAACTTCTTCACCGGAGAGATTCCCGAGAGTTACGGAGATATCCAAAGCTTGGAGTATCTCGGCCTCAACGGAGCCGGACTCTCCGGCGAATCTCCGGCGTTCTTGTCACGTCTCAAGAATCTCAGAGAAATGTACGTCGGCTACTTCAACAGCTACACCGGCGGCGTTCCGCCGGAGTTCGGCGAATTAACGAAGCTAGAAATCCTCGACATGGCGAGCTGTACTCTCACCGGAGAGATTCCGACGACACTGAGTAATCTGAAACACTTGCACACGCTGTTCCTCCACATCAACAACTTAACCGGAAACATCCCACCAGAACTCTCCGGTTTAATCAGCTTAAAATCTCTCGATCTATCAATTAACCAGCTAACCGGAGAGATTCCTCAGAGCTTCATCTCTCTGGGAAACATCACTCTCATCAACCTCTTCAGAAACAACCTCCACGGGCCGATACCGGAGTTCATCGGAGAAATGCCGAACCTCCAAGTCTTCCAGGTGTGGGAGAACAACTTCACGTTAGAGTTACCGGCGAATATCGGCCGGAACGGGAATCTGAAAAAGCTCGACGTCTCTGAAAACCATCTCACCGGACTCATCCCCGTGGATTTATGCAGAGGTGGGAAGCTGGAGGTTCTGGTACTCTCCAACAACTTCTTCTTCGGCTCCATCCCGGAGAAGCTAGGTCAATGCAAATCTCTAAACAAGATCAGAATCGTCAAGAATCTCCTCAACGGGACCGTTCCGGCGGGACTATTCAACTTACCACTCGTTACAATCATCGAGCTCGCGGATAACTTCTTCTCCGGGGAACTTCCTACGGAGATGTCCGGCGACGTTCTCGATCATATCTACCTATCTAACAACTGGTTTACCGGTTTAATCCCTCCGGCTATCGGTAATTTCAAAAATCTACAGGATCTGTTCTTAGACCGGAACCGGTTTAGCGGGAATATTCCGAGAGAAGTTTTCGAGCTGAAGCATCTCACGAAGATCAACACGAGCGCTAACAACCTAACCGGCGATATCCCTGACTCATTCTCGCGATGCACTTCCTTAATCTCCGTCGATCTCAGCCGTAACAGAATCGGCGGAGATATCCCGAAAGACATCCACGACGTGATTAACTTAGGAACTCTCAATCTCTCCGGGAATCAGCTCACCGGCTCGATCCCGATCGGAATCGGGAAGATGACGAGCTTAACCACTCTCGATCTCTCCTTCAACGACCTCTCCGGGAGAGTACCACTCGGCGGTCAGTTCCTAGTCTTCAACGACACTTCCTTCGCCGGAAACCCTTACCTCTGCCTCCCTCACCACGCCTCCTGCCTAACGCGTCCAGGACAAACCTCTGATCGCATCCACGCGGCGCTGTTCTCTCCGTCGAGGATCGTCATCACGATCATCGCGGCGATCACGGCGTTGATCCTCATCAGCGTCGCGATCCGTCAGATGAACAAGAAGAAACACGAGAGATCCCTCTCGTGGAAGCTAACCGCCTTCCAGCGACTCGATTTCAAGGCGGAAGACGTCCTCGAGTGCCTTCAAGAAGAGAACATAATCGGCAAAGGCGGAGCGGGGATCGTCTACCGCGGATCCATGCCGAACAACGTAGACGTCGCGATCAAACGGTTAGTTGGACGCGGAACGGGGAGGAGCGATCACGGATTCACGGCGGAGATTCAGACGTTAGGAAGAATCCGCCACCGTCATATAGTGAGACTACTCGGATACGTGGCGAACAAGGACACGAACCTGCTTCTATACGAGTACATGCCTAACGGGAGCCTCGGCGAGCTTTTGCACGGGTCTAAAGGCGGTCATCTTCAGTGGGAGACGAGGCACAGAGTAGCCGTTGAAGCGGCGAAAGGACTGTGTTATCTTCACCATGACTGTTCGCCGTTGATCTTGCACAGAGACGTTAAGTCCAATAACATTCTCCTGGACTCTGATTTCGAAGCCCATGTTGCTGATTTTGGGCTTGCTAAGTTCTTAGTGGACGGTGCTGCTTCTGAGTGTATGTCCTCAATAGCTGGCTCCTACGGTTACATCGCTCCAGAGTATGCATACACTCTCAAAGTAGACGAGAAGAGTGATGTGTATAGCTTTGGAGTGGTCTTGTTGGAGCTGATAGCTGGGAAGAAACCGGTTGGTGAGTTTGGGGAAGGAGTGGATATAGTGAGGTGGGTGAGGAACACGGAGGGTGAGATACCTCAGCCTTCGGATGCAGCTACTGTTGTTGCGATCGTTGACCCGAGGTTGACTGGTTACCCGTTGACCAGTGTGATTCATGTGTTCAAGATAGCGATGATGTGTGTGGAGGATGAGGCCGCGTCAAGGCCTACGATGAGGGAAGTTGTGCACATGCTAACTAACTCTCCTAAGTCCGTGACTAACTTGATCGCCTTCTGAGTTCTGACCCAATCAAAGGCTAATGAAAAAATAAGATTGTGTTGTGTGTAATGATAATATTTTTTGTTTTTTTAAGTGGACATATATCGTTTTAAATTATTGCCGAGTTGGATGTAAAAAAAAAAAAAAAAAAAGA

*Bjmc1* cDNA (2579bp)

ACATGGGGACGCTAACTTCTTATTCTCTCTCTCTTTCTCTGAGAAGACAGCTTTGAAGAAAGAAAGAAAAAATGGAGATGAGACTTTTGAAAACACACCTTCTGTTTCTGCATCTTCACTACGTTATCTCGATTTTGCTTCTATGTTTCTCACCATGCTTCGCTTCCACTGACATGGACCATCTCCTCAACCTCAAATCCTCAATGACTGGTCACAACGGACACGGTCTCCACGACTGGGTTCACTCCACTTCTCCCACGGCTCACTGTTCTTTCTCCGGCGTCTCATGCGACGCCGACGCTCGTGTTGTCTCACTCAACGTCTCTTTCACTCCTCTGTTCGGAACCATCTCACCGGAGATTGGGATGCTGAACCGTTTGGTGAATCTGACGTTAGCCGCGAATAACTTCTCCGGTAGGTTGCCGCTGGAGATGAAGAGTCTCACTTCACTAAAGGTTCTCAACATCTCCAACAACGTAAACCTCAACGGAACCTTCCCCGGAGAGATTCTCACTTCCATGGTCGACCTCGAAGTCCTCGACGCCTATAACAACAACTTCACTGGGCCGTTACCGCCGGAGATTCCCGGGCTGAACAAGCTCAAACACCTCTCTCTCGGAGGAAACTTCTTCACCGGAGAGATTCCCGAGAGTTACGGAGATATCCAAAGCTTGGAGTATCTCGGCCTCAACGGAGCCGGACTCTCCGGCGAATCTCCGGCGTTCTTGTCACGTCTCAAGAATCTCAGAGAAATGTACGTCGGCTACTTCAACAGCTACACCGGCGGCGTTCCGCCGGAGTTCGGCGAATTAACGAAGCTAGAAATCCTCGACATGGCGAGCTGTACTCTCACCGGAGAGATTCCGACGACACTGAGTAATCTGAAACACTTGCACACGCTGTTCCTCCACATCAACAACTTAACCGGAAACATCCCACCAGAACTCTCCGGTTTAATCAGCTTAAAATCTCTCGATCTATCAATTAACCAGCTAACCGGAGAGATTCCTCAGAGCTTCATCTCTCTGGGAAACATCACTCTCATCAACCTCTTCAGAAACAACCTCCACGGGCCGATACCGGAGTTCATCGGAGAAATGCCGAACCTCCAAGTCTTCCAGGTGTGGGAGAACAACTTCACGTTAGAGTTACCGGCGAATATCGGCCGGAACGGGAATCTGAAAAAGCTCGACGTCTCTGAAAACCATCTCACCGGACTCATCCCCGTGGATTTATGCAGAGGTGGGAAGCTGGAGGTTCTGGTACTCTCCAACAACTTCTTCTTCGGCTCCATCCCGGAGAAGCTAGGTCAATGCAAATCTCTAAACAAGATCAGAATCGTCAAGAATCTCCTCAACGGGACCGTTCCGGCGGGACTATTCAACTTACCACTCGTTACAATCATCGAGCTCGCGGATAACTTCTTCTCCGGGGAACTTCCTACGGAGATGTCCGGCGACGTTCTCGATCATATCTACCTATCTAACAACTGGTTTACCGGTTTAATCCCTCCGGCTATCGGTAATTTCAAAAATCTACAGGATCTGTTCTTAGACCGGAACCGGTTTAGCGGGAATATTCCGAGAGAAGTTTTCGAGCTGAAGCATCTCACGAAGATCAACACGAGCGCTAACAACCTAACCGGCGATATCCCTGACTCATTCTCGCGATGCACTTCCTTAATCTCCGTCGATCTCAGCCGTAACAGAATCGGCGGAGATATCCCGAAAGACATCCACGACGTGATTAACTTAGGAACTCTCAATCTCTCCGGGAATCAGCTCACCGGCTCGATCCCGATCGGAATCGGGAAGATGACGAGCTTAACCACTCTCGATCTCTCCTTCAACGACCTCTCCGGGAGAGTACCACTCGGCGGTCAGTTCCTAGTCTTCAACGACACTTCCTTCGCCGGAAACCCTTACCTCTGCCTCCCTCACCACGCCTCCTGCCTAACGCGTCCAGGACAAACCTCTGATCGCATCCACGCGGCGCTGTTCTCTCCGTCGAGGATCGTCATCACGATCATCGCGGCGATCACGGCGTTGATCCTCATCAGCGTCGCGATCCGTCAGATGAACAAGAAGAAACACGAGAGATCCCTCTCGTGGAAGCTAACCGCCTTCCAGCGACTCGATTTCAAGGCGGAAGACGTCCTCGAGTGCCTTCAAGAAGAGAACATAATCGGCAAAGGCGGAGCGGGGATCGTCTACCGCGGATCCATGCCGAACAACGTAGACGTCGCGATCAAACGGTTAGTTGGACGCGGAACGGGGAGGAGCGATCACGGATTCACGGCGGAGATTCAGACGTTAGGAAGAATCCGCCACCGTCATATAGTGAGACTACTCGGATACGTGGCGAACAAGGACACGAACCTGCTTCTATACGAGTACATGCCTAACGGGAGCCTCGGCTGAGAACAAGAGGAGACAGAGAGAATTTTAATAAAACTTCCTTTTGTATTCACTTTCTCAAAACGTTTCTTTACAAGATAATCGAGTATATATACAACCTCAAGATAAACCCTAGTTCCCTTAACTTTGGACCAAAAAAAAAAAAAAAAAAAAAAAAAAAGT
